# Global Proteomic Changes Induced by the Epstein-Barr Virus Oncoproteins Latent Membrane Protein 1 and 2A

**DOI:** 10.1128/mBio.00959-18

**Published:** 2018-06-19

**Authors:** Robert M. DeKroon, Harsha P. Gunawardena, Rachel Edwards, Nancy Raab-Traub

**Affiliations:** a Lineberger Comprehensive Cancer Center, University of North Carolina at Chapel Hill, Chapel Hill, North Carolina, USA; b Janssen Research & Development, The Janssen Pharmaceutical Companies of Johnson & Johnson, Spring House, Pennsylvania, USA; c Department of Microbiology and Immunology, University of North Carolina at Chapel Hill, Chapel Hill, North Carolina, USA; University of Michigan

## Abstract

The Epstein-Barr virus (EBV) oncoproteins latent membrane protein 1 (LMP1) and LMP2A constitutively activate multiple signaling pathways, and both have been shown to interact with cellular ubiquitin ligases and affect cellular ubiquitination. To detect the LMP1- and LMP2A-mediated effects on the global cellular proteome, epithelial cell lines expressing LMP1 or LMP2A were analyzed using label-free quantitative proteomics. To identify proteins whose ubiquitination is affected by the viral proteins, the cells were cultured in the presence and absence of deubiquitinase (DUB) and proteasome inhibitors. More than 7,700 proteins were identified with high confidence and considerably more proteins showed significant differences in expression in the presence of inhibitors. Few of the differentially expressed proteins with or without inhibitors were common between LMP1 and LMP2A, confirming that the viral proteins induce unique changes in cell expression and function. However, ingenuity pathway analysis (IPA) of the data indicated that LMP1 and LMP2A modulate many of the same cellular regulatory pathways, including cell death and survival, cell movement, and actin filament dynamics. In addition, various proteasome subunits, ubiquitin-specific peptidases and conjugating enzymes, vesicle trafficking proteins, and NF-κB and mitogen-activated protein kinase signaling proteins were affected by LMP1 or LMP2A. These findings suggest that LMP1 and LMP2A may commonly target critical cell pathways through effects on distinct genes, with many cellular proteins modified by ubiquitination and/or degradation.

## IMPORTANCE

The Epstein-Barr virus proteins latent membrane protein 1 and 2 have potent effects on cell growth and signaling. Both proteins bind to specific ubiquitin ligases and likely modulate the cellular proteome through ubiquitin-mediated effects on stability and intracellular location. In this study, a comprehensive proteomic analysis of the effects of LMP1 and LMP2A revealed that both proteins affected proteasome subunits, ubiquitin-specific conjugases and peptidases, and vesical trafficking proteins. The data suggest that the effects of these proteins on the abundance and ubiquitination of cellular proteins are in part responsible for their effects on cell growth regulation.

## INTRODUCTION

Epstein-Barr virus (EBV) is an important factor in the etiology of several major malignancies, including endemic Burkitt’s lymphoma (BL), nasopharyngeal carcinoma (NPC), Hodgkin disease (HD), and malignant lymphomas that arise in immunosuppressed or immunodeficient patients (1). The EBV proteins latent membrane proteins 1 and 2 (LMP1 and LMP2A) have both been shown to alter cell growth regulation and are considered the EBV oncogenes. LMP1 was initially identified as a viral oncogene, as it can transform rodent fibroblasts *in vitro* and is essential for B-cell transformation (2). LMP1 is expressed in multiple malignancies associated with EBV, including posttransplant lymphoma, Hodgkin disease, and nasopharyngeal carcinoma (NPC) (1). Many studies have shown that LMP1 has considerable effects on cellular biological properties and gene expression (2). Recent studies indicated that the carboxy-terminal activation domain 2 (CTAR2) is responsible for most of the LMP1-induced effects on cellular transcription through its activation of the canonical NF-κB pathway (3, 4). In contrast, CTAR1 activates the noncanonical NF-κB pathways but induces relatively few changes in transcription (4–6). However, it has profound effects on cellular biological properties and is sufficient for induction of epidermal growth factor receptor (EGFR), activation of phosphatidylinositol 3-kinase (PI3-kinase)/Akt, extracellular signal-regulated kinase (ERK), and rodent and epithelial cell transformation (6–8).

LMP2A is not essential for B-cell immortalization; however, it affects cell signaling and blocks signal transduction from the B-cell receptor (BCR) (9–12). The biological effects of LMP2A are subtle, but intriguing effects have been discerned in transgenic mice in which LMP2A expression enables B-cell survival in the absence of BCR signaling (12, 13). In transgenic mice that express both LMP1 and LMP2A in epithelial cells, LMP2A increased the development of squamous cell carcinoma (14). Additionally, LMP2A activates Akt in B lymphocytes and in normal, primary keratinocytes, resulting in the induction of the nuclear translocation of β-catenin and the activation of cellular promoters that are regulated by β-catenin (15–17). In organotypic raft cultures of the HaCat epithelial cell line LMP2A inhibited differentiation, and in the gastric carcinoma cell line HSC-39, LMP2A induced anchorage independence (18–20). Additionally, LMP2A inhibits the process of differentiation and lumen formation in the MCF10A cell line through inhibition of anoikis and induction of autophagy (21, 22).

Both LMP1 and LMP2A interact with cellular ubiquitin ligases that can affect both the levels and locations of cellular proteins. LMP1 is considered a constitutive member of the tumor necrosis factor receptor family and interacts with the same factors, TRAF1, -2, -3, -5, and -6, TRADD, and A20, most of which have ubiquitin ligase activity through a RING finger domain (23–26). LMP2A interacts with tyrosine kinases and with the NEDD 4 family of ubiquitin ligases (21, 27). Multiple studies have assessed the effects of LMP1 and LMP2A on cellular transcription; however, it is likely that both proteins have profound effects on the cellular proteome that contribute to their effects on cell growth regulation and transformation (28).

To identify proteins and pathways directly and indirectly affected by LMP1 and LMP2A through effects on protein levels, the individual cellular proteomes of cells expressing LMP1 or LMP2A were determined in the absence or presence of deubiquitinase and proteasome inhibitors *N*-ethylmaleimide and MG132, respectively. The use of deubiquitinase inhibitors would stabilize proteins whose ubiquitination is induced by the viral proteins, while the proteasome inhibitors would block their degradation. This approach would tentatively identify proteins that would otherwise be undetectable due to virus-mediated effects on ubiquitination and protein turnover. The ubiquitinated proteins include proteins whose levels are affected by the viral proteins through protein degradation but also would include proteins whose ubiquitination is induced by LMP1 or LMP2A to modulate cellular location or activity. The effects of these viral proteins on the proteome could have considerable functional outcomes in multiple aspects of cellular function, including signaling, endocytosis, and vesicle trafficking.

## RESULTS

**FIG 1:**
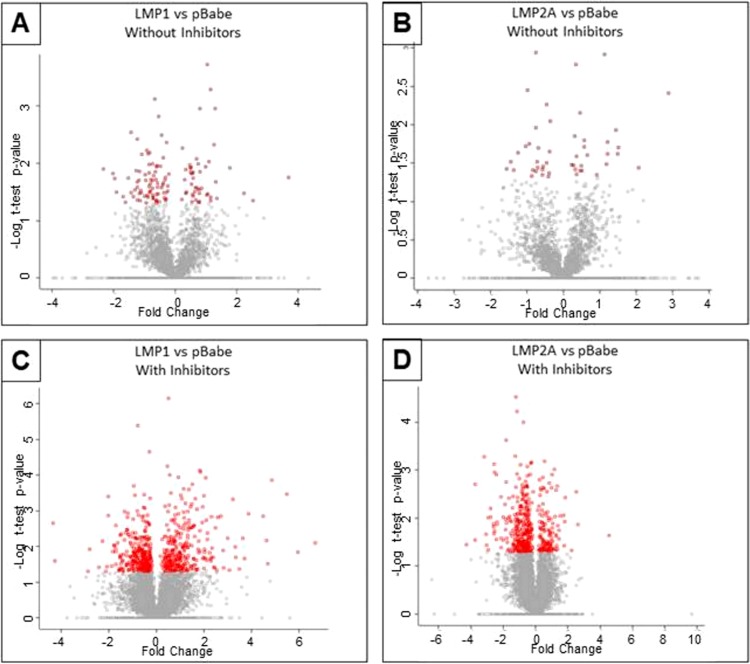
Scatter plots (“volcano plots”) of *t* test *P* values against fold change between LMP1 and pBabe without inhibitors (A), LMP2A and pBabe without inhibitors (B), LMP1 and pBabe with inhibitors (C), and LMP2A and pBabe with inhibitors (D). Proteins with significant *P* values are indicated in red. The number of significantly changed proteins greatly increased with the addition of inhibitors.

Equal amounts of cell lysates from MCF10 epithelial cells expressing LMP1, LMP2A, or pBabe empty vector control, incubated for 6 h with or without inhibitors, were fractionated by basic reverse-phase high-performance liquid chromatography (RP-HPLC) and were analyzed using LC-tandem mass spectrometry (LC-MS/MS). Two biological replicates with two technical replicates of each were analyzed using MaxQuant software (29). A total of 7,713 proteins were identified with 95% confidence limits with appropriate effects on abundance and distribution of increased and decreased expression (Fig. 1) in the presence of LMP1 or LMP2A. The addition of inhibitors consistently increased the number of proteins with significant differences from the pBabe control and between LMP1 and LMP2A (Table 1). In the absence of inhibitors, the LMP1-expressing cells showed increased expression of 49 proteins and decreased expression of 78 proteins, while LMP2A affected the abundance of 48 proteins, with 24 decreased and 24 increased. Expression of LMP1 with inhibitors affected 598 proteins (*P* < 0.05) in comparison to the pBabe-containing control cells, with an almost equal distribution of increased and decreased expression. In the LMP2A-expressing cells, 433 proteins (*P* < 0.05) were changed in comparison to the control cells; however, a considerably greater number of proteins were decreased relative to control cells, with 308 decreased and 125 increased. These data suggest that both LMP1 and LMP2A affect the cellular proteome through proteasome-mediated effects on levels of specific proteins. All *P* values and fold changes for each comparison are presented in Table S1 in the supplemental material).

10.1128/mBio.00959-18.1 TABLE S1  Proteomics data set. *P* values, fold changes, and gene names are provided for *t* test comparisons between LMP1 and pBabe without inhibitors, LMP2A and pBabe without inhibitors, LMP1 and pBabe with inhibitors, and LMP2A and pBabe with inhibitors. The results for each *t* test are presented on a separate tab. Download TABLE S1, PDF file, 1.9 MB. Copyright © 2018 DeKroon et al. 2018 DeKroon et al. This content is distributed under the terms of the Creative Commons Attribution 4.0 International license.

**TABLE 1:** Abundance and distribution of significantly different proteins based on LC-MS/MS data^a^

Comparison	Inhibitors			
	LMP1		LMP2	
	−	+	−	+
No. of proteins with *P* < 0.05	127	598	48	433
Increased vs pBabe	49	286	24	125
Decreased vs pBabe	78	312	24	308
Increased 2-fold or more	15	136	9	29
Decreased 2-fold or more	23	70	5	107

^a^ Data reported are numbers of proteins for a given condition (in the presence [+] or absence [−] of inhibitors) that were found to have a significantly different abundance or distribution.

**FIG 2:**
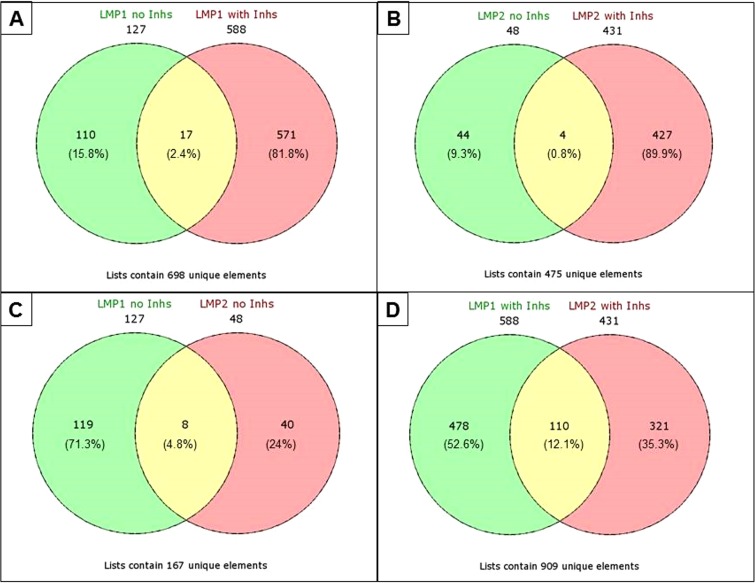
Venn diagrams displaying the overlap in the number of significantly different proteins between treatment groups. (A) LMP1-noInhs compared to LMP1+Inhs; (B) LMP2A-noInhs compared to LMP2A+Inhs; (C) LMP1-noInhs compared to LMP2A-noInhs; (D) LMP1+Inhs compared to LMP2A+Inhs. Proteins common to both groups are listed in Table 2.

Comparison of the individual, significantly different proteins indicated little overlap between groups with and without inhibitors for either LMP1 (2.4%) or LMP2A (0.8%) (Fig. 2A and B; Table 2). The overlapping proteins were consistently affected in the same direction. There was also little overlap between genotypes (Fig. 2C and D), although with the addition of inhibitors the number of proteins in common between LMP1 and LMP2A increased to 110 (or 12%) from 8 (or 4.5%) proteins without inhibitors. This suggests that there is some overlap in their effects on the proteome through the ubiquitin pathway. Common proteins affected included Rab7A, TLR3, ATG2A, mitogen-activated protein kinase kinase 2 (MAP2K2), EIF3B and -3E, ribosomal protein L8 (RPL8) and RPL9, and integrin α5 (ITGA5) (Table 2). Additionally, despite the few overlapping individual significantly different proteins, further assessment of the data by use of the bioinformatics software Ingenuity Pathway Analysis (IPA) revealed substantial overlap in biological functions and signaling pathways between the study groups.

**TABLE 2:** Significantly affected proteins altered in common between groups

Comparison	Significantly different proteins in common between groups
LMP1-noInhs vs LMP1+Inhs	AARS, CA2, CALD1, CCDC47, CNN2, CSK, EIF4A1, ETHEd1, FARSB, HEBP2, LAMB1, MTHFD1, MVP, MYBBP1A, SARG, TBCE, TPD52L1
LMP2-noInhs vs LMP2+Inhs	CHTOP, IGF2BP3, PML, RAB7A
LMP1-noInhs vs LMP2-noInhs	CS, EHD4, GARS, IAH1, NDRG1, SORD, SRSF5, ZC3H4
LMP1+Inhs vs LMP2+Inhs	ABT1, ACLY, ACTR1A, ASMTL, ATG2A, ATP6V0D1, ATXN7L3, C9orf78, CAPZB, CCDC171, CCDC47, CHD1, CLIC2, CLIC3, CLPTM1L, COPS6, CSDA, CSNK1D, CTAGE5, DCAF4L2, DHX30, DOCK9, DUS3L, ECSIT, EFR3A, EFTUD2, EIF3B, EIF3E, EIF4A1, EIF4H, ENO1, EPPK1, EXOC3, FBXO7, FHL1, FHOD1, FSCN1, FUBP3, FXR1, G3BP1, G3BP2, GBAS, GBE1, GINS4, GRSF1, GTF2I, HDDC2, HDGFRP3, HEBP1, HELZ2, IL18, IPO9, ITGA5, KIF16B, LAMA5, LARP4, MAP2K2, MEAF6, MTERFD1, MYL6, NOLC1, NUDT19, P4HA2, PACSIN2, PGRMC1, PHGDH, PKD1, PPP1CC, PRMT7, PSMB1, PSMC2, PSMG1, RAB7A, RAP1GDS1, RBBP7, RMND1, RNF214, RPL8, RPL9, RPS6KC1, RRP7A, SCFD2, Selm, SERPINB11, SMARCAD1, SNRNP200, SNX24, SNX3, SPCS3, strap, TCEB2, THOC5, TLR3, TMEM63B, TMEM87A, TOMM5, TOMM7, TOR2A, UAP1, UBE3B, UBE4A, UFL1, USP38, UTP11L, VIM, WDR6, YIPF2, YIPF4, ZMYM2, ZNHIT2

**FIG 3:**
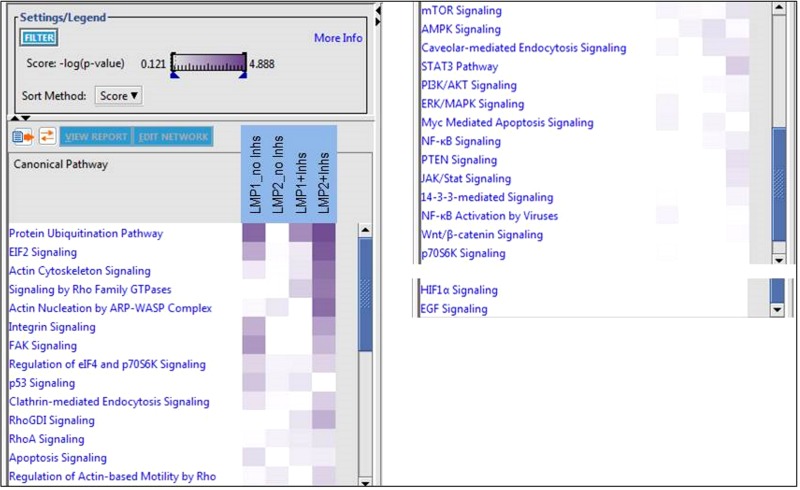
Analysis comparison of canonical pathways. A comparison of the canonical pathways associated with significantly different protein expression for each genotype, with or without inhibitors, was performed using IPA. The comparison was further sorted according to the *P* value of association (−log_10_) with a particular pathway and is represented by the intensity of each node in the heat map. A greater number of pathways were significantly associated with the addition of inhibitors, reflecting the increased number of significant changes in expression associated with these samples. In addition, the most significant pathway associated across almost all samples was the Protein ubiquitination pathway.

Identification of canonical pathways (Fig. 3) revealed major effects, with highly significant *P* values on the protein ubiquitination pathway in LMP1 with and without inhibitors and in LMP2A with inhibitors compared to the pBabe controls. Canonical pathways involved in EIF2 signaling, regulation of eIF4 and p70s6K signaling, actin cytoskeleton signaling, regulation of actin-based motility by Rho, integrin, FAK, PI3K/Akt, ERK/MAPK, and p53 signaling were all differentially regulated. Although not all of these pathways had a significant number of proteins associated with them to indicate activation or deactivation, the results did indicate that there were a number of significantly different proteins for these associated functions. Multiple pathways involved in vesicle formation and endocytosis, including Clathrin- and Caveolar-mediated endocytosis, were also differentially regulated (Fig. 3).

**FIG 4:**
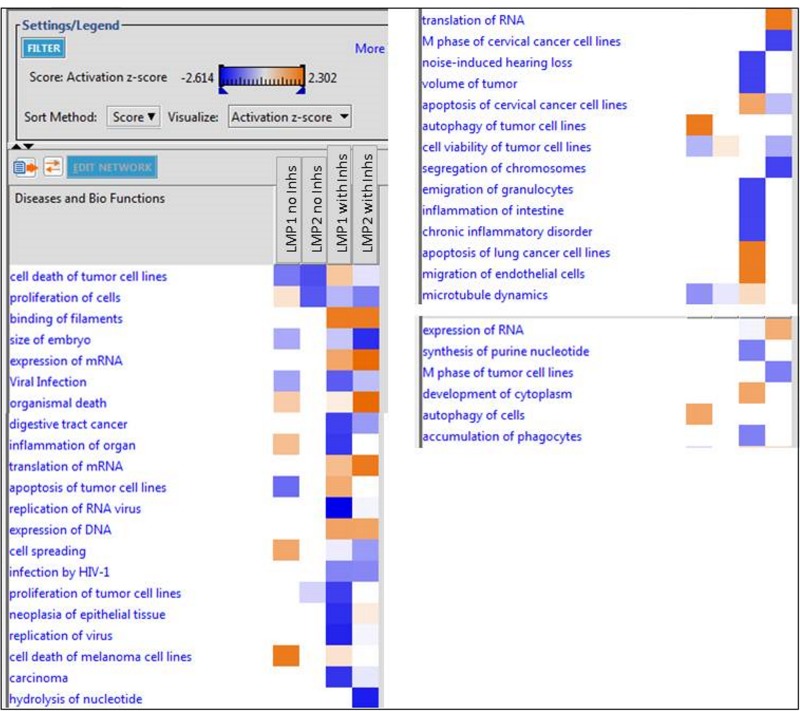
Analysis of the Diseases and bio-functions group. A comparison of the diseases and biological functions associated with significant expression changes for LMP1 and LMP2A, with or without inhibitors, versus the pBabe control, is shown. The scores are displayed on a scale from dark blue to orange, with dark blue representing the most negative scores or inactivation and orange representing the highest positive scores or activation. Only diseases and functions with a *P* value of <0.05 are displayed. Activation scores with an absolute value of 2 or more are considered significant. Again, an increase in the number of significant scores was found in samples where inhibitors were added.

Analysis of diseases and biofunction identified many of the functions previously described for LMP1 based on significant *Z* scores (Fig. 4). A *Z* score greater than 2 strongly suggests activation or inhibition. Thus, cell death of tumor cells was considerably impaired in the LMP1 and LMP2A cells in the absence of inhibitors, while cell proliferation was activated in the LMP1 cells in the absence of inhibitors. Networks for cell death or proliferation and changes in migration of endothelial cells were significantly altered in LMP1 cells compared to the pBabe control. LMP1 without inhibitors also significantly decreased apoptosis but increased autophagy.

The significant differences in diseases and biofunctions induced by the expression of LMP2A were also assessed using IPA. Many of the functions previously shown to be affected by LMP2A were identified in this analysis to be differentially regulated in the presence of inhibitors compared to the pBabe control. Binding of filaments was activated by LMP2A with inhibitors, while cell spreading was decreased (Fig. 4). In addition, potential new cellular functions predicted to be affected by LMP2A included RNA, DNA, and protein expression (expression of mRNA, translation of mRNA, expression of DNA, translation of mRNA, translation of RNA, and expression of RNA) (Fig. 4).

Overall, many of the pathways identified based on canonical pathways analysis have been previously described to be affected by LMP1 and LMP2A, including cell motility, cell junction signaling, actin cytoskeleton regulation, RhoA, PI3K/AKT, PTEN signaling, mTOR signaling, and p53 signaling, although not all of these had significant *P* values in each comparison. These data provided identification of novel protein targets that contribute to the effects mediated by LMP1 and LMP2A. Additionally, these data identified new effects on EIF2 signaling and regulation of eIF4 and p70s6K signaling. Analysis of canonical pathways identified new potential effects on mRNA processing, DNA methylation and transcriptional repression, cleavage and polyadenylation of pre-mRNA, and tRNA charging, which were all differentially regulated.

In order to simplify the description of results, the four comparison groups are referred to as LMP1-noInh (LMP1 versus pBabe without inhibitors), LMP2A-noInh (LMP2A versus pBabe without inhibitors), LMP1+Inh (LMP1 versus pBabe with inhibitors), and LMP2A+Inh (LMP2A versus pBabe with inhibitors), with specific attention on the protein ubiquitination pathway, vesicle formation and trafficking, transcription and translation, cytoskeleton cell movement and cell junctions, and pathways previously described for LMP1 and LMP2A (Tables 3 and 4; Tables S2, S3, S4, and S5).

10.1128/mBio.00959-18.2 TABLE S2  IPA data: vesicle formation and trafficking. The *P* value and fold change data for all proteins associated with vesicle formation and trafficking pathways in the IPA analysis are shown. In each pathway, the significant data for each comparison (LMP1-noInhs, LMP2A-noInhs, LMP1+Inhs, LMP2A+Inhs) are presented, with significant *P* values highlighted following the convention used for IPA. Green cells indicate significantly downregulated levels versus pBabe, and red cells indicate significantly upregulated levels versus pBabe. For each protein (or gene) that was significant in one comparison, data for other comparisons are also presented, unless they were from the manual curation. Download TABLE S2, PDF file, 0.11 MB. Copyright © 2018 DeKroon et al. 2018 DeKroon et al. This content is distributed under the terms of the Creative Commons Attribution 4.0 International license.

10.1128/mBio.00959-18.3 TABLE S3  IPA data: transcription and translation. The *P* value and fold change data for all proteins associated with transcription and translation pathways in the IPA analysis are shown. In each pathway, the significant data for each comparison (LMP1-noInhs, LMP2A-noInhs, LMP1+Inhs, LMP2A+Inhs) are presented, with significant *P* values highlighted following the convention used by IPA. Green cells indicate significantly downregulated levels versus pBabe, and red cells indicate significantly upregulated levels versus pBabe. For each protein (or gene) that was significant in one comparison, the data from other comparisons are also presented. Download TABLE S3, PDF file, 0.24 MB. Copyright © 2018 DeKroon et al. 2018 DeKroon et al. This content is distributed under the terms of the Creative Commons Attribution 4.0 International license.

10.1128/mBio.00959-18.4 TABLE S4  IPA data: cytoskeleton, cell movement, and cell junctions. The *P* value and fold change data for all proteins associated with cytoskeleton, cell movement, or cell junction pathways in the IPA analysis are shown. In each pathway, the significant data for each comparison (LMP1-noInhs, LMP2A-noInhs, LMP1+Inhs, LMP2A+Inhs) are presented, with significant *P* values highlighted following the convention used by IPA. Green cells indicate significantly downregulated levels versus pBabe, and red cells indicate significantly upregulated levels versus pBabe. For each protein (or gene) that was significant in one comparison, the data in other comparisons are also presented. Download TABLE S4, PDF file, 0.16 MB. Copyright © 2018 DeKroon et al. 2018 DeKroon et al. This content is distributed under the terms of the Creative Commons Attribution 4.0 International license.

10.1128/mBio.00959-18.5 TABLE S5  IPA data: other or known LMP1-LMP2A pathways. The *P* value and fold change data for all proteins associated with known LMP1- or LMP2A-associated pathways in the IPA analysis are shown. In each pathway, the significant data for each comparison (LMP1-noInhs, LMP2A-noInhs, LMP1+Inhs, LMP2A+Inhs) are presented, with significant *P* values highlighted following the convention used by IPA. Green cells indicate significantly downregulated levels versus pBabe, and red cells indicate significantly upregulated levels versus pBabe. For each protein (or gene) significant in one comparison, the data in other comparisons are also presented. Download TABLE S5, PDF file, 0.25 MB. Copyright © 2018 DeKroon et al. 2018 DeKroon et al. This content is distributed under the terms of the Creative Commons Attribution 4.0 International license.

**TABLE 3:** Proteins in the ubiquitination pathway identified via IPA

Comparison and protein	Entrez gene name	*P* value	Fold change
LMP1 vs pBabe: no inhibitors (*P* = 1.92E−05)			
DNAJC7	DnaJ (Hsp40) homologue, subfamily C, member 7	3.90E−02	−1.852
HSPA14	Heat shock 70-kDa protein 14	1.98E−02	−1.667
HSPB1	Heat shock 27-kDa protein 1	1.20E−02	3.427
PSMD2	Proteasome (prosome, macropain) 26S subunit, non-ATPase, 2	5.38E−03	1.171
PSMD8	Proteasome (prosome, macropain) 26S subunit, non-ATPase, 8	1.92E−02	−1.728
THOP1	Thimet oligopeptidase 1	3.73E−02	−1.982
UBE2V1	Ubiquitin-conjugating enzyme E2 variant 1	1.10E−03	1.739
USP5	Ubiquitin-specific peptidase 5	2.61E−02	−1.490
USP14	Ubiquitin-specific peptidase 14	2.57E−02	−2.353
LMP2 vs pBabe: no inhibitors (*P* = 1)			
None			
LMP1 vs pBabe: with inhibitors (*P* = 2.35E−06)			
BAG1	BCL2-associated athanogene	2.96E−03	2.613
BIRC3	Baculoviral IAP repeat-containing 3	1.58E−02	−1.760
CUL1	Cullin-1	3.57E−03	−1.414
DNAJC5	DnaJ (Hsp40) homologue, subfamily C, member 5	2.41E−02	−2.692
HLA-B	Major histocompatibility complex, class I, B	2.06E−02	4.993
HSP90AA1	Heat shock protein 90-kDa alpha (cytosolic), class A member 1	3.43E−02	−1.335
HSPA6	Heat shock 70-kDa protein 6 (HSP70B′)	5.54E−03	−1.492
HSPA8	Heat shock 70-kDa protein 8	4.62E−02	−2.934
HSPA1A	Heat shock 70-kDa protein 1A	2.34E−03	−1.242
PSMB1	Proteasome (prosome, macropain) subunit, beta type, 1	1.08E−03	2.223
PSMB8	Proteasome (prosome, macropain) subunit, beta type, 8	9.89E−03	2.207
PSMB10	Proteasome (prosome, macropain) subunit, beta type, 10	1.03E−02	2.319
PSMC2	Proteasome (prosome, macropain) 26S subunit, ATPase, 2	1.89E−02	1.127
PSMD14	Proteasome (prosome, macropain) 26S subunit, non-ATPase, 14	4.95E−02	−1.390
RBX1	Ring box 1, E3 ubiquitin protein ligase	2.30E−02	−1.494
TCEB1	Transcription elongation factor B (SIII), polypeptide 1	4.49E−02	−1.414
TCEB2	Transcription elongation factor B (SIII), polypeptide 2	1.33E−03	−1.690
UBE2I	Ubiquitin-conjugating enzyme E2I	1.19E−02	−1.485
UBE2L3	Ubiquitin-conjugating enzyme E2L 3	1.42E−02	4.031
UBE3B	Ubiquitin protein ligase E3B	6.61E−03	1.659
UBE4A	Ubiquitination factor E4A	1.94E−02	−2.941
USP38	Ubiquitin-specific peptidase 38	3.60E−02	−3.717
LMP2 vs pBabe: with inhibitors (*P* = 4.26E−05)			
CDC34	Cell division cycle 34	3.99E−02	−2.274
DNAJA1	DnaJ (Hsp40) homologue, subfamily A, member 1	5.00E−02	−1.763
HSCB	HscB mitochondrial iron-sulfur cluster cochaperone	4.67E−02	−2.333
PSMB1	Proteasome (prosome, macropain) subunit, beta type, 1	1.73E−02	1.456
PSMC2	Proteasome (prosome, macropain) 26S subunit, ATPase, 2	2.62E−03	1.287
PSMD2	Proteasome (prosome, macropain) 26S subunit, non-ATPase, 2	2.70E−02	−1.227
TAP2	Transporter 2, ATP-binding cassette, subfamily B	3.16E−02	−2.098
TCEB2	Transcription elongation factor B (SIII), polypeptide 2	1.27E−02	−1.275
TRAF6	TNF receptor-associated factor 6, E3 ubiquitin protein ligase	5.71E−03	−1.943
UBA1	Ubiquitin-like modifier activating enzyme 1	2.85E−03	−1.117
UBE2C	Ubiquitin-conjugating enzyme E2C	1.21E−02	−5.242
UBE2L6	Ubiquitin-conjugating enzyme E2L 6	2.87E−02	−2.430
UBE3B	Ubiquitin protein ligase E3B	4.58E−02	1.134
UBE4A	Ubiquitination factor E4A	9.86E−03	−7.551
USP38	Ubiquitin-specific peptidase 38	2.81E−02	−5.336
USP48	Ubiquitin-specific peptidase 48	8.21E−03	−1.919

**TABLE 4:** Ubiquitination pathway proteins based on manual curation

Comparison and protein	Entrez gene name	*P* value	Fold change
LMP1 vs pBabe: no inhibitors			
VBP1	Von Hippel-Lindau binding protein 1	1.50E−02	−4.055781694
CCDC50 (Ymer)	Coiled-coil domain containing 50	4.08E−02	2.574692252
BAG2	BCL2-associated athanogene 2	1.56E−02	−1.975596065
THOC1 (HPR1)	THO complex 1	7.01E−03	−2.12573241
LMP2 versus pBabe: no inhibitors			
RPS25	Ribosomal protein S25	3.99E−02	1.423354477
SPG20 (Spartin)	Spastic paraplegia 20 (Troyer syndrome)	1.20E−03	2.185767576
LMP1 versus pBabe: with inhibitors			
DCAF4L2	DDB1- and CUL4-associated factor 4-like protein 2	3.00E−03	1.997755058
FBXL18	Isoform 4 of F-box/LRR-repeat protein 18	1.08E−02	2.490401919
FBXO7	F-box only protein 7	4.35E−02	1.868799777
PSMG1	Isoform 2 of proteasome assembly chaperone 1	2.51E−02	−1.933470638
SH3RF2	Putative E3 ubiquitin-protein ligase SH3RF2	1.22E−02	1.739935853
TRIM27	Isoform beta of zinc finger protein RFP	2.37E−02	1.677203226
UBAP1	Isoform 2 of ubiquitin-associated protein 1	5.99E−03	1.662662369
UBE2K	Ubiquitin-conjugating enzyme E2 K	1.86E−03	−1.489748169
UBR7	Putative E3 ubiquitin-protein ligase UBR7	4.61E−02	0.364044384
UFL1	E3 UFM1-protein ligase 1	3.58E−02	−1.27422905
UFM1	Ubiquitin-fold modifier 1	4.72E−02	1.622521302
COPS6	COP9 signalosome complex subunit 6	5.68E−03	−1.935473811
LMP2 vs pBabe: with inhibitors			
ATXN3L	Putative ataxin-3-like protein	2.32E−02	23.37327866
ATXN7L3	Ataxin-7-like protein 3	4.19E−02	1.367686338
CBX4	E3 SUMO-protein ligase CBX4	4.31E−02	1.424713914
CCM2	Isoform 2 of malcavernin	1.69E−02	−2.262188449
COPS6	COP9 signalosome complex subunit 6	3.01E−05	−2.303932902
FBXO7	F-box only protein 7	1.37E−02	1.404454865
PIAS3	E3 SUMO-protein ligase PIAS3	1.35E−02	6.19446027
POMP	Proteasome maturation protein	4.47E−02	−2.469462113
PSMG1	Isoform 2 of proteasome assembly chaperone 1	6.54E−03	−1.961205514
RNF114	RING finger protein 114	4.65E−02	−1.466037981
RNF138	E3 ubiquitin-protein ligase RNF138	2.37E−03	1.371846044
SDCBP (MDA-9)	Isoform 3 of syntenin-1	2.26E−03	−1.419647959
UFL1	E3 UFM1-protein ligase 1	2.88E−02	−1.316652316

### Protein ubiquitination pathway.

One of the top canonical pathways found by IPA was the protein ubiquitination pathway (Table 3). For LMP1-noInh, IPA found a significant number of proteins associated with the pathway (*P* = 1.92 × 10^−5^) (Table 3), some of which included DNAJC7 (downregulated 1.85-fold), the proteasome subunits PSMD2 (upregulated 1.17-fold) and PSMD8 (downregulated 1.73-fold), ubiquitin-specific peptidases USP5 and USP14 (downregulated 1.49- and 2.35-fold, respectively), and the E2 conjugase UBE2V1 (upregulated 1.74-fold). In contrast, for LMP2A-noInh, no proteins associated with the protein ubiquitination pathway were identified by IPA. However, further examination of the IPA data revealed a number of proteins with significantly different expression than their pBabe control that were not included by IPA but could be added to the protein ubiquitination pathway based on functions described in the literature. This manual curation (Table 4) identified RPS25 (upregulated 1.42-fold), which has been demonstrated to bind MDM2 and inhibit its E3 ubiquitin ligase activity, leading to a reduction of its p53 ubiquitylation and resulting in p53 stabilization (Table 4) (30). In addition, Spartin (SPG20) was significantly upregulated in LMP2A-expressing cells compared to the pBabe control (upregulated 2.19-fold). Spartin binds and activates ITCH, a major E3 ubiquitin ligase that binds to LMP2A and is responsible for LMP2A-induced turnover of the B-cell receptor (27, 31, 32).

IPA also found a significant number of proteins from the LMP1+Inh group that are associated with the protein ubiquitination pathway (*P* = 2.35 × 10^−6^) (Table 3). Some of these included the downregulation of a number of heat shock proteins, the E3 ubiquitin ligase component Cullin-1 (downregulated 1.414-fold), the proteasome subunits PSMB1 (upregulated 2.223-fold), PSMB8 (upregulated 2.207-fold), PSMB10 (upregulated 2.319-fold), PSMC2 (upregulated 1.127-fold), and PSMD14 (downregulated 1.390-fold), the E2 ubiquitin conjugases UBE2I (downregulated 1.485-fold) and UBE2L3 (upregulated 4.031-fold), the E3 ubiquitin ligase UBE3B (upregulated 1.659-fold), the ubiquitination factor UBE4A (downregulated 2.941-fold), the ubiquitin-specific peptidase USP38 (downregulated 3.717-fold), and the E3 ubiquitin ligase RBX1 (downregulated 1.494-fold). Manual curation (Table 4) also added two F-box proteins, FBXO7 (upregulated 1.869-fold) and FBXL18 (upregulated 2.490-fold), both substrate-recruiting subunits of Skp1-cullin1-FBP (SCF)-type E3 ubiquitin ligases (33), PSMG1 (downregulated 1.933-fold), E2 ubiquitin conjugase UBE2K (downregulated 1.490-fold), which is known to interact with RING finger proteins to modulate the ubiquitin linkage (34), and UFM1 (upregulated 1.623-fold), a ubiquitin-like protein.

For LMP2A+Inh, IPA revealed a significant number of proteins with functions associated with the protein ubiquitination pathway (*P* = 4.26 × 10^−5^) (Table 3). This group of proteins contained the E3 ubiquitin ligases TRAF6 (downregulated 1.943-fold) and UBE3B (upregulated 1.134-fold), the ubiquitin E2 conjugase enzymes UBE2C (downregulated 5.242-fold), UBE2L6 (downregulated 2.430-fold), and CDC34 (UBE2R1) (downregulated 2.274-fold), the proteasome subunits PSMB1 (upregulated 1.456-fold), PSMC2 (upregulated 1.287-fold), and PSMD2 (downregulated 1.227-fold), and the ubiquitin-specific peptidases USP38 (downregulated 5.336-fold) and USP48 (downregulated 1.919-fold). Manual curation (Table 4) added the E3-SUMO ligases CBX4 (upregulated 1.425-fold), which SUMOylates HNRNPK (35), and PIAS3 (upregulated 6.194-fold), which binds and stabilizes p53, inhibiting its ubiquitination by MDM2 and thereby regulating cell growth. In addition, manual curation also added FBOX7 (upregulated 1.404-fold), two proteasome component chaperones, POMP (downregulated 2.469-fold) and PSMG1 (downregulated 1.961-fold), and two RING finger proteins, RNF114 (downregulated 1.466-fold) and RNF138 (upregulated 1.372-fold), both of which act as E3 ubiquitin ligases. RNF114 interacts with A20 and modulates NF-κB activity (36). LMP2A has been shown to affect NF-κB activation by LMP1 through effects on TRAF2 (37). These proteomic study results suggest that effects on TRAF6 and RNF114 may be additional mechanisms through which LMP2A might affect the activity of LMP1.

### Vesicle formation and trafficking.

IPA identified multiple proteins with functions in vesicle formation and trafficking pathways, including exocytosis, endocytosis, and autophagy. For LMP1-noInh, IPA identified a significant number of proteins in the “Autophagy of tumor cell lines” (*P* = 0.0126; *Z* score,1.987) and “Autophagy of cells” (*P* = 0.0279; *z* score, 1.334) categories in the Disease and Biofunctions group (Fig. 4; Table S2). Some of these proteins included Rab1A, calpain-1 (CAPN1), and EIF2AK2 (PKR). Manual curation added NDRG1 (N-myc downstream regulated 1 protein; upregulated 2.06-fold; *P* = 0.00019), which is a metastasis suppressor that inhibits stress-induced autophagy in cancer cells (38), CLIC4 (upregulated 1.73-fold; *P* = 0.048) which when downregulated enhances autophagy and endoplasmic reticulum (ER) stress-induced apoptosis (39), and VBP1 (von Hippel-Lindau-binding protein; downregulated 4.06-fold; *P* = 0.015), which mediates autophagy-dependent protein degradation of hMSH4, a MutS protein involved in regulating DNA double-strand break repair in homologous recombination (40) (Table S2).

Other proteins with known functions in vesical formation and trafficking, for example, Clathrin (CLTA), ARPC4, and Flottilin-2 (FLOT2), had significantly different expression in LMP1-expressing cells than in the pBabe control. However, the IPA pathways associated with these proteins did not contain a significant number of proteins (e.g., those from the groups for Viral entry via endocytic pathway, Clathrin-mediated endocytosis signaling, Caveolar-mediated endocytosis signaling, and Fc-gamma receptor phagocytosis). In addition, manual curation found a number of other proteins with functions in vesicle formation control (Table S2), including ATP6V1C1, a vacuolar ATPase subunit important for the acidification of intracellular components (41), SEC23B, a subunit of the COPII complex required for EGFR transport from the ER to the plasma membrane (42), EHD4, an Eps homology domain protein involved in the control of early endosome trafficking (43), and UBE2V1, an E2 Ub-conjugase that binds TRAF6 and ESCRT-0, a complex important for sorting of ubiquitinated cargo (44).

With LMP2A-noInh, IPA found a significant association with the Fusion of autophagosomes (*P* = 0.0154) and Fusion of late endosomes (*P* = 0.0176) categories, both of which only contained Rab7a from our data set, which was downregulated 1.678-fold (*P* = 0.04) (Table S2). Rab7A is a Rab family member of small RAS-related GTP-binding proteins that is important for regulating vesicle traffic from the late endosome to lysosomes and autolysosome formation (45). Rab7a was also associated with the Clathrin-mediated endocytosis signaling group (Table S2). Other differentially expressed proteins associated with IPA vesicle formation and trafficking pathways were VAMP3 (Fc-gamma receptor phagocytosis), SNX5 (macropinocytosis), and CHMP4B/VPS32 (Viral exit from cells) (Table S2). Manual curation also added a number of proteins to this functional category: MYOF and EHD4 (early endosome transport), which were downregulated, and RIN1 (ras effector protein capable of activating Rab5), NDRG1 (an inhibitor of stress induced autophagy), and SPG20 (Spartin), which were upregulated (Table S2) (46, 47). Spartin, which as described above activates Itch, also binds the ESCRT-III protein complex, which regulates vesicle trafficking and cargo sorting, primarily at the last stage of vesicle formation or budding. It has been shown that Spartin upregulation results in decreased EGFR degradation and decreased internalization, properties that would complement the EGFR induction by LMP1 (48).

IPA analysis of LMP1+Inh found 51 proteins with significant associations with 5 vesicle formation and trafficking pathways, including the groups Autophagy (*P* = 0.00219), Endocytosis (*P* = 2.29 × 10^−4^), Macropinocytosis (*P* = 0.01), Viral entry via endocytic pathway (*P* = 0.0437), and Caveolar-mediated endocytosis signaling (*P* = 0.0497) (Table S2). The Autophagy pathway contained 21 proteins in total, with 10 upregulated and 11 downregulated. Upregulated proteins included MAP1LC3B (LC3), RB1CC1, and ULK3, while downregulated proteins included Rab7a, eIF2S1, ATG2A, TBC1D4, and TBK1. ATG2A is an autophagy-related protein thought to regulate formation and dispersion from autophagosomes (49). Interestingly, TBK1 (TANK-binding kinase) is important in innate immunity and autophagy. Its activation is critically regulated by its cellular localization. The detection of this protein in the presence of inhibitors suggests that LMP1 may affect TBK localization and activation through effects on ubiquitination (50). Manual curation added ELMO2 (engulfment and cell motility protein 2) (upregulated 1.27-fold; *P* = 0.039), which is essential for phagocytosis and migration (47), TMEM59 (upregulated 1.53-fold; *P* = 0.037), which contains a novel ATG16L1-binding motif and promotes local activation of LC3 (also upregulated more than 4-fold). It has been demonstrated to bind ATG16L1 and mediate autophagy during *Staphylococcus aureus* infection (51). In addition, Rab8B, which is important for autophagosome maturation, was upregulated 1.9-fold (*P* = 0.034) (52) and WDR6 was downregulated 1.78-fold (*P* = 0.028). WDR6 is a negative regulator of amino acid starvation-induced autophagy (53).

Similarly, the Endocytosis pathway included 24 proteins in total, with 11 proteins upregulated and 13 downregulated. Upregulated proteins included ATP6V0D1, CLIC3, Ezrin (EZR), and RAC2, while downregulated proteins included Rab7a, SNX3, ATG2A, and AP1S1. Ezrin acts as a cross-linker between membranes and the cytoskeleton and is important for endocytosis, phagocytosis, and vesicle trafficking, enhancing cell motility and invasiveness. It is known that Ezrin phosphorylation is induced by LMP1-enhanced cell motility (54). Manual curation added another 18 proteins with Rab11FIP1, Rab GTPase-binding effector protein 2 (RabEP2), ATP6V1B1, TBC1D13, sorting nexin-24 (SNX24), and synaptogyrin-2 (SYNGR2) being upregulated. Rab11FIP1 is a Rab-interacting protein that is critical for trafficking into early endosomes (55). TBC1D13, a Rab35-specific GAP that contributes to GLUT4 trafficking, was also upregulated (56), while CCT8, NISCH, and VAMP5 were downregulated (Table S2).

For LMP2A+Inh, IPA revealed 32 proteins with significant associations with 3 vesicle formation and trafficking pathways (Table S2), Endocytosis (*P* = 2.63 × 10^−4^), Fusion of late endosomes (*P* = 0.0095), and Endocytosis by tumor cell lines (*P* = 0.013). The Endocytosis group contained Annexin-6 (ANXA6), which has been suggested to mediate the aggregation and fusion of endosomes during exocytosis in epithelial layers (57), as well as ATG2A, ATP6V1F, ATP6V1H, Rab7a, and SNX3, all of which were downregulated. Only ATP6V0D1 was upregulated. The Fusion of late endosomes group contained STX8, a syntaxin protein implicated in protein trafficking from early to late endosomes (58), Rab7a, and C20orf24 (RIP5), a Rab5 interaction protein (Table S2) (59). Other proteins associated via IPA with vesicle formation and trafficking pathways included MAP2K2, integrin α5, CHMP3 (VPS24), CD2AP, and VPS4A, which were significantly differentially expressed compared to the pBabe control (Table S2). CHMP3 is part of the ESCRT-III complex that recruits transmembrane proteins to lysosomes via the MVB, while VSP4 catalyzes ESCRT-III disassembly, reallocating the components to the cytoplasm for MVB sorting and contributing to herpesvirus envelopment (60). Manual curation added a number of significantly different proteins compared to the pBabe control with functions in vesicle formation and trafficking. Some of these included LAMTOR4, Rab1B, Rab38, EXOC3 (sec6), EXOC5, EXOC6B (sec15), and TMED4. LAMTOR4 recruits mTORC1 to lysosomes, leading to its activation, while Rab38 mediates protein trafficking to lysosomes (61, 62). EXOC3, EXOC5, and EXOC6B are components of the exocyst complex that targets exocytic vesicles to specific sites on the plasma membrane (63).

### Transcription and translation.

IPA also revealed a number of overlapping pathways containing significant numbers of proteins with functions in transcription and translation (Table S3). For LMP1-noInh, IPA resulted in significant Fisher exact test scores in the pathways for EIF2 signaling (*P* = 4.65 × 10^−3^), tRNA charging (*P* = 1.18 × 10^−2^), translation of protein (*P* = 2.52 × 10^−4^), translation of mRNA (*P* = 7.45 × 10^−5^), expression of protein (*P* = 4.69 × 10^−4^), and processing of RNA (*P* = 9.51 × 10^−3^). In EIF2 signaling EIF2AK2, EIF3L, EIF4A1, and RPL12 were downregulated while EIF2B4 was upregulated. EIF2AK2, EIF2B4, and EIF3L were also included in the Translation of mRNA, Translation of protein, and Expression of protein pathways. All other proteins associated with these pathways are listed in Table S3. In contrast, for LMP2A-noInh, the only pathway IPA found with a significant number of proteins functioning in transcription and translation was the Cleavage and polyadenylation of pre-RNA pathway (*P* = 3 × 10^−4^). IPA found other pathways with LMP2A-noInh, but each of these had only one protein listed, ribosomal protein S25 (RPS25) in EIF2 signaling and eIF4-p70S6K signaling and glycyl-tRNA-synthetase (GARS) in tRNA charging.

With the addition of inhibitors, a greater number of transcription and translation pathways had significant scores with IPA. In addition, there were a greater number of differentially expressed proteins associated with these pathways, most of which were downregulated (Table S3). For instance, in LMP1+Inh, 13 out of 15 proteins associated with the EIF2 signaling pathway were downregulated. Among the proteins downregulated in LMP1+Inh were EIF2, -3, and -4 subunits and ribosomal proteins L8, L9, L31, and L32. In LMP2A+Inh, 10 out of 14 proteins were downregulated in the EIF2 signaling pathway, including EIF3, -4, and -5 subunits, as well as several ribosomal proteins. These proteins were also included in the eIF4 and p70s6K signaling, expression of mRNA, translation of mRNA, translation of RNA, expression of protein, and translation of protein pathways (Table S3).

### Cytoskeleton, cell movement, and cell junctions.

With the addition of inhibitors, the number of cytoskeleton, cell movement, and cell junction pathways containing significant numbers of proteins identified via IPA increased dramatically in LMP1-expressing cells (Table S4). Without inhibitors, LMP1-expressing cells had only one significant pathway, Integrin signaling (*P* = 6.27 × 10^−3^), in which ARPC4, CAPN1, and RAP1B were downregulated while CAPNS1 and vinculin (VCL) were upregulated. Other pathways had proteins listed for LMP1-noInhs but their *P* values were not significant. In addition, for LMP2A-noInh there were no pathways with statistically significant numbers of proteins in these functional categories. With the addition of inhibitors to LMP2A-expressing cells, only the Rac signaling (*P* = 1.32 × 10^−2^) and Binding of filaments (*P* = 4.51 × 10^−3^) pathways contained significant numbers of proteins. In Rac signaling, six proteins were downregulated (ACTR2, BRK1, ITGA5, MAP2K2, NCKAP1, and RPS6KB1) and one was upregulated (NF-κB1), while in Binding of filaments all four proteins were downregulated (CSNK1D, FAS, FHL1, and VIM). It is intriguing that LMP2A would affect the expression of NF-κB1, a major component of the canonical NF-κB pathway that is activated by LMP1. This provides another mechanism for synergy between LMP1 and LMP2A.

In contrast, with LMP1+Inh cells the number of pathways IPA found with statistically significant numbers of proteins increased to 13 (Table S4). These pathways included Integrin signaling (*P* = 3.67 × 10^−5^), Actin cytoskeleton signaling (*P* = 9.58 × 10^−6^), Epithelial adherens junction signaling (*P* = 4.98 × 10^−5^), Sertoli cell-Sertoli cell junction signaling (*P* = 3.15 × 10^−6^), Tight junction signaling (*P* = 6.25 × 10^−4^), Rac signaling (*P* = 2.6 × 10^−2^), RhoA signaling (*P* = 6.52 × 10^−3^), Leucocyte extravasation signaling (*P* = 4.52 × 10^−4^), and ILK signaling (*P* = 5.18 × 10^−3^).

### Pathways previously described for LMP1 and LMP2A (or other pathways).

A number of functional pathways previously described for LMP1 and LMP2A were also implicated by IPA (Table S5). In LMP1-noInh, the p53 signaling pathway contained a statistically significant number of proteins (*P* = 1.9 × 10^−2^), although only 3 proteins were listed. These were SERPINB5 and Stratifin (SFN), which were upregulated, and PCNA, which was downregulated. For LMP1+Inh, p53 signaling contained PERP (TP53 apoptosis effector) was downregulated 7.4-fold and TP53IP3 was upregulated 3.2-fold. LMP1+Inh also contained a significant number of proteins in the CDC42 signaling pathway (*P* = 1.67 × 10^−2^), with 8 of 10 proteins being upregulated. Some of these included IQGAP3, HLA-B, FDG1, and ARPC1A.

For LMP2A+Inh, the mTOR signaling (*P* = 1.33 × 10^−3^), PI3K/AKT signaling (*P* = 3.26 × 10^−3^), and PTEN signaling (*P* = 8.34 × 10^−3^) pathways contained significant numbers of proteins. In the mTOR signaling pathway, 9 of 11 proteins were downregulated and this included a number of EIF3 and EIF4 proteins. In the PI3K/AKT signaling pathway, 6 of 8 proteins were downregulated and also included INPP5K, MAP2K2, and PPP2R2A. For the PTEN signaling pathway, in which 5 of 7 proteins were downregulated, INPP5K, MAP2K2, and RPS6KB1 were also included (Table S5).

Several members of the NF-κB signaling pathway were identified, including NF-κB1 (*P* = 0.0162), and manual curation found NF-κB-activating protein (NKAP) (*P* = 0.0379) was significantly upregulated in LMP2A+Inh compared to its pBabe control. In addition, IKBKG was upregulated 1.742-fold in LMP1 with inhibitors (*P* = 5.03 × 10^−3^), while TANK-binding kinase (TBK1) was downregulated 1.305-fold (*P* = 0.0425) (Table S5). These data suggest that in the presence of LMP1, IKBKG had increased ubiquitination, which is in keeping with the activation of NF-κB by LMP1 mediated through effects on IKBKG.

### Confirmation of protein expression changes associated with vesicle formation and trafficking.

**FIG 5:**
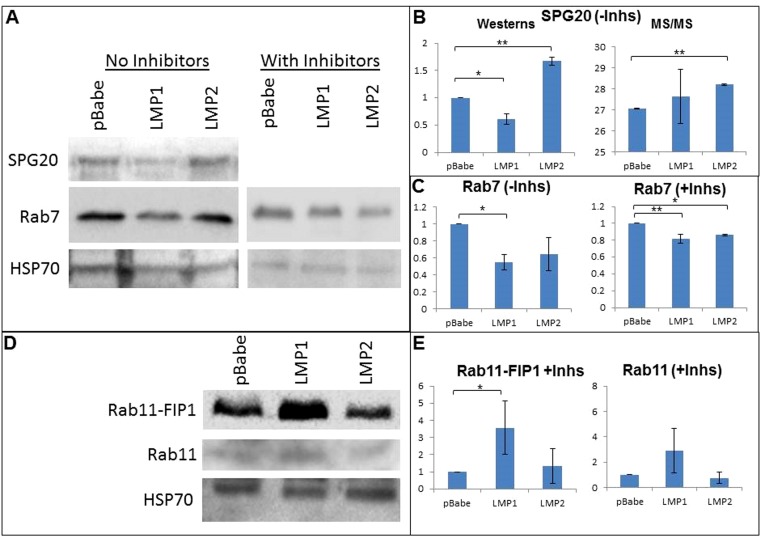
Western transfers of SPG20, Rab7, Rab11FIP1, and Rab11. Protein expression changes were assessed for SPG20 and Rab7 in pBabe, LMP1 and LMP2A samples without inhibitors, and Rab7, Rab11FIP1, and Rab11 in pBabe, LMP1, and LMP2A samples with inhibitors. Western blots for SPG20 (A and B) confirmed its increased expression in LMP2A-noInh cells (*P* < 0.01). With LMP1-noInh cells, SPG20 was significantly reduced (*P* < 0.05), in contrast to the MS/MS data, which contained greater variance. Rab7 expression (A and C) without inhibitors was downregulated in both LMP1 and LMP2A samples, although this change was only significant with LMP1 expression (*P* < 0.05). With the addition of inhibitors, Rab7 levels in LMP1 and LMP2A cells were both significantly decreased compared to their pBabe controls (*P* = 0.05 and 0.01, respectively). Rab11FIP1 and Rab11 (D and E) were increased based on Western blot analysis in LMP1+Inh, but only Rab11-FIP1 in LMP1+Inh was significantly different (*P* < 0.05).

Several of the vesicle trafficking proteins altered with the expression of LMP1 or LMP2A were confirmed by Western transfer (Fig. 5). SPG20 (Spartin) expression was confirmed to be increased in LMP2A-noInh cells compared to the pBabe-noInh control, in agreement with the MS data (Fig. 5A and B). However, Spartin was significantly reduced in the LMP1-noInh cells compared to the pBabe-noInh control (*P* = 0.03), in contrast to the MS/MS data which indicated greater variance and were not significantly different (Fig. 5B). Western transfer for Rab7 expression without inhibitors was down in both LMP1 and LMP2A samples, although it was only significant with LMP1 expression (*P* < 0.05) (Fig. 5A and C). With the addition of inhibitors, Rab7 was significantly decreased by both LMP1 and LMP2A compared to their pBabe controls (*P* = 0.05 and 0.01, respectively). Western transfers also suggested that Rab11-FIP1 was significantly increased by LMP1 in the presence of inhibitors, in agreement with the MS/MS data. Interestingly, Rab11 was also shown to be increased based on immunoblotting, with significant variability (Fig. 5D and E).

## DISCUSSION

This is the first analysis of the effects of the EBV proteins LMP1 and LMP2A on the cellular proteome. As both proteins bind ubiquitin ligases, to identify proteins potentially regulated through this mechanism, the proteomes were also assessed in the presence of inhibitors of both ubiquitination and proteasome-mediated degradation. Not surprisingly, this analysis identified considerably more proteins in the presence of inhibitors that are apparently regulated by LMP1 or LMP2A through effects on ubiquitination or proteasomal processing. Inhibition did not necessarily increase the abundance of proteins, and considerable numbers of the affected proteins decreased in the presence of inhibitors. It is possible that these proteins are regulated at some level by LMP1 or LMP2A through effects on negative regulators of expression which are not degraded with proteasomal inhibition. Additionally, the inability to deubiquitinate proteins could also result in their degradation through standard lysosomal mechanisms.

However, the differences in the levels of expression suggest that LMP1 and LMP2A modulate the ubiquitination of these proteins. Both proteins also affected multiple ubiquitin-conjugating enzymes and peptidases, which could affect the specificity for ubiquitin targeting. It is known that LMP1 activates NF-κB through effects on the function of the canonical NF-κB inhibitor IκBα, and the data presented here indicate that it is highly ubiquitinated in the presence of LMP1 (64). Additionally, LMP1 also promotes the proteasome-mediated processing of NF-κB p100 into p52 in the noncanonical activation of NF-κB. Thus, it is not surprising that there would be little overlap between proteins regulated by NF-κB whose abundance is affected in the presence or absence of inhibitors. It is difficult to predict how the increase or decrease of specific proteins in the absence of deubiquitination and proteasomal degradation would modulate their activity *in vivo*. However, these data identified many proteins that are likely affected by LMP1 and LMP2A through ubiquitination and proteasomal turnover. Interestingly, multiple specific proteasome components were affected, and the effects of the viral proteins on these components may contribute to proteasome function and specificity.

The effects of LMP1 and LMP2A on cellular expression have previously largely been identified by changes in transcription. Although most of these data have been determined in EBV-infected lymphocytes, comparison of these data to existing transcriptional data identified several genes in common. Approximately 39% of genes modulated by LMP2A without inhibitors have been shown to be affected transcriptionally, while 29% with inhibitors were also identified transcriptionally (65, 66). Similarly, 28% of genes identified in transcriptional studies were identified in the proteome in the LMP1-expressing cells (3). For both LMP1 and LMP2A, the effects on protein abundance did not necessarily agree with effects identified on transcription, which is in keeping with some of the known mechanisms of transcriptional activation. However, comparison with the ENCODE data set indicated that for LMP1 and LMP2A with or without inhibitors, more than 80% of the proteins were genes previously identified in chromatin immunoprecipitation studies for relA (p65). Both LMP1 and LMP2A have known effects on NF-κB, and these findings indicate that both viral proteins affect this pathway directly as well as indirectly, with resulting effects on NF-κB binding.

**FIG 6:**
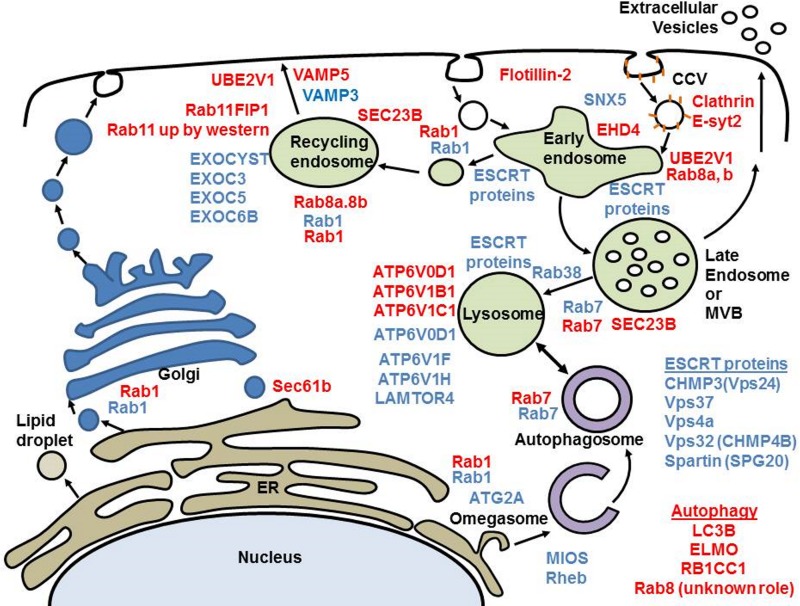
Schematic of vesicle trafficking changes associated with LMP1 or LMP2A expression. Proteins significantly different with LMP1 expression are in red text, and proteins affected by LMP2A are in blue text. Only Rab1 and Rab7 were altered by both LMP1 and LMP2A expression. Although there are few proteins altered by both LMP1 and LMP2A, there is important overlap in the stages of vesicle trafficking predicted to be modulated by their individual expression. Both LMP1 and LMP2A affect the expression of different V-ATPase subunits potentially modulating lysosome activity, and both likely affect mTORC1 signaling via alternate intermediaries. In addition, LMP1 appears to affect the recycling endosome through Rab11/Rab11FIP1, while LMP2A affects the exocyst complex. CCV, Clathrin-coated vesicle; MVB, multivesicular body.

It is interesting to overlay the effects of both LMP1 and LMP2A on the pathways most clearly modulated. Evaluation of the effects on pathways involved in vesicle trafficking (Fig. 6) showed that LMP1 potentially affects the recycling endosome through activation of Rab11/Rab11FIP, while LMP2A potentially modulates activity of the exocyst complex. Additionally, both proteins potentially modulate lysosome activity through effects on ATP6V0D1 and other subunits of the proton pump V-ATPase. Interestingly, our analysis of pathways regulating autophagy predicts a greater effect of LMP1 on autophagosome-to-lysosome flux. It has previously been shown that autophagic flux is blocked in the final steps during the reactivation of EBV from latency. In this case, reduced Rab7 expression correlated with the lack of fusion or flux between lysosomes and autophagosomes (67). LMP1 has also been demonstrated to induce autophagy, increasing the levels of LC3II (MAP1LC3B) (68). In our experiments, LC3 was also significantly elevated and Rab7 significantly reduced in LMP1+Inh samples, with other proteins involved in autophagy, including RB1CC1, ELMO2, TMEM59, and Rab8B, also upregulated in LMP1+Inh samples. Thus, our results expand our knowledge of potential intermediaries regulating autophagosome-lysosome interactions influenced by LMP1.

In these same MCF10A cells, we previously showed that LMP2A activates autophagy to prolong survival during the differentiation process of luminal formation (22). In contrast, the results presented here show that only Rab7 was downregulated in both LMP2A-Inh and LMP2A+Inh samples. This may reflect the considerable differences in growth conditions. The cells analyzed here were adherent cultures with the addition of serum, which likely inhibited the induction of autophagy and differentiation that occurs during luminal formation. However, significant changes in the LMP2A+Inh proteome were associated with vesicle trafficking and included a number of vacuolar ATPase subunits, exocyst subunits, Rab1, Rab38, VPS24 (CHMP3), VPS37B, and VPS4A. The majority of these proteins were downregulated, suggesting a dysregulation of vesicle trafficking not previously described with LMP2A.

The importance of virus-mediated effects on the ubiquitin system was clearly revealed in initial studies demonstrating that growth-transforming viral proteins such as E6 induced growth through specific effects and interactions with ubiquitination components, such as E6AP. The data presented here revealed that the EBV oncogenes also likely affect the abundance of many proteins through effects on ubiquitination. Additionally, these proteomics data suggest altered regulation of both endosomal vesicle trafficking and autophagosome-to-lysosome flux with the expression of LMP1 or LMP2A. The ability to affect vesicle formation and trafficking would also contribute to the regulation of many signaling complexes, such as those that are associated with endocytosed receptors or occur at the vesicle membrane. It will be important to further dissect how these virus-mediated effects on protein ubiquitination alter specific protein localization.

## MATERIALS AND METHODS

### Cell culture.

MCF10A breast epithelial cells expressing LMP1, LMP2A, or pBabe vector (21) were cultured in Dulbecco’s modified Eagle medium with F-12 medium (Gibco, Grand Island, NY) supplemented with 5% horse serum, 20 ng/ml recombinant human epidermal growth factor (rhEGF), 100 ng/ml cholera toxin, 500 µg/ml hydrocortisone, 10 µg/ml insulin, and 1% penicillin/streptomycin in a humidified growth chamber with 5% CO_2_. Each treatment/experimental group was plated in 6 replicates in 15 cm dishes. Treatment groups consisted of pBabe-, LMP1- or LMP2A-expressing cells untreated or incubated for 6 h with the DUB and proteasome inhibitors *N*-ethylmaleimide (2 µM final concentration) and MG-132 (10 µM final concentration), respectively.

### Sample preparation.

Cells were lysed at 4°C in denaturing lysis buffer (8 M urea, 50 mM Tris [pH 7.5], 150 mM NaCl, 1 mM EDTA) with the addition of protease inhibitors (Complete EDTA-free; Roche) and DUB and proteasome inhibitors when applicable. The resulting lysates were centrifuged at 13,000 × *g* for 15 min at 4°C. Protein concentrations were estimated using a colorimetric protein assay (Pierce 660 nm; Thermo Scientific). For each treatment group, 5 mg of protein was reduced with 5 mM dithiothreitol (DTT) for 45 min at room temperature, followed by 10 mM *N*-ethylmaleimide for 30 min while protected from light. Samples were digested with 5 µg LysC (1:100 ratio; Promega) for 4 h at 37°C, then diluted to 2 M urea (final) by using 50 mM Tris (pH 7.5) and digested with 100 µg sequencing-grade trypsin (1:50 ratio; Promega) overnight at 37°C.

### Peptide desalting and fractionation.

Following digestion, samples were acidified with formic acid (FA) to a final concentration of 0.5% and consequently desalted using a 500-mg C_18_ Sep-Pak Vac RC cartridge (Waters) according to the manufacturer’s instructions.

To increase the depth of coverage and quantitation of these lysates, the trypsin-digested desalted samples were fractionated using strong cation exchange (SCX) chromatography, collecting 32 fractions in each run. These fractions were then recombined noncontiguously into 8 final fractions per sample, such that each resulting fraction contained a less complex mixture of peptides with a maximum range of charges. Each fraction was subsequently analyzed by nano-liquid chromatography (nano-LC) run in-line with MS/MS identification of peptides. Samples were fractionated by basic RPLC on an XBridge 4.6- by 250-mm C_18_ column (Waters).

### LC-MS/MS and label-free quantitative proteomics.

Samples were resuspended in 0.1% formic acid. Identification and quantitation of proteins were performed via RPLC-MS/MS on a two-dimensional nano-LC Ultra system coupled to an LTQ-Orbitrap Velos mass spectrometer (Thermo Scientific). Separation of the tryptic peptides was achieved with a 70-min linear gradient of 2 to 30% buffer B in buffer A at 200 nl/min, where buffer A was an aqueous solution of 0.1% formic acid and buffer B was a solution of 0.1% formic acid in acetonitrile (ACN).

Mass spectra were processed, and peptide identification was performed by using the Andromeda search engine found in MaxQuant software version 2.2.1. Peptides were identified using a peptide false-discovery rate (FDR) of 0.05 and a protein FDR of 0.01 with at least 2 unique peptides. Label-free quantitation (LFQ) was calculated by utilizing the measured area under the curve (AUC) of *m/z* and a retention time-aligned extracted ion chromatogram for each peptide. All 4 replicates (biological and technical) of each sample group were included in the LFQ experimental design, with protein-level quantitation and normalization performed using unique and razor peptide features. Statistical analysis was performed using Perseus (version 1.4.1.3), and the differences in expression profiles were further analyzed using Ingenuity Pathway Analysis software.
