# Supplementary material for: Global Proteomic Changes Induced by the Epstein-Barr Virus Oncoproteins Latent Membrane Protein 1 and 2A
Source: mBio. 2018 Jun 19;9(3):e00959-18. doi: 10.1128/mBio.00959-18 (PMC6016245; doi:10.1128/mBio.00959-18)
Supplement: TABLE S2 [file mbo003183928st2.pdf]

Supplemental Table-2

LMP1-no Inhibitors: Autophagy (P = 1)

		LMP1 no Inhs		LMP2 no Inhs		LMP1 with Inhs		LMP2 with Inhs	
**Symbol**	**Entrez Gene Name**	**p-value**	**Fold Change**	**p-value**	**Fold Change**	**p-value**	**Fold Change**	**p-value**	**Fold Change**
None									

LMP2-no Inhibitors: Autophagy (P = 1)

		LMP1 no Inhs		LMP2 no Inhs		LMP1 with Inhs		LMP2 with Inhs	
**Symbol**	**Entrez Gene Name**	**p-value**	**Fold Change**	**p-value**	**Fold Change**	**p-value**	**Fold Change**	**p-value**	**Fold Change**
RAB7A	RAB7A, member RAS oncogene family	8.84E-02	-1.314	4.01E-02	-1.687	4.68E-02	-1.305	3.69E-02	-1.902

LMP1-with Inhibitors: Autophagy (P = 0.00219)

		LMP1 no Inhs		LMP2 no Inhs		LMP1 with Inhs		LMP2 with Inhs	
**Symbol**	**Entrez Gene Name**	**p-value**	**Fold Change**	**p-value**	**Fold Change**	**p-value**	**Fold Change**	**p-value**	**Fold Change**
ARSA	arylsulfatase A	1.00E+00	0	1.00E+00	0	5.36E-03	-1.419	7.43E-01	1.121
ATG2A	autophagy related 2A	1.00E+00	0	1.00E+00	0	9.39E-03	-3.435	2.44E-02	-1.216
BAG3	BCL2-associated athanogene 3	3.91E-01	1.292	1.46E-01	1.807	1.74E-03	1.712	2.66E-01	-1.176
CASP1	caspase 1, apoptosis-related cysteine peptidase	1.00E+00	1.079	1.57E-01	-1.776	3.91E-02	1.376	4.70E-01	-1.299
CAST	calpastatin	5.69E-01	1.357	1.59E-01	2.535	6.79E-03	1.556	2.02E-01	1.279
EIF2S1	eukaryotic translation initiation factor 2, subunit 1 alpha, 35kDa	1.87E-01	1.365	7.04E-01	1.067	2.93E-03	-1.354	7.24E-01	-1.028
HDAC2	histone deacetylase 2	1.00E+00	-3.841	1.00E+00	-1.241	4.63E-02	-1.22	4.45E-01	-1.123
IKBKG	inhibitor of kappa light polypeptide gene enhancer in B-cells, kinase gamma	1.00E+00	1.673	1.00E+00	1.769	5.03E-03	1.742	2.19E-01	1.327
MAP1LC3B	microtubule-associated protein 1 light chain 3 beta					2.18E-02	4.19	1.00E+00	
MAP2K2	mitogen-activated protein kinase kinase 2	1.00E+00	-1.03	1.00E+00	1.079	2.57E-02	-1.397	2.02E-02	-1.306
MTMR3	myotubularin related protein 3					1.29E-02	6.957	1.00E+00	-11.459
PLA2G4A	phospholipase A2, group IVA (cytosolic, calcium-dependent)	1.00E+00	0	1.00E+00	0	4.44E-02	-1.379	3.22E-01	1.211
RAB7A	RAB7A, member RAS oncogene family	8.84E-02	-1.314	4.01E-02	-1.687	4.68E-02	-1.305	3.69E-02	-1.902
RB1CC1	RB1-inducible coiled-coil 1	1.00E+00	0	1.00E+00	0	3.58E-02	1.808	5.38E-02	1.256
SMYD3	SET and MYND domain containing 3	1.00E+00	0	1.00E+00	0	1.27E-02	-1.56	4.29E-01	1.101
SOD1	superoxide dismutase 1, soluble	4.63E-01	2.67	7.65E-01	1.356	1.68E-02	2.011	6.23E-01	-1.434
TBC1D4	TBC1 domain family, member 4	5.93E-01	-1.311	3.31E-01	1.669	3.87E-02	-1.69	7.34E-01	-1.071
TBK1	TANK-binding kinase 1	1.00E+00	1.058	1.00E+00	0	4.25E-02	-1.305	3.19E-01	-1.093
TLR3	toll-like receptor 3					4.57E-02	-1.3	4.35E-02	-2.468
TOP1MT	topoisomerase (DNA) I, mitochondrial					3.86E-02	1.338	7.10E-02	2.347
ULK3	unc-51 like kinase 3					4.74E-02	2.199	3.33E-01	1.17

LMP2-with Inhs Autophagy (P = 1)

		LMP1 no Inhs		LMP2 no Inhs		LMP1 with Inhs		LMP2 with Inhs	
**Symbol**	**Entrez Gene Name**	**p-value**	**Fold Change**	**p-value**	**Fold Change**	**p-value**	**Fold Change**	**p-value**	**Fold Change**
ATG2A	autophagy related 2A	1.00E+00	0	1.00E+00	0	9.39E-03	-3.435	2.44E-02	-1.216
MAP2K2	mitogen-activated protein kinase kinase 2	1.00E+00	-1.03	1.00E+00	1.079	2.57E-02	-1.397	2.02E-02	-1.306
RAB7A	RAB7A, member RAS oncogene family	8.84E-02	-1.314	4.01E-02	-1.687	4.68E-02	-1.305	3.69E-02	-1.902
TLR3	toll-like receptor 3					4.57E-02	-1.3	4.35E-02	-2.468

LMP1 - no inhibitors: Autophagy (manually curated)

Symbol	Entrez Gene Name	p-value	Fold Change
VBP1	von Hippel-Lindau binding protein 1	1.50E-02	-4.055781694
CLIC4	chloride intracellular channel 4	4.78E-02	1.729643845
UBE2V1;TMEM189 (UBE2V1B)	ubiquitin-conjugating enzyme E2 variant 1	1.10E-03	1.739125446
NDRG1	N-myc downstream regulated 1	1.89E-04	2.065999636

LMP2 - no inhibitors: Autophagy (manually curated)

Symbol	Entrez Gene Name	p-value	Fold Change
NDRG1	N-myc downstream regulated 1	3.24E-02	2.278556272

LMP1 - with inhibitors: Autophagy (manually curated)

Symbol	Entrez Gene Name	p-value	Fold Change
ELMO2	Engulfment and cell motility protein 2	3.89E-02	1.267055971
RAB8A	Ras-related protein Rab-8A	2.23E-02	1.300652663
RAB8B	Ras-related protein Rab-8B	3.45E-02	1.900816849
RAC2	Ras-related C3 botulinum toxin substrate 2	1.04E-03	1.398859457
TMEM59	Transmembrane protein 59	3.67E-02	1.527766906
WDR6	WD repeat-containing protein 6	2.82E-02	-1.777276375

LMP2 - with inhibitors: Autophagy (manually curated)

Symbol	Entrez Gene Name	p-value	Fold Change
LAMTOR4	Ragulator complex protein LAMTOR4	6.46E-03	-1.623568981
RAB1B	Ras-related protein Rab-1B	3.47E-02	-1.29326287
RAB38	Ras-related protein Rab-38	8.78E-03	1.575185125

LMP1-no Inhibitors: Autophagy of Cells (P = 0.0279)

Symbol	Entrez Gene Name	LMP1 no Inhs		LMP2 no Inhs		LMP1 with Inhs		LMP2 with Inhs	
		p-value	Fold Change	p-value	Fold Change	p-value	Fold Change	p-value	Fold Change
CAPN1	calpain 1, (mu/I) large subunit	4.38E-02	-1.239	9.13E-01	1.017	1.09E-01	1.277	8.11E-01	1.037
CAPNS1	calpain, small subunit 1	1.07E-02	1.386	1.35E-01	1.316	2.97E-01	1.078	1.28E-01	-1.125
EIF2AK2	eukaryotic translation initiation factor 2-alpha kinase 2	3.04E-02	-1.756	5.90E-01	1.081	5.77E-01	-1.088	3.84E-01	-1.125
KRT18	keratin 18	1.12E-02	-1.391	3.43E-01	-1.225	2.47E-01	1.202	3.85E-01	1.150
RAB1A	RAB1A, member RAS oncogene family	2.65E-02	1.585	5.74E-01	-1.775	6.03E-01	-1.222	5.66E-01	-1.260

LMP2-no Inhibitors: Autophagy of Cells (P = 1)

Symbol	Entrez Gene Name	LMP1 no Inhs		LMP2 no Inhs		LMP1 with Inhs		LMP2 with Inhs	
		p-value	Fold Change	p-value	Fold Change	p-value	Fold Change	p-value	Fold Change
None									

LMP1-with Inhibitors: Autophagy of Cells (P = 1)

Symbol	Entrez Gene Name	LMP1 no Inhs		LMP2 no Inhs		LMP1 with Inhs		LMP2 with Inhs	
		p-value	Fold Change	p-value	Fold Change	p-value	Fold Change	p-value	Fold Change
None									

LMP2-with Inhibitors: Autophagy of Cells (P = 1)

Symbol	Entrez Gene Name	LMP1 no Inhs		LMP2 no Inhs		LMP1 with Inhs		LMP2 with Inhs	
		p-value	Fold Change	p-value	Fold Change	p-value	Fold Change	p-value	Fold Change
None									

LMP1-no Inhibitors: Autophagy of Tumor Cell Lines (P = 0.0126)

		LMP1 no Inhs		LMP2 no Inhs		LMP1 with Inhs		LMP2 with Inhs	
**Symbol**	**Entrez Gene Name**	**p-value**	**Fold Change**	**p-value**	**Fold Change**	**p-value**	**Fold Change**	**p-value**	**Fold Change**
CAPN1	calpain 1, (mu/l) large subunit	4.38E-02	-1.239	9.13E-01	1.017	1.09E-01	1.277	8.11E-01	1.037
CAPNS1	calpain, small subunit 1	1.07E-02	1.386	1.35E-01	1.316	2.97E-01	1.078	1.28E-01	-1.125
KRT18	keratin 18	1.12E-02	-1.391	3.43E-01	-1.225	2.47E-01	1.202	3.85E-01	1.15
RAB1A	RAB1A, member RAS oncogene family	2.65E-02	1.585	5.74E-01	-1.775	6.03E-01	-1.222	5.66E-01	-1.26

LMP2-no Inhibitors: Autophagy of Tumor Cell Lines (P = 1)

		LMP1 no Inhs		LMP2 no Inhs		LMP1 with Inhs		LMP2 with Inhs	
**Symbol**	**Entrez Gene Name**	**p-value**	**Fold Change**	**p-value**	**Fold Change**	**p-value**	**Fold Change**	**p-value**	**Fold Change**
None									

LMP1 with Inhibitors: Autophagy of Tumor Cell Lines (P = 1)

		LMP1 no Inhs		LMP2 no Inhs		LMP1 with Inhs		LMP2 with Inhs	
**Symbol**	**Entrez Gene Name**	**p-value**	**Fold Change**	**p-value**	**Fold Change**	**p-value**	**Fold Change**	**p-value**	**Fold Change**
None									

LMP2 with Inhibitors: Autophagy of Tumor Cell Lines (P = 1)

		LMP1 no Inhs		LMP2 no Inhs		LMP1 with Inhs		LMP2 with Inhs	
**Symbol**	**Entrez Gene Name**	**p-value**	**Fold Change**	**p-value**	**Fold Change**	**p-value**	**Fold Change**	**p-value**	**Fold Change**
None									

LMP1 - no inhibitors: Fusion of Autophagosomes (P = 1)

		LMP1 no Inhs		LMP2 no Inhs		LMP1 with Inhs		LMP2 with Inhs	
**Symbol**	**Entrez Gene Name**	**p-value**	**Fold Change**	**p-value**	**Fold Change**	**p-value**	**Fold Change**	**p-value**	**Fold Change**
None									

LMP2 - no inhibitors: Fusion of Autophagosomes (P = 0.0154)

		LMP1 no Inhs		LMP2 no Inhs		LMP1 with Inhs		LMP2 with Inhs	
**Symbol**	**Entrez Gene Name**	**p-value**	**Fold Change**	**p-value**	**Fold Change**	**p-value**	**Fold Change**	**p-value**	**Fold Change**
RAB7A	RAB7A, member RAS oncogene family	8.84E-02	-1.314	4.01E-02	-1.687	4.68E-02	-1.305	3.69E-02	-1.902

LMP1 - with inhibitors: Fusion of Autophagosomes (P = 1)

		LMP1 no Inhs		LMP2 no Inhs		LMP1 with Inhs		LMP2 with Inhs	
**Symbol**	**Entrez Gene Name**	**p-value**	**Fold Change**	**p-value**	**Fold Change**	**p-value**	**Fold Change**	**p-value**	**Fold Change**
RAB7A	RAB7A, member RAS oncogene family	8.84E-02	-1.314	4.01E-02	-1.687	4.68E-02	-1.305	3.69E-02	-1.902

LMP2 - with inhibitors: Fusion of Autophagosomes (P = 1)

		LMP1 no Inhs		LMP2 no Inhs		LMP1 with Inhs		LMP2 with Inhs	
**Symbol**	**Entrez Gene Name**	**p-value**	**Fold Change**	**p-value**	**Fold Change**	**p-value**	**Fold Change**	**p-value**	**Fold Change**
RAB7A	RAB7A, member RAS oncogene family	8.84E-02	-1.314	4.01E-02	-1.687	4.68E-02	-1.305	3.69E-02	-1.902

LMP1-no Inhibitors: Endocytosis (P = 1)

		LMP1 no Inhs		LMP2 no Inhs		LMP1 with Inhs		LMP2 with Inhs	
**Symbol**	**Entrez Gene Name**	**p-value**	**Fold Change**	**p-value**	**Fold Change**	**p-value**	**Fold Change**	**p-value**	**Fold Change**
None									

LMP2-no Inhibitors: Endocytosis (P = 1)

		LMP1 no Inhs		LMP2 no Inhs		LMP1 with Inhs		LMP2 with Inhs	
**Symbol**	**Entrez Gene Name**	**p-value**	**Fold Change**	**p-value**	**Fold Change**	**p-value**	**Fold Change**	**p-value**	**Fold Change**
None									

LMP1-with Inhibitors: Endocytosis (P = 0.000229)

		LMP1 no Inhs		LMP2 no Inhs		LMP1 with Inhs		LMP2 with Inhs	
**Symbol**	**Entrez Gene Name**	**p-value**	**Fold Change**	**p-value**	**Fold Change**	**p-value**	**Fold Change**	**p-value**	**Fold Change**
ACTN4	actinin, alpha 4	3.46E-01	1.264	9.40E-01	1.023	3.67E-04	2.025	5.14E-01	1.040
AP1S1	adaptor-related protein complex 1, sigma 1 subunit	1.00E00	-1.237	1.00E00	1.223	5.48E-03	-1.441	5.03E-02	-1.487
ATG2A	autophagy related 2A	1.00E00	0.000	1.00E00	0.000	9.39E-03	-3.435	2.44E-02	-1.216
ATP6V0D1	ATPase, H+ transporting, lysosomal 38kDa, V0 subunit d1	1.00E00	0.000	1.00E00	0.000	3.25E-02	1.934	4.54E-02	1.720
CAP1	CAP, adenylate cyclase-associated protein 1 (yeast)	1.00E00	3.389	1.00E00	-4.715	4.49E-02	3.202	8.22E-01	-1.155
CCNT1	cyclin T1	1.00E00	0.000	1.00E00	0.000	3.29E-02	-2.021	2.75E-01	-1.373
CD44	CD44 molecule (Indian blood group)	4.40E-01	-2.277	1.34E-01	-3.127	5.16E-03	-1.316	4.93E-01	-1.089
CDK5	cyclin-dependent kinase 5	1.00E00	0.000	1.00E00	0.000	1.64E-02	-1.426	2.70E-01	1.029
CLIC3	chloride intracellular channel 3					1.54E-03	5.988	2.17E-02	-8.334
CYTH2	cytohesin 2	1.00E00	1.312	1.00E00	0.000	3.25E-02	2.162	6.74E-01	-1.104
EPB41L2	erythrocyte membrane protein band 4.1-like 2	1.00E00	0.000	1.00E00	0.000	5.58E-03	-1.850	1.00E00	1.520
EZR	ezrin	4.70E-01	1.533	6.72E-01	1.354	3.55E-02	2.326	9.76E-01	-1.012
HP	haptoglobin					2.21E-02	1.965	6.28E-01	-1.086
HSPA8	heat shock 70kDa protein 8	7.91E-01	1.055	3.99E-01	1.176	4.62E-02	-2.934	5.98E-01	2.678
ICAM1	intercellular adhesion molecule 1	1.00E00	1.102	1.00E00	0.000	1.20E-02	4.093	4.97E-01	-1.175
LGALS3	lectin, galactoside-binding, soluble, 3	2.19E-01	1.602	5.08E-01	-1.695	1.83E-02	2.188	6.11E-01	1.139
MYO6	myosin VI	7.07E-01	1.104	2.96E-01	-1.384	2.53E-02	1.468	7.71E-02	-1.689
PAK1	p21 protein (Cdc42/Rac)-activated kinase 1					3.34E-02	-1.594	1.00E00	
PLA2G4A	phospholipase A2, group IVA (cytosolic, calcium-dependent)	1.00E00	0.000	1.00E00	0.000	4.44E-02	-1.379	3.22E-01	1.211
RAB7A	RAB7A, member RAS oncogene family	8.84E-02	-1.314	4.01E-02	-1.687	4.68E-02	-1.305	3.69E-02	-1.902
RAC2	ras-related C3 botulinum toxin substrate 2 (rho family, small GTP binding protein Rac2)	1.00E00	0.000	1.00E00	0.000	1.04E-03	1.399	7.67E-02	-1.210
RUFY1	RUN and FYVE domain containing 1	7.78E-01	-1.071	5.71E-01	-1.496	1.42E-03	-1.445	6.14E-01	1.079
SNX3	sorting nexin 3	5.64E-01	-1.325	9.25E-01	1.030	1.45E-02	-1.306	4.88E-02	-1.335
TLR3	toll-like receptor 3					4.57E-02	-1.300	4.35E-02	-2.468

LMP2-with Inhibitors: Endocytosis (P = 0.000263)

		LMP1 no Inhs		LMP2 no Inhs		LMP1 with Inhs		LMP2 with Inhs	
**Symbol**	**Entrez Gene Name**	**p-value**	**Fold Change**	**p-value**	**Fold Change**	**p-value**	**Fold Change**	**p-value**	**Fold Change**
ANXA6	annexin A6	1.00E00	0.000	1.00E00	0.000	9.17E-01	1.062	3.45E-02	-1.977
ATG2A	autophagy related 2A	1.00E00	0.000	1.00E00	0.000	9.39E-03	-3.435	2.44E-02	-1.216
ATP5B	ATP synthase, H+ transporting, mitochondrial F1 complex, beta polypeptide	3.44E-01	-1.221	8.35E-01	1.103	1.00E00	-1.570	1.52E-02	-2.313
ATP6V0D1	ATPase, H+ transporting, lysosomal 38kDa, V0 subunit d1	1.00E00	0.000	1.00E00	0.000	3.25E-02	1.934	4.54E-02	1.720
ATP6V1F	ATPase, H+ transporting, lysosomal 14kDa, V1 subunit F	1.00E00	0.000	1.00E00	0.000	6.61E-01	1.174	2.08E-02	-1.398
ATP6V1H	ATPase, H+ transporting, lysosomal 50/57kDa, V1 subunit H	2.75E-01	-2.373	1.00E00	-1.701	4.91E-01	1.126	1.63E-02	-1.311
CD2AP	CD2-associated protein	6.28E-01	1.300	1.59E-01	2.012	8.76E-01	1.007	1.93E-02	-1.208
CLIC3	chloride intracellular channel 3					1.54E-03	5.988	2.17E-02	-8.334

DYRK1A	dual-specificity tyrosine-(Y)-phosphorylation regulated kinase 1A					7.80E-02	-1.271	3.54E-02	-1.395
FMR1	fragile X mental retardation 1	7.18E-02	-2.219	3.05E-01	-1.654	3.63E-01	-1.454	3.98E-02	-1.650
NKAP	NFKB activating protein	1.00E00	0.000	1.00E00	0.000	5.51E-01	1.185	3.79E-02	1.518
PDLIM7	PDZ and LIM domain 7 (enigma)	2.42E-01	1.589	4.20E-01	-1.346	7.52E-01	-1.132	5.62E-03	-1.875
RAB7A	RAB7A, member RAS oncogene family	8.84E-02	-1.314	4.01E-02	-1.687	4.68E-02	-1.305	3.69E-02	-1.902
SCAMP2	secretory carrier membrane protein 2	1.00E00	-1.884	1.00E00	0.000	7.57E-01	-1.075	8.07E-03	-2.146
SCARB2	scavenger receptor class B, member 2	1.00E00	0.000	1.00E00	0.000	5.73E-02	2.970	4.44E-02	2.514
SNX3	sorting nexin 3	5.64E-01	-1.325	9.25E-01	1.030	1.45E-02	-1.306	4.88E-02	-1.335
STIM1	stromal interaction molecule 1	1.00E00	0.000	1.00E00	0.000	4.59E-01	1.167	3.48E-02	-1.984
TLN1	talin 1	6.90E-01	1.081	1.64E-01	-1.316	7.06E-02	1.063	2.74E-02	-1.154
TLR3	toll-like receptor 3					4.57E-02	-1.300	4.35E-02	-2.468

LMP1 - no inhibitors: Endocytosis (manually curated)

Symbol	Entrez Gene Name	p-value	Fold Change
CENPF	centromere protein F, 350/400kDa	3.29E-02	-2.717499934
ATP6V1C1	ATPase, H+ transporting, lysosomal 42kDa, V1 subunit C1	4.86E-02	-1.907228897
SEC23B	Sec23 homolog B (S. cerevisiae)	1.06E-02	-1.810327859
EHD4	EH-domain containing 4	3.96E-02	-1.275960793
ESYT2 (E-Syt2)	extended synaptotagmin-like protein 2	4.36E-03	-1.209342592
MAP4	microtubule-associated protein 4	3.74E-02	1.492165958
RAB1B;RAB1C	RAB1B, member RAS oncogene family	2.65E-02	1.584738721
UBE2V1;TMEM189	ubiquitin-conjugating enzyme E2 variant 1	1.10E-03	1.739125446
HSPB1	heat shock 27kDa protein 1	1.20E-02	3.426533995

LMP2 - no inhibitors: Endocytosis (manually curated)

Symbol	Entrez Gene Name	p-value	Fold Change
MYOF	myoferlin	0.018898616	-2.037951898
EHD4	EH-domain containing 4	0.022249487	-1.300305845
SNX5	sorting nexin 5	0.039402157	1.383029568
SPG20 (Spartin)	spastic paraplegia 20 (Troyer syndrome)	0.001199997	2.185767576
RIN1	Ras and Rab interactor 1	0.011817075	2.719666969

LMP1 - with inhibitors: Endocytosis (manually curated)

Symbol	Entrez Gene Name	p-value	Fold Change
ACTR1A	Alpha-centractin	1.81E-02	-2.395098694
ATP6V1B1	V-type proton ATPase subunit B	2.21E-02	3.881224741
CCT8	T-complex protein 1 subunit theta	9.35E-03	-1.291184584
COPS6	COP9 signalosome complex subunit 6	5.68E-03	-1.935473811
CPNE2	Copine-2	1.96E-02	1.790844494
DCTN2	Dynactin subunit 2	7.02E-07	1.41671649
CAST	Isoform 5 of Calpastatin	6.79E-03	1.555674618
DPYSL2 (CRMP2)	Dihydropyrimidinase-related protein 2	2.49E-03	1.350911587
HSP90AA1	Heat shock protein HSP 90-alpha	3.43E-02	-1.334943391
NISCH	Nischarin	4.48E-02	-1.51707755
RAB11FIP1	Rab11 family-interacting protein 1	2.21E-02	2.94610588
RAB8A	Ras-related protein Rab-8A	2.23E-02	1.300652663
RAB8B	Ras-related protein Rab-8B	3.45E-02	1.900816849
RABEP2	Rab GTPase-binding effector protein 2	3.52E-03	1.433408397
SEC61B	Protein transport protein Sec61 subunit beta	1.98E-02	-1.829414207
SNX24	Isoform 2 of Sorting nexin-24	7.27E-03	1.582019087
SYNGR2	Synaptogyrin-2	2.59E-03	1.884764
TBC1D13	Isoform 2 of TBC1 domain family member 13	2.44E-02	3.209758156
TBC1D4	Isoform 2 of TBC1 domain family member 4	3.87E-02	-1.690175505
TBC1D8	TBC1 domain family member 8	4.02E-02	7.102937893
TBK1	Serine/threonine-protein kinase TBK1	4.25E-02	-1.305041485
TM9SF2	Transmembrane 9 superfamily member 2	8.18E-03	1.671112068
TMEM59	Transmembrane protein 59	3.67E-02	1.527766906
VAMP5	Vesicle-associated membrane protein 5	4.68E-02	-1.960523711

LMP2 - with inhibitors: Endocytosis (manually curated)

Symbol	Entrez Gene Name	p-value	Fold Change

AP4S1	AP-4 complex subunit sigma-1	3.58E-02	1.652048133
CTSA	cathepsin A (Lysosomal protective protein)	5.14E-03	-3.701413478
CTSH	Pro-cathepsin H	5.12E-04	-2.389562152
EXOC3	Exocyst complex component 3	3.09E-02	-1.456172244
EXOC5	Exocyst complex component 5	1.07E-02	-2.130538312
EXOC6B	Exocyst complex component 6B	4.85E-02	-1.809070274
LAMTOR4	Ragulator complex protein LAMTOR4	6.46E-03	-1.623568981
RAB1B	Ras-related protein Rab-1B	3.47E-02	-1.29326287
RAB38	Ras-related protein Rab-38	8.78E-03	1.575185125
SCRN1	Secernin-1	6.02E-05	-2.200461935
SDCBP (MDA-9/Syntenin)	Syntenin-1	2.26E-03	-1.419647959
SNX24	Sorting nexin-24	4.15E-02	-1.626506406
SYNJ2	Synaptojanin-2	4.39E-02	1.937258143
TMED4	Transmembrane emp24 domain-containing protein 4	3.37E-03	-1.475541002
TMEM192	Transmembrane protein 192	3.72E-02	1.936598107
TMEM63B	Transmembrane protein 63B	2.85E-03	5.755350433
TRAPPC1	Trafficking protein particle complex subunit 1	2.03E-02	-1.473162003
VPS37B	Vacuolar protein sorting-associated protein 37B	1.85E-02	-1.485121003

LMP1-no Inhibitors: Fusion of Late Endosomes (P = 1)

Symbol	Entrez Gene Name	LMP1 no Inhs		LMP2 no Inhs		LMP1 with Inhs		LMP2 with Inhs	
		p-value	Fold Change	p-value	Fold Change	p-value	Fold Change	p-value	Fold Change
None									

LMP2-no Inhibitors: Fusion of Late Endosomes (P = 0.0176)

Symbol	Entrez Gene Name	LMP1 no Inhs		LMP2 no Inhs		LMP1 with Inhs		LMP2 with Inhs	
		p-value	Fold Change	p-value	Fold Change	p-value	Fold Change	p-value	Fold Change
RAB7A	RAB7A, member RAS oncogene family	8.84E-02	-1.314	4.01E-02	-1.687	4.68E-02	-1.305	3.69E-02	-1.902

LMP1-with Inhibitors: Fusion of Late Endosomes (P = 1)

Symbol	Entrez Gene Name	p-value	Fold Change	p-value	Fold Change	p-value	Fold Change	p-value	Fold Change
RAB7A	RAB7A, member RAS oncogene family	8.84E-02	-1.314	4.01E-02	-1.687	4.68E-02	-1.305	3.69E-02	-1.902

LMP2-with Inhibitors: Fusion of Late Endosomes (P = 0.0095)

Symbol	Entrez Gene Name	LMP1 no Inhs		LMP2 no Inhs		LMP1 with Inhs		LMP2 with Inhs	
		p-value	Fold Change	p-value	Fold Change	p-value	Fold Change	p-value	Fold Change
C20orf24	chromosome 20 open reading frame 24					2.01E-01	-1.450	4.27E-02	-1.390
RAB7A	RAB7A, member RAS oncogene family	8.84E-02	-1.314	4.01E-02	-1.687	4.68E-02	-1.305	3.69E-02	-1.902
STX8	syntaxin 8	1.00E00	-3.941	1.00E00	-1.525	7.26E-01	-1.081	2.91E-02	-1.653

LMP1-no Inhibitors: Endocytosis by Tumor cell lines (P = 1)

none

LMP2-no Inhibitors: Endocytosis by Tumor cell lines (P = 1)

none

LMP1-with Inhibitors: Endocytosis by Tumor cell lines (P = 1)

		LMP1 no Inhs		LMP2 no Inhs		LMP1 with Inhs		LMP2 with Inhs	
**Symbol**	**Entrez Gene Name**	**p-value**	**Fold Change**	**p-value**	**Fold Change**	**p-value**	**Fold Change**	**p-value**	**Fold Change**
ATP6V0D1	ATPase, H+ transporting, lysosomal 38kDa, V0 subunit d1	1.00E+00	0	1.00E+00	0	3.25E-02	1.934	4.54E-02	1.72
CLIC3	chloride intracellular channel 3					1.54E-03	5.988	2.17E-02	-8.334

LMP2-with Inhibitors: Endocytosis by Tumor cell lines (P = 0.013)

		LMP1 no Inhs		LMP2 no Inhs		LMP1 with Inhs		LMP2 with Inhs	
**Symbol**	**Entrez Gene Name**	**p-value**	**Fold Change**	**p-value**	**Fold Change**	**p-value**	**Fold Change**	**p-value**	**Fold Change**
ATP6V0D1	ATPase, H+ transporting, lysosomal 38kDa, V0 subunit d1	1.00E+00	0	1.00E+00	0	3.25E-02	1.934	4.54E-02	1.72
ATP6V1F	ATPase, H+ transporting, lysosomal 14kDa, V1 subunit F	1.00E+00	0	1.00E+00	0	6.61E-01	1.174	2.08E-02	-1.398
CLIC3	chloride intracellular channel 3					1.54E-03	5.988	2.17E-02	-8.334
NKAP	NFKB activating protein	1.00E+00	0	1.00E+00	0	5.51E-01	1.185	3.79E-02	1.518
SCARB2	scavenger receptor class B, member 2	1.00E+00	0	1.00E+00	0	5.73E-02	2.97	4.44E-02	2.514

Symbol	Entrez Gene Name	LMP1 no Inhs p-value	LMP1 no Inhs Fold Change	LMP2 no Inhs p-value	LMP2 no Inhs Fold Change	LMP1 with Inhs p-value	LMP1 with Inhs Fold Change	LMP2 with Inhs p-value	LMP2 with Inhs Fold Change
	**LMP1-no Inhibitors: Caveolar-Mediated Endocytosis (P = 0.3477)**								
FLOT2	flotillin 2	3.55E-02	-1.664	3.63E-01	-2.093	6.31E-01	1.078	9.33E-01	1.015

LMP2-no Inhibitors: Caveolar-Mediated Endocytosis (P = 1)

None

Symbol	Entrez Gene Name	LMP1 no Inhs p-value	LMP1 no Inhs Fold Change	LMP2 no Inhs p-value	LMP2 no Inhs Fold Change	LMP1 with Inhs p-value	LMP1 with Inhs Fold Change	LMP2 with Inhs p-value	LMP2 with Inhs Fold Change
	**LMP1-with Inhibitors: Caveolar-Mediated Endocytosis (P = 0.0496)**								
ACTG1	actin, gamma 1	4.38E-01	2.212	9.31E-01	1.090	6.29E-03	1.182	1.00E00	-1.548
FLNB	filamin B, beta	1.24E-01	1.607	6.49E-01	1.104	3.33E-02	1.281	1.84E-01	-3.752
HLA-B	major histocompatibility complex, class I, B	1.00E00	-2.445	1.00E00	-1.504	2.06E-02	4.993	1.00E00	1.329
ITGA5	integrin, alpha 5 (fibronectin receptor, alpha polypeptide)	3.64E-01	-2.172	1.00E00	-3.726	4.33E-02	-2.098	2.27E-02	-2.119
ITGB4	integrin, beta 4	5.06E-01	-1.180	7.55E-01	-1.092	3.98E-02	-1.164	1.00E00	-1.292

Symbol	Entrez Gene Name	LMP1 no Inhs p-value	LMP1 no Inhs Fold Change	LMP2 no Inhs p-value	LMP2 no Inhs Fold Change	LMP1 with Inhs p-value	LMP1 with Inhs Fold Change	LMP2 with Inhs p-value	LMP2 with Inhs Fold Change
	**LMP2-with Inhibitors: Caveolar-Mediated Endocytosis (P = 0.4151)**								
ITGA5	integrin, alpha 5 (fibronectin receptor, alpha polypeptide)	3.64E-01	-2.172	1.00E00	-3.726	4.33E-02	-2.098	2.27E-02	-2.119
ITGAE	integrin, alpha E (antigen CD103, human mucosal lymphocyte antigen 1; alpha polypeptide)					7.25E-01	-1.098	4.70E-02	-1.586

LMP1-no Inhibitors: Viral Entry via Endocytic Pathway (P = 0.422)

Symbol	Entrez Gene Name	LMP1 no Inhs		LMP2 no Inhs		LMP1 with Inhs		LMP2 with Inhs	
		p-value	Fold Change	p-value	Fold Change	p-value	Fold Change	p-value	Fold Change
CLTA	clathrin, light chain A	4.43E-02	-2.209	4.56E-01	-1.644	9.74E-01	-1.024	9.20E-01	1.084

LMP2-no Inhibitors: Viral Entry via Endocytic Pathway (P = 1)

None

LMP1-with Inhibitors: Viral Entry via Endocytic Pathway (P = 0.043)

Symbol	Entrez Gene Name	LMP1 no Inhs		LMP2 no Inhs		LMP1 with Inhs		LMP2 with Inhs	
		p-value	Fold Change	p-value	Fold Change	p-value	Fold Change	p-value	Fold Change
ACTG1	actin, gamma 1	4.38E-01	2.212	9.31E-01	1.090	6.29E-03	1.182	1.00E00	-1.548
FLNB	filamin B, beta	1.24E-01	1.607	6.49E-01	1.104	3.33E-02	1.281	1.84E-01	-3.752
HLA-B	major histocompatibility complex, class I, B	1.00E00	-2.445	1.00E00	-1.504	2.06E-02	4.993	1.00E00	1.329
ITGA5	integrin, alpha 5 (fibronectin receptor, alpha polypeptide)	3.64E-01	-2.172	1.00E00	-3.726	4.33E-02	-2.098	2.27E-02	-2.119
ITGB4	integrin, beta 4	5.06E-01	-1.180	7.55E-01	-1.092	3.98E-02	-1.164	1.00E00	-1.292
RAC2	ras-related C3 botulinum toxin substrate 2 (rho family, small GTP binding protein Rac2)	1.00E00	0.000	1.00E00	0.000	1.04E-03	1.399	7.67E-02	-1.210

LMP2-with Inhibitors: Viral Entry via Endocytic Pathway (P = 1)

Symbol	Entrez Gene Name	LMP1 no Inhs		LMP2 no Inhs		LMP1 with Inhs		LMP2 with Inhs	
		p-value	Fold Change	p-value	Fold Change	p-value	Fold Change	p-value	Fold Change
ITGA5	integrin, alpha 5 (fibronectin receptor, alpha polypeptide)	3.64E-01	-2.172	1.00E00	-3.726	4.33E-02	-2.098	2.27E-02	-2.119

LMP1-no Inhibitors: Clathrin-Medaited Endocytosis (P = 0.136)

Symbol	Entrez Gene Name	LMP1 no Inhs p-value	LMP1 no Inhs Fold Change	LMP2 no Inhs p-value	LMP2 no Inhs Fold Change	LMP1 with Inhs p-value	LMP1 with Inhs Fold Change	LMP2 with Inhs p-value	LMP2 with Inhs Fold Change
ARPC4	actin related protein 2/3 complex, subunit 4, 20kDa	1.11E-02	-1.511	9.32E-01	-1.010	5.30E-01	-1.042	2.83E-01	1.177
CLTA	clathrin, light chain A	4.43E-02	-2.209	4.56E-01	-1.644	9.74E-01	-1.024	9.20E-01	1.084
MYO1E	myosin IE	5.17E-04	2.221	1.00E00	2.228	5.39E-01	1.315	9.06E-01	-1.033

LMP2-no Inhibitors: Clathrin-Medaited Endocytosis (P = 0.151)

Symbol	Entrez Gene Name	LMP1 no Inhs p-value	LMP1 no Inhs Fold Change	LMP2 no Inhs p-value	LMP2 no Inhs Fold Change	LMP1 with Inhs p-value	LMP1 with Inhs Fold Change	LMP2 with Inhs p-value	LMP2 with Inhs Fold Change
RAB7A	RAB7A, member RAS oncogene family	8.84E-02	-1.314	4.01E-02	-1.687	4.68E-02	-1.305	3.69E-02	-1.902

LMP1-with Inhibitors: Clathrin-Medaited Endocytosis (P = 0.04)

Symbol	Entrez Gene Name	LMP1 no Inhs p-value	LMP1 no Inhs Fold Change	LMP2 no Inhs p-value	LMP2 no Inhs Fold Change	LMP1 with Inhs p-value	LMP1 with Inhs Fold Change	LMP2 with Inhs p-value	LMP2 with Inhs Fold Change
ACTG1	actin, gamma 1	4.38E-01	2.212	9.31E-01	1.090	6.29E-03	1.182	1.00E00	-1.548
ARPC1A	actin related protein 2/3 complex, subunit 1A, 41kDa	3.33E-01	1.922	7.89E-01	1.212	3.81E-02	1.382	2.37E-01	-1.175
HSPA8	heat shock 70kDa protein 8	7.91E-01	1.055	3.99E-01	1.176	4.62E-02	-2.934	5.98E-01	2.678
ITGA5	integrin, alpha 5 (fibronectin receptor, alpha polypeptide)	3.64E-01	-2.172	1.00E00	-3.726	4.33E-02	-2.098	2.27E-02	-2.119
ITGB4	integrin, beta 4	5.06E-01	-1.180	7.55E-01	-1.092	3.98E-02	-1.164	1.00E00	-1.292
MET	MET proto-oncogene, receptor tyrosine kinase	1.00E00	0.000	1.00E00	0.000	4.99E-02	-1.277	6.91E-01	1.046
MYO6	myosin VI	7.07E-01	1.104	2.96E-01	-1.384	2.53E-02	1.468	7.71E-02	-1.689
RAB7A	RAB7A, member RAS oncogene family	8.84E-02	-1.314	4.01E-02	-1.687	4.68E-02	-1.305	3.69E-02	-1.902

LMP2-with Inhibitors: Clathrin-Medaited Endocytosis (P = 0.0028)

Symbol	Entrez Gene Name	LMP1 no Inhs p-value	LMP1 no Inhs Fold Change	LMP2 no Inhs p-value	LMP2 no Inhs Fold Change	LMP1 with Inhs p-value	LMP1 with Inhs Fold Change	LMP2 with Inhs p-value	LMP2 with Inhs Fold Change
ACTR2	ARP2 actin-related protein 2 homolog (yeast)	3.55E-01	1.594	9.05E-01	-1.028	8.73E-01	-1.010	4.13E-02	-1.124
CD2AP	CD2-associated protein	6.28E-01	1.300	1.59E-01	2.012	8.76E-01	1.007	1.93E-02	-1.208
CHP1	calcineurin-like EF-hand protein 1					9.61E-01	1.008	5.71E-03	-1.477
ITGA5	integrin, alpha 5 (fibronectin receptor, alpha polypeptide)	3.64E-01	-2.172	1.00E00	-3.726	4.33E-02	-2.098	2.27E-02	-2.119
PPP3R1	protein phosphatase 3, regulatory subunit B, alpha	1.00E00	0.000	1.00E00	0.000	1.33E-01	-1.373	1.02E-02	-1.585
RAB7A	RAB7A, member RAS oncogene family	8.84E-02	-1.314	4.01E-02	-1.687	4.68E-02	-1.305	3.69E-02	-1.902

LMP1-no Inhibitors: Viral Exit from Cells (P = 1)

None

LMP2-no Inhibitors: Viral Exit from Cells (P = 0.0864)

		LMP1 no Inhs		LMP2 no Inhs		LMP1 with Inhs		LMP2 with Inhs	
**Symbol**	**Entrez Gene Name**	**p-value**	**Fold Change**	**p-value**	**Fold Change**	**p-value**	**Fold Change**	**p-value**	**Fold Change**
CHMP4B	charged multivesicular body protein 4B	1.18E-01	-2.014	1.76E-02	-1.900	2.24E-01	1.859	6.93E-01	1.222

LMP1-with Inhibitors: Viral Exit from Cells (P = 1)

		LMP1 no Inhs		LMP2 no Inhs		LMP1 with Inhs		LMP2 with Inhs	
**Symbol**	**Entrez Gene Name**	**p-value**	**Fold Change**	**p-value**	**Fold Change**	**p-value**	**Fold Change**	**p-value**	**Fold Change**
ACTG1	actin, gamma 1	4.38E-01	2.212	9.31E-01	1.090	6.29E-03	1.182	1.00E00	-1.548

LMP2-with Inhibitors: Viral Exit from Cells (P = 0.0431)

		LMP1 no Inhs		LMP2 no Inhs		LMP1 with Inhs		LMP2 with Inhs	
**Symbol**	**Entrez Gene Name**	**p-value**	**Fold Change**	**p-value**	**Fold Change**	**p-value**	**Fold Change**	**p-value**	**Fold Change**
CHMP3	charged multivesicular body protein 3	1.00E00	-1.412	1.00E00	1.152	2.51E-01	-1.233	3.16E-03	-1.427
PDCD6IP	programmed cell death 6 interacting protein	1.16E-01	1.678	5.08E-01	1.196	1.39E-01	-1.067	2.27E-02	-1.149
VPS4A	vacuolar protein sorting 4 homolog A (S. cerevisiae)	9.14E-01	1.023	8.11E-01	-1.087	1.52E-01	-1.253	4.18E-02	-1.437

LMP1-no Inhibitors: Macropinocytosis (P = 1)

None

LMP2-no Inhibitors: Macropinocytosis (P = 0.033)

		LMP1 no Inhs		LMP2 no Inhs		LMP1 with Inhs		LMP2 with Inhs	
**Symbol**	**Entrez Gene Name**	**p-value**	**Fold Change**	**p-value**	**Fold Change**	**p-value**	**Fold Change**	**p-value**	**Fold Change**
SNX5	sorting nexin 5	6.37E-01	-1.110	3.94E-02	1.383	3.59E-01	-1.231	9.97E-01	1.001

LMP1-with Inhibitors: Macropinocytosis (P = 1)

		LMP1 no Inhs		LMP2 no Inhs		LMP1 with Inhs		LMP2 with Inhs	
**Symbol**	**Entrez Gene Name**	**p-value**	**Fold Change**	**p-value**	**Fold Change**	**p-value**	**Fold Change**	**p-value**	**Fold Change**
ACTN4	actinin, alpha 4	3.46E-01	1.264	9.40E-01	1.023	3.67E-04	2.025	5.14E-01	1.040
ANKFY1	ankyrin repeat and FYVE domain containing 1	2.94E-01	-1.400	1.00E00	1.026	2.63E-02	1.360	9.71E-01	-1.007
ITGA5	integrin, alpha 5 (fibronectin receptor, alpha polypeptide)	3.64E-01	-2.172	1.00E00	-3.726	4.33E-02	-2.098	2.27E-02	-2.119
ITGB4	integrin, beta 4	5.06E-01	-1.180	7.55E-01	-1.092	3.98E-02	-1.164	1.00E00	-1.292
MET	MET proto-oncogene, receptor tyrosine kinase	1.00E00	0.000	1.00E00	0.000	4.99E-02	-1.277	6.91E-01	1.046
PAK1	p21 protein (Cdc42/Rac)-activated kinase 1					3.34E-02	-1.594	1.00E00	

LMP2-with Inhibitors: Macropinocytosis (P = 1)

		LMP1 no Inhs		LMP2 no Inhs		LMP1 with Inhs		LMP2 with Inhs	
**Symbol**	**Entrez Gene Name**	**p-value**	**Fold Change**	**p-value**	**Fold Change**	**p-value**	**Fold Change**	**p-value**	**Fold Change**
ITGA5	integrin, alpha 5 (fibronectin receptor, alpha polypeptide)	3.64E-01	-2.172	1.00E00	-3.726	4.33E-02	-2.098	2.27E-02	-2.119

Fc-gamma receptor-mediated phagocytosis in macrophages and monocytes

LMP1-no Inhibitors: FcR-Phagocytosis (P = 0.426)

		LMP1 no Inhs		LMP2 no Inhs		LMP1 with Inhs		LMP2 with Inhs	
**Symbol**	**Entrez Gene Name**	**p-value**	**Fold Change**	**p-value**	**Fold Change**	**p-value**	**Fold Change**	**p-value**	**Fold Change**
ARPC4	actin related protein 2/3 complex, subunit 4, 20kDa	1.11E-02	-1.511	9.32E-01	-1.010	5.30E-01	-1.042	2.83E-01	1.177

LMP2-no Inhibitors: FcR-Phagocytosis (P = 0.180)

		LMP1 no Inhs		LMP2 no Inhs		LMP1 with Inhs		LMP2 with Inhs	
**Symbol**	**Entrez Gene Name**	**p-value**	**Fold Change**	**p-value**	**Fold Change**	**p-value**	**Fold Change**	**p-value**	**Fold Change**
VAMP3	vesicle-associated membrane protein 3	5.35E-01	-1.358	1.14E-03	-1.687	2.83E-01	-1.167	5.43E-02	1.295

LMP1-withInhibitors: FcR-Phagocytosis (P = 0.043)

		LMP1 no Inhs		LMP2 no Inhs		LMP1 with Inhs		LMP2 with Inhs	
**Symbol**	**Entrez Gene Name**	**p-value**	**Fold Change**	**p-value**	**Fold Change**	**p-value**	**Fold Change**	**p-value**	**Fold Change**
ACTG1	actin, gamma 1	4.38E-01	2.212	9.31E-01	1.090	6.29E-03	1.182	1.00E00	-1.548
ARPC1A	actin related protein 2/3 complex, subunit 1A, 41kDa	3.33E-01	1.922	7.89E-01	1.212	3.81E-02	1.382	2.37E-01	-1.175
EZR	ezrin	4.70E-01	1.533	6.72E-01	1.354	3.55E-02	2.326	9.76E-01	-1.012
NCK1	NCK adaptor protein 1	1.00E00	1.371	1.00E00	1.688	3.97E-03	1.850	3.49E-01	1.681
PAK1	p21 protein (Cdc42/Rac)-activated kinase 1					3.34E-02	-1.594	1.00E00	
RAC2	ras-related C3 botulinum toxin substrate 2 (rho family, small GTP binding protein Rac2)	1.00E00	0.000	1.00E00	0.000	1.04E-03	1.399	7.67E-02	-1.210

LMP2-with Inhibitors: FcR-Phagocytosis (P = 0.114)

		LMP1 no Inhs		LMP2 no Inhs		LMP1 with Inhs		LMP2 with Inhs	
**Symbol**	**Entrez Gene Name**	**p-value**	**Fold Change**	**p-value**	**Fold Change**	**p-value**	**Fold Change**	**p-value**	**Fold Change**
ACTR2	ARP2 actin-related protein 2 homolog (yeast)	3.55E-01	1.594	9.05E-01	-1.028	8.73E-01	-1.010	4.13E-02	-1.124
PXN	paxillin	2.26E-01	1.287	1.27E-01	-1.543	3.24E-01	1.546	1.68E-02	-1.271
RPS6KB1	ribosomal protein S6 kinase, 70kDa, polypeptide 1	1.00E00	1.861	1.00E00	1.983	1.04E-01	-1.675	3.62E-02	-1.878
TLN1	talin 1	6.90E-01	1.081	1.64E-01	-1.316	7.06E-02	1.063	2.74E-02	-1.154
