# Supplementary material for: Global Proteomic Changes Induced by the Epstein-Barr Virus Oncoproteins Latent Membrane Protein 1 and 2A
Source: mBio. 2018 Jun 19;9(3):e00959-18. doi: 10.1128/mBio.00959-18 (PMC6016245; doi:10.1128/mBio.00959-18)
Supplement: TABLE S3 [file mbo003183928st3.pdf]

Supplemental Table-3

Symbol	Entrez Gene Name	LMP1 no Inhs p-value	LMP1 no Inhs Fold Change	LMP2 no Inhs p-value	LMP2 no Inhs Fold Change	LMP1 with Inhs p-value	LMP1 with Inhs Fold Change	LMP2 with Inhs p-value	LMP2 with Inhs Fold Change
	**LMP1-no Inhibitors: EIF2 signaling (P = 0.0046)**								
EIF2AK2	eukaryotic translation initiation factor 2-alpha kinase 2	3.04E-02	-1.756	5.90E-01	1.081	5.77E-01	-1.088	3.84E-01	-1.125
EIF2B4	eukaryotic translation initiation factor 2B, subunit 4 delta, 67kDa	1.95E-02	1.233	1.00E00	1.446	3.35E-01	-1.208	5.09E-01	-1.133
EIF3L	eukaryotic translation initiation factor 3, subunit L	7.50E-04	-1.578	3.03E-01	-1.421	1.52E-01	-1.223	1.01E-01	-1.191
EIF4A1	eukaryotic translation initiation factor 4A1	2.22E-02	-1.494	6.00E-01	-1.108	5.36E-03	-1.474	2.21E-03	-1.518
RPL12	ribosomal protein L12	2.67E-02	-1.352	2.12E-01	-1.700	8.90E-01	1.051	6.08E-01	1.225
	**LMP2-noInhibitors: EIF2 signaling (P = 0.342)**								
RPS25	ribosomal protein S25	1.52E-01	1.140	3.99E-02	1.423	8.54E-01	-1.043	4.58E-01	-1.207
	**LMP1-with Inhibitors: EIF2 signaling (P = 0.00019)**								
AGO2	argonaute RISC catalytic component 2	1.00E00	1.578	1.00E00	1.280	1.64E-02	-1.539	1.37E-01	-1.335
EIF2A	eukaryotic translation initiation factor 2A, 65kDa	9.33E-01	-1.021	3.74E-01	1.287	2.67E-02	-1.316	5.59E-02	-1.223
EIF2S1	eukaryotic translation initiation factor 2, subunit 1 alpha, 35kDa	1.87E-01	1.365	7.04E-01	1.067	2.93E-03	-1.354	7.24E-01	-1.028
EIF2S3	eukaryotic translation initiation factor 2, subunit 3 gamma, 52kDa	5.73E-01	-1.064	7.36E-01	1.157	3.03E-02	-1.424	3.71E-01	-1.114
EIF3B	eukaryotic translation initiation factor 3, subunit B	3.42E-01	-1.386	8.48E-01	1.074	1.02E-02	-1.676	2.12E-03	-1.435
EIF3C	eukaryotic translation initiation factor 3, subunit C	5.73E-01	-1.271	7.02E-01	-1.177	9.18E-03	-1.354	1.15E-01	-1.170
EIF3E	eukaryotic translation initiation factor 3, subunit E	2.79E-01	1.270	5.54E-01	-1.191	4.88E-02	-1.981	2.96E-02	-1.483
EIF4A1	eukaryotic translation initiation factor 4A1	2.22E-02	-1.494	6.00E-01	-1.108	5.36E-03	-1.474	2.21E-03	-1.518
EIF4G3	eukaryotic translation initiation factor 4 gamma, 3	1.00E00	-1.319	1.00E00	1.355	2.49E-02	1.703	1.09E-01	-1.517
MAP2K2	mitogen-activated protein kinase kinase 2	1.00E00	-1.030	1.00E00	1.079	2.57E-02	-1.397	2.02E-02	-1.306
PPP1CC	protein phosphatase 1, catalytic subunit, gamma isozyme	1.00E00	0.000	1.00E00	-3.795	3.37E-02	1.428	2.43E-03	1.450
RPL8	ribosomal protein L8	6.23E-01	-1.990	8.60E-01	-1.250	2.05E-02	-1.439	6.73E-03	-1.280
RPL9	ribosomal protein L9	2.39E-01	-1.319	4.97E-01	-1.221	1.47E-03	-1.543	2.01E-02	-1.259
RPL31	ribosomal protein L31	9.13E-01	1.098	9.27E-01	-1.066	2.51E-02	-1.318	7.37E-02	-1.195
RPL32	ribosomal protein L32	6.55E-01	-1.348	7.98E-01	-1.100	6.12E-03	-1.235	1.22E-01	-1.203
	**LMP2-with Inhibitors: EIF2 signaling (P = 1.656 x 10^{-5})**								
EIF5	eukaryotic translation initiation factor 5	3.46E-01	1.602	6.89E-01	1.275	1.22E-01	-1.603	4.54E-02	-1.458
EIF3B	eukaryotic translation initiation factor 3, subunit B	3.42E-01	-1.386	8.48E-01	1.074	1.02E-02	-1.676	2.12E-03	-1.435
EIF3E	eukaryotic translation initiation factor 3, subunit E	2.79E-01	1.270	5.54E-01	-1.191	4.88E-02	-1.981	2.96E-02	-1.483
EIF3K	eukaryotic translation initiation factor 3, subunit K	4.74E-01	-1.416	6.92E-01	-1.129	3.06E-01	-1.210	4.08E-02	-1.481
EIF3M	eukaryotic translation initiation factor 3, subunit M	8.12E-01	-1.073	1.13E-01	-1.587	2.70E-01	-1.202	4.54E-02	-1.495
EIF4A1	eukaryotic translation initiation factor 4A1	2.22E-02	-1.494	6.00E-01	-1.108	5.36E-03	-1.474	2.21E-03	-1.518
EIF4A2	eukaryotic translation initiation factor 4A2	1.00E00	0.000	1.00E00	0.000	5.80E-01	1.224	4.97E-02	-2.874
MAP2K2	mitogen-activated protein kinase kinase 2	1.00E00	-1.030	1.00E00	1.079	2.57E-02	-1.397	2.02E-02	-1.306
PPP1CC	protein phosphatase 1, catalytic subunit, gamma isozyme	1.00E00	0.000	1.00E00	-3.795	3.37E-02	1.428	2.43E-03	1.450
RPL8	ribosomal protein L8	6.23E-01	-1.990	8.60E-01	-1.250	2.05E-02	-1.439	6.73E-03	-1.280
RPL9	ribosomal protein L9	2.39E-01	-1.319	4.97E-01	-1.221	1.47E-03	-1.543	2.01E-02	-1.259
RPL26	ribosomal protein L26	6.22E-01	-1.402	8.23E-01	1.176	5.30E-01	1.102	1.21E-02	1.300
RPS27	ribosomal protein S27	8.41E-01	1.058	2.67E-01	-2.025	4.95E-01	1.154	3.28E-02	1.640
RPS15A	ribosomal protein S15a	6.98E-02	-1.702	3.15E-01	-1.182	9.19E-01	-1.093	2.40E-03	1.238

LMP1-no Inhibitors: eIF4-p70S6K signaling (P = 0.056)

Symbol	Entrez Gene Name	LMP1 no Inhs p-value	Fold Change	LMP2 no Inhs p-value	Fold Change	LMP1 with Inhs p-value	Fold Change	LMP2 with Inhs p-value	Fold Change
EIF2B4	eukaryotic translation initiation factor 2B, subunit 4 delta, 67kDa	1.95E-02	1.233	1.00E00	1.446	3.35E-01	-1.208	5.09E-01	-1.133
EIF3L	eukaryotic translation initiation factor 3, subunit L	7.50E-04	-1.578	3.03E-01	-1.421	1.52E-01	-1.223	1.01E-01	-1.191
EIF4A1	eukaryotic translation initiation factor 4A1	2.22E-02	-1.494	6.00E-01	-1.108	5.36E-03	-1.474	2.21E-03	-1.518

LMP2-no Inhibitors: eIF4-p70S6K signaling (P = 0.284)

Symbol	Entrez Gene Name	LMP1 no Inhs p-value	Fold Change	LMP2 no Inhs p-value	Fold Change	LMP1 with Inhs p-value	Fold Change	LMP2 with Inhs p-value	Fold Change
RPS25	ribosomal protein S25	1.52E-01	1.140	3.99E-02	1.423	8.54E-01	-1.043	4.58E-01	-1.207

LMP1-with Inhibitors: eIF4-p70S6K signaling (P = 0.00256)

Symbol	Entrez Gene Name	LMP1 no Inhs p-value	Fold Change	LMP2 no Inhs p-value	Fold Change	LMP1 with Inhs p-value	Fold Change	LMP2 with Inhs p-value	Fold Change
AGO2	argonaute RISC catalytic component 2	1.00E00	1.578	1.00E00	1.280	1.64E-02	-1.539	1.37E-01	-1.335
EIF2A	eukaryotic translation initiation factor 2A, 65kDa	9.33E-01	-1.021	3.74E-01	1.287	2.67E-02	-1.316	5.59E-02	-1.223
EIF2S1	eukaryotic translation initiation factor 2, subunit 1 alpha, 35kDa	1.87E-01	1.365	7.04E-01	1.067	2.93E-03	-1.354	7.24E-01	-1.028
EIF2S3	eukaryotic translation initiation factor 2, subunit 3 gamma, 52kDa	5.73E-01	-1.064	7.36E-01	1.157	3.03E-02	-1.424	3.71E-01	-1.114
EIF3B	eukaryotic translation initiation factor 3, subunit B	3.42E-01	-1.386	8.48E-01	1.074	1.02E-02	-1.676	2.12E-03	-1.435
EIF3C	eukaryotic translation initiation factor 3, subunit C	5.73E-01	-1.271	7.02E-01	-1.177	9.18E-03	-1.354	1.15E-01	-1.170
EIF3E	eukaryotic translation initiation factor 3, subunit E	2.79E-01	1.270	5.54E-01	-1.191	4.88E-02	-1.981	2.96E-02	-1.483
EIF4A1	eukaryotic translation initiation factor 4A1	2.22E-02	-1.494	6.00E-01	-1.108	5.36E-03	-1.474	2.21E-03	-1.518
EIF4G3	eukaryotic translation initiation factor 4 gamma, 3	1.00E00	-1.319	1.00E00	1.355	2.49E-02	1.703	1.09E-01	-1.517
ITGA5	integrin, alpha 5 (fibronectin receptor, alpha polypeptide)	3.64E-01	-2.172	1.00E00	-3.726	4.33E-02	-2.098	2.27E-02	-2.119
MAP2K2	mitogen-activated protein kinase kinase 2	1.00E00	-1.030	1.00E00	1.079	2.57E-02	-1.397	2.02E-02	-1.306

LMP2-with Inhibitors: eIF4-p70S6K signaling (P = 6.5 x 10^{-6})

Symbol	Entrez Gene Name	LMP1 no Inhs p-value	Fold Change	LMP2 no Inhs p-value	Fold Change	LMP1 with Inhs p-value	Fold Change	LMP2 with Inhs p-value	Fold Change
EIF3B	eukaryotic translation initiation factor 3, subunit B	3.42E-01	-1.386	8.48E-01	1.074	1.02E-02	-1.676	2.12E-03	-1.435
EIF3E	eukaryotic translation initiation factor 3, subunit E	2.79E-01	1.270	5.54E-01	-1.191	4.88E-02	-1.981	2.96E-02	-1.483
EIF3K	eukaryotic translation initiation factor 3, subunit K	4.74E-01	-1.416	6.92E-01	-1.129	3.06E-01	-1.210	4.08E-02	-1.481
EIF3M	eukaryotic translation initiation factor 3, subunit M	8.12E-01	-1.073	1.13E-01	-1.587	2.70E-01	-1.202	4.54E-02	-1.495
EIF4A1	eukaryotic translation initiation factor 4A1	2.22E-02	-1.494	6.00E-01	-1.108	5.36E-03	-1.474	2.21E-03	-1.518
EIF4A2	eukaryotic translation initiation factor 4A2	1.00E00	0.000	1.00E00	0.000	5.80E-01	1.224	4.97E-02	-2.874
EIF4EBP2	eukaryotic translation initiation factor 4E binding protein 2					2.53E-01	1.480	2.60E-02	-4.372
ITGA5	integrin, alpha 5 (fibronectin receptor, alpha polypeptide)	3.64E-01	-2.172	1.00E00	-3.726	4.33E-02	-2.098	2.27E-02	-2.119
MAP2K2	mitogen-activated protein kinase kinase 2	1.00E00	-1.030	1.00E00	1.079	2.57E-02	-1.397	2.02E-02	-1.306
PPP2R2A	protein phosphatase 2, regulatory subunit B, alpha	4.81E-01	1.459	8.73E-01	1.147	2.85E-01	-1.232	7.48E-03	-1.358
RPS27	ribosomal protein S27	8.41E-01	1.058	2.67E-01	-2.025	4.95E-01	1.154	3.28E-02	1.640
RPS15A	ribosomal protein S15a	6.98E-02	-1.702	3.15E-01	-1.182	9.19E-01	-1.093	2.40E-03	1.238
RPS6KB1	ribosomal protein S6 kinase, 70kDa, polypeptide 1	1.00E00	1.861	1.00E00	1.983	1.04E-01	-1.675	3.62E-02	-1.878

LMP1-no Inhibitor: Expression of mRNA (P = 1) | LMP1 no Inhs | LMP2 no Inhs | LMP1 with Inhs | LMP2 with Inhs

none

LMP2-no Inhibitor: Expression of mRNA (P = 1) | LMP1 no Inhs | LMP2 no Inhs | LMP1 with Inhs | LMP2 with Inhs

none

LMP1-with Inhibitor: Expression of mRNA (P = 0.00069)

		LMP1 no Inhs		LMP2 no Inhs		LMP1 with Inhs		LMP2 with Inhs	
**Symbol**	**Entrez Gene Name**	**p-value**	**Fold Change**	**p-value**	**Fold Change**	**p-value**	**Fold Change**	**p-value**	**Fold Change**
AGO2	argonaute RISC catalytic component 2	1.00E+00	1.578	1.00E+00	1.28	1.64E-02	-1.539	1.37E-01	-1.335
DARS	aspartyl-tRNA synthetase	9.00E-01	-1.035	8.33E-01	1.067	4.73E-02	-1.295	2.76E-01	-1.181
EIF2A	eukaryotic translation initiation factor 2A, 65kDa	9.33E-01	-1.021	3.74E-01	1.287	2.67E-02	-1.316	5.59E-02	-1.223
EIF2S1	eukaryotic translation initiation factor 2, subunit 1 alpha, 35kDa	1.87E-01	1.365	7.04E-01	1.067	2.93E-03	-1.354	7.24E-01	-1.028
EIF2S3	eukaryotic translation initiation factor 2, subunit 3 gamma, 52kDa	5.73E-01	-1.064	7.36E-01	1.157	3.03E-02	-1.424	3.71E-01	-1.114
EIF3B	eukaryotic translation initiation factor 3, subunit B	3.42E-01	-1.386	8.48E-01	1.074	1.02E-02	-1.676	2.12E-03	-1.435
EIF3C	eukaryotic translation initiation factor 3, subunit C	5.73E-01	-1.271	7.02E-01	-1.177	9.18E-03	-1.354	1.15E-01	-1.17
EIF3E	eukaryotic translation initiation factor 3, subunit E	2.79E-01	1.27	5.54E-01	-1.191	4.88E-02	-1.981	2.96E-02	-1.483
EIF4G3	eukaryotic translation initiation factor 4 gamma, 3	1.00E+00	-1.319	1.00E+00	1.355	2.49E-02	1.703	1.09E-01	-1.517
EIF4H	eukaryotic translation initiation factor 4H	4.51E-01	-1.362	5.80E-01	-1.527	2.09E-03	-1.36	1.23E-02	-1.316
EPRS	glutamyl-prolyl-tRNA synthetase	8.40E-02	-1.28	4.72E-01	-1.176	3.69E-02	-1.214	6.05E-01	1.029
FARSB	phenylalanyl-tRNA synthetase, beta subunit	1.46E-02	-1.791	5.16E-01	-1.186	3.76E-02	-1.225	8.77E-01	1.013
FXR1	fragile X mental retardation, autosomal homolog 1	5.43E-01	-1.461	8.26E-01	1.117	1.51E-02	-1.295	8.38E-03	-1.647
GRB7	growth factor receptor-bound protein 7	1.00E+00	0	1.00E+00	0	3.20E-02	1.867	1.00E+00	1.058
GTF2H3	general transcription factor IIH, polypeptide 3, 34kDa					1.85E-02	-2.117	3.93E-01	-1.393
PABPC4	poly(A) binding protein, cytoplasmic 4 (inducible form)	7.73E-01	-1.126	7.92E-01	1.027	6.62E-03	-1.267	7.58E-01	-1.046
TIMP3	TIMP metallopeptidase inhibitor 3	1.00E+00	-2.697	1.00E+00	0	4.13E-03	-1.852	4.38E-01	-1.628

LMP2-with Inhibitor: Expression of mRNA (P = 0.00148)

		LMP1 no Inhs		LMP2 no Inhs		LMP1 with Inhs		LMP2 with Inhs	
**Symbol**	**Entrez Gene Name**	**p-value**	**Fold Change**	**p-value**	**Fold Change**	**p-value**	**Fold Change**	**p-value**	**Fold Change**
ATF1	activating transcription factor 1					5.54E-01	1.108	3.67E-02	1.33
DAPK3	death-associated protein kinase 3	1.00E+00	0	1.00E+00	0	2.18E-01	1.285	4.16E-02	-1.518
EIF5	eukaryotic translation initiation factor 5	3.46E-01	1.602	6.89E-01	1.275	1.22E-01	-1.603	4.54E-02	-1.458
EIF3B	eukaryotic translation initiation factor 3, subunit B	3.42E-01	-1.386	8.48E-01	1.074	1.02E-02	-1.676	2.12E-03	-1.435
EIF3E	eukaryotic translation initiation factor 3, subunit E	2.79E-01	1.27	5.54E-01	-1.191	4.88E-02	-1.981	2.96E-02	-1.483
EIF3K	eukaryotic translation initiation factor 3, subunit K	4.74E-01	-1.416	6.92E-01	-1.129	3.06E-01	-1.21	4.08E-02	-1.481
EIF4A2	eukaryotic translation initiation factor 4A2	1.00E+00	0	1.00E+00	0	5.80E-01	1.224	4.97E-02	-2.874
EIF4EBP2	eukaryotic translation initiation factor 4E binding protein 2					2.53E-01	1.48	2.60E-02	-4.372
EIF4H	eukaryotic translation initiation factor 4H	4.51E-01	-1.362	5.80E-01	-1.527	2.09E-03	-1.36	1.23E-02	-1.316
FMR1	fragile X mental retardation 1	7.18E-02	-2.219	3.05E-01	-1.654	3.63E-01	-1.454	3.98E-02	-1.65
FXR1	fragile X mental retardation, autosomal homolog 1	5.43E-01	-1.461	8.26E-01	1.117	1.51E-02	-1.295	8.38E-03	-1.647
IGF2BP3	insulin-like growth factor 2 mRNA binding protein 3	7.30E-01	1.216	3.56E-03	-1.963	5.05E-01	-1.102	1.29E-02	-2.325
MTIF3	mitochondrial translational initiation factor 3					4.09E-01	1.248	2.99E-02	1.596

LMP1-no Inhibitors: Translation of Protein (P = 0.00025)

Symbol	Entrez Gene Name	LMP1 no Inhs p-value	LMP1 no Inhs Fold Change	LMP2 no Inhs p-value	LMP2 no Inhs Fold Change	LMP1 with Inhs p-value	LMP1 with Inhs Fold Change	LMP2 with Inhs p-value	LMP2 with Inhs Fold Change
EIF2AK2	eukaryotic translation initiation factor 2-alpha kinase 2	3.04E-02	-1.756	5.90E-01	1.081	5.77E-01	-1.088	3.84E-01	-1.125
EIF2B4	eukaryotic translation initiation factor 2B, subunit 4 delta, 67kDa	1.95E-02	1.233	1.00E+00	1.446	3.35E-01	-1.208	5.09E-01	-1.133
EIF3L	eukaryotic translation initiation factor 3, subunit L	7.50E-04	-1.578	3.03E-01	-1.421	1.52E-01	-1.223	1.01E-01	-1.191
EIF4A1	eukaryotic translation initiation factor 4A1	2.22E-02	-1.494	6.00E-01	-1.108	5.36E-03	-1.474	2.21E-03	-1.518
FARSB	phenylalanyl-tRNA synthetase, beta subunit	1.46E-02	-1.791	5.16E-01	-1.186	3.76E-02	-1.225	8.77E-01	1.013
HSPB1	heat shock 27kDa protein 1	1.20E-02	3.427	2.30E-01	1.936	2.29E-01	1.449	9.29E-01	-1.043
NCBP1	nuclear cap binding protein subunit 1, 80kDa	3.83E-02	-2.567	2.13E-01	-2.049	6.56E-01	1.111	5.33E-01	-1.062
TIA1	TIA1 cytotoxic granule-associated RNA binding protein	2.69E-02	1.805	1.00E+00	1.963	9.38E-01	-1.024	6.20E-01	1.141

LMP2-no Inhibitors: Translation of Protein (P = 1)

		LMP1 no Inhs		LMP2 no Inhs		LMP1 with Inhs		LMP2 with Inhs	
none									

LMP1-with Inhibitors: Translation of Protein (P = 3.63×10^{-5})

Symbol	Entrez Gene Name	LMP1 no Inhs p-value	LMP1 no Inhs Fold Change	LMP2 no Inhs p-value	LMP2 no Inhs Fold Change	LMP1 with Inhs p-value	LMP1 with Inhs Fold Change	LMP2 with Inhs p-value	LMP2 with Inhs Fold Change
AGO2	argonaute RISC catalytic component 2	1.00E+00	1.578	1.00E+00	1.28	1.64E-02	-1.539	1.37E-01	-1.335
DARS	aspartyl-tRNA synthetase	9.00E-01	-1.035	8.33E-01	1.067	4.73E-02	-1.295	2.76E-01	-1.181
EIF2A	eukaryotic translation initiation factor 2A, 65kDa	9.33E-01	-1.021	3.74E-01	1.287	2.67E-02	-1.316	5.59E-02	-1.223
EIF2S1	eukaryotic translation initiation factor 2, subunit 1 alpha, 35kDa	1.87E-01	1.365	7.04E-01	1.067	2.93E-03	-1.354	7.24E-01	-1.028
EIF2S3	eukaryotic translation initiation factor 2, subunit 3 gamma, 52kDa	5.73E-01	-1.064	7.36E-01	1.157	3.03E-02	-1.424	3.71E-01	-1.114
EIF3B	eukaryotic translation initiation factor 3, subunit B	3.42E-01	-1.386	8.48E-01	1.074	1.02E-02	-1.676	2.12E-03	-1.435
EIF3C	eukaryotic translation initiation factor 3, subunit C	5.73E-01	-1.271	7.02E-01	-1.177	9.18E-03	-1.354	1.15E-01	-1.17
EIF3E	eukaryotic translation initiation factor 3, subunit E	2.79E-01	1.27	5.54E-01	-1.191	4.88E-02	-1.981	2.96E-02	-1.483
EIF4A1	eukaryotic translation initiation factor 4A1	2.22E-02	-1.494	6.00E-01	-1.108	5.36E-03	-1.474	2.21E-03	-1.518
EIF4G3	eukaryotic translation initiation factor 4 gamma, 3	1.00E+00	-1.319	1.00E+00	1.355	2.49E-02	1.703	1.09E-01	-1.517
EIF4H	eukaryotic translation initiation factor 4H	4.51E-01	-1.362	5.80E-01	-1.527	2.09E-03	-1.36	1.23E-02	-1.316
EPRS	glutamyl-prolyl-tRNA synthetase	8.40E-02	-1.28	4.72E-01	-1.176	3.69E-02	-1.214	6.05E-01	1.029
FARSB	phenylalanyl-tRNA synthetase, beta subunit	1.46E-02	-1.791	5.16E-01	-1.186	3.76E-02	-1.225	8.77E-01	1.013
FXR1	fragile X mental retardation, autosomal homolog 1	5.43E-01	-1.461	8.26E-01	1.117	1.51E-02	-1.295	8.38E-03	-1.647
GRB7	growth factor receptor-bound protein 7	1.00E+00	0	1.00E+00	0	3.20E-02	1.867	1.00E+00	1.058
GTF2H3	general transcription factor IIH, polypeptide 3, 34kDa					1.85E-02	-2.117	3.93E-01	-1.393
HNRNPK	heterogeneous nuclear ribonucleoprotein K	6.23E-01	1.079	6.77E-01	1.057	1.97E-02	-1.205	3.08E-01	-2.032
HSPA1A/H	heat shock 70kDa protein 1A	1.43E-01	1.284	3.50E-01	1.333	2.34E-03	-1.242	1.93E-01	-1.064
NCK1	NCK adaptor protein 1	1.00E+00	1.371	1.00E+00	1.688	3.97E-03	1.85	3.49E-01	1.681
NXF1	nuclear RNA export factor 1	3.09E-01	2.024	6.23E-02	1.906	1.52E-02	-1.445	1.41E-01	-1.309
PABPC4	poly(A) binding protein, cytoplasmic 4 (inducible form)	7.73E-01	-1.126	7.92E-01	1.027	6.62E-03	-1.267	7.58E-01	-1.046

LMP2-with Inhibitors: Translation of Protein (P = 1.11×10^{-5})

Symbol	Entrez Gene Name	LMP1 no Inhs p-value	LMP1 no Inhs Fold Change	LMP2 no Inhs p-value	LMP2 no Inhs Fold Change	LMP1 with Inhs p-value	LMP1 with Inhs Fold Change	LMP2 with Inhs p-value	LMP2 with Inhs Fold Change
BPNT1	3'(2'), 5'-bisphosphate nucleotidase 1	8.47E-01	1.04	5.96E-01	1.109	7.85E-02	1.155	4.49E-02	1.199
DAPK3	death-associated protein kinase 3	1.00E+00	0	1.00E+00	0	2.18E-01	1.285	4.16E-02	-1.518
EIF5	eukaryotic translation initiation factor 5	3.46E-01	1.602	6.89E-01	1.275	1.22E-01	-1.603	4.54E-02	-1.458
EIF3B	eukaryotic translation initiation factor 3, subunit B	3.42E-01	-1.386	8.48E-01	1.074	1.02E-02	-1.676	2.12E-03	-1.435
EIF3E	eukaryotic translation initiation factor 3, subunit E	2.79E-01	1.27	5.54E-01	-1.191	4.88E-02	-1.981	2.96E-02	-1.483
EIF3K	eukaryotic translation initiation factor 3, subunit K	4.74E-01	-1.416	6.92E-01	-1.129	3.06E-01	-1.21	4.08E-02	-1.481
EIF4A1	eukaryotic translation initiation factor 4A1	2.22E-02	-1.494	6.00E-01	-1.108	5.36E-03	-1.474	2.21E-03	-1.518
EIF4A2	eukaryotic translation initiation factor 4A2	1.00E+00	0	1.00E+00	0	5.80E-01	1.224	4.97E-02	-2.874
EIF4EBP2	eukaryotic translation initiation factor 4E binding protein 2					2.53E-01	1.48	2.60E-02	-4.372

EIF4H	eukaryotic translation initiation factor 4H	4.51E-01	-1.362	5.80E-01	-1.527	2.09E-03	-1.36	1.23E-02	-1.316
FMR1	fragile X mental retardation 1	7.18E-02	-2.219	3.05E-01	-1.654	3.63E-01	-1.454	3.98E-02	-1.65
FXR1	fragile X mental retardation, autosomal homolog 1	5.43E-01	-1.461	8.26E-01	1.117	1.51E-02	-1.295	8.38E-03	-1.647
IGF2BP3	insulin-like growth factor 2 mRNA binding protein 3	7.30E-01	1.216	3.56E-03	-1.963	5.05E-01	-1.102	1.29E-02	-2.325
MTIF3	mitochondrial translational initiation factor 3					4.09E-01	1.248	2.99E-02	1.596
MTRF1	mitochondrial translational release factor 1	1.00E+00	0	1.00E+00	0	3.14E-01	1.37	4.93E-02	-2.68
RHEB	Ras homolog enriched in brain	1.00E+00	0	1.00E+00	0	4.60E-01	-1.64	3.30E-02	-1.975
RPS6KB1	ribosomal protein S6 kinase, 70kDa, polypeptide 1	1.00E+00	1.861	1.00E+00	1.983	1.04E-01	-1.675	3.62E-02	-1.878
STAU1	staufen double-stranded RNA binding protein 1	2.56E-01	1.342	4.38E-01	1.344	4.04E-01	1.111	3.33E-02	-1.379

LMP1-noInhs_Translation of mRNA (P = 7.45 x 10^{-5})

Symbol	Entrez Gene Name	LMP1 no Inhs p-value	LMP1 no Inhs Fold Change	LMP2 no Inhs p-value	LMP2 no Inhs Fold Change	LMP1 with Inhs p-value	LMP1 with Inhs Fold Change	LMP2 with Inhs p-value	LMP2 with Inhs Fold Change
EIF2AK2	eukaryotic translation initiation factor 2-alpha kinase 2	3.04E-02	-1.756	5.90E-01	1.081	5.77E-01	-1.088	3.84E-01	-1.125
EIF2B4	eukaryotic translation initiation factor 2B, subunit 4 delta, 67kDa	1.95E-02	1.233	1.00E+00	1.446	3.35E-01	-1.208	5.09E-01	-1.133
EIF3L	eukaryotic translation initiation factor 3, subunit L	7.50E-04	-1.578	3.03E-01	-1.421	1.52E-01	-1.223	1.01E-01	-1.191
FARSB	phenylalanyl-tRNA synthetase, beta subunit	1.46E-02	-1.791	5.16E-01	-1.186	3.76E-02	-1.225	8.77E-01	1.013
HSPB1	heat shock 27kDa protein 1	1.20E-02	3.427	2.30E-01	1.936	2.29E-01	1.449	9.29E-01	-1.043
NCBP1	nuclear cap binding protein subunit 1, 80kDa	3.83E-02	-2.567	2.13E-01	-2.049	6.56E-01	1.111	5.33E-01	-1.062
TIA1	TIA1 cytotoxic granule-associated RNA binding protein	2.69E-02	1.805	1.00E+00	1.963	9.38E-01	-1.024	6.20E-01	1.141

LMP2-noInhs_Translation of mRNA (P = 1)

LMP1 no Inhs | LMP2 no Inhs | LMP1 with Inhs | LMP2 with Inhs

none

LMP1 with Inhs_Translation of mRNA (P = 2.59 x 10^{-5})

Symbol	Entrez Gene Name	LMP1 no Inhs p-value	LMP1 no Inhs Fold Change	LMP2 no Inhs p-value	LMP2 no Inhs Fold Change	LMP1 with Inhs p-value	LMP1 with Inhs Fold Change	LMP2 with Inhs p-value	LMP2 with Inhs Fold Change
AGO2	argonaute RISC catalytic component 2	1.00E+00	1.578	1.00E+00	1.28	1.64E-02	-1.539	1.37E-01	-1.335
DARS	aspartyl-tRNA synthetase	9.00E-01	-1.035	8.33E-01	1.067	4.73E-02	-1.295	2.76E-01	-1.181
EIF2A	eukaryotic translation initiation factor 2A, 65kDa	9.33E-01	-1.021	3.74E-01	1.287	2.67E-02	-1.316	5.59E-02	-1.223
EIF2S1	eukaryotic translation initiation factor 2, subunit 1 alpha, 35kDa	1.87E-01	1.365	7.04E-01	1.067	2.93E-03	-1.354	7.24E-01	-1.028
EIF2S3	eukaryotic translation initiation factor 2, subunit 3 gamma, 52kDa	5.73E-01	-1.064	7.36E-01	1.157	3.03E-02	-1.424	3.71E-01	-1.114
EIF3B	eukaryotic translation initiation factor 3, subunit B	3.42E-01	-1.386	8.48E-01	1.074	1.02E-02	-1.676	2.12E-03	-1.435
EIF3C	eukaryotic translation initiation factor 3, subunit C	5.73E-01	-1.271	7.02E-01	-1.177	9.18E-03	-1.354	1.15E-01	-1.17
EIF3E	eukaryotic translation initiation factor 3, subunit E	2.79E-01	1.27	5.54E-01	-1.191	4.88E-02	-1.981	2.96E-02	-1.483
EIF4G3	eukaryotic translation initiation factor 4 gamma, 3	1.00E+00	-1.319	1.00E+00	1.355	2.49E-02	1.703	1.09E-01	-1.517
EIF4H	eukaryotic translation initiation factor 4H	4.51E-01	-1.362	5.80E-01	-1.527	2.09E-03	-1.36	1.23E-02	-1.316
EPRS	glutamyl-prolyl-tRNA synthetase	8.40E-02	-1.28	4.72E-01	-1.176	3.69E-02	-1.214	6.05E-01	1.029
FARSB	phenylalanyl-tRNA synthetase, beta subunit	1.46E-02	-1.791	5.16E-01	-1.186	3.76E-02	-1.225	8.77E-01	1.013
FXR1	fragile X mental retardation, autosomal homolog 1	5.43E-01	-1.461	8.26E-01	1.117	1.51E-02	-1.295	8.38E-03	-1.647
GRB7	growth factor receptor-bound protein 7	1.00E+00	0	1.00E+00	0	3.20E-02	1.867	1.00E+00	1.058
GTF2H3	general transcription factor IIH, polypeptide 3, 34kDa					1.85E-02	-2.117	3.93E-01	-1.393
PABPC4	poly(A) binding protein, cytoplasmic 4 (inducible form)	7.73E-01	-1.126	7.92E-01	1.027	6.62E-03	-1.267	7.58E-01	-1.046

LMP2 with Inhs_Translation of mRNA (P = 0.00016)

Symbol	Entrez Gene Name	LMP1 no Inhs p-value	LMP1 no Inhs Fold Change	LMP2 no Inhs p-value	LMP2 no Inhs Fold Change	LMP1 with Inhs p-value	LMP1 with Inhs Fold Change	LMP2 with Inhs p-value	LMP2 with Inhs Fold Change
DAPK3	death-associated protein kinase 3	1.00E+00	0	1.00E+00	0	2.18E-01	1.285	4.16E-02	-1.518
EIF5	eukaryotic translation initiation factor 5	3.46E-01	1.602	6.89E-01	1.275	1.22E-01	-1.603	4.54E-02	-1.458
EIF3B	eukaryotic translation initiation factor 3, subunit B	3.42E-01	-1.386	8.48E-01	1.074	1.02E-02	-1.676	2.12E-03	-1.435
EIF3E	eukaryotic translation initiation factor 3, subunit E	2.79E-01	1.27	5.54E-01	-1.191	4.88E-02	-1.981	2.96E-02	-1.483
EIF3K	eukaryotic translation initiation factor 3, subunit K	4.74E-01	-1.416	6.92E-01	-1.129	3.06E-01	-1.21	4.08E-02	-1.481
EIF4A2	eukaryotic translation initiation factor 4A2	1.00E+00	0	1.00E+00	0	5.80E-01	1.224	4.97E-02	-2.874
EIF4EBP2	eukaryotic translation initiation factor 4E binding protein 2					2.53E-01	1.48	2.60E-02	-4.372
EIF4H	eukaryotic translation initiation factor 4H	4.51E-01	-1.362	5.80E-01	-1.527	2.09E-03	-1.36	1.23E-02	-1.316
FMR1	fragile X mental retardation 1	7.18E-02	-2.219	3.05E-01	-1.654	3.63E-01	-1.454	3.98E-02	-1.65
FXR1	fragile X mental retardation, autosomal homolog 1	5.43E-01	-1.461	8.26E-01	1.117	1.51E-02	-1.295	8.38E-03	-1.647
IGF2BP3	insulin-like growth factor 2 mRNA binding protein 3	7.30E-01	1.216	3.56E-03	-1.963	5.05E-01	-1.102	1.29E-02	-2.325
MTIF3	mitochondrial translational initiation factor 3					4.09E-01	1.248	2.99E-02	1.596

LMP1-no Inhibitors: Translation of RNA (P = 1)

	LMP1 no Inhs	LMP2 no Inhs	LMP1 with Inhs	LMP2 with Inhs

None

LMP2-no Inhibitors: Translation of RNA (P = 1)

	LMP1 no Inhs	LMP2 no Inhs	LMP1 with Inhs	LMP2 with Inhs

None

LMP1-with Inhibitors: Translation of RNA (P = 1)

	LMP1 no Inhs	LMP2 no Inhs	LMP1 with Inhs	LMP2 with Inhs

None

LMP2-with Inhibitors: Translation of RNA (P = 4.82 x 10^{-5})

		LMP1 no Inhs		LMP2 no Inhs		LMP1 with Inhs		LMP2 with Inhs	
**Symbol**	**Entrez Gene Name**	**p-value**	**Fold Change**	**p-value**	**Fold Change**	**p-value**	**Fold Change**	**p-value**	**Fold Change**
DAPK3	death-associated protein kinase 3	1.00E+00	0	1.00E+00	0	2.18E-01	1.285	4.16E-02	-1.518
EIF5	eukaryotic translation initiation factor 5	3.46E-01	1.602	6.89E-01	1.275	1.22E-01	-1.603	4.54E-02	-1.458
EIF3B	eukaryotic translation initiation factor 3, subunit B	3.42E-01	-1.386	8.48E-01	1.074	1.02E-02	-1.676	2.12E-03	-1.435
EIF3E	eukaryotic translation initiation factor 3, subunit E	2.79E-01	1.27	5.54E-01	-1.191	4.88E-02	-1.981	2.96E-02	-1.483
EIF3K	eukaryotic translation initiation factor 3, subunit K	4.74E-01	-1.416	6.92E-01	-1.129	3.06E-01	-1.21	4.08E-02	-1.481
EIF4A2	eukaryotic translation initiation factor 4A2	1.00E+00	0	1.00E+00	0	5.80E-01	1.224	4.97E-02	-2.874
EIF4EBP2	eukaryotic translation initiation factor 4E binding protein 2					2.53E-01	1.48	2.60E-02	-4.372
EIF4H	eukaryotic translation initiation factor 4H	4.51E-01	-1.362	5.80E-01	-1.527	2.09E-03	-1.36	1.23E-02	-1.316
FMR1	fragile X mental retardation 1	7.18E-02	-2.219	3.05E-01	-1.654	3.63E-01	-1.454	3.98E-02	-1.65
FXR1	fragile X mental retardation, autosomal homolog 1	5.43E-01	-1.461	8.26E-01	1.117	1.51E-02	-1.295	8.38E-03	-1.647
IGF2BP3	insulin-like growth factor 2 mRNA binding protein 3	7.30E-01	1.216	3.56E-03	-1.963	5.05E-01	-1.102	1.29E-02	-2.325
MTIF3	mitochondrial translational initiation factor 3					4.09E-01	1.248	2.99E-02	1.596
MTRF1	mitochondrial translational release factor 1	1.00E+00	0	1.00E+00	0	3.14E-01	1.37	4.93E-02	-2.68

LMP1-no Inhibitors: Expression of Protein (P = 0.00047)

Symbol	Entrez Gene Name	LMP1 no Inhs p-value	LMP1 no Inhs Fold Change	LMP2 no Inhs p-value	LMP2 no Inhs Fold Change	LMP1 with Inhs p-value	LMP1 with Inhs Fold Change	LMP2 with Inhs p-value	LMP2 with Inhs Fold Change
EIF2AK2	eukaryotic translation initiation factor 2-alpha kinase 2	3.04E-02	-1.756	5.90E-01	1.081	5.77E-01	-1.088	3.84E-01	-1.125
EIF2B4	eukaryotic translation initiation factor 2B, subunit 4 delta, 67kDa	1.95E-02	1.233	1.00E+00	1.446	3.35E-01	-1.208	5.09E-01	-1.133
EIF3L	eukaryotic translation initiation factor 3, subunit L	7.50E-04	-1.578	3.03E-01	-1.421	1.52E-01	-1.223	1.01E-01	-1.191
EIF4A1	eukaryotic translation initiation factor 4A1	2.22E-02	-1.494	6.00E-01	-1.108	5.36E-03	-1.474	2.21E-03	-1.518
FARSB	phenylalanyl-tRNA synthetase, beta subunit	1.46E-02	-1.791	5.16E-01	-1.186	3.76E-02	-1.225	8.77E-01	1.013
HSPB1	heat shock 27kDa protein 1	1.20E-02	3.427	2.30E-01	1.936	2.29E-01	1.449	9.29E-01	-1.043
MYBBP1A	MYB binding protein (P160) 1a	1.16E-02	-1.524	5.32E-01	-1.07	3.38E-02	-1.329	5.27E-02	-1.161
NCBP1	nuclear cap binding protein subunit 1, 80kDa	3.83E-02	-2.567	2.13E-01	-2.049	6.56E-01	1.111	5.33E-01	-1.062
TIA1	TIA1 cytotoxic granule-associated RNA binding protein	2.69E-02	1.805	1.00E+00	1.963	9.38E-01	-1.024	6.20E-01	1.141

LMP2-no Inhibitors: Expression of Protein (P = 1)

LMP1 no Inhs | LMP2 no Inhs | LMP1 with Inhs | LMP2 with Inhs

none

LMP1-with Inhibitors: Expression of Protein (P = 0.0002)

Symbol	Entrez Gene Name	LMP1 no Inhs p-value	LMP1 no Inhs Fold Change	LMP2 no Inhs p-value	LMP2 no Inhs Fold Change	LMP1 with Inhs p-value	LMP1 with Inhs Fold Change	LMP2 with Inhs p-value	LMP2 with Inhs Fold Change
AGO2	argonaute RISC catalytic component 2	1.00E+00	1.578	1.00E+00	1.28	1.64E-02	-1.539	1.37E-01	-1.335
ANXA1	annexin A1	1.02E-01	1.681	1.66E-01	1.297	1.25E-02	1.272	7.77E-01	-1.021
DARS	aspartyl-tRNA synthetase	9.00E-01	-1.035	8.33E-01	1.067	4.73E-02	-1.295	2.76E-01	-1.181
EIF2A	eukaryotic translation initiation factor 2A, 65kDa	9.33E-01	-1.021	3.74E-01	1.287	2.67E-02	-1.316	5.59E-02	-1.223
EIF2S1	eukaryotic translation initiation factor 2, subunit 1 alpha, 35kDa	1.87E-01	1.365	7.04E-01	1.067	2.93E-03	-1.354	7.24E-01	-1.028
EIF2S3	eukaryotic translation initiation factor 2, subunit 3 gamma, 52kDa	5.73E-01	-1.064	7.36E-01	1.157	3.03E-02	-1.424	3.71E-01	-1.114
EIF3B	eukaryotic translation initiation factor 3, subunit B	3.42E-01	-1.386	8.48E-01	1.074	1.02E-02	-1.676	2.12E-03	-1.435
EIF3C	eukaryotic translation initiation factor 3, subunit C	5.73E-01	-1.271	7.02E-01	-1.177	9.18E-03	-1.354	1.15E-01	-1.17
EIF3E	eukaryotic translation initiation factor 3, subunit E	2.79E-01	1.27	5.54E-01	-1.191	4.88E-02	-1.981	2.96E-02	-1.483
EIF4A1	eukaryotic translation initiation factor 4A1	2.22E-02	-1.494	6.00E-01	-1.108	5.36E-03	-1.474	2.21E-03	-1.518
EIF4G3	eukaryotic translation initiation factor 4 gamma, 3	1.00E+00	-1.319	1.00E+00	1.355	2.49E-02	1.703	1.09E-01	-1.517
EIF4H	eukaryotic translation initiation factor 4H	4.51E-01	-1.362	5.80E-01	-1.527	2.09E-03	-1.36	1.23E-02	-1.316
EPRS	glutamyl-prolyl-tRNA synthetase	8.40E-02	-1.28	4.72E-01	-1.176	3.69E-02	-1.214	6.05E-01	1.029
FARSB	phenylalanyl-tRNA synthetase, beta subunit	1.46E-02	-1.791	5.16E-01	-1.186	3.76E-02	-1.225	8.77E-01	1.013
FXR1	fragile X mental retardation, autosomal homolog 1	5.43E-01	-1.461	8.26E-01	1.117	1.51E-02	-1.295	8.38E-03	-1.647
GRB7	growth factor receptor-bound protein 7	1.00E+00	0	1.00E+00	0	3.20E-02	1.867	1.00E+00	1.058
GTF2H3	general transcription factor IIH, polypeptide 3, 34kDa					1.85E-02	-2.117	3.93E-01	-1.393
HNRNPK	heterogeneous nuclear ribonucleoprotein K	6.23E-01	1.079	6.77E-01	1.057	1.97E-02	-1.205	3.08E-01	-2.032
HSPA1A/HSPA1B	heat shock 70kDa protein 1A	1.43E-01	1.284	3.50E-01	1.333	2.34E-03	-1.242	1.93E-01	-1.064
MYBBP1A	MYB binding protein (P160) 1a	1.16E-02	-1.524	5.32E-01	-1.07	3.38E-02	-1.329	5.27E-02	-1.161
NCK1	NCK adaptor protein 1	1.00E+00	1.371	1.00E+00	1.688	3.97E-03	1.85	3.49E-01	1.681
NXF1	nuclear RNA export factor 1	3.09E-01	2.024	6.23E-02	1.906	1.52E-02	-1.445	1.41E-01	-1.309
PABPC4	poly(A) binding protein, cytoplasmic 4 (inducible form)	7.73E-01	-1.126	7.92E-01	1.027	6.62E-03	-1.267	7.58E-01	-1.046
SOD1	superoxide dismutase 1, soluble	4.63E-01	2.67	7.65E-01	1.356	1.68E-02	2.011	6.23E-01	-1.434

LMP2-with Inhibitors: Expression of Protein (P = 8.29×10^{-5})

Symbol	Entrez Gene Name	LMP1 no Inhs p-value	LMP1 no Inhs Fold Change	LMP2 no Inhs p-value	LMP2 no Inhs Fold Change	LMP1 with Inhs p-value	LMP1 with Inhs Fold Change	LMP2 with Inhs p-value	LMP2 with Inhs Fold Change
BPNT1	3'(2'), 5'-bisphosphate nucleotidase 1	8.47E-01	1.04	5.96E-01	1.109	7.85E-02	1.155	4.49E-02	1.199
DAPK3	death-associated protein kinase 3	1.00E+00	0	1.00E+00	0	2.18E-01	1.285	4.16E-02	-1.518
EIF5	eukaryotic translation initiation factor 5	3.46E-01	1.602	6.89E-01	1.275	1.22E-01	-1.603	4.54E-02	-1.458
EIF3B	eukaryotic translation initiation factor 3, subunit B	3.42E-01	-1.386	8.48E-01	1.074	1.02E-02	-1.676	2.12E-03	-1.435
EIF3E	eukaryotic translation initiation factor 3, subunit E	2.79E-01	1.27	5.54E-01	-1.191	4.88E-02	-1.981	2.96E-02	-1.483

EIF3K	eukaryotic translation initiation factor 3, subunit K	4.74E-01	-1.416	6.92E-01	-1.129	3.06E-01	-1.21	4.08E-02	-1.481
EIF4A1	eukaryotic translation initiation factor 4A1	2.22E-02	-1.494	6.00E-01	-1.108	5.36E-03	-1.474	2.21E-03	-1.518
EIF4A2	eukaryotic translation initiation factor 4A2	1.00E+00	0	1.00E+00	0	5.80E-01	1.224	4.97E-02	-2.874
EIF4EBP2	eukaryotic translation initiation factor 4E binding protein 2					2.53E-01	1.48	2.60E-02	-4.372
EIF4H	eukaryotic translation initiation factor 4H	4.51E-01	-1.362	5.80E-01	-1.527	2.09E-03	-1.36	1.23E-02	-1.316
FAS	Fas cell surface death receptor	1.00E+00	-1.102	1.00E+00	0	1.24E-01	-1.23	3.08E-03	-1.72
FMR1	fragile X mental retardation 1	7.18E-02	-2.219	3.05E-01	-1.654	3.63E-01	-1.454	3.98E-02	-1.65
FXR1	fragile X mental retardation, autosomal homolog 1	5.43E-01	-1.461	8.26E-01	1.117	1.51E-02	-1.295	8.38E-03	-1.647
IGF2BP3	insulin-like growth factor 2 mRNA binding protein 3	7.30E-01	1.216	3.56E-03	-1.963	5.05E-01	-1.102	1.29E-02	-2.325
MTIF3	mitochondrial translational initiation factor 3					4.09E-01	1.248	2.99E-02	1.596
MTRF1	mitochondrial translational release factor 1	1.00E+00	0	1.00E+00	0	3.14E-01	1.37	4.93E-02	-2.68
NFKB1	nuclear factor of kappa light polypeptide gene enhancer in B-cells 1	7.54E-01	-1.28	8.35E-01	1.177	9.42E-02	1.205	1.63E-02	1.226
RHEB	Ras homolog enriched in brain	1.00E+00	0	1.00E+00	0	4.60E-01	-1.64	3.30E-02	-1.975
RPS6KB1	ribosomal protein S6 kinase, 70kDa, polypeptide 1	1.00E+00	1.861	1.00E+00	1.983	1.04E-01	-1.675	3.62E-02	-1.878
STAU1	staufen double-stranded RNA binding protein 1	2.56E-01	1.342	4.38E-01	1.344	4.04E-01	1.111	3.33E-02	-1.379

	LMP1-no Inhibitors: Expression of DNA (P = 1)	LMP1 no Inhs		LMP2 no Inhs		LMP1 with Inhs		LMP2 with Inhs	
none									

	LMP2-no Inhibitors: Expression of DNA (P = 1)	LMP1 no Inhs		LMP2 no Inhs		LMP1 with Inhs		LMP2 with Inhs	
none									

	LMP1-with Inhibitors: Expression of DNA (P = 0.0043)	LMP1 no Inhs		LMP2 no Inhs		LMP1 with Inhs		LMP2 with Inhs	
**Symbol**	**Entrez Gene Name**	**p-value**	**Fold Change**	**p-value**	**Fold Change**	**p-value**	**Fold Change**	**p-value**	**Fold Change**
AATF	apoptosis antagonizing transcription factor	3.12E-01	-1.37	4.76E-01	1.207	3.10E-02	-1.21	4.32E-01	-1.074
ABT1	activator of basal transcription 1	1.00E+00	0	1.00E+00	0	4.46E-02	-1.639	1.10E-02	-2.15
ARHGEF11	Rho guanine nucleotide exchange factor (GEF) 11	1.00E+00	0	1.00E+00	0	1.83E-02	1.612	6.07E-01	-1.132
ATF7	activating transcription factor 7	1.00E+00	0	1.00E+00	0	4.54E-02	-2.237	3.51E-01	-1.311
ATF7IP	activating transcription factor 7 interacting protein	1.00E+00	0	1.00E+00	0	2.41E-02	2.025	4.06E-01	-1.148
ATXN7L3	ataxin 7-like 3					5.71E-03	-1.231	4.19E-02	1.368
BCLAF1	BCL2-associated transcription factor 1	2.45E-01	1.432	8.27E-01	-1.07	1.38E-02	-1.654	8.10E-01	1.037
BPTF	bromodomain PHD finger transcription factor	1.00E+00	0	1.00E+00	0	3.55E-02	-2.04	1.83E-01	-1.528
BRD8	bromodomain containing 8	1.00E+00	-2.574	1.00E+00	1.393	2.52E-02	1.56	3.21E-01	1.296
CBX1	chromobox homolog 1	1.00E+00	-2.276	1.00E+00	1.612	2.66E-02	-1.502	2.12E-01	-1.267
CD44	CD44 molecule (Indian blood group)	4.40E-01	-2.277	1.34E-01	-3.127	5.16E-03	-1.316	4.93E-01	-1.089
CHD1	chromodomain helicase DNA binding protein 1	1.00E+00	0	1.00E+00	0	1.19E-02	-3.257	2.45E-02	-2.212
CHD2	chromodomain helicase DNA binding protein 2	1.00E+00	1.064	1.00E+00	0	2.19E-02	-1.743	3.30E-01	-1.109
CNN2	calponin 2	3.14E-02	1.55	3.23E-01	1.388	8.13E-04	1.753	6.26E-02	1.246
CRABP2	cellular retinoic acid binding protein 2	1.00E+00	0	1.00E+00	0	2.01E-02	10.196	5.80E-01	1.753
CTBP1	C-terminal binding protein 1	5.49E-01	-1.175	5.46E-01	1.181	3.58E-02	-1.525	2.73E-01	-1.224
CXXC1	CXXC finger protein 1	1.00E+00	0	1.00E+00	0	1.74E-02	1.379	1.37E-01	1.277
DDX58	DEAD (Asp-Glu-Ala-Asp) box polypeptide 58					2.50E-02	4.906	9.74E-01	-1.016
DMAP1	DNA methyltransferase 1 associated protein 1	1.00E+00	0	1.00E+00	0	3.08E-02	-2.365	1.76E-01	-1.46
ECSIT	ECSIT signalling integrator	1.00E+00	0	1.00E+00	0	1.41E-02	-2.365	1.77E-02	-2.634
ELAVL2	ELAV like neuron-specific RNA binding protein 2					5.31E-03	-2.148	6.25E-01	-1.118
ENO1	enolase 1, (alpha)	3.39E-01	1.109	1.62E-01	1.484	6.46E-03	-2.532	8.54E-03	-2.414
FHOD1	formin homology 2 domain containing 1	2.01E-01	-2.55	1.00E+00	-1.756	7.70E-03	-2.15	1.38E-02	-2.1
FUBP3	far upstream element (FUSE) binding protein 3	8.49E-01	-1.062	6.42E-01	1.068	1.17E-02	-1.238	1.72E-02	-1.155
GPX1	glutathione peroxidase 1	5.95E-01	1.428	6.56E-01	1.449	1.46E-02	1.273	1.51E-01	1.193
GTF2H3	general transcription factor IIH, polypeptide 3, 34kDa					1.85E-02	-2.117	3.93E-01	-1.393
GTF2I	general transcription factor IIi	2.48E-01	-1.295	5.24E-01	-1.528	5.99E-03	-1.282	1.35E-02	-1.186
HDAC2	histone deacetylase 2	1.00E+00	-3.841	1.00E+00	-1.241	4.63E-02	-1.22	4.45E-01	-1.123
HELZ2	helicase with zinc finger 2, transcriptional coactivator					6.15E-04	2.306	4.72E-02	1.675
HSPA8	heat shock 70kDa protein 8	7.91E-01	1.055	3.99E-01	1.176	4.62E-02	-2.934	5.98E-01	2.678
IFI16	interferon, gamma-inducible protein 16	9.19E-01	-1.03	3.01E-01	-1.441	4.97E-02	1.281	9.11E-01	1.012
IL18	interleukin 18	3.74E-01	-1.508	6.32E-01	-1.222	5.63E-03	1.981	1.43E-03	1.372
IL1RN	interleukin 1 receptor antagonist	1.00E+00	0	1.00E+00	0	4.59E-02	5.796	2.60E-01	3.522
IQGAP3	IQ motif containing GTPase activating protein 3	1.00E+00	0	1.00E+00	0	8.66E-03	2.33	3.17E-01	-1.12
KEAP1	kelch-like ECH-associated protein 1	1.00E-01	1.388	1.00E+00	0	4.76E-02	1.31	1.49E-01	-1.211
KLF13	Kruppel-like factor 13					2.56E-02	-2.008	1.00E+00	-2.097
MAFF	v-maf avian musculoaponeurotic fibrosarcoma oncogene homolog F	1.00E+00	0	1.00E+00	0	6.53E-04	2.808	2.29E-01	1.384
MED21	mediator complex subunit 21	1.00E+00	0	1.00E+00	0	1.37E-02	1.604	3.51E-01	1.245
MET	MET proto-oncogene, receptor tyrosine kinase	1.00E+00	0	1.00E+00	0	4.99E-02	-1.277	6.91E-01	1.046
MTERF3	mitochondrial transcription termination factor 3	1.00E+00	0	1.00E+00	0	1.35E-03	-4.077	1.33E-02	-6.005
MYBBP1A	MYB binding protein (P160) 1a	1.16E-02	-1.524	5.32E-01	-1.07	3.38E-02	-1.329	5.27E-02	-1.161

MYO6	myosin VI	7.07E-01	1.104	2.96E-01	-1.384	2.53E-02	1.468	7.71E-02	-1.689
NCK1	NCK adaptor protein 1	1.00E+00	1.371	1.00E+00	1.688	3.97E-03	1.85	3.49E-01	1.681
NOLC1	nucleolar and coiled-body phosphoprotein 1	5.80E-01	1.785	8.25E-01	1.283	4.09E-06	-1.731	3.65E-02	-8.09
PARP1	poly (ADP-ribose) polymerase 1	2.31E-01	-1.364	4.50E-01	-1.262	2.46E-03	-1.234	3.58E-01	1.134
PAWR	PRKC, apoptosis, WT1, regulator	1.00E+00	1.098	1.00E+00	0	7.64E-04	2.685	1.95E-01	1.444
PHB2	prohibitin 2	6.92E-01	-1.156	4.83E-01	1.24	3.05E-02	-1.193	3.36E-01	1.051
PKD1	polycystic kidney disease 1 (autosomal dominant)					3.21E-02	1.598	1.83E-02	1.797
PPID	peptidylprolyl isomerase D	1.43E-01	-1.434	1.18E-01	-1.53	3.17E-02	-1.441	6.87E-01	-1.041
PPM1A	protein phosphatase, Mg2+/Mn2+ dependent, 1A	1.00E+00	3.041	1.00E+00	0	1.54E-02	-1.391	3.32E-01	-1.153
PRMT7	protein arginine methyltransferase 7	1.00E+00	0	1.00E+00	0	4.76E-02	1.493	3.99E-02	1.951
RBBP7	retinoblastoma binding protein 7	9.33E-01	1.031	7.44E-01	1.077	1.38E-02	-3.281	6.28E-03	-6.779
RPS6KA4	ribosomal protein S6 kinase, 90kDa, polypeptide 4	1.00E+00	-1.026	1.00E+00	-1.418	2.13E-02	-1.624	1.13E-01	-1.257
SAP18	Sin3A-associated protein, 18kDa	3.87E-01	-1.837	1.00E+00	-1.141	1.58E-02	-1.203	2.11E-01	1.25
SMARCB1	SWI/SNF related, matrix associated, actin dependent regulator of chrom	1.00E+00	0	1.00E+00	0	1.38E-02	-1.689	1.69E-01	-1.22
STRAP	serine/threonine kinase receptor associated protein	4.66E-01	-1.445	7.06E-01	-1.186	2.65E-02	-1.333	6.87E-03	-1.382
TBK1	TANK-binding kinase 1	1.00E+00	1.058	1.00E+00	0	4.25E-02	-1.305	3.19E-01	-1.093
TCEB1	transcription elongation factor B (SIII), polypeptide 1 (15kDa, elongin C)	6.52E-01	1.202	7.71E-01	1.235	4.49E-02	-1.414	8.04E-01	-1.028
TCEB3	transcription elongation factor B (SIII), polypeptide 3 (110kDa, elongin A)	8.17E-01	1.238	1.00E+00	1.033	1.71E-02	-2.843	5.92E-01	-1.37
TCF12	transcription factor 12					1.43E-02	-1.871	1.00E+00	-2.456
TLR3	toll-like receptor 3					4.57E-02	-1.3	4.35E-02	-2.468
TRIM27	tripartite motif containing 27	1.00E+00	0	1.00E+00	0	2.37E-02	1.677	3.69E-01	1.23
TROVE2	TROVE domain family, member 2	3.20E-01	-1.312	3.38E-01	1.404	3.41E-02	1.343	7.75E-02	1.264
UBE2I	ubiquitin-conjugating enzyme E2I	1.38E-01	-1.801	8.79E-02	-1.654	1.19E-02	-1.485	6.95E-02	-1.283
UBE2L3	ubiquitin-conjugating enzyme E2L 3	9.58E-01	-1.053	9.55E-01	-1.079	1.42E-02	4.031	2.62E-01	1.81
VIM	vimentin	9.23E-01	-1.058	7.32E-01	-1.226	9.94E-04	-1.74	2.98E-02	-1.433
XAB2	XPA binding protein 2	1.00E+00	1.029	1.00E+00	1.851	2.07E-02	-1.513	2.66E-01	-1.533
YBX3	Y box binding protein 3	6.81E-01	1.32	4.39E-01	-1.621	3.77E-02	-1.3	1.55E-02	-1.41
ZNF143	zinc finger protein 143	1.00E+00	0	1.00E+00	0	1.00E-02	-1.233	4.06E-01	1.085

	LMP2-with Inhibitors: Expression of DNA (P = 0.00041)	LMP1 no Inhs		LMP2 no Inhs		LMP1 with Inhs		LMP2 with Inhs	
**Symbol**	**Entrez Gene Name**	**p-value**	**Fold Change**	**p-value**	**Fold Change**	**p-value**	**Fold Change**	**p-value**	**Fold Change**
ABLIM3	actin binding LIM protein family, member 3	1.00E+00	-1.711	1.00E+00	0	8.58E-01	-1.06	2.38E-02	-2.771
ABT1	activator of basal transcription 1	1.00E+00	0	1.00E+00	0	4.46E-02	-1.639	1.10E-02	-2.15
ASCC1	activating signal cointegrator 1 complex subunit 1	1.00E+00	0	1.00E+00	0	1.38E-01	-1.522	3.15E-02	-1.873
ATF1	activating transcription factor 1					5.54E-01	1.108	3.67E-02	1.33
ATRX	alpha thalassemia/mental retardation syndrome X-linked	1.00E+00	1.154	1.00E+00	0	1.46E-01	-1.223	2.48E-02	-1.745
ATXN7L3	ataxin 7-like 3					5.71E-03	-1.231	4.19E-02	1.368
CAPN15	calpain 15	1.00E+00	0	1.00E+00	0	9.67E-02	2.017	2.23E-02	2.423
CBX4	chromobox homolog 4	1.00E+00	0	1.00E+00	0	5.50E-01	-1.056	4.31E-02	1.425
CDKN1C	cyclin-dependent kinase inhibitor 1C (p57, Kip2)	1.00E+00	0	1.00E+00	0	2.48E-01	1.536	4.13E-03	-1.767
CEBPD	CCAAT/enhancer binding protein (C/EBP), delta	9.02E-02	-1.547	2.94E-01	-1.458	6.40E-01	1.213	3.42E-02	1.828
CHD1	chromodomain helicase DNA binding protein 1	1.00E+00	0	1.00E+00	0	1.19E-02	-3.257	2.45E-02	-2.212
CHD3	chromodomain helicase DNA binding protein 3	1.00E+00	0	1.00E+00	0	9.24E-02	-1.173	8.40E-03	1.216
CHD4	chromodomain helicase DNA binding protein 4	7.52E-01	-1.213	7.10E-01	1.11	9.81E-02	-1.276	4.86E-02	-1.42
DAB2IP	DAB2 interacting protein	1.00E+00	0	1.00E+00	0	1.99E-01	1.401	3.60E-02	-1.565
ECSIT	ECSIT signalling integrator	1.00E+00	0	1.00E+00	0	1.41E-02	-2.365	1.77E-02	-2.634
ENO1	enolase 1, (alpha)	3.39E-01	1.109	1.62E-01	1.484	6.46E-03	-2.532	8.54E-03	-2.414
ERCC2	excision repair cross-complementation group 2	1.00E+00	0	1.00E+00	0	9.09E-01	1.06	4.97E-02	1.761
FAS	Fas cell surface death receptor	1.00E+00	-1.102	1.00E+00	0	1.24E-01	-1.23	3.08E-03	-1.72

FHOD1	formin homology 2 domain containing 1	2.01E-01	-2.55	1.00E+00	-1.756	7.70E-03	-2.15	1.38E-02	-2.1
FUBP3	far upstream element (FUSE) binding protein 3	8.49E-01	-1.062	6.42E-01	1.068	1.17E-02	-1.238	1.72E-02	-1.155
GTF2I	general transcription factor IIi	2.48E-01	-1.295	5.24E-01	-1.528	5.99E-03	-1.282	1.35E-02	-1.186
GTF3C1	general transcription factor IIIC, polypeptide 1, alpha 220kDa	1.00E+00	-2.402	1.00E+00	-1.807	1.83E-01	-1.864	4.22E-02	-2.687
GTF3C2	general transcription factor IIIC, polypeptide 2, beta 110kDa	1.00E+00	-1.152	1.00E+00	1.455	7.51E-02	-1.646	1.13E-02	-1.855
HELZ2	helicase with zinc finger 2, transcriptional coactivator					6.15E-04	2.306	4.72E-02	1.675
IGBP1	immunoglobulin (CD79A) binding protein 1	8.70E-01	-1.056	1.00E+00	-1.076	2.00E-01	-1.267	7.97E-03	-1.947
IL18	interleukin 18	3.74E-01	-1.508	6.32E-01	-1.222	5.63E-03	1.981	1.43E-03	1.372
IRF2BP1	interferon regulatory factor 2 binding protein 1	7.55E-01	-1.075	1.00E+00	0	2.06E-01	-1.286	4.85E-02	-2.121
KANK2	KN motif and ankyrin repeat domains 2	1.00E+00	0	1.00E+00	0	8.80E-02	-1.928	6.18E-03	-2.708
KCTD1	potassium channel tetramerization domain containing 1	1.00E+00	0	1.00E+00	0	5.53E-02	1.501	4.47E-02	1.634
MBD1	methyl-CpG binding domain protein 1	1.00E+00	0	1.00E+00	0	3.34E-01	-1.117	2.46E-03	-1.734
MCM7	minichromosome maintenance complex component 7	2.36E-01	-1.658	1.82E-01	-1.311	1.73E-01	-1.253	4.74E-02	-1.355
MMS19	MMS19 nucleotide excision repair homolog (S. cerevisiae)	1.00E+00	0	1.00E+00	0	4.19E-01	1.154	1.89E-02	1.478
MTERF3	mitochondrial transcription termination factor 3	1.00E+00	0	1.00E+00	0	1.35E-03	-4.077	1.33E-02	-6.005
MYCBP	MYC binding protein	1.00E+00	-1.266	1.00E+00	-1.433	1.96E-01	1.464	4.50E-02	-1.363
NFKB1	nuclear factor of kappa light polypeptide gene enhancer in B-cells 1	7.54E-01	-1.28	8.35E-01	1.177	9.42E-02	1.205	1.63E-02	1.226
NKAP	NFKB activating protein	1.00E+00	0	1.00E+00	0	5.51E-01	1.185	3.79E-02	1.518
NOLC1	nucleolar and coiled-body phosphoprotein 1	5.80E-01	1.785	8.25E-01	1.283	4.09E-06	-1.731	3.65E-02	-8.09
PIAS3	protein inhibitor of activated STAT, 3					9.02E-02	4.761	1.35E-02	6.194
PKD1	polycystic kidney disease 1 (autosomal dominant)					3.21E-02	1.598	1.83E-02	1.797
PML	promyelocytic leukemia	4.01E-01	-1.174	2.33E-02	-2.367	7.11E-01	1.114	4.66E-02	-1.449
PPP1R13L	protein phosphatase 1, regulatory subunit 13 like	4.18E-01	1.766	3.18E-01	-1.383	3.89E-01	1.17	4.07E-02	-1.352
PRMT7	protein arginine methyltransferase 7	1.00E+00	0	1.00E+00	0	4.76E-02	1.493	3.99E-02	1.951
PSIP1	PC4 and SFRS1 interacting protein 1	4.98E-01	-1.186	1.00E+00	1.241	1.30E-01	-1.136	4.78E-03	-1.159
RBBP7	retinoblastoma binding protein 7	9.33E-01	1.031	7.44E-01	1.077	1.38E-02	-3.281	6.28E-03	-6.779
RIPK1	receptor (TNFRSF)-interacting serine-threonine kinase 1	1.00E+00	0	1.00E+00	0	3.61E-01	1.444	3.39E-03	-1.61
SIX4	SIX homeobox 4	1.00E+00	0	1.00E+00	0	5.98E-02	1.77	4.28E-02	2.119
SMAD2	SMAD family member 2	9.07E-01	-1.022	1.00E+00	-1.036	3.94E-01	1.118	3.74E-02	-1.304
STRAP	serine/threonine kinase receptor associated protein	4.66E-01	-1.445	7.06E-01	-1.186	2.65E-02	-1.333	6.87E-03	-1.382
TAF4	TAF4 RNA polymerase II, TATA box binding protein (TBP)-associated fa	1.00E+00	0	1.00E+00	0	8.06E-01	-1.053	4.00E-02	-1.449
TAF9	TAF9 RNA polymerase II, TATA box binding protein (TBP)-associated fa			1.00E+00	1.159	6.24E-01	-1.18	4.79E-03	-1.507
TLR3	toll-like receptor 3					4.57E-02	-1.3	4.35E-02	-2.468
TRAF6	TNF receptor-associated factor 6, E3 ubiquitin protein ligase	1.00E+00	0	1.00E+00	0	1.29E-01	-1.539	5.71E-03	-1.943
TSC22D1	TSC22 domain family, member 1	1.00E+00	0	1.00E+00	0	1.00E+00	-3.302	2.90E-02	-3.96
VEZF1	vascular endothelial zinc finger 1					6.06E-02	-2.015	7.20E-03	-1.926
VIM	vimentin	9.23E-01	-1.058	7.32E-01	-1.226	9.94E-04	-1.74	2.98E-02	-1.433
YBX3	Y box binding protein 3	6.81E-01	1.32	4.39E-01	-1.621	3.77E-02	-1.3	1.55E-02	-1.41
ZNF593	zinc finger protein 593	1.00E+00	0	1.00E+00	0	2.07E-01	-1.615	8.80E-03	-1.441

	LMP1 no Inhibitors: Transcription of RNA (P = 1)	LMP1 no Inhs		LMP2 no Inhs		LMP1 with Inhs		LMP2 with Inhs	
none									

	LMP2 no Inhibitors: Transcription of RNA (P = 1)	LMP1 no Inhs		LMP2 no Inhs		LMP1 with Inhs		LMP2 with Inhs	
none									

	LMP1 with Inhibitors: Transcription of RNA (P = 0.0031)	LMP1 no Inhs		LMP2 no Inhs		LMP1 with Inhs		LMP2 with Inhs	
Symbol	Entrez Gene Name	p-value	Fold Change	p-value	Fold Change	p-value	Fold Change	p-value	Fold Change
AATF	apoptosis antagonizing transcription factor	3.12E-01	-1.37	4.76E-01	1.207	3.10E-02	-1.21	4.32E-01	-1.074
ABT1	activator of basal transcription 1	1.00E+00	0	1.00E+00	0	4.46E-02	-1.639	1.10E-02	-2.15
ACTR1B	ARP1 actin-related protein 1 homolog B, centractin beta (yeast)	9.35E-01	-1.051	1.00E+00	1.386	2.25E-02	1.414	7.61E-01	1.077
ARHGEF11	Rho guanine nucleotide exchange factor (GEF) 11	1.00E+00	0	1.00E+00	0	1.83E-02	1.612	6.07E-01	-1.132
ATF7	activating transcription factor 7	1.00E+00	0	1.00E+00	0	4.54E-02	-2.237	3.51E-01	-1.311
ATF7IP	activating transcription factor 7 interacting protein	1.00E+00	0	1.00E+00	0	2.41E-02	2.025	4.06E-01	-1.148
ATP2C1	ATPase, Ca++ transporting, type 2C, member 1	1.00E+00	0	1.00E+00	0	4.62E-02	2.343	6.24E-01	1.139
ATXN7L3	ataxin 7-like 3					5.71E-03	-1.231	4.19E-02	1.368
BAG1	BCL2-associated athanogene	1.00E+00	0	1.00E+00	0	2.96E-03	2.613	9.49E-01	1.028
BCLAF1	BCL2-associated transcription factor 1	2.45E-01	1.432	8.27E-01	-1.07	1.38E-02	-1.654	8.10E-01	1.037
BPTF	bromodomain PHD finger transcription factor	1.00E+00	0	1.00E+00	0	3.55E-02	-2.04	1.83E-01	-1.528
BRD8	bromodomain containing 8	1.00E+00	-2.574	1.00E+00	1.393	2.52E-02	1.56	3.21E-01	1.296
CASP1	caspase 1, apoptosis-related cysteine peptidase	1.00E+00	1.079	1.57E-01	-1.776	3.91E-02	1.376	4.70E-01	-1.299
CBX1	chromobox homolog 1	1.00E+00	-2.276	1.00E+00	1.612	2.66E-02	-1.502	2.12E-01	-1.267
CCNT1	cyclin T1	1.00E+00	0	1.00E+00	0	3.29E-02	-2.021	2.75E-01	-1.373
CD44	CD44 molecule (Indian blood group)	4.40E-01	-2.277	1.34E-01	-3.127	5.16E-03	-1.316	4.93E-01	-1.089
CHD1	chromodomain helicase DNA binding protein 1	1.00E+00	0	1.00E+00	0	1.19E-02	-3.257	2.45E-02	-2.212
CHD2	chromodomain helicase DNA binding protein 2	1.00E+00	1.064	1.00E+00	0	2.19E-02	-1.743	3.30E-01	-1.109
CRABP2	cellular retinoic acid binding protein 2	1.00E+00	0	1.00E+00	0	2.01E-02	10.196	5.80E-01	1.753
CTBP1	C-terminal binding protein 1	5.49E-01	-1.175	5.46E-01	1.181	3.58E-02	-1.525	2.73E-01	-1.224
CXXC1	CXXC finger protein 1	1.00E+00	0	1.00E+00	0	1.74E-02	1.379	1.37E-01	1.277
DDX58	DEAD (Asp-Glu-Ala-Asp) box polypeptide 58					2.50E-02	4.906	9.74E-01	-1.016
DMAP1	DNA methyltransferase 1 associated protein 1	1.00E+00	0	1.00E+00	0	3.08E-02	-2.365	1.76E-01	-1.46
ECSIT	ECSIT signalling integrator	1.00E+00	0	1.00E+00	0	1.41E-02	-2.365	1.77E-02	-2.634
EIF2S1	eukaryotic translation initiation factor 2, subunit 1 alpha, 35kDa	1.87E-01	1.365	7.04E-01	1.067	2.93E-03	-1.354	7.24E-01	-1.028
ELAVL2	ELAV like neuron-specific RNA binding protein 2					5.31E-03	-2.148	6.25E-01	-1.118
ENO1	enolase 1, (alpha)	3.39E-01	1.109	1.62E-01	1.484	6.46E-03	-2.532	8.54E-03	-2.414
FHOD1	formin homology 2 domain containing 1	2.01E-01	-2.55	1.00E+00	-1.756	7.70E-03	-2.15	1.38E-02	-2.1
FUBP3	far upstream element (FUSE) binding protein 3	8.49E-01	-1.062	6.42E-01	1.068	1.17E-02	-1.238	1.72E-02	-1.155
GBP2	guanylate binding protein 2, interferon-inducible	1.00E+00	0	1.00E+00	0	1.36E-02	2.228	1.29E-01	-1.931
GRN	granulin					7.46E-04	1.284	2.37E-01	1.124
GTF2H3	general transcription factor IIH, polypeptide 3, 34kDa					1.85E-02	-2.117	3.93E-01	-1.393
GTF2I	general transcription factor IIi	2.48E-01	-1.295	5.24E-01	-1.528	5.99E-03	-1.282	1.35E-02	-1.186
H1F0	H1 histone family, member 0	1.00E+00	0	1.00E+00	0	4.62E-04	3.114	5.34E-01	1.213
HDAC2	histone deacetylase 2	1.00E+00	-3.841	1.00E+00	-1.241	4.63E-02	-1.22	4.45E-01	-1.123
HELZ2	helicase with zinc finger 2, transcriptional coactivator					6.15E-04	2.306	4.72E-02	1.675
HSPA8	heat shock 70kDa protein 8	7.91E-01	1.055	3.99E-01	1.176	4.62E-02	-2.934	5.98E-01	2.678
IFI16	interferon, gamma-inducible protein 16	9.19E-01	-1.03	3.01E-01	-1.441	4.97E-02	1.281	9.11E-01	1.012
IKBKG	inhibitor of kappa light polypeptide gene enhancer in B-cells, kinase gamma	1.00E+00	1.673	1.00E+00	1.769	5.03E-03	1.742	2.19E-01	1.327
IL18	interleukin 18	3.74E-01	-1.508	6.32E-01	-1.222	5.63E-03	1.981	1.43E-03	1.372
KEAP1	kelch-like ECH-associated protein 1	1.00E-01	1.388	1.00E+00	0	4.76E-02	1.31	1.49E-01	-1.211

KLF13	Kruppel-like factor 13					2.56E-02	-2.008	1.00E+00	-2.097
MAFF	v-maf avian musculoaponeurotic fibrosarcoma oncogene homolog F	1.00E+00	0	1.00E+00	0	6.53E-04	2.808	2.29E-01	1.384
MED21	mediator complex subunit 21	1.00E+00	0	1.00E+00	0	1.37E-02	1.604	3.51E-01	1.245
MET	MET proto-oncogene, receptor tyrosine kinase	1.00E+00	0	1.00E+00	0	4.99E-02	-1.277	6.91E-01	1.046
MTERF3	mitochondrial transcription termination factor 3	1.00E+00	0	1.00E+00	0	1.35E-03	-4.077	1.33E-02	-6.005
MYBBP1A	MYB binding protein (P160) 1a	1.16E-02	-1.524	5.32E-01	-1.07	3.38E-02	-1.329	5.27E-02	-1.161
MYO6	myosin VI	7.07E-01	1.104	2.96E-01	-1.384	2.53E-02	1.468	7.71E-02	-1.689
NCK1	NCK adaptor protein 1	1.00E+00	1.371	1.00E+00	1.688	3.97E-03	1.85	3.49E-01	1.681
NOLC1	nucleolar and coiled-body phosphoprotein 1	5.80E-01	1.785	8.25E-01	1.283	4.09E-06	-1.731	3.65E-02	-8.09
PARP1	poly (ADP-ribose) polymerase 1	2.31E-01	-1.364	4.50E-01	-1.262	2.46E-03	-1.234	3.58E-01	1.134
PAWR	PRKC, apoptosis, WT1, regulator	1.00E+00	1.098	1.00E+00	0	7.64E-04	2.685	1.95E-01	1.444
PHB2	prohibitin 2	6.92E-01	-1.156	4.83E-01	1.24	3.05E-02	-1.193	3.36E-01	1.051
PHGDH	phosphoglycerate dehydrogenase	6.87E-01	-1.119	3.93E-01	1.376	1.42E-02	-1.542	6.59E-04	1.472
PKD1	polycystic kidney disease 1 (autosomal dominant)					3.21E-02	1.598	1.83E-02	1.797
PPID	peptidylprolyl isomerase D	1.43E-01	-1.434	1.18E-01	-1.53	3.17E-02	-1.441	6.87E-01	-1.041
PPM1A	protein phosphatase, Mg2+/Mn2+ dependent, 1A	1.00E+00	3.041	1.00E+00	0	1.54E-02	-1.391	3.32E-01	-1.153
PPP1CC	protein phosphatase 1, catalytic subunit, gamma isozyme	1.00E+00	0	1.00E+00	-3.795	3.37E-02	1.428	2.43E-03	1.45
PSMB10	proteasome (prosome, macropain) subunit, beta type, 10	1.00E+00	0	1.00E+00	0	1.03E-02	2.319	1.00E+00	-1.566
PSMD14	proteasome (prosome, macropain) 26S subunit, non-ATPase, 14	8.70E-01	-1.028	9.65E-01	1.043	4.95E-02	-1.39	4.85E-01	-1.234
RB1CC1	RB1-inducible coiled-coil 1	1.00E+00	0	1.00E+00	0	3.58E-02	1.808	5.38E-02	1.256
RBBP7	retinoblastoma binding protein 7	9.33E-01	1.031	7.44E-01	1.077	1.38E-02	-3.281	6.28E-03	-6.779
RBX1	ring-box 1, E3 ubiquitin protein ligase	1.00E+00	0	1.00E+00	0	2.30E-02	-1.494	7.36E-01	-1.081
RPS6KA4	ribosomal protein S6 kinase, 90kDa, polypeptide 4	1.00E+00	-1.026	1.00E+00	-1.418	2.13E-02	-1.624	1.13E-01	-1.257
SAP18	Sin3A-associated protein, 18kDa	3.87E-01	-1.837	1.00E+00	-1.141	1.58E-02	-1.203	2.11E-01	1.25
SMARCB1	SWI/SNF related, matrix associated, actin dependent regulator of chromatin, subfa	1.00E+00	0	1.00E+00	0	1.38E-02	-1.689	1.69E-01	-1.22
STRAP	serine/threonine kinase receptor associated protein	4.66E-01	-1.445	7.06E-01	-1.186	2.65E-02	-1.333	6.87E-03	-1.382
TBK1	TANK-binding kinase 1	1.00E+00	1.058	1.00E+00	0	4.25E-02	-1.305	3.19E-01	-1.093
TCEB1	transcription elongation factor B (SIII), polypeptide 1 (15kDa, elongin C)	6.52E-01	1.202	7.71E-01	1.235	4.49E-02	-1.414	8.04E-01	-1.028
TCEB3	transcription elongation factor B (SIII), polypeptide 3 (110kDa, elongin A)	8.17E-01	1.238	1.00E+00	1.033	1.71E-02	-2.843	5.92E-01	-1.37
TCF12	transcription factor 12					1.43E-02	-1.871	1.00E+00	-2.456
TFG	TRK-fused gene	3.41E-01	-1.314	1.00E+00	-1.723	5.74E-03	2.478	1.40E-01	1.537
TIMP1	TIMP metallopeptidase inhibitor 1	8.12E-01	1.079	1.00E+00	-1.174	1.98E-02	1.741	9.21E-01	1.008
TIMP3	TIMP metallopeptidase inhibitor 3	1.00E+00	-2.697	1.00E+00	0	4.13E-03	-1.852	4.38E-01	-1.628
TLR3	toll-like receptor 3					4.57E-02	-1.3	4.35E-02	-2.468
TOM1L1	target of myb1 (chicken)-like 1	1.00E+00	2.504	1.00E+00	0	2.89E-02	-1.651	7.87E-02	-1.487
TRIM27	tripartite motif containing 27	1.00E+00	0	1.00E+00	0	2.37E-02	1.677	3.69E-01	1.23
TROVE2	TROVE domain family, member 2	3.20E-01	-1.312	3.38E-01	1.404	3.41E-02	1.343	7.75E-02	1.264
TUBG1	tubulin, gamma 1	9.19E-01	-1.036	8.47E-01	1.068	4.40E-02	-1.277	5.92E-02	-1.25
UBE2I	ubiquitin-conjugating enzyme E2I	1.38E-01	-1.801	8.79E-02	-1.654	1.19E-02	-1.485	6.95E-02	-1.283
UBE2L3	ubiquitin-conjugating enzyme E2L 3	9.58E-01	-1.053	9.55E-01	-1.079	1.42E-02	4.031	2.62E-01	1.81
XAB2	XPA binding protein 2	1.00E+00	1.029	1.00E+00	1.851	2.07E-02	-1.513	2.66E-01	-1.533
YBX3	Y box binding protein 3	6.81E-01	1.32	4.39E-01	-1.621	3.77E-02	-1.3	1.55E-02	-1.41
ZNF143	zinc finger protein 143	1.00E+00	0	1.00E+00	0	1.00E-02	-1.233	4.06E-01	1.085

	**LMP2 with Inhibitors: Transcription of RNA (P = 0.00055)**	**LMP1 no Inhs**		**LMP2 no Inhs**		**LMP1 with Inhs**		**LMP2 with Inhs**	
**Symbol**	**Entrez Gene Name**	**p-value**	**Fold Change**	**p-value**	**Fold Change**	**p-value**	**Fold Change**	**p-value**	**Fold Change**
ABLIM3	actin binding LIM protein family, member 3	1.00E+00	-1.711	1.00E+00	0	8.58E-01	-1.06	2.38E-02	-2.771
ABT1	activator of basal transcription 1	1.00E+00	0	1.00E+00	0	4.46E-02	-1.639	1.10E-02	-2.15
ACTR2	ARP2 actin-related protein 2 homolog (yeast)	3.55E-01	1.594	9.05E-01	-1.028	8.73E-01	-1.01	4.13E-02	-1.124

ASCC1	activating signal cointegrator 1 complex subunit 1	1.00E+00	0	1.00E+00	0	1.38E-01	-1.522	3.15E-02	-1.873
ATF1	activating transcription factor 1					5.54E-01	1.108	3.67E-02	1.33
ATRX	alpha thalassemia/mental retardation syndrome X-linked	1.00E+00	1.154	1.00E+00	0	1.46E-01	-1.223	2.48E-02	-1.745
ATXN7L3	ataxin 7-like 3					5.71E-03	-1.231	4.19E-02	1.368
BAZ2A	bromodomain adjacent to zinc finger domain, 2A					4.48E-01	1.266	2.16E-02	-1.528
CAPN15	calpain 15	1.00E+00	0	1.00E+00	0	9.67E-02	2.017	2.23E-02	2.423
CBX4	chromobox homolog 4	1.00E+00	0	1.00E+00	0	5.50E-01	-1.056	4.31E-02	1.425
CDKN1C	cyclin-dependent kinase inhibitor 1C (p57, Kip2)	1.00E+00	0	1.00E+00	0	2.48E-01	1.536	4.13E-03	-1.767
CEBPD	CCAAT/enhancer binding protein (C/EBP), delta	9.02E-02	-1.547	2.94E-01	-1.458	6.40E-01	1.213	3.42E-02	1.828
CHD1	chromodomain helicase DNA binding protein 1	1.00E+00	0	1.00E+00	0	1.19E-02	-3.257	2.45E-02	-2.212
CHD3	chromodomain helicase DNA binding protein 3	1.00E+00	0	1.00E+00	0	9.24E-02	-1.173	8.40E-03	1.216
CHD4	chromodomain helicase DNA binding protein 4	7.52E-01	-1.213	7.10E-01	1.11	9.81E-02	-1.276	4.86E-02	-1.42
DAB2IP	DAB2 interacting protein	1.00E+00	0	1.00E+00	0	1.99E-01	1.401	3.60E-02	-1.565
DYRK1A	dual-specificity tyrosine-(Y)-phosphorylation regulated kinase 1A					7.80E-02	-1.271	3.54E-02	-1.395
ECSIT	ECSIT signalling integrator	1.00E+00	0	1.00E+00	0	1.41E-02	-2.365	1.77E-02	-2.634
EEF1D	eukaryotic translation elongation factor 1 delta (guanine nucleotide exchange protei	6.47E-01	1.485	5.90E-01	1.601	1.17E-01	-1.375	8.67E-03	3.326
ENO1	enolase 1, (alpha)	3.39E-01	1.109	1.62E-01	1.484	6.46E-03	-2.532	8.54E-03	-2.414
ERCC2	excision repair cross-complementation group 2	1.00E+00	0	1.00E+00	0	9.09E-01	1.06	4.97E-02	1.761
FHOD1	formin homology 2 domain containing 1	2.01E-01	-2.55	1.00E+00	-1.756	7.70E-03	-2.15	1.38E-02	-2.1
FUBP3	far upstream element (FUSE) binding protein 3	8.49E-01	-1.062	6.42E-01	1.068	1.17E-02	-1.238	1.72E-02	-1.155
GTF2I	general transcription factor IIi	2.48E-01	-1.295	5.24E-01	-1.528	5.99E-03	-1.282	1.35E-02	-1.186
GTF3C1	general transcription factor IIIC, polypeptide 1, alpha 220kDa	1.00E+00	-2.402	1.00E+00	-1.807	1.83E-01	-1.864	4.22E-02	-2.687
GTF3C2	general transcription factor IIIC, polypeptide 2, beta 110kDa	1.00E+00	-1.152	1.00E+00	1.455	7.51E-02	-1.646	1.13E-02	-1.855
H2AFX	H2A histone family, member X	8.16E-01	-1.078	9.95E-01	1.009	8.35E-01	1.046	4.72E-02	1.489
HELZ2	helicase with zinc finger 2, transcriptional coactivator					6.15E-04	2.306	4.72E-02	1.675
HNRNPA2B1	heterogeneous nuclear ribonucleoprotein A2/B1	3.72E-01	-1.186	9.16E-01	1.021	7.09E-02	-1.33	2.14E-03	2.533
IGBP1	immunoglobulin (CD79A) binding protein 1	8.70E-01	-1.056	1.00E+00	-1.076	2.00E-01	-1.267	7.97E-03	-1.947
IL18	interleukin 18	3.74E-01	-1.508	6.32E-01	-1.222	5.63E-03	1.981	1.43E-03	1.372
IRF2BP1	interferon regulatory factor 2 binding protein 1	7.55E-01	-1.075	1.00E+00	0	2.06E-01	-1.286	4.85E-02	-2.121
KANK2	KN motif and ankyrin repeat domains 2	1.00E+00	0	1.00E+00	0	8.80E-02	-1.928	6.18E-03	-2.708
KCTD1	potassium channel tetramerization domain containing 1	1.00E+00	0	1.00E+00	0	5.53E-02	1.501	4.47E-02	1.634
MBD1	methyl-CpG binding domain protein 1	1.00E+00	0	1.00E+00	0	3.34E-01	-1.117	2.46E-03	-1.734
MCM7	minichromosome maintenance complex component 7	2.36E-01	-1.658	1.82E-01	-1.311	1.73E-01	-1.253	4.74E-02	-1.355
MMS19	MMS19 nucleotide excision repair homolog (S. cerevisiae)	1.00E+00	0	1.00E+00	0	4.19E-01	1.154	1.89E-02	1.478
MTERF3	mitochondrial transcription termination factor 3	1.00E+00	0	1.00E+00	0	1.35E-03	-4.077	1.33E-02	-6.005
MYCBP	MYC binding protein	1.00E+00	-1.266	1.00E+00	-1.433	1.96E-01	1.464	4.50E-02	-1.363
NFKB1	nuclear factor of kappa light polypeptide gene enhancer in B-cells 1	7.54E-01	-1.28	8.35E-01	1.177	9.42E-02	1.205	1.63E-02	1.226
NKAP	NFKB activating protein	1.00E+00	0	1.00E+00	0	5.51E-01	1.185	3.79E-02	1.518
NOLC1	nucleolar and coiled-body phosphoprotein 1	5.80E-01	1.785	8.25E-01	1.283	4.09E-06	-1.731	3.65E-02	-8.09
NPM1	nucleophosmin (nucleolar phosphoprotein B23, numatrin)	3.04E-01	-1.476	1.10E-01	-1.239	2.68E-01	-1.67	1.58E-02	1.215
OGT	O-linked N-acetylglucosamine (GlcNAc) transferase	4.88E-01	1.135	9.90E-01	1.007	9.40E-01	1.015	2.70E-02	-1.424
PHGDH	phosphoglycerate dehydrogenase	6.87E-01	-1.119	3.93E-01	1.376	1.42E-02	-1.542	6.59E-04	1.472
PIAS3	protein inhibitor of activated STAT, 3					9.02E-02	4.761	1.35E-02	6.194
PKD1	polycystic kidney disease 1 (autosomal dominant)					3.21E-02	1.598	1.83E-02	1.797
PML	promyelocytic leukemia	4.01E-01	-1.174	2.33E-02	-2.367	7.11E-01	1.114	4.66E-02	-1.449
PPP1CC	protein phosphatase 1, catalytic subunit, gamma isozyme	1.00E+00	0	1.00E+00	-3.795	3.37E-02	1.428	2.43E-03	1.45
PPP1R13L	protein phosphatase 1, regulatory subunit 13 like	4.18E-01	1.766	3.18E-01	-1.383	3.89E-01	1.17	4.07E-02	-1.352
PSIP1	PC4 and SFRS1 interacting protein 1	4.98E-01	-1.186	1.00E+00	1.241	1.30E-01	-1.136	4.78E-03	-1.159
RBBP7	retinoblastoma binding protein 7	9.33E-01	1.031	7.44E-01	1.077	1.38E-02	-3.281	6.28E-03	-6.779

RFX5	regulatory factor X, 5 (influences HLA class II expression)	1.00E+00	0	1.00E+00	0	7.07E-02	1.684	7.53E-03	1.675
RIPK1	receptor (TNFRSF)-interacting serine-threonine kinase 1	1.00E+00	0	1.00E+00	0	3.61E-01	1.444	3.39E-03	-1.61
RPS6KB1	ribosomal protein S6 kinase, 70kDa, polypeptide 1	1.00E+00	1.861	1.00E+00	1.983	1.04E-01	-1.675	3.62E-02	-1.878
SIX4	SIX homeobox 4	1.00E+00	0	1.00E+00	0	5.98E-02	1.77	4.28E-02	2.119
SMAD2	SMAD family member 2	9.07E-01	-1.022	1.00E+00	-1.036	3.94E-01	1.118	3.74E-02	-1.304
STRAP	serine/threonine kinase receptor associated protein	4.66E-01	-1.445	7.06E-01	-1.186	2.65E-02	-1.333	6.87E-03	-1.382
TAF4	TAF4 RNA polymerase II, TATA box binding protein (TBP)-associated factor, 135kI	1.00E+00	0	1.00E+00	0	8.06E-01	-1.053	4.00E-02	-1.449
TAF9	TAF9 RNA polymerase II, TATA box binding protein (TBP)-associated factor, 32kD			1.00E+00	1.159	6.24E-01	-1.18	4.79E-03	-1.507
TLR3	toll-like receptor 3					4.57E-02	-1.3	4.35E-02	-2.468
TRAF6	TNF receptor-associated factor 6, E3 ubiquitin protein ligase	1.00E+00	0	1.00E+00	0	1.29E-01	-1.539	5.71E-03	-1.943
TSC22D1	TSC22 domain family, member 1	1.00E+00	0	1.00E+00	0	1.00E+00	-3.302	2.90E-02	-3.96
UNC45A	unc-45 homolog A (C. elegans)	3.92E-01	-1.183	6.63E-01	1.063	5.46E-02	-1.327	3.87E-02	-1.362
VEZF1	vascular endothelial zinc finger 1					6.06E-02	-2.015	7.20E-03	-1.926
YBX3	Y box binding protein 3	6.81E-01	1.32	4.39E-01	-1.621	3.77E-02	-1.3	1.55E-02	-1.41
ZNF593	zinc finger protein 593	1.00E+00	0	1.00E+00	0	2.07E-01	-1.615	8.80E-03	-1.441

	LMP1-no Inhibitors: Transcription of DNA (P = 1)	LMP1 no Inhs		LMP2 no Inhs		LMP1 with Inhs		LMP2 with Inhs	
None									

	LMP2-no Inhibitors: Transcription of DNA (P = 1)	LMP1 no Inhs		LMP2 no Inhs		LMP1 with Inhs		LMP2 with Inhs	
None									

	LMP1-with Inhibitors: Transcription of DNA (P = 1)	LMP1 no Inhs		LMP2 no Inhs		LMP1 with Inhs		LMP2 with Inhs	
None									

	LMP2-with Inhibitors: Transcription of DNA (P = 0.00173)	LMP1 no Inhs		LMP2 no Inhs		LMP1 with Inhs		LMP2 with Inhs	
**Symbol**	**Entrez Gene Name**	**p-value**	**Fold Change**	**p-value**	**Fold Change**	**p-value**	**Fold Change**	**p-value**	**Fold Change**
ABLIM3	actin binding LIM protein family, member 3	1.00E+00	-1.711	1.00E+00	0	8.58E-01	-1.06	2.38E-02	-2.771
ABT1	activator of basal transcription 1	1.00E+00	0	1.00E+00	0	4.46E-02	-1.639	1.10E-02	-2.15
ASCC1	activating signal cointegrator 1 complex subunit 1	1.00E+00	0	1.00E+00	0	1.38E-01	-1.522	3.15E-02	-1.873
ATF1	activating transcription factor 1					5.54E-01	1.108	3.67E-02	1.33
ATRX	alpha thalassemia/mental retardation syndrome X-linked	1.00E+00	1.154	1.00E+00	0	1.46E-01	-1.223	2.48E-02	-1.745
ATXN7L3	ataxin 7-like 3					5.71E-03	-1.231	4.19E-02	1.368
CAPN15	calpain 15	1.00E+00	0	1.00E+00	0	9.67E-02	2.017	2.23E-02	2.423
CBX4	chromobox homolog 4	1.00E+00	0	1.00E+00	0	5.50E-01	-1.056	4.31E-02	1.425
CDKN1C	cyclin-dependent kinase inhibitor 1C (p57, Kip2)	1.00E+00	0	1.00E+00	0	2.48E-01	1.536	4.13E-03	-1.767
CEBPD	CCAAT/enhancer binding protein (C/EBP), delta	9.02E-02	-1.547	2.94E-01	-1.458	6.40E-01	1.213	3.42E-02	1.828
CHD1	chromodomain helicase DNA binding protein 1	1.00E+00	0	1.00E+00	0	1.19E-02	-3.257	2.45E-02	-2.212
CHD3	chromodomain helicase DNA binding protein 3	1.00E+00	0	1.00E+00	0	9.24E-02	-1.173	8.40E-03	1.216
CHD4	chromodomain helicase DNA binding protein 4	7.52E-01	-1.213	7.10E-01	1.11	9.81E-02	-1.276	4.86E-02	-1.42
DAB2IP	DAB2 interacting protein	1.00E+00	0	1.00E+00	0	1.99E-01	1.401	3.60E-02	-1.565
ECSIT	ECSIT signalling integrator	1.00E+00	0	1.00E+00	0	1.41E-02	-2.365	1.77E-02	-2.634
ENO1	enolase 1, (alpha)	3.39E-01	1.109	1.62E-01	1.484	6.46E-03	-2.532	8.54E-03	-2.414
ERCC2	excision repair cross-complementation group 2	1.00E+00	0	1.00E+00	0	9.09E-01	1.06	4.97E-02	1.761
FHOD1	formin homology 2 domain containing 1	2.01E-01	-2.55	1.00E+00	-1.756	7.70E-03	-2.15	1.38E-02	-2.1
FUBP3	far upstream element (FUSE) binding protein 3	8.49E-01	-1.062	6.42E-01	1.068	1.17E-02	-1.238	1.72E-02	-1.155
GTF2I	general transcription factor IIi	2.48E-01	-1.295	5.24E-01	-1.528	5.99E-03	-1.282	1.35E-02	-1.186
GTF3C1	general transcription factor IIIC, polypeptide 1, alpha 220kDa	1.00E+00	-2.402	1.00E+00	-1.807	1.83E-01	-1.864	4.22E-02	-2.687
GTF3C2	general transcription factor IIIC, polypeptide 2, beta 110kDa	1.00E+00	-1.152	1.00E+00	1.455	7.51E-02	-1.646	1.13E-02	-1.855
HELZ2	helicase with zinc finger 2, transcriptional coactivator					6.15E-04	2.306	4.72E-02	1.675
IGBP1	immunoglobulin (CD79A) binding protein 1	8.70E-01	-1.056	1.00E+00	-1.076	2.00E-01	-1.267	7.97E-03	-1.947
IRF2BP1	interferon regulatory factor 2 binding protein 1	7.55E-01	-1.075	1.00E+00	0	2.06E-01	-1.286	4.85E-02	-2.121
KANK2	KN motif and ankyrin repeat domains 2	1.00E+00	0	1.00E+00	0	8.80E-02	-1.928	6.18E-03	-2.708
KCTD1	potassium channel tetramerization domain containing 1	1.00E+00	0	1.00E+00	0	5.53E-02	1.501	4.47E-02	1.634
MBD1	methyl-CpG binding domain protein 1	1.00E+00	0	1.00E+00	0	3.34E-01	-1.117	2.46E-03	-1.734
MCM7	minichromosome maintenance complex component 7	2.36E-01	-1.658	1.82E-01	-1.311	1.73E-01	-1.253	4.74E-02	-1.355
MMS19	MMS19 nucleotide excision repair homolog (S. cerevisiae)	1.00E+00	0	1.00E+00	0	4.19E-01	1.154	1.89E-02	1.478
MTERF3	mitochondrial transcription termination factor 3	1.00E+00	0	1.00E+00	0	1.35E-03	-4.077	1.33E-02	-6.005
MYCBP	MYC binding protein	1.00E+00	-1.266	1.00E+00	-1.433	1.96E-01	1.464	4.50E-02	-1.363
NFKB1	nuclear factor of kappa light polypeptide gene enhancer in B-cells 1	7.54E-01	-1.28	8.35E-01	1.177	9.42E-02	1.205	1.63E-02	1.226
NKAP	NFKB activating protein	1.00E+00	0	1.00E+00	0	5.51E-01	1.185	3.79E-02	1.518
NOLC1	nucleolar and coiled-body phosphoprotein 1	5.80E-01	1.785	8.25E-01	1.283	4.09E-06	-1.731	3.65E-02	-8.09
PKD1	polycystic kidney disease 1 (autosomal dominant)					3.21E-02	1.598	1.83E-02	1.797
PML	promyelocytic leukemia	4.01E-01	-1.174	2.33E-02	-2.367	7.11E-01	1.114	4.66E-02	-1.449
PPP1R13L	protein phosphatase 1, regulatory subunit 13 like	4.18E-01	1.766	3.18E-01	-1.383	3.89E-01	1.17	4.07E-02	-1.352
PSIP1	PC4 and SFRS1 interacting protein 1	4.98E-01	-1.186	1.00E+00	1.241	1.30E-01	-1.136	4.78E-03	-1.159

RBBP7	retinoblastoma binding protein 7	9.33E-01	1.031	7.44E-01	1.077	1.38E-02	-3.281	6.28E-03	-6.779
RIPK1	receptor (TNFRSF)-interacting serine-threonine kinase 1	1.00E+00	0	1.00E+00	0	3.61E-01	1.444	3.39E-03	-1.61
SIX4	SIX homeobox 4	1.00E+00	0	1.00E+00	0	5.98E-02	1.77	4.28E-02	2.119
SMAD2	SMAD family member 2	9.07E-01	-1.022	1.00E+00	-1.036	3.94E-01	1.118	3.74E-02	-1.304
STRAP	serine/threonine kinase receptor associated protein	4.66E-01	-1.445	7.06E-01	-1.186	2.65E-02	-1.333	6.87E-03	-1.382
TAF4	TAF4 RNA polymerase II, TATA box binding protein (TBP)-associated factor, 135kD	1.00E+00	0	1.00E+00	0	8.06E-01	-1.053	4.00E-02	-1.449
TAF9	TAF9 RNA polymerase II, TATA box binding protein (TBP)-associated factor, 32kDa			1.00E+00	1.159	6.24E-01	-1.18	4.79E-03	-1.507
TLR3	toll-like receptor 3					4.57E-02	-1.3	4.35E-02	-2.468
TRAF6	TNF receptor-associated factor 6, E3 ubiquitin protein ligase	1.00E+00	0	1.00E+00	0	1.29E-01	-1.539	5.71E-03	-1.943
TSC22D1	TSC22 domain family, member 1	1.00E+00	0	1.00E+00	0	1.00E+00	-3.302	2.90E-02	-3.96
VEZF1	vascular endothelial zinc finger 1					6.06E-02	-2.015	7.20E-03	-1.926
YBX3	Y box binding protein 3	6.81E-01	1.32	4.39E-01	-1.621	3.77E-02	-1.3	1.55E-02	-1.41
ZNF593	zinc finger protein 593	1.00E+00	0	1.00E+00	0	2.07E-01	-1.615	8.80E-03	-1.441

LMP1-no Inhibitors: Cleavage and PolyAdenylation of Pre-mRNA (P = 1)

None

LMP2-no Inhibitors: Cleavage and PolyAdenylation of Pre-mRNA (P = 0.0003)

		LMP1 no Inhs		LMP2 no Inhs		LMP1 with Inhs		LMP2 with Inhs	
**Symbol**	**Entrez Gene Name**	**p-value**	**Fold Change**	**p-value**	**Fold Change**	**p-value**	**Fold Change**	**p-value**	**Fold Change**
CPSF6	cleavage and polyadenylation specific factor 6, 68kDa	4.86E-01	-1.65	1.63E-03	1.269	9.69E-01	1.014	8.00E-01	-1.077
PABPN1	poly(A) binding protein, nuclear 1	4.09E-01	-1.239	1.42E-02	1.245	4.24E-01	-1.182	2.09E-01	1.491

LMP1-with Inhibitors: Cleavage and PolyAdenylation of Pre-mRNA (P = 0.04)

		LMP1 no Inhs		LMP2 no Inhs		LMP1 with Inhs		LMP2 with Inhs	
**Symbol**	**Entrez Gene Name**	**p-value**	**Fold Change**	**p-value**	**Fold Change**	**p-value**	**Fold Change**	**p-value**	**Fold Change**
CPSF3	cleavage and polyadenylation specific factor 3, 73kDa	2.10E-01	-1.563	4.60E-01	-1.418	3.02E-02	-1.601	1.42E-01	-1.175
CSTF2	cleavage stimulation factor, 3' pre-RNA, subunit 2, 64kDa	7.34E-01	-1.161	7.83E-01	-1.222	1.17E-02	-1.791	2.56E-01	-1.242

LMP2-with Inhibitors: Cleavage and PolyAdenylation of Pre-mRNA (P = 1)

None

LMP1-no Inhibitors: DNA methylation and transcriptional repression (P = 1)

		LMP1 no Inhs		LMP2 no Inhs		LMP1 with Inhs		LMP2 with Inhs	
none									

LMP2-no Inhibitors: DNA methylation and transcriptional repression (P = 1)

		LMP1 no Inhs		LMP2 no Inhs		LMP1 with Inhs		LMP2 with Inhs	
none									

LMP1-with Inhibitors: DNA methylation and transcriptional repression (P = 0.0018)

		LMP1 no Inhs		LMP2 no Inhs		LMP1 with Inhs		LMP2 with Inhs	
**Symbol**	**Entrez Gene Name**	**p-value**	**Fold Change**	**p-value**	**Fold Change**	**p-value**	**Fold Change**	**p-value**	**Fold Change**
HDAC2	histone deacetylase 2	1.00E+00	-3.841	1.00E+00	-1.241	4.63E-02	-1.22	4.45E-01	-1.123
RBBP7	retinoblastoma binding protein 7	9.33E-01	1.031	7.44E-01	1.077	1.38E-02	-3.281	6.28E-03	-6.779
SAP18	Sin3A-associated protein, 18kDa	3.87E-01	-1.837	1.00E+00	-1.141	1.58E-02	-1.203	2.11E-01	1.25
SAP130	Sin3A-associated protein, 130kDa	1.00E+00	0	1.00E+00	0	8.35E-03	-1.877	8.34E-01	1.023

LMP2-with Inhibitors: DNA methylation and transcriptional repression (P = 0.0062)

		LMP1 no Inhs		LMP2 no Inhs		LMP1 with Inhs		LMP2 with Inhs	
**Symbol**	**Entrez Gene Name**	**p-value**	**Fold Change**	**p-value**	**Fold Change**	**p-value**	**Fold Change**	**p-value**	**Fold Change**
CHD3	chromodomain helicase DNA binding protein 3	1.00E+00	0	1.00E+00	0	9.24E-02	-1.173	8.40E-03	1.216
CHD4	chromodomain helicase DNA binding protein 4	7.52E-01	-1.213	7.10E-01	1.11	9.81E-02	-1.276	4.86E-02	-1.42
RBBP7	retinoblastoma binding protein 7	9.33E-01	1.031	7.44E-01	1.077	1.38E-02	-3.281	6.28E-03	-6.779

LMP1-no Inhibitors: Processing of RNA (P = 0.0095)

		LMP1 no Inhs		LMP2 no Inhs		LMP1 with Inhs		LMP2 with Inhs	
**Symbol**	**Entrez Gene Name**	**p-value**	**Fold Change**	**p-value**	**Fold Change**	**p-value**	**Fold Change**	**p-value**	**Fold Change**
AARS	alanyl-tRNA synthetase	1.92E-02	-1.264	8.69E-01	-1.099	8.53E-03	-1.189	2.05E-01	1.093
HNRNPD	heterogeneous nuclear ribonucleoprotein D (AU-rich element RNA binding protein 1, 37kDa)	1.54E-03	-1.454	2.69E-01	-1.169	6.70E-01	2.011	5.09E-01	2.793
NCBP1	nuclear cap binding protein subunit 1, 80kDa	3.83E-02	-2.567	2.13E-01	-2.049	6.56E-01	1.111	5.33E-01	-1.062
SREK1	splicing regulatory glutamine/lysine-rich protein 1	3.30E-02	1.269	1.00E+00	2.535	9.32E-01	1.021	7.29E-01	1.067
SRSF5	serine/arginine-rich splicing factor 5	1.02E-02	1.674	2.60E-02	1.289	5.90E-01	-1.144	7.75E-01	1.067
THOC1	THO complex 1	7.01E-03	-2.126	1.00E+00	-2.396	3.57E-01	-1.181	8.65E-01	1.021

LMP2-no Inhibitors: Processing of RNA (P = 1)

		LMP1 no Inhs		LMP2 no Inhs		LMP1 with Inhs		LMP2 with Inhs	
**Symbol**	**Entrez Gene Name**	**p-value**	**Fold Change**	**p-value**	**Fold Change**	**p-value**	**Fold Change**	**p-value**	**Fold Change**
none									

LMP1-with Inhibitors: Processing of RNA (P = 4.62×10^{-5})

Symbol	Entrez Gene Name	p-value	Fold Change	p-value	Fold Change	p-value	Fold Change	p-value	Fold Change
AARS	alanyl-tRNA synthetase	1.92E-02	-1.264	8.69E-01	-1.099	8.53E-03	-1.189	2.05E-01	1.093
AGO2	argonaute RISC catalytic component 2	1.00E+00	1.578	1.00E+00	1.28	1.64E-02	-1.539	1.37E-01	-1.335
CLP1	cleavage and polyadenylation factor I subunit 1	1.00E+00	0	1.00E+00	0	2.63E-02	-1.566	1.00E+00	-1.783
CPSF3	cleavage and polyadenylation specific factor 3, 73kDa	2.10E-01	-1.563	4.60E-01	-1.418	3.02E-02	-1.601	1.42E-01	-1.175
CSTF2	cleavage stimulation factor, 3' pre-RNA, subunit 2, 64kDa	7.34E-01	-1.161	7.83E-01	-1.222	1.17E-02	-1.791	2.56E-01	-1.242
EFTUD2	elongation factor Tu GTP binding domain containing 2	7.55E-01	-1.107	7.59E-01	1.118	1.91E-02	-1.189	4.82E-02	-1.18
GEMIN5	gem (nuclear organelle) associated protein 5	1.36E-01	-2.474	9.82E-01	1.008	3.64E-02	-1.558	6.21E-01	1.116
GRSF1	G-rich RNA sequence binding factor 1	3.84E-01	-1.514	4.46E-01	-1.579	8.00E-04	-1.445	1.71E-03	-1.516
HNRNPK	heterogeneous nuclear ribonucleoprotein K	6.23E-01	1.079	6.77E-01	1.057	1.97E-02	-1.205	3.08E-01	-2.032
IMP3	IMP3, U3 small nucleolar ribonucleoprotein	8.90E-02	-2.463	1.00E+00	1.934	4.74E-02	-1.289	1.66E-01	-1.198
IMP4	IMP4, U3 small nucleolar ribonucleoprotein	1.00E+00	0	1.00E+00	0	3.30E-02	-2.288	1.81E-01	-1.48
KHDRBS3	KH domain containing, RNA binding, signal transduction associated 3					3.18E-02	1.751	6.46E-02	1.52
NOLC1	nucleolar and coiled-body phosphoprotein 1	5.80E-01	1.785	8.25E-01	1.283	4.09E-06	-1.731	3.65E-02	-8.09
PABPC4	poly(A) binding protein, cytoplasmic 4 (inducible form)	7.73E-01	-1.126	7.92E-01	1.027	6.62E-03	-1.267	7.58E-01	-1.046
SAP18	Sin3A-associated protein, 18kDa	3.87E-01	-1.837	1.00E+00	-1.141	1.58E-02	-1.203	2.11E-01	1.25
SCAF4	SR-related CTD-associated factor 4	1.00E+00	-1.978	1.00E+00	-1.804	3.08E-02	-1.704	3.49E-01	-1.334
SF3B1	splicing factor 3b, subunit 1, 155kDa	9.53E-01	-1.019	1.36E-01	-1.064	1.12E-02	-1.179	4.97E-01	1.046
SF3B2	splicing factor 3b, subunit 2, 145kDa	5.57E-01	1.231	4.54E-01	1.157	4.07E-02	-1.251	1.39E-01	-1.242
SNRNP20(	small nuclear ribonucleoprotein 200kDa (U5)	5.93E-01	-1.232	4.62E-01	-1.956	4.44E-04	-1.211	1.24E-03	-1.258
SNRPB2	small nuclear ribonucleoprotein polypeptide B	1.00E+00	0	1.00E+00	0	1.26E-02	-1.538	4.60E-01	-1.19
TRNT1	tRNA nucleotidyl transferase, CCA-adding, 1	5.05E-01	-1.093	1.00E+00	2.177	4.64E-04	2.083	2.44E-01	1.287
XAB2	XPA binding protein 2	1.00E+00	1.029	1.00E+00	1.851	2.07E-02	-1.513	2.66E-01	-1.533

LMP2-with Inhibitors: Processing of RNA (P = 1)

		LMP1 no Inhs		LMP2 no Inhs		LMP1 with Inhs		LMP2 with Inhs	
**Symbol**	**Entrez Gene Name**	**p-value**	**Fold Change**	**p-value**	**Fold Change**	**p-value**	**Fold Change**	**p-value**	**Fold Change**
none									

LMP1-no Inhibitors: tRNA charging (P = 0.0118)

		LMP1 no Inhs		LMP2 no Inhs		LMP1 with Inhs		LMP2 with Inhs	
Symbol	Entrez Gene Name	p-value	Fold Change	p-value	Fold Change	p-value	Fold Change	p-value	Fold Change
AARS	alanyl-tRNA synthetase	1.92E-02	-1.264	8.69E-01	-1.099	8.53E-03	-1.189	2.05E-01	1.093
FARSB	phenylalanyl-tRNA synthetase, beta subunit	1.46E-02	-1.791	5.16E-01	-1.186	3.76E-02	-1.225	8.77E-01	1.013
GARS	glycyl-tRNA synthetase	4.89E-02	-1.532	3.92E-02	-2.567	8.52E-01	-1.077	4.86E-01	-1.325

LMP2-no Inhibitors: tRNA charging (P = 0.1665)

		LMP1 no Inhs		LMP2 no Inhs		LMP1 with Inhs		LMP2 with Inhs	
Symbol	Entrez Gene Name	p-value	Fold Change	p-value	Fold Change	p-value	Fold Change	p-value	Fold Change
GARS	glycyl-tRNA synthetase	4.89E-02	-1.532	3.92E-02	-2.567	8.52E-01	-1.077	4.86E-01	-1.325

LMP1-with Inhibitors: tRNA charging (P = 0.071)

		LMP1 no Inhs		LMP2 no Inhs		LMP1 with Inhs		LMP2 with Inhs	
Symbol	Entrez Gene Name	p-value	Fold Change	p-value	Fold Change	p-value	Fold Change	p-value	Fold Change
AARS	alanyl-tRNA synthetase	1.92E-02	-1.264	8.69E-01	-1.099	8.53E-03	-1.189	2.05E-01	1.093
DARS	aspartyl-tRNA synthetase	9.00E-01	-1.035	8.33E-01	1.067	4.73E-02	-1.295	2.76E-01	-1.181
EPRS	glutamyl-prolyl-tRNA synthetase	8.40E-02	-1.28	4.72E-01	-1.176	3.69E-02	-1.214	6.05E-01	1.029
FARSB	phenylalanyl-tRNA synthetase, beta subunit	1.46E-02	-1.791	5.16E-01	-1.186	3.76E-02	-1.225	8.77E-01	1.013
VARS	valyl-tRNA synthetase	1.46E-01	-1.412	6.13E-01	1.04	2.67E-02	-1.144	2.92E-01	-1.065

LMP2-with Inhibitors: tRNA charging (P = 0.467)

		LMP1 no Inhs		LMP2 no Inhs		LMP1 with Inhs		LMP2 with Inhs	
Symbol	Entrez Gene Name	p-value	Fold Change	p-value	Fold Change	p-value	Fold Change	p-value	Fold Change
CARS	cysteinyl-tRNA synthetase	8.68E-01	1.022	7.10E-01	1.067	1.54E-01	-1.124	1.19E-02	1.196
RARS	arginyl-tRNA synthetase	7.76E-01	-1.084	9.21E-01	-1.036	2.97E-01	-1.164	2.82E-03	-1.816
