# Supplementary material for: Global Proteomic Changes Induced by the Epstein-Barr Virus Oncoproteins Latent Membrane Protein 1 and 2A
Source: mBio. 2018 Jun 19;9(3):e00959-18. doi: 10.1128/mBio.00959-18 (PMC6016245; doi:10.1128/mBio.00959-18)
Supplement: TABLE S4 [file mbo003183928st4.pdf]

Supplemental Table-4

LMP1-no Inhibitors: Integrin signaling (P = 0.0063)

		LMP1 no Inhs		LMP2 no Inhs		LMP1 with Inhs		LMP2 with Inhs	
**Symbol**	**Entrez Gene Name**	**p-value**	**Fold Change**	**p-value**	**Fold Change**	**p-value**	**Fold Change**	**p-value**	**Fold Change**
ARPC4	actin related protein 2/3 complex, subunit 4, 20kDa	1.11E-02	-1.511	9.32E-01	-1.010	5.30E-01	-1.042	2.83E-01	1.177
CAPN1	calpain 1, (mu/l) large subunit	4.38E-02	-1.239	9.13E-01	1.017	1.09E-01	1.277	8.11E-01	1.037
CAPNS1	calpain, small subunit 1	1.07E-02	1.386	1.35E-01	1.316	2.97E-01	1.078	1.28E-01	-1.125
RAP1B	RAP1B, member of RAS oncogene family	3.17E-02	-3.786	1.00E00	-3.401	8.56E-01	1.071	9.80E-01	1.010
VCL	vinculin	4.67E-02	1.468	9.93E-01	-1.001	9.62E-02	-1.607	1.00E00	-1.583

LMP2-no Inhibitors: Integrin signaling (P = 1)

None

LMP1-with Inhibitors: Integrin signaling (P = 3.67 x 10^{-5})

		LMP1 no Inhs		LMP2 no Inhs		LMP1 with Inhs		LMP2 with Inhs	
**Symbol**	**Entrez Gene Name**	**p-value**	**Fold Change**	**p-value**	**Fold Change**	**p-value**	**Fold Change**	**p-value**	**Fold Change**
ACTG1	actin, gamma 1	4.38E-01	2.212	9.31E-01	1.090	6.29E-03	1.182	1.00E00	-1.548
ACTN1	actinin, alpha 1	2.46E-01	1.725	7.05E-01	-1.250	1.22E-03	1.884	9.70E-01	1.079
ACTN4	actinin, alpha 4	3.46E-01	1.264	9.40E-01	1.023	3.67E-04	2.025	5.14E-01	1.040
ARPC1A	actin related protein 2/3 complex, subunit 1A, 41kDa	3.33E-01	1.922	7.89E-01	1.212	3.81E-02	1.382	2.37E-01	-1.175
CRKL	v-crk avian sarcoma virus CT10 oncogene homolog-like	7.18E-01	1.127	9.97E-01	1.001	3.25E-02	2.215	7.70E-01	1.132
GRB7	growth factor receptor-bound protein 7	1.00E00	0.000	1.00E00	0.000	3.20E-02	1.867	1.00E00	1.058
ILKAP	integrin-linked kinase-associated serine/threonine phosphatase	1.00E00	-1.000	1.00E00	-1.490	4.27E-02	-1.589	2.27E-01	-1.253
ITGA5	integrin, alpha 5 (fibronectin receptor, alpha polypeptide)	3.64E-01	-2.172	1.00E00	-3.726	4.33E-02	-2.098	2.27E-02	-2.119
ITGB4	integrin, beta 4	5.06E-01	-1.180	7.55E-01	-1.092	3.98E-02	-1.164	1.00E00	-1.292
MAP2K2	mitogen-activated protein kinase kinase 2	1.00E00	-1.030	1.00E00	1.079	2.57E-02	-1.397	2.02E-02	-1.306
MYL9	myosin, light chain 9, regulatory	1.00E00	0.000	1.00E00	0.000	4.54E-02	3.181	1.00E00	-1.094
NCK1	NCK adaptor protein 1	1.00E00	1.371	1.00E00	1.688	3.97E-03	1.850	3.49E-01	1.681
PAK1	p21 protein (Cdc42/Rac)-activated kinase 1					3.34E-02	-1.594	1.00E00	
RAC2	ras-related C3 botulinum toxin substrate 2 (rho family, small GTP binding protein Rac2)	1.00E00	0.000	1.00E00	0.000	1.04E-03	1.399	7.67E-02	-1.210
RAP2B	RAP2B, member of RAS oncogene family	1.00E00	0.000	1.00E00	0.000	2.53E-04	2.586	5.82E-01	-1.170
RHOT1	ras homolog family member T1	1.00E00	1.452	1.00E00	-1.341	4.97E-02	2.050	6.21E-01	1.195
TTN	titin	1.00E00	0.000	1.00E00	0.000	1.49E-02	1.477	7.70E-02	1.501

LMP2-with Inhibitors: Integrin signaling (P = 0.1914)

		LMP1 no Inhs		LMP2 no Inhs		LMP1 with Inhs		LMP2 with Inhs	
**Symbol**	**Entrez Gene Name**	**p-value**	**Fold Change**	**p-value**	**Fold Change**	**p-value**	**Fold Change**	**p-value**	**Fold Change**
ACTR2	ARP2 actin-related protein 2 homolog (yeast)	3.55E-01	1.594	9.05E-01	-1.028	8.73E-01	-1.010	4.13E-02	-1.124
ITGA5	integrin, alpha 5 (fibronectin receptor, alpha polypeptide)	3.64E-01	-2.172	1.00E00	-3.726	4.33E-02	-2.098	2.27E-02	-2.119
ITGAE	integrin, alpha E (antigen CD103, human mucosal lymphocyte antigen 1; alpha polypep					7.25E-01	-1.098	4.70E-02	-1.586
MAP2K2	mitogen-activated protein kinase kinase 2	1.00E00	-1.030	1.00E00	1.079	2.57E-02	-1.397	2.02E-02	-1.306
PXN	paxillin	2.26E-01	1.287	1.27E-01	-1.543	3.24E-01	1.546	1.68E-02	-1.271
TLN1	talin 1	6.90E-01	1.081	1.64E-01	-1.316	7.06E-02	1.063	2.74E-02	-1.154

LMP1-no Inhibitors: ILK signaling (P = 0.294)

Symbol	Entrez Gene Name	LMP1 no Inhs p-value	LMP1 no Inhs Fold Change	LMP2 no Inhs p-value	LMP2 no Inhs Fold Change	LMP1 with Inhs p-value	LMP1 with Inhs Fold Change	LMP2 with Inhs p-value	LMP2 with Inhs Fold Change
DSP	desmoplakin	1.42E-02	1.418	6.00E-01	1.081	7.88E-01	1.044	8.70E-01	1.019
KRT18	keratin 18	1.12E-02	-1.391	3.43E-01	-1.225	2.47E-01	1.202	3.85E-01	1.15

LMP2-no Inhibitors: ILK signaling (P = 1)

LMP1 no Inhs | LMP2 no Inhs | LMP1 with Inhs | LMP2 with Inhs

none

LMP1-with Inhibitors: ILK signaling (P = 0.0052)

Symbol	Entrez Gene Name	LMP1 no Inhs p-value	LMP1 no Inhs Fold Change	LMP2 no Inhs p-value	LMP2 no Inhs Fold Change	LMP1 with Inhs p-value	LMP1 with Inhs Fold Change	LMP2 with Inhs p-value	LMP2 with Inhs Fold Change
ACTG1	actin, gamma 1	4.38E-01	2.212	9.31E-01	1.09	6.29E-03	1.182	1.00E+00	-1.548
ACTN1	actinin, alpha 1	2.46E-01	1.725	7.05E-01	-1.25	1.22E-03	1.884	9.70E-01	1.079
ACTN4	actinin, alpha 4	3.46E-01	1.264	9.40E-01	1.023	3.67E-04	2.025	5.14E-01	1.04
FLNB	filamin B, beta	1.24E-01	1.607	6.49E-01	1.104	3.33E-02	1.281	1.84E-01	-3.752
ILKAP	integrin-linked kinase-associated serine/threonine phosphatase	1.00E+00	-1	1.00E+00	-1.49	4.27E-02	-1.589	2.27E-01	-1.253
ITGB4	integrin, beta 4	5.06E-01	-1.18	7.55E-01	-1.092	3.98E-02	-1.164	1.00E+00	-1.292
MYL6	myosin, light chain 6, alkali, smooth muscle and non-muscle	5.11E-02	1.436	4.10E-01	1.254	1.40E-02	1.973	9.73E-03	1.755
MYL9	myosin, light chain 9, regulatory	1.00E+00	0	1.00E+00	0	4.54E-02	3.181	1.00E+00	-1.094
RHOT1	ras homolog family member T1	1.00E+00	1.452	1.00E+00	-1.341	4.97E-02	2.05	6.21E-01	1.195
RPS6KA4	ribosomal protein S6 kinase, 90kDa, polypeptide 4	1.00E+00	-1.026	1.00E+00	-1.418	2.13E-02	-1.624	1.13E-01	-1.257
TMSB10/TMS	thymosin beta 10	2.63E-01	3.37	2.81E-01	1.836	3.77E-02	5.288	7.55E-01	-1.269
VIM	vimentin	9.23E-01	-1.058	7.32E-01	-1.226	9.94E-04	-1.74	2.98E-02	-1.433

LMP2-with Inhibitors: ILK signaling (P = 0.2916)

Symbol	Entrez Gene Name	LMP1 no Inhs p-value	LMP1 no Inhs Fold Change	LMP2 no Inhs p-value	LMP2 no Inhs Fold Change	LMP1 with Inhs p-value	LMP1 with Inhs Fold Change	LMP2 with Inhs p-value	LMP2 with Inhs Fold Change
MYL6	myosin, light chain 6, alkali, smooth muscle and non-muscle	5.11E-02	1.436	4.10E-01	1.254	1.40E-02	1.973	9.73E-03	1.755
NFKB1	nuclear factor of kappa light polypeptide gene enhancer in B-cells 1	7.54E-01	-1.28	8.35E-01	1.177	9.42E-02	1.205	1.63E-02	1.226
PPP2R2A	protein phosphatase 2, regulatory subunit B, alpha	4.81E-01	1.459	8.73E-01	1.147	2.85E-01	-1.232	7.48E-03	-1.358
PXN	paxillin	2.26E-01	1.287	1.27E-01	-1.543	3.24E-01	1.546	1.68E-02	-1.271
VIM	vimentin	9.23E-01	-1.058	7.32E-01	-1.226	9.94E-04	-1.74	2.98E-02	-1.433

LMP1-no Inhibitors: Rac Signaling (P = 0.461)

		LMP1 no Inhs		LMP2 no Inhs		LMP1 with Inhs		LMP2 with Inhs	
**Symbol**	**Entrez Gene Name**	**p-value**	**Fold Change**	**p-value**	**Fold Change**	**p-value**	**Fold Change**	**p-value**	**Fold Change**
ARPC4	actin related protein 2/3 complex, subunit 4, 20kDa	1.11E-02	-1.511	9.32E-01	-1.010	5.30E-01	-1.042	2.83E-01	1.177

LMP2-no Inhibitors: Rac Signaling (P = 0.212)

		LMP1 no Inhs		LMP2 no Inhs		LMP1 with Inhs		LMP2 with Inhs	
**Symbol**	**Entrez Gene Name**	**p-value**	**Fold Change**	**p-value**	**Fold Change**	**p-value**	**Fold Change**	**p-value**	**Fold Change**
BAIAP2	BAI1-associated protein 2	9.89E-01	-1.006	3.81E-02	-1.488	1.71E-01	1.399	7.03E-01	-1.090

LMP1-with Inhibitors: Rac Signaling (P = 0.026)

Symbol	Entrez Gene Name	p-value	Fold Change	p-value	Fold Change	p-value	Fold Change	p-value	Fold Change
ARPC1A	actin related protein 2/3 complex, subunit 1A, 41kDa	3.33E-01	1.922	7.89E-01	1.212	3.81E-02	1.382	2.37E-01	-1.175
CD44	CD44 molecule (Indian blood group)	4.40E-01	-2.277	1.34E-01	-3.127	5.16E-03	-1.316	4.93E-01	-1.089
CYFIP2	cytoplasmic FMR1 interacting protein 2					4.71E-02	-1.275	1.42E-01	-3.469
IQGAP3	IQ motif containing GTPase activating protein 3	1.00E00	0.000	1.00E00	0.000	8.66E-03	2.330	3.17E-01	-1.120
ITGA5	integrin, alpha 5 (fibronectin receptor, alpha polypeptide)	3.64E-01	-2.172	1.00E00	-3.726	4.33E-02	-2.098	2.27E-02	-2.119
MAP2K2	mitogen-activated protein kinase kinase 2	1.00E00	-1.030	1.00E00	1.079	2.57E-02	-1.397	2.02E-02	-1.306
PAK1	p21 protein (Cdc42/Rac)-activated kinase 1					3.34E-02	-1.594	1.00E00	

LMP2-with Inhibitors: Rac Signaling (P = 0.0045)

		LMP1 no Inhs		LMP2 no Inhs		LMP1 with Inhs		LMP2 with Inhs	
**Symbol**	**Entrez Gene Name**	**p-value**	**Fold Change**	**p-value**	**Fold Change**	**p-value**	**Fold Change**	**p-value**	**Fold Change**
ACTR2	ARP2 actin-related protein 2 homolog (yeast)	3.55E-01	1.594	9.05E-01	-1.028	8.73E-01	-1.010	4.13E-02	-1.124
BRK1	BRICK1, SCAR/WAVE actin-nucleating complex subunit	1.00E00	1.672	1.00E00	0.000	4.45E-01	1.045	2.15E-02	-1.382
ITGA5	integrin, alpha 5 (fibronectin receptor, alpha polypeptide)	3.64E-01	-2.172	1.00E00	-3.726	4.33E-02	-2.098	2.27E-02	-2.119
MAP2K2	mitogen-activated protein kinase kinase 2	1.00E00	-1.030	1.00E00	1.079	2.57E-02	-1.397	2.02E-02	-1.306
NCKAP1	NCK-associated protein 1	5.12E-01	1.397	1.00E00	1.192	1.94E-01	-1.207	1.15E-02	-1.502
NFKB1	nuclear factor of kappa light polypeptide gene enhancer in B-cells 1	7.54E-01	-1.280	8.35E-01	1.177	9.42E-02	1.205	1.63E-02	1.226
RPS6KB1	ribosomal protein S6 kinase, 70kDa, polypeptide 1	1.00E00	1.861	1.00E00	1.983	1.04E-01	-1.675	3.62E-02	-1.878

LMP1-no Inhibitors: Leukocyte Extravasation Signaling (P = 0.328)

		LMP1 no Inhs		LMP2 no Inhs		LMP1 with Inhs		LMP2 with Inhs	
**Symbol**	**Entrez Gene Name**	**p-value**	**Fold Change**	**p-value**	**Fold Change**	**p-value**	**Fold Change**	**p-value**	**Fold Change**
RAP1B	RAP1B, member of RAS oncogene family	3.17E-02	-3.786	1.00E00	-3.401	8.56E-01	1.071	9.80E-01	1.010
VCL	vinculin	4.67E-02	1.468	9.93E-01	-1.001	9.62E-02	-1.607	1.00E00	-1.583

LMP2-no Inhibitors: Leukocyte Extravasation Signaling (P = 1)

None

LMP1-with Inhibitors: Leukocyte Extravasation Signaling (P = 0.00045)

		LMP1 no Inhs		LMP2 no Inhs		LMP1 with Inhs		LMP2 with Inhs	
**Symbol**	**Entrez Gene Name**	**p-value**	**Fold Change**	**p-value**	**Fold Change**	**p-value**	**Fold Change**	**p-value**	**Fold Change**
ACTG1	actin, gamma 1	4.38E-01	2.212	9.31E-01	1.090	6.29E-03	1.182	1.00E00	-1.548
ACTN1	actinin, alpha 1	2.46E-01	1.725	7.05E-01	-1.250	1.22E-03	1.884	9.70E-01	1.079
ACTN4	actinin, alpha 4	3.46E-01	1.264	9.40E-01	1.023	3.67E-04	2.025	5.14E-01	1.040
CD44	CD44 molecule (Indian blood group)	4.40E-01	-2.277	1.34E-01	-3.127	5.16E-03	-1.316	4.93E-01	-1.089
CD99	CD99 molecule	1.00E00	0.000	1.00E00	0.000	1.94E-02	2.356	2.42E-01	1.329
CRKL	v-crk avian sarcoma virus CT10 oncogene homolog-like	7.18E-01	1.127	9.97E-01	1.001	3.25E-02	2.215	7.70E-01	1.132
EZR	ezrin	4.70E-01	1.533	6.72E-01	1.354	3.55E-02	2.326	9.76E-01	-1.012
ICAM1	intercellular adhesion molecule 1	1.00E00	1.102	1.00E00	0.000	1.20E-02	4.093	4.97E-01	-1.175
ITGA5	integrin, alpha 5 (fibronectin receptor, alpha polypeptide)	3.64E-01	-2.172	1.00E00	-3.726	4.33E-02	-2.098	2.27E-02	-2.119
MAP2K2	mitogen-activated protein kinase kinase 2	1.00E00	-1.030	1.00E00	1.079	2.57E-02	-1.397	2.02E-02	-1.306
MLLT4	myeloid/lymphoid or mixed-lineage leukemia (trithorax homolog, Drosophila); translocat	9.93E-01	1.006	8.64E-01	1.126	5.67E-03	1.452	4.75E-01	1.126
MYL6	myosin, light chain 6, alkali, smooth muscle and non-muscle	5.11E-02	1.436	4.10E-01	1.254	1.40E-02	1.973	9.73E-03	1.755
RAC2	ras-related C3 botulinum toxin substrate 2 (rho family, small GTP binding protein Rac2)	1.00E00	0.000	1.00E00	0.000	1.04E-03	1.399	7.67E-02	-1.210
TIMP1	TIMP metallopeptidase inhibitor 1	8.12E-01	1.079	1.00E00	-1.174	1.98E-02	1.741	9.21E-01	1.008
TIMP3	TIMP metallopeptidase inhibitor 3	1.00E00	-2.697	1.00E00	0.000	4.13E-03	-1.852	4.38E-01	-1.628

LMP2-with Inhibitors: Leukocyte Extravasation Signaling (P = 0.352)

		LMP1 no Inhs		LMP2 no Inhs		LMP1 with Inhs		LMP2 with Inhs	
**Symbol**	**Entrez Gene Name**	**p-value**	**Fold Change**	**p-value**	**Fold Change**	**p-value**	**Fold Change**	**p-value**	**Fold Change**
GNAI3	guanine nucleotide binding protein (G protein), alpha inhibiting activity polypeptide 3	9.21E-01	-1.135	1.00E00	2.354	2.38E-01	-1.140	3.91E-03	-1.355
ITGA5	integrin, alpha 5 (fibronectin receptor, alpha polypeptide)	3.64E-01	-2.172	1.00E00	-3.726	4.33E-02	-2.098	2.27E-02	-2.119
MAP2K2	mitogen-activated protein kinase kinase 2	1.00E00	-1.030	1.00E00	1.079	2.57E-02	-1.397	2.02E-02	-1.306
MYL6	myosin, light chain 6, alkali, smooth muscle and non-muscle	5.11E-02	1.436	4.10E-01	1.254	1.40E-02	1.973	9.73E-03	1.755
PXN	paxillin	2.26E-01	1.287	1.27E-01	-1.543	3.24E-01	1.546	1.68E-02	-1.271

LMP1-no Inhibitors: Signaling by RhoFamily GTPases (P = 1)

		LMP1 no Inhs		LMP2 no Inhs		LMP1 with Inhs		LMP2 with Inhs	
**Symbol**	**Entrez Gene Name**	**p-value**	**Fold Change**	**p-value**	**Fold Change**	**p-value**	**Fold Change**	**p-value**	**Fold Change**
ARPC4	actin related protein 2/3 complex, subunit 4, 20kDa	1.11E-02	-1.511	9.32E-01	-1.01	5.30E-01	-1.042	2.83E-01	1.177

LMP2-no Inhibitors: Signaling by RhoFamily GTPases (P = 0.409)

		LMP1 no Inhs		LMP2 no Inhs		LMP1 with Inhs		LMP2 with Inhs	
**Symbol**	**Entrez Gene Name**	**p-value**	**Fold Change**	**p-value**	**Fold Change**	**p-value**	**Fold Change**	**p-value**	**Fold Change**
BAIAP2	BAI1-associated protein 2	9.89E-01	-1.006	3.81E-02	-1.488	1.71E-01	1.399	7.03E-01	-1.09

LMP1-with Inhibitors: Signaling by RhoFamily GTPases (P = 0.0051)

		LMP1 no Inhs		LMP2 no Inhs		LMP1 with Inhs		LMP2 with Inhs	
**Symbol**	**Entrez Gene Name**	**p-value**	**Fold Change**	**p-value**	**Fold Change**	**p-value**	**Fold Change**	**p-value**	**Fold Change**
ACTG1	actin, gamma 1	4.38E-01	2.212	9.31E-01	1.09	6.29E-03	1.182	1.00E+00	-1.548
ARHGEF11	Rho guanine nucleotide exchange factor (GEF) 11	1.00E+00	0	1.00E+00	0	1.83E-02	1.612	6.07E-01	-1.132
ARPC1A	actin related protein 2/3 complex, subunit 1A, 41kDa	3.33E-01	1.922	7.89E-01	1.212	3.81E-02	1.382	2.37E-01	-1.175
CDC42EP1	CDC42 effector protein (Rho GTPase binding) 1	1.00E+00	0	1.00E+00	0	7.91E-04	3.842	1.48E-01	-1.617
CLIP1	CAP-GLY domain containing linker protein 1	7.27E-01	1.138	8.90E-02	-2.449	4.59E-03	1.901	5.89E-01	1.072
EZR	ezrin	4.70E-01	1.533	6.72E-01	1.354	3.55E-02	2.326	9.76E-01	-1.012
ITGA5	integrin, alpha 5 (fibronectin receptor, alpha polypeptide)	3.64E-01	-2.172	1.00E+00	-3.726	4.33E-02	-2.098	2.27E-02	-2.119
MAP2K2	mitogen-activated protein kinase kinase 2	1.00E+00	-1.03	1.00E+00	1.079	2.57E-02	-1.397	2.02E-02	-1.306
MYL6	myosin, light chain 6, alkali, smooth muscle and non-muscle	5.11E-02	1.436	4.10E-01	1.254	1.40E-02	1.973	9.73E-03	1.755
MYL9	myosin, light chain 9, regulatory	1.00E+00	0	1.00E+00	0	4.54E-02	3.181	1.00E+00	-1.094
PAK1	p21 protein (Cdc42/Rac)-activated kinase 1					3.34E-02	-1.594	1.00E+00	
RHOT1	ras homolog family member T1	1.00E+00	1.452	1.00E+00	-1.341	4.97E-02	2.05	6.21E-01	1.195
SEPT8	septin 8					3.23E-02	-1.242	1.28E-01	-1.163
VIM	vimentin	9.23E-01	-1.058	7.32E-01	-1.226	9.94E-04	-1.74	2.98E-02	-1.433

LMP2-with Inhibitors: Signaling by RhoFamily GTPases (P = 0.168)

		LMP1 no Inhs		LMP2 no Inhs		LMP1 with Inhs		LMP2 with Inhs	
**Symbol**	**Entrez Gene Name**	**p-value**	**Fold Change**	**p-value**	**Fold Change**	**p-value**	**Fold Change**	**p-value**	**Fold Change**
ACTR2	ARP2 actin-related protein 2 homolog (yeast)	3.55E-01	1.594	9.05E-01	-1.028	8.73E-01	-1.01	4.13E-02	-1.124
GNAI3	guanine nucleotide binding protein (G protein), alpha inhibiting activity polypeptide 3	9.21E-01	-1.135	1.00E+00	2.354	2.38E-01	-1.14	3.91E-03	-1.355
ITGA5	integrin, alpha 5 (fibronectin receptor, alpha polypeptide)	3.64E-01	-2.172	1.00E+00	-3.726	4.33E-02	-2.098	2.27E-02	-2.119
MAP2K2	mitogen-activated protein kinase kinase 2	1.00E+00	-1.03	1.00E+00	1.079	2.57E-02	-1.397	2.02E-02	-1.306
MYL6	myosin, light chain 6, alkali, smooth muscle and non-muscle	5.11E-02	1.436	4.10E-01	1.254	1.40E-02	1.973	9.73E-03	1.755
NFKB1	nuclear factor of kappa light polypeptide gene enhancer in B-cells 1	7.54E-01	-1.28	8.35E-01	1.177	9.42E-02	1.205	1.63E-02	1.226
VIM	vimentin	9.23E-01	-1.058	7.32E-01	-1.226	9.94E-04	-1.74	2.98E-02	-1.433

	LMP1-no Inhibitors: RhoA signaling (P = 0.512)	LMP1 no Inhs		LMP2 no Inhs		LMP1 with Inhs		LMP2 with Inhs	
**Symbol**	**Entrez Gene Name**	**p-value**	**Fold Change**	**p-value**	**Fold Change**	**p-value**	**Fold Change**	**p-value**	**Fold Change**
ARPC4	actin related protein 2/3 complex, subunit 4, 20kDa	1.11E-02	-1.511	9.32E-01	-1.01	5.30E-01	-1.042	2.83E-01	1.177

	LMP2-no Inhibitors: RhoA signaling (P = 0.241)	LMP1 no Inhs		LMP2 no Inhs		LMP1 with Inhs		LMP2 with Inhs	
**Symbol**	**Entrez Gene Name**	**p-value**	**Fold Change**	**p-value**	**Fold Change**	**p-value**	**Fold Change**	**p-value**	**Fold Change**
BAIAP2	BAI1-associated protein 2	9.89E-01	-1.006	3.81E-02	-1.488	1.71E-01	1.399	7.03E-01	-1.09

	LMP1-with Inhibitors: RhoA signaling (P = 0.0065)	LMP1 no Inhs		LMP2 no Inhs		LMP1 with Inhs		LMP2 with Inhs	
**Symbol**	**Entrez Gene Name**	**p-value**	**Fold Change**	**p-value**	**Fold Change**	**p-value**	**Fold Change**	**p-value**	**Fold Change**
ACTG1	actin, gamma 1	4.38E-01	2.212	9.31E-01	1.09	6.29E-03	1.182	1.00E+00	-1.548
ARHGEF11	Rho guanine nucleotide exchange factor (GEF) 11	1.00E+00	0	1.00E+00	0	1.83E-02	1.612	6.07E-01	-1.132
ARPC1A	actin related protein 2/3 complex, subunit 1A, 41kDa	3.33E-01	1.922	7.89E-01	1.212	3.81E-02	1.382	2.37E-01	-1.175
CDC42EP1	CDC42 effector protein (Rho GTPase binding) 1	1.00E+00	0	1.00E+00	0	7.91E-04	3.842	1.48E-01	-1.617
EZR	ezrin	4.70E-01	1.533	6.72E-01	1.354	3.55E-02	2.326	9.76E-01	-1.012
MYL6	myosin, light chain 6, alkali, smooth muscle and non-muscle	5.11E-02	1.436	4.10E-01	1.254	1.40E-02	1.973	9.73E-03	1.755
MYL9	myosin, light chain 9, regulatory	1.00E+00	0	1.00E+00	0	4.54E-02	3.181	1.00E+00	-1.094
SEPT8	septin 8					3.23E-02	-1.242	1.28E-01	-1.163
TTN	titin	1.00E+00	0	1.00E+00	0	1.49E-02	1.477	7.70E-02	1.501

	LMP2-with Inhibitors: RhoA signaling (P = 1)	LMP1 no Inhs		LMP2 no Inhs		LMP1 with Inhs		LMP2 with Inhs	
**Symbol**	**Entrez Gene Name**	**p-value**	**Fold Change**	**p-value**	**Fold Change**	**p-value**	**Fold Change**	**p-value**	**Fold Change**
ACTR2	ARP2 actin-related protein 2 homolog (yeast)	3.55E-01	1.594	9.05E-01	-1.028	8.73E-01	-1.01	4.13E-02	-1.124
MYL6	myosin, light chain 6, alkali, smooth muscle and non-muscle	5.11E-02	1.436	4.10E-01	1.254	1.40E-02	1.973	9.73E-03	1.755

LMP1-no Inhibitors: Cell Movement of tumor Cells (P = 1)

None

LMP2-no Inhibitors: Cell Movement of tumor Cells (P = 1)

None

LMP1-with Inhibitors: Cell Movement of tumor Cells (P = 0.0016)

		LMP1 no Inhs		LMP2 no Inhs		LMP1 with Inhs		LMP2 with Inhs	
**Symbol**	**Entrez Gene Name**	**p-value**	**Fold Change**	**p-value**	**Fold Change**	**p-value**	**Fold Change**	**p-value**	**Fold Change**
CD44	CD44 molecule (Indian blood group)	4.40E-01	-2.277	1.34E-01	-3.127	5.16E-03	-1.316	4.93E-01	-1.089
CD97	CD97 molecule	1.00E00	0.000	1.00E00	0.000	4.05E-02	1.752	2.20E-01	-1.646
CTSB	cathepsin B	9.68E-02	1.528	9.13E-01	1.055	1.55E-02	1.920	1.58E-01	1.368
EZR	ezrin	4.70E-01	1.533	6.72E-01	1.354	3.55E-02	2.326	9.76E-01	-1.012
F3	coagulation factor III (thromboplastin, tissue factor)	1.00E00	2.169	1.00E00	0.000	4.20E-03	4.412	3.68E-01	1.350
GSN	gelsolin	4.22E-01	1.945	1.00E00	-1.706	2.18E-03	1.703	1.23E-01	-1.197
LAMA5	laminin, alpha 5	2.69E-01	-2.706	1.00E00	-3.277	1.45E-02	-1.401	4.71E-02	-2.294
LGALS3	lectin, galactoside-binding, soluble, 3	2.19E-01	1.602	5.08E-01	-1.695	1.83E-02	2.188	6.11E-01	1.139
MET	MET proto-oncogene, receptor tyrosine kinase	1.00E00	0.000	1.00E00	0.000	4.99E-02	-1.277	6.91E-01	1.046
PAK1	p21 protein (Cdc42/Rac)-activated kinase 1					3.34E-02	-1.594	1.00E00	
S100A2	S100 calcium binding protein A2	6.15E-01	1.327	2.43E-02	2.805	1.50E-03	-2.333	3.34E-01	-2.009
S100P	S100 calcium binding protein P	1.00E00	0.000	1.00E00	0.000	1.45E-02	61.983	5.04E-01	2.709
SLPI	secretory leukocyte peptidase inhibitor					1.03E-02	9.998	3.86E-01	1.397
VIM	vimentin	9.23E-01	-1.058	7.32E-01	-1.226	9.94E-04	-1.740	2.98E-02	-1.433

LMP2-with Inhibitors: Cell Movement of tumor Cells (P = 1)

None

LMP1-no Inhibitors: Regulation of Actin-based motility by Rho (P = 0.41)

		LMP1 no Inhs		LMP2 no Inhs		LMP1 with Inhs		LMP2 with Inhs	
**Symbol**	**Entrez Gene Name**	**p-value**	**Fold Change**	**p-value**	**Fold Change**	**p-value**	**Fold Change**	**p-value**	**Fold Change**
ARPC4	actin related protein 2/3 complex, subunit 4, 20kDa	1.11E-02	-1.511	9.32E-01	-1.01	5.30E-01	-1.042	2.83E-01	1.177

LMP2-no Inhibitors: Regulation of Actin-based motility by Rho (P = 0.19)

		LMP1 no Inhs		LMP2 no Inhs		LMP1 with Inhs		LMP2 with Inhs	
**Symbol**	**Entrez Gene Name**	**p-value**	**Fold Change**	**p-value**	**Fold Change**	**p-value**	**Fold Change**	**p-value**	**Fold Change**
BAIAP2	BAI1-associated protein 2	9.89E-01	-1.006	3.81E-02	-1.488	1.71E-01	1.399	7.03E-01	-1.09

LMP1-with Inhibitors: Regulation of Actin-based motility by Rho (P = 0.0037)

		LMP1 no Inhs		LMP2 no Inhs		LMP1 with Inhs		LMP2 with Inhs	
**Symbol**	**Entrez Gene Name**	**p-value**	**Fold Change**	**p-value**	**Fold Change**	**p-value**	**Fold Change**	**p-value**	**Fold Change**
ARPC1A	actin related protein 2/3 complex, subunit 1A, 41kDa	3.33E-01	1.922	7.89E-01	1.212	3.81E-02	1.382	2.37E-01	-1.175
GSN	gelsolin	4.22E-01	1.945	1.00E+00	-1.706	2.18E-03	1.703	1.23E-01	-1.197
ITGA5	integrin, alpha 5 (fibronectin receptor, alpha polypeptide)	3.64E-01	-2.172	1.00E+00	-3.726	4.33E-02	-2.098	2.27E-02	-2.119
MYL6	myosin, light chain 6, alkali, smooth muscle and non-muscle	5.11E-02	1.436	4.10E-01	1.254	1.40E-02	1.973	9.73E-03	1.755
MYL9	myosin, light chain 9, regulatory	1.00E+00	0	1.00E+00	0	4.54E-02	3.181	1.00E+00	-1.094
PAK1	p21 protein (Cdc42/Rac)-activated kinase 1					3.34E-02	-1.594	1.00E+00	
RAC2	ras-related C3 botulinum toxin substrate 2 (rho family, small GTP binding protein Rac2)	1.00E+00	0	1.00E+00	0	1.04E-03	1.399	7.67E-02	-1.21
RHOT1	ras homolog family member T1	1.00E+00	1.452	1.00E+00	-1.341	4.97E-02	2.05	6.21E-01	1.195

LMP2-with Inhibitors: Regulation of Actin-based motility by Rho (P = 0.263)

		LMP1 no Inhs		LMP2 no Inhs		LMP1 with Inhs		LMP2 with Inhs	
**Symbol**	**Entrez Gene Name**	**p-value**	**Fold Change**	**p-value**	**Fold Change**	**p-value**	**Fold Change**	**p-value**	**Fold Change**
ACTR2	ARP2 actin-related protein 2 homolog (yeast)	3.55E-01	1.594	9.05E-01	-1.028	8.73E-01	-1.01	4.13E-02	-1.124
ITGA5	integrin, alpha 5 (fibronectin receptor, alpha polypeptide)	3.64E-01	-2.172	1.00E+00	-3.726	4.33E-02	-2.098	2.27E-02	-2.119
MYL6	myosin, light chain 6, alkali, smooth muscle and non-muscle	5.11E-02	1.436	4.10E-01	1.254	1.40E-02	1.973	9.73E-03	1.755

LMP1-no Inhibitors: Actin cytoskeleton (P = 0.135)

Symbol	Entrez Gene Name	LMP1 no Inhs p-value	Fold Change	LMP2 no Inhs p-value	Fold Change	LMP1 with Inhs p-value	Fold Change	LMP2 with Inhs p-value	Fold Change
ARPC4	actin related protein 2/3 complex, subunit 4, 20kDa	1.11E-02	-1.511	9.32E-01	-1.010	5.30E-01	-1.042	2.83E-01	1.177
CSK	c-src tyrosine kinase	5.95E-03	-1.902	3.88E-01	-1.572	1.36E-02	1.380	1.62E-01	-1.099
VCL	vinculin	4.67E-02	1.468	9.93E-01	-1.001	9.62E-02	-1.607	1.00E00	-1.583

LMP2-no Inhibitors: Actin cytoskeleton (P = 0.389)

Symbol	Entrez Gene Name	LMP1 no Inhs p-value	Fold Change	LMP2 no Inhs p-value	Fold Change	LMP1 with Inhs p-value	Fold Change	LMP2 with Inhs p-value	Fold Change
BAIAP2	BAI1-associated protein 2	9.89E-01	-1.006	3.81E-02	-1.488	1.71E-01	1.399	7.03E-01	-1.090

LMP1-with Inhibitors: Actin cytoskeleton (P = 9.58 x 10^{-6})

Symbol	Entrez Gene Name	LMP1 no Inhs p-value	Fold Change	LMP2 no Inhs p-value	Fold Change	LMP1 with Inhs p-value	Fold Change	LMP2 with Inhs p-value	Fold Change
ACTG1	actin, gamma 1	4.38E-01	2.212	9.31E-01	1.090	6.29E-03	1.182	1.00E00	-1.548
ACTN1	actinin, alpha 1	2.46E-01	1.725	7.05E-01	-1.250	1.22E-03	1.884	9.70E-01	1.079
ACTN4	actinin, alpha 4	3.46E-01	1.264	9.40E-01	1.023	3.67E-04	2.025	5.14E-01	1.040
ARPC1A	actin related protein 2/3 complex, subunit 1A, 41kDa	3.33E-01	1.922	7.89E-01	1.212	3.81E-02	1.382	2.37E-01	-1.175
CRKL	v-crk avian sarcoma virus CT10 oncogene homolog-like	7.18E-01	1.127	9.97E-01	1.001	3.25E-02	2.215	7.70E-01	1.132
CSK	c-src tyrosine kinase	5.95E-03	-1.902	3.88E-01	-1.572	1.36E-02	1.380	1.62E-01	-1.099
CYFIP2	cytoplasmic FMR1 interacting protein 2					4.71E-02	-1.275	1.42E-01	-3.469
EZR	ezrin	4.70E-01	1.533	6.72E-01	1.354	3.55E-02	2.326	9.76E-01	-1.012
FGD1	FYVE, RhoGEF and PH domain containing 1	1.00E00	0.000	1.00E00	0.000	1.73E-02	1.362	1.00E00	-1.038
GSN	gelsolin	4.22E-01	1.945	1.00E00	-1.706	2.18E-03	1.703	1.23E-01	-1.197
IQGAP3	IQ motif containing GTPase activating protein 3	1.00E00	0.000	1.00E00	0.000	8.66E-03	2.330	3.17E-01	-1.120
ITGA5	integrin, alpha 5 (fibronectin receptor, alpha polypeptide)	3.64E-01	-2.172	1.00E00	-3.726	4.33E-02	-2.098	2.27E-02	-2.119
MAP2K2	mitogen-activated protein kinase kinase 2	1.00E00	-1.030	1.00E00	1.079	2.57E-02	-1.397	2.02E-02	-1.306
MYL6	myosin, light chain 6, alkali, smooth muscle and non-muscle	5.11E-02	1.436	4.10E-01	1.254	1.40E-02	1.973	9.73E-03	1.755
MYL9	myosin, light chain 9, regulatory	1.00E00	0.000	1.00E00	0.000	4.54E-02	3.181	1.00E00	-1.094
PAK1	p21 protein (Cdc42/Rac)-activated kinase 1					3.34E-02	-1.594	1.00E00	
RAC2	ras-related C3 botulinum toxin substrate 2 (rho family, small GTP binding prot	1.00E00	0.000	1.00E00	0.000	1.04E-03	1.399	7.67E-02	-1.210
TMSB10/T	thymosin beta 10	2.63E-01	3.370	2.81E-01	1.836	3.77E-02	5.288	7.55E-01	-1.269
TTN	titin	1.00E00	0.000	1.00E00	0.000	1.49E-02	1.477	7.70E-02	1.501

LMP2-with Inhibitors: Actin cytoskeleton (P = 0.063)

Symbol	Entrez Gene Name	LMP1 no Inhs p-value	Fold Change	LMP2 no Inhs p-value	Fold Change	LMP1 with Inhs p-value	Fold Change	LMP2 with Inhs p-value	Fold Change
ACTR2	ARP2 actin-related protein 2 homolog (yeast)	3.55E-01	1.594	9.05E-01	-1.028	8.73E-01	-1.010	4.13E-02	-1.124
BRK1	BRICK1, SCAR/WAVE actin-nucleating complex subunit	1.00E00	1.672	1.00E00	0.000	4.45E-01	1.045	2.15E-02	-1.382
ITGA5	integrin, alpha 5 (fibronectin receptor, alpha polypeptide)	3.64E-01	-2.172	1.00E00	-3.726	4.33E-02	-2.098	2.27E-02	-2.119
MAP2K2	mitogen-activated protein kinase kinase 2	1.00E00	-1.030	1.00E00	1.079	2.57E-02	-1.397	2.02E-02	-1.306
MYL6	myosin, light chain 6, alkali, smooth muscle and non-muscle	5.11E-02	1.436	4.10E-01	1.254	1.40E-02	1.973	9.73E-03	1.755
NCKAP1	NCK-associated protein 1	5.12E-01	1.397	1.00E00	1.192	1.94E-01	-1.207	1.15E-02	-1.502
PXN	paxillin	2.26E-01	1.287	1.27E-01	-1.543	3.24E-01	1.546	1.68E-02	-1.271
TLN1	talin 1	6.90E-01	1.081	1.64E-01	-1.316	7.06E-02	1.063	2.74E-02	-1.154

		LMP1 no Inhs		LMP2 no Inhs		LMP1 with Inhs		LMP2 with Inhs	
	**LMP1-no Inhibitors: Binding of Filaments (P = 1)**	LMP1 no Inhs		LMP2 no Inhs		LMP1 with Inhs		LMP2 with Inhs	
none									
	**LMP2-no Inhibitors: Binding of Filaments (P = 1)**	LMP1 no Inhs		LMP2 no Inhs		LMP1 with Inhs		LMP2 with Inhs	
none									
	**LMP1-with Inhibitors: Binding of Filaments (P = 0.0093)**	LMP1 no Inhs		LMP2 no Inhs		LMP1 with Inhs		LMP2 with Inhs	
**Symbol**	**Entrez Gene Name**	**p-value**	**Fold Change**	**p-value**	**Fold Change**	**p-value**	**Fold Change**	**p-value**	**Fold Change**
CGN	cingulin	1.00E+00	0	1.00E+00	0	1.41E-04	28.752	9.66E-01	1.025
CSNK1D	casein kinase 1, delta	1.00E+00	0	1.00E+00	0	2.53E-02	-1.754	4.94E-02	-1.485
FHL1	four and a half LIM domains 1					2.10E-02	-2.88	3.44E-02	-1.783
MAP2	microtubule-associated protein 2	1.00E+00	0	1.00E+00	0	1.21E-03	14.588	1.00E+00	5.876
VIM	vimentin	9.23E-01	-1.058	7.32E-01	-1.226	9.94E-04	-1.74	2.98E-02	-1.433

	**LMP2-with Inhibitors: Binding of Filaments (P = 0.0132)**	LMP1 no Inhs		LMP2 no Inhs		LMP1 with Inhs		LMP2 with Inhs	
**Symbol**	**Entrez Gene Name**	**p-value**	**Fold Change**	**p-value**	**Fold Change**	**p-value**	**Fold Change**	**p-value**	**Fold Change**
CSNK1D	casein kinase 1, delta	1.00E+00	0	1.00E+00	0	2.53E-02	-1.754	4.94E-02	-1.485
FAS	Fas cell surface death receptor	1.00E+00	-1.102	1.00E+00	0	1.24E-01	-1.23	3.08E-03	-1.72
FHL1	four and a half LIM domains 1					2.10E-02	-2.88	3.44E-02	-1.783
VIM	vimentin	9.23E-01	-1.058	7.32E-01	-1.226	9.94E-04	-1.74	2.98E-02	-1.433

LMP1-no Inhibitors: Epithelial Adherens Junction signaling (P = 0.0538)

		LMP1 no Inhs		LMP2 no Inhs		LMP1 with Inhs		LMP2 with Inhs	
**Symbol**	**Entrez Gene Name**	**p-value**	**Fold Change**	**p-value**	**Fold Change**	**p-value**	**Fold Change**	**p-value**	**Fold Change**
ARPC4	actin related protein 2/3 complex, subunit 4, 20kDa	1.11E-02	-1.511	9.32E-01	-1.010	5.30E-01	-1.042	2.83E-01	1.177
RAP1B	RAP1B, member of RAS oncogene family	3.17E-02	-3.786	1.00E00	-3.401	8.56E-01	1.071	9.80E-01	1.010
VCL	vinculin	4.67E-02	1.468	9.93E-01	-1.001	9.62E-02	-1.607	1.00E00	-1.583

LMP2-no Inhibitors: Epithelial Adherens Junction signaling (P = 0.2805)

		LMP1 no Inhs		LMP2 no Inhs		LMP1 with Inhs		LMP2 with Inhs	
**Symbol**	**Entrez Gene Name**	**p-value**	**Fold Change**	**p-value**	**Fold Change**	**p-value**	**Fold Change**	**p-value**	**Fold Change**
BAIAP2	BAI1-associated protein 2	9.89E-01	-1.006	3.81E-02	-1.488	1.71E-01	1.399	7.03E-01	-1.090

LMP1-with Inhibitors: Epithelial Adherens Junction signaling (P = 4.976 x 10^{-5})

		LMP1 no Inhs		LMP2 no Inhs		LMP1 with Inhs		LMP2 with Inhs	
**Symbol**	**Entrez Gene Name**	**p-value**	**Fold Change**	**p-value**	**Fold Change**	**p-value**	**Fold Change**	**p-value**	**Fold Change**
ACTG1	actin, gamma 1	4.38E-01	2.212	9.31E-01	1.090	6.29E-03	1.182	1.00E00	-1.548
ACTN1	actinin, alpha 1	2.46E-01	1.725	7.05E-01	-1.250	1.22E-03	1.884	9.70E-01	1.079
ACTN4	actinin, alpha 4	3.46E-01	1.264	9.40E-01	1.023	3.67E-04	2.025	5.14E-01	1.040
ARPC1A	actin related protein 2/3 complex, subunit 1A, 41kDa	3.33E-01	1.922	7.89E-01	1.212	3.81E-02	1.382	2.37E-01	-1.175
CLINT1	clathrin interactor 1	1.00E00	1.298	1.00E00	-1.059	1.95E-02	2.029	3.86E-01	1.386
CLIP1	CAP-GLY domain containing linker protein 1	7.27E-01	1.138	8.90E-02	-2.449	4.59E-03	1.901	5.89E-01	1.072
KEAP1	kelch-like ECH-associated protein 1	1.00E-01	1.388	1.00E00	0.000	4.76E-02	1.310	1.49E-01	-1.211
LMO7	LIM domain 7	1.00E00	3.035			7.46E-05	3.556	1.71E-01	1.471
MET	MET proto-oncogene, receptor tyrosine kinase	1.00E00	0.000	1.00E00	0.000	4.99E-02	-1.277	6.91E-01	1.046
MLLT4	myeloid/lymphoid or mixed-lineage leukemia (trithorax homolog, Drosophila); t	9.93E-01	1.006	8.64E-01	1.126	5.67E-03	1.452	4.75E-01	1.126
MYL6	myosin, light chain 6, alkali, smooth muscle and non-muscle	5.11E-02	1.436	4.10E-01	1.254	1.40E-02	1.973	9.73E-03	1.755
MYL9	myosin, light chain 9, regulatory	1.00E00	0.000	1.00E00	0.000	4.54E-02	3.181	1.00E00	-1.094
PVRL1	poliovirus receptor-related 1 (herpesvirus entry mediator C)	1.00E00	2.603	1.00E00	3.312	4.31E-02	-2.987	1.00E00	1.056
TUBG1	tubulin, gamma 1	9.19E-01	-1.036	8.47E-01	1.068	4.40E-02	-1.277	5.92E-02	-1.250

LMP2-with Inhibitors: Epithelial Adherens Junction signaling (P = 0.5404)

		LMP1 no Inhs		LMP2 no Inhs		LMP1 with Inhs		LMP2 with Inhs	
**Symbol**	**Entrez Gene Name**	**p-value**	**Fold Change**	**p-value**	**Fold Change**	**p-value**	**Fold Change**	**p-value**	**Fold Change**
ACTR2	ARP2 actin-related protein 2 homolog (yeast)	3.55E-01	1.594	9.05E-01	-1.028	8.73E-01	-1.010	4.13E-02	-1.124
EPN3	epsin 3					1.73E-01	1.129	2.05E-02	-1.594
MYL6	myosin, light chain 6, alkali, smooth muscle and non-muscle	5.11E-02	1.436	4.10E-01	1.254	1.40E-02	1.973	9.73E-03	1.755

LMP1-no Inhibitors: Sertoli Cell-Sertoli Cell Junction signaling (P = 1)

None

LMP2-no Inhibitors: Sertoli Cell-Sertoli Cell Junction signaling (P = 0.339)

		LMP1 no Inhs		LMP2 no Inhs		LMP1 with Inhs		LMP2 with Inhs	
**Symbol**	**Entrez Gene Name**	**p-value(A1)**	**Fold Change(A**	**p-value(A2#)**	**Fold Change(A**	**p-value(A3)**	**Fold Change(A**	**p-value(A4)**	**Fold Change(A4)**
SPTBN1	spectrin, beta, non-erythrocytic 1	2.24E-01	-1.138	2.01E-02	-1.613	7.98E-03	1.407	7.00E-01	-1.251

LMP1-with Inhibitors: Sertoli Cell-Sertoli Cell Junction signaling (P = 3.15 x 10^{-6})

		LMP1 no Inhs		LMP2 no Inhs		LMP1 with Inhs		LMP2 with Inhs	
**Symbol**	**Entrez Gene Name**	**p-value(A1)**	**Fold Change(A**	**p-value(A2)**	**Fold Change(A**	**p-value(A3#)**	**Fold Change(A**	**p-value(A4)**	**Fold Change(A4)**
ACTG1	actin, gamma 1	4.38E-01	2.212	9.31E-01	1.090	6.29E-03	1.182	1.00E00	-1.548
ACTN1	actinin, alpha 1	2.46E-01	1.725	7.05E-01	-1.250	1.22E-03	1.884	9.70E-01	1.079
ACTN4	actinin, alpha 4	3.46E-01	1.264	9.40E-01	1.023	3.67E-04	2.025	5.14E-01	1.040
CGN	cingulin	1.00E00	0.000	1.00E00	0.000	1.41E-04	28.752	9.66E-01	1.025
CLINT1	clathrin interactor 1	1.00E00	1.298	1.00E00	-1.059	1.95E-02	2.029	3.86E-01	1.386
ITGA5	integrin, alpha 5 (fibronectin receptor, alpha polypeptide)	3.64E-01	-2.172	1.00E00	-3.726	4.33E-02	-2.098	2.27E-02	-2.119
KEAP1	kelch-like ECH-associated protein 1	1.00E-01	1.388	1.00E00	0.000	4.76E-02	1.310	1.49E-01	-1.211
MAP2K2	mitogen-activated protein kinase kinase 2	1.00E00	-1.030	1.00E00	1.079	2.57E-02	-1.397	2.02E-02	-1.306
MLLT4	myeloid/lymphoid or mixed-lineage leukemia (trithorax homolog, Drosophila); t	9.93E-01	1.006	8.64E-01	1.126	5.67E-03	1.452	4.75E-01	1.126
PVRL1	poliovirus receptor-related 1 (herpesvirus entry mediator C)	1.00E00	2.603	1.00E00	3.312	4.31E-02	-2.987	1.00E00	1.056
RAB8B	RAB8B, member RAS oncogene family	1.00E00	0.000	1.00E00	0.000	3.45E-02	1.901	9.61E-01	1.014
SPTAN1	spectrin, alpha, non-erythrocytic 1	5.03E-01	1.440	7.50E-01	1.140	8.59E-03	-2.105	5.65E-01	1.132
SPTBN1	spectrin, beta, non-erythrocytic 1	2.24E-01	-1.138	2.01E-02	-1.613	7.98E-03	1.407	7.00E-01	-1.251
TJP1	tight junction protein 1	2.00E-01	1.561	7.48E-01	-1.153	2.54E-02	1.457	6.26E-01	-1.098
TJP2	tight junction protein 2	9.86E-01	-1.004	3.42E-01	-1.502	1.24E-02	1.377	1.70E-01	1.164
TJP3	tight junction protein 3					4.53E-02	2.144	5.41E-01	1.335
TUBG1	tubulin, gamma 1	9.19E-01	-1.036	8.47E-01	1.068	4.40E-02	-1.277	5.92E-02	-1.250
YBX3	Y box binding protein 3	6.81E-01	1.320	4.39E-01	-1.621	3.77E-02	-1.300	1.55E-02	-1.410

LMP2-with Inhibitors: Sertoli Cell-Sertoli Cell Junction signaling (P = 0.477)

		LMP1 no Inhs		LMP2 no Inhs		LMP1 with Inhs		LMP2 with Inhs	
**Symbol**	**Entrez Gene Name**	**p-value(A1)**	**Fold Change(A**	**p-value(A2)**	**Fold Change(A**	**p-value(A3)**	**Fold Change(A**	**p-value(A4#)**	**Fold Change(A4#)**
EPN3	epsin 3					1.73E-01	1.129	2.05E-02	-1.594
ITGA5	integrin, alpha 5 (fibronectin receptor, alpha polypeptide)	3.64E-01	-2.172	1.00E00	-3.726	4.33E-02	-2.098	2.27E-02	-2.119
MAP2K2	mitogen-activated protein kinase kinase 2	1.00E00	-1.030	1.00E00	1.079	2.57E-02	-1.397	2.02E-02	-1.306
YBX3	Y box binding protein 3	6.81E-01	1.320	4.39E-01	-1.621	3.77E-02	-1.300	1.55E-02	-1.410

LMP1-no Inhibitors: Remodeling of Epithelial Adherens Junctions (P = 0.0583)

		LMP1 no Inhs		LMP2 no Inhs		LMP1 with Inhs		LMP2 with Inhs	
**Symbol**	**Entrez Gene Name**	**p-value**	**Fold Change**	**p-value**	**Fold Change**	**p-value**	**Fold Change**	**p-value**	**Fold Change**
ARPC4	actin related protein 2/3 complex, subunit 4, 20kDa	1.11E-02	-1.511	9.32E-01	-1.01	5.30E-01	-1.042	2.83E-01	1.177
VCL	vinculin	4.67E-02	1.468	9.93E-01	-1.001	9.62E-02	-1.607	1.00E+00	-1.583

LMP2-no Inhibitors: Remodeling of Epithelial Adherens Junctions (P = 0.14)

		LMP1 no Inhs		LMP2 no Inhs		LMP1 with Inhs		LMP2 with Inhs	
**Symbol**	**Entrez Gene Name**	**p-value**	**Fold Change**	**p-value**	**Fold Change**	**p-value**	**Fold Change**	**p-value**	**Fold Change**
RAB7A	RAB7A, member RAS oncogene family	8.84E-02	-1.314	4.01E-02	-1.687	4.68E-02	-1.305	3.69E-02	-1.902

LMP1-with Inhibitors: Remodeling of Epithelial Adherens Junctions (P = 0.00048)

		LMP1 no Inhs		LMP2 no Inhs		LMP1 with Inhs		LMP2 with Inhs	
**Symbol**	**Entrez Gene Name**	**p-value**	**Fold Change**	**p-value**	**Fold Change**	**p-value**	**Fold Change**	**p-value**	**Fold Change**
ACTG1	actin, gamma 1	4.38E-01	2.212	9.31E-01	1.09	6.29E-03	1.182	1.00E+00	-1.548
ACTN1	actinin, alpha 1	2.46E-01	1.725	7.05E-01	-1.25	1.22E-03	1.884	9.70E-01	1.079
ACTN4	actinin, alpha 4	3.46E-01	1.264	9.40E-01	1.023	3.67E-04	2.025	5.14E-01	1.04
ARPC1A	actin related protein 2/3 complex, subunit 1A, 41kDa	3.33E-01	1.922	7.89E-01	1.212	3.81E-02	1.382	2.37E-01	-1.175
CLIP1	CAP-GLY domain containing linker protein 1	7.27E-01	1.138	8.90E-02	-2.449	4.59E-03	1.901	5.89E-01	1.072
MET	MET proto-oncogene, receptor tyrosine kinase	1.00E+00	0	1.00E+00	0	4.99E-02	-1.277	6.91E-01	1.046
RAB7A	RAB7A, member RAS oncogene family	8.84E-02	-1.314	4.01E-02	-1.687	4.68E-02	-1.305	3.69E-02	-1.902
TUBG1	tubulin, gamma 1	9.19E-01	-1.036	8.47E-01	1.068	4.40E-02	-1.277	5.92E-02	-1.25

LMP2-with Inhibitors: Remodeling of Epithelial Adherens Junctions (P = 0.3746)

		LMP1 no Inhs		LMP2 no Inhs		LMP1 with Inhs		LMP2 with Inhs	
**Symbol**	**Entrez Gene Name**	**p-value**	**Fold Change**	**p-value**	**Fold Change**	**p-value**	**Fold Change**	**p-value**	**Fold Change**
ACTR2	ARP2 actin-related protein 2 homolog (yeast)	3.55E-01	1.594	9.05E-01	-1.028	8.73E-01	-1.01	4.13E-02	-1.124
RAB7A	RAB7A, member RAS oncogene family	8.84E-02	-1.314	4.01E-02	-1.687	4.68E-02	-1.305	3.69E-02	-1.902

LMP1-no Inhibitors: Tight Junction signaling (P = 0.620)

		LMP1 no Inhs		LMP2 no Inhs		LMP1 with Inhs		LMP2 with Inhs	
Symbol	Entrez Gene Name	p-value	Fold Change	p-value	Fold Change	p-value	Fold Change	p-value	Fold Change
VCL	vinculin	4.67E-02	1.468	9.93E-01	-1.001	9.62E-02	-1.607	1.00E+00	-1.583

LMP2-no Inhibitors: Tight Junction signaling (P = 0.0528)

		LMP1 no Inhs		LMP2 no Inhs		LMP1 with Inhs		LMP2 with Inhs	
Symbol	Entrez Gene Name	p-value	Fold Change	p-value	Fold Change	p-value	Fold Change	p-value	Fold Change
CPSF6	cleavage and polyadenylation specific factor 6, 68kDa	4.86E-01	-1.65	1.63E-03	1.269	9.69E-01	1.014	8.00E-01	-1.077
VAMP3	vesicle-associated membrane protein 3	5.35E-01	-1.358	1.14E-03	-1.687	2.83E-01	-1.167	5.43E-02	1.295

LMP1-with Inhibitors: Tight Junction signaling (P = 0.00062)

		LMP1 no Inhs		LMP2 no Inhs		LMP1 with Inhs		LMP2 with Inhs	
Symbol	Entrez Gene Name	p-value	Fold Change	p-value	Fold Change	p-value	Fold Change	p-value	Fold Change
ACTG1	actin, gamma 1	4.38E-01	2.212	9.31E-01	1.09	6.29E-03	1.182	1.00E+00	-1.548
CGN	cingulin	1.00E+00	0	1.00E+00	0	1.41E-04	28.752	9.66E-01	1.025
CPSF3	cleavage and polyadenylation specific factor 3, 73kDa	2.10E-01	-1.563	4.60E-01	-1.418	3.02E-02	-1.601	1.42E-01	-1.175
CSTF2	cleavage stimulation factor, 3' pre-RNA, subunit 2, 64kDa	7.34E-01	-1.161	7.83E-01	-1.222	1.17E-02	-1.791	2.56E-01	-1.242
MLLT4	myeloid/lymphoid or mixed-lineage leukemia (trithorax homolog, Drosophila); translocatec	9.93E-01	1.006	8.64E-01	1.126	5.67E-03	1.452	4.75E-01	1.126
MYL6	myosin, light chain 6, alkali, smooth muscle and non-muscle	5.11E-02	1.436	4.10E-01	1.254	1.40E-02	1.973	9.73E-03	1.755
MYL9	myosin, light chain 9, regulatory	1.00E+00	0	1.00E+00	0	4.54E-02	3.181	1.00E+00	-1.094
PVRL1	poliovirus receptor-related 1 (herpesvirus entry mediator C)	1.00E+00	2.603	1.00E+00	3.312	4.31E-02	-2.987	1.00E+00	1.056
SPTAN1	spectrin, alpha, non-erythrocytic 1	5.03E-01	1.44	7.50E-01	1.14	8.59E-03	-2.105	5.65E-01	1.132
TJP1	tight junction protein 1	2.00E-01	1.561	7.48E-01	-1.153	2.54E-02	1.457	6.26E-01	-1.098
TJP2	tight junction protein 2	9.86E-01	-1.004	3.42E-01	-1.502	1.24E-02	1.377	1.70E-01	1.164
TJP3	tight junction protein 3					4.53E-02	2.144	5.41E-01	1.335
YBX3	Y box binding protein 3	6.81E-01	1.32	4.39E-01	-1.621	3.77E-02	-1.3	1.55E-02	-1.41

LMP2-with Inhibitors: Tight Junction signaling (P = 0.2164)

		LMP1 no Inhs		LMP2 no Inhs		LMP1 with Inhs		LMP2 with Inhs	
Symbol	Entrez Gene Name	p-value	Fold Change	p-value	Fold Change	p-value	Fold Change	p-value	Fold Change
GOSR1	golgi SNAP receptor complex member 1	1.00E+00	1.325	1.00E+00	0	3.79E-01	-1.545	9.61E-03	-3.951
MYL6	myosin, light chain 6, alkali, smooth muscle and non-muscle	5.11E-02	1.436	4.10E-01	1.254	1.40E-02	1.973	9.73E-03	1.755
NFKB1	nuclear factor of kappa light polypeptide gene enhancer in B-cells 1	7.54E-01	-1.28	8.35E-01	1.177	9.42E-02	1.205	1.63E-02	1.226
PPP2R2A	protein phosphatase 2, regulatory subunit B, alpha	4.81E-01	1.459	8.73E-01	1.147	2.85E-01	-1.232	7.48E-03	-1.358
YBX3	Y box binding protein 3	6.81E-01	1.32	4.39E-01	-1.621	3.77E-02	-1.3	1.55E-02	-1.41
