# Supplementary material for: Global Proteomic Changes Induced by the Epstein-Barr Virus Oncoproteins Latent Membrane Protein 1 and 2A
Source: mBio. 2018 Jun 19;9(3):e00959-18. doi: 10.1128/mBio.00959-18 (PMC6016245; doi:10.1128/mBio.00959-18)
Supplement: TABLE S5 [file mbo003183928st5.pdf]

Supplemental Table-5

Symbol	LMP1-no Inhibitors: Proliferation of Cells (P = 1.89 x 10^{-5}) Entrez Gene Name	LMP1 no Inhs p-value	Fold Change	LMP2 no Inhs p-value	Fold Change	LMP1 with Inhs p-value	Fold Change	LMP2 with Inhs p-value	Fold Change
AARS	alanyl-tRNA synthetase	1.92E-02	-1.264	8.69E-01	-1.099	8.53E-03	-1.189	2.05E-01	1.093
ACACA	acetyl-CoA carboxylase alpha	1.81E-02	-2.870	2.91E-01	-1.911	8.33E-01	1.051	9.42E-01	-1.014
CALD1	caldesmon 1	4.76E-03	2.401	5.94E-01	1.512	2.10E-03	3.792	8.07E-01	-1.175
CAPN1	calpain 1, (mu/l) large subunit	4.38E-02	-1.239	9.13E-01	1.017	1.09E-01	1.277	8.11E-01	1.037
CAPNS1	calpain, small subunit 1	1.07E-02	1.386	1.35E-01	1.316	2.97E-01	1.078	1.28E-01	-1.125
CENPF	centromere protein F, 350/400kDa	3.29E-02	-2.717	1.00E00	-1.196	8.97E-02	5.906	1.00E00	3.202
CNN2	calponin 2	3.14E-02	1.550	3.23E-01	1.388	8.13E-04	1.753	6.26E-02	1.246
CNP	2',3'-cyclic nucleotide 3' phosphodiesterase	1.12E-03	2.459	1.57E-01	2.946	3.90E-01	1.116	7.81E-01	1.035
CSK	c-src tyrosine kinase	5.95E-03	-1.902	3.88E-01	-1.572	1.36E-02	1.380	1.62E-01	-1.099
DSP	desmoplakin	1.42E-02	1.418	6.00E-01	1.081	7.88E-01	1.044	8.70E-01	1.019
EHD4	EH-domain containing 4	3.96E-02	-1.276	2.22E-02	-1.300	1.77E-01	-1.137	6.55E-01	-1.055
EIF6	eukaryotic translation initiation factor 6	4.67E-02	1.734	5.51E-01	1.093	6.08E-01	-1.138	6.49E-01	-1.115
EIF2AK2	eukaryotic translation initiation factor 2-alpha kinase 2	3.04E-02	-1.756	5.90E-01	1.081	5.77E-01	-1.088	3.84E-01	-1.125
EIF4A1	eukaryotic translation initiation factor 4A1	2.22E-02	-1.494	6.00E-01	-1.108	5.36E-03	-1.474	2.21E-03	-1.518
ETFB	electron-transfer-flavoprotein, beta polypeptide	4.27E-02	1.162	3.16E-01	1.041	1.85E-01	-1.093	1.00E00	818.746
FAH	fumarylacetoacetate hydrolase (fumarylacetoacetase)	4.01E-02	-1.640	9.24E-01	-1.035	9.52E-01	1.046	7.64E-01	-1.253
FERMT1	fermitin family member 1	4.37E-02	1.591	1.00E00	0.000	2.18E-01	-1.197	2.25E-01	-1.175
FLOT2	flotillin 2	3.55E-02	-1.664	3.63E-01	-2.093	6.31E-01	1.078	9.33E-01	1.015
FTO	fat mass and obesity associated	1.66E-02	-1.632	1.00E00	-1.286	1.40E-01	1.294	9.58E-01	-1.014
GTPBP4	GTP binding protein 4	3.60E-02	-1.865	1.48E-01	-1.236	7.49E-01	-1.158	4.71E-01	-1.345
HK1	hexokinase 1	3.25E-03	-1.363	2.59E-01	-1.360	5.00E-02	-1.264	8.03E-01	1.025
HMGB3	high mobility group box 3	1.91E-02	-1.541	1.01E-01	-2.475	4.81E-01	1.394	3.92E-01	-1.465
HNRNPAB	heterogeneous nuclear ribonucleoprotein A/B	3.29E-02	-1.223	5.36E-01	1.118	1.53E-01	-1.147	1.58E-01	-1.152
HNRNPD	heterogeneous nuclear ribonucleoprotein D (AU-rich element RNA binding protein	1.54E-03	-1.454	2.69E-01	-1.169	6.70E-01	2.011	5.09E-01	2.793
HSPB1	heat shock 27kDa protein 1	1.20E-02	3.427	2.30E-01	1.936	2.29E-01	1.449	9.29E-01	-1.043
ILF2	interleukin enhancer binding factor 2	4.35E-02	-1.524	7.87E-01	1.098	5.75E-01	-1.092	8.51E-01	-1.031
LAMB1	laminin, beta 1	2.92E-02	-2.479	1.00E00	1.431	1.80E-02	-1.475	2.43E-01	-1.522
LAMC1	laminin, gamma 1 (formerly LAMB2)	3.17E-02	-1.770	7.30E-02	-2.375	6.76E-01	-1.543	7.57E-01	-1.306
MTHFD1	methylenetetrahydrofolate dehydrogenase (NADP+ dependent) 1, methenyltetrahy	8.02E-03	-1.311	2.64E-01	-1.172	1.29E-03	-1.305	3.85E-01	-1.071
MVP	major vault protein	1.50E-02	1.448	5.34E-01	-1.110	3.14E-04	1.512	1.29E-01	-1.259
MYBBP1A	MYB binding protein (P160) 1a	1.16E-02	-1.524	5.32E-01	-1.070	3.38E-02	-1.329	5.27E-02	-1.161
NDRG1	N-myc downstream regulated 1	1.89E-04	2.066	3.24E-02	2.279	2.95E-01	-1.325	1.22E-01	1.474
PCNA	proliferating cell nuclear antigen	1.39E-02	-1.567	8.60E-01	-1.042	1.07E-01	-1.523	8.93E-02	-1.434
PGK1	phosphoglycerate kinase 1	2.51E-02	-1.177	5.67E-01	-1.100	4.53E-01	-1.196	9.56E-01	-1.011
PHB	prohibitin	2.29E-02	1.435	2.84E-01	1.437	7.44E-01	1.094	4.09E-01	1.192
PRKCDBP	protein kinase C, delta binding protein	2.12E-02	2.481	3.25E-01	2.001	3.10E-01	2.533	6.45E-01	1.506
PRMT1	protein arginine methyltransferase 1	3.87E-02	-1.653	7.42E-01	-1.151	5.54E-01	-1.430	7.56E-01	-1.140
PSMD2	proteasome (prosome, macropain) 26S subunit, non-ATPase, 2	5.38E-03	1.171	1.21E-01	1.472	2.87E-01	-1.067	2.70E-02	-1.227
RAB1A	RAB1A, member RAS oncogene family	2.65E-02	1.585	5.74E-01	-1.775	6.03E-01	-1.222	5.66E-01	-1.260
RAP1B	RAP1B, member of RAS oncogene family	3.17E-02	-3.786	1.00E00	-3.401	8.56E-01	1.071	9.80E-01	1.010
SART1	squamous cell carcinoma antigen recognized by T cells	3.31E-02	1.731	8.46E-01	-1.160	6.71E-01	1.075	1.65E-01	1.212
SERPINB5	serpin peptidase inhibitor, clade B (ovalbumin), member 5	1.15E-02	1.984	9.97E-02	1.719	5.35E-01	1.261	5.81E-01	1.299
SFN	stratifin	3.53E-02	2.029	2.01E-01	1.754	2.44E-01	-1.200	6.46E-01	-1.123
SRSF5	serine/arginine-rich splicing factor 5	1.02E-02	1.674	2.60E-02	1.289	5.90E-01	-1.144	7.75E-01	1.067

THOC1	THO complex 1	7.01E-03	-2.126	1.00E00	-2.396	3.57E-01	-1.181	8.65E-01	1.021
TOP1	topoisomerase (DNA) I	4.71E-02	-1.434	6.68E-01	-1.064	2.27E-01	-1.276	9.74E-01	1.005
TPD52L1	tumor protein D52-like 1	4.74E-02	1.692	3.73E-01	1.739	3.92E-02	2.140	8.26E-01	1.079
TXN	thioredoxin	1.16E-02	1.420	2.42E-01	1.211	9.26E-02	1.313	5.66E-01	1.169
USP5	ubiquitin specific peptidase 5 (isopeptidase T)	2.61E-02	-1.490	2.34E-01	1.187	2.52E-01	-1.063	6.03E-02	-1.150
VCL	vinculin	4.67E-02	1.468	9.93E-01	-1.001	9.62E-02	-1.607	1.00E00	-1.583
XRCC1	X-ray repair complementing defective repair in Chinese hamster cells 1	1.26E-02	-5.060	1.00E00	0.000	8.02E-01	1.055	8.55E-01	1.061

	LMP2-no Inhibitors: Proliferation of Cells (P = 0.00195)	LMP1 no Inhs		LMP2 no Inhs		LMP1 with Inhs		LMP2 with Inhs	
**Symbol**	**Entrez Gene Name**	**p-value**	**Fold Change**	**p-value**	**Fold Change**	**p-value**	**Fold Change**	**p-value**	**Fold Change**
BAIAP2	BAI1-associated protein 2	9.89E-01	-1.006	3.81E-02	-1.488	1.71E-01	1.399	7.03E-01	-1.090
BYSL	bystin-like	1.00E00	-1.533	3.65E-02	-1.628	2.67E-01	1.290	9.19E-02	1.381
CWC27	CWC27 spliceosome-associated protein homolog (S. cerevisiae)	7.64E-01	-1.078	1.98E-02	2.835	7.88E-01	-1.172	8.24E-01	-1.106
EHD4	EH-domain containing 4	3.96E-02	-1.276	2.22E-02	-1.300	1.77E-01	-1.137	6.55E-01	-1.055
FABP5	fatty acid binding protein 5 (psoriasis-associated)	8.16E-01	-1.168	4.48E-02	-1.493	8.47E-01	1.072	3.98E-01	-1.361
HNRNPA1	heterogeneous nuclear ribonucleoprotein A1	1.89E-01	-1.175	4.40E-02	1.292	1.79E-01	-1.311	4.20E-01	-1.220
IGF2BP3	insulin-like growth factor 2 mRNA binding protein 3	7.30E-01	1.216	3.56E-03	-1.963	5.05E-01	-1.102	1.29E-02	-2.325
LAP3	leucine aminopeptidase 3	2.18E-01	-1.088	1.94E-02	1.487	7.39E-02	1.138	8.98E-01	-1.012
MYOF	myoferlin	7.11E-01	-1.120	1.89E-02	-2.038	6.03E-02	1.643	2.61E-01	-1.391
NDRG1	N-myc downstream regulated 1	1.89E-04	2.066	3.24E-02	2.279	2.95E-01	-1.325	1.22E-01	1.474
PALLD	palladin, cytoskeletal associated protein	4.62E-01	1.576	3.76E-02	-2.936	1.09E-01	1.526	5.44E-01	-1.088
PML	promyelocytic leukemia	4.01E-01	-1.174	2.33E-02	-2.367	7.11E-01	1.114	4.66E-02	-1.449
PNPT1	polyribonucleotide nucleotidyltransferase 1	1.96E-01	-1.028	6.97E-03	1.380	6.08E-01	-1.064	2.69E-01	1.150
PRDX2	peroxiredoxin 2	1.52E-01	1.178	3.39E-02	1.213	6.38E-01	1.268	6.44E-01	1.294
RPS25	ribosomal protein S25	1.52E-01	1.140	3.99E-02	1.423	8.54E-01	-1.043	4.58E-01	-1.207
S100A4	S100 calcium binding protein A4	9.45E-01	1.096	3.90E-03	7.360	3.00E-02	-1.797	1.74E-01	1.771
SERPINH1	serpin peptidase inhibitor, clade H (heat shock protein 47), member 1, (collagen bi	1.60E-01	-1.194	3.32E-02	1.173	5.04E-01	-1.226	3.12E-01	-1.246
SPTBN1	spectrin, beta, non-erythrocytic 1	2.24E-01	-1.138	2.01E-02	-1.613	7.98E-03	1.407	7.00E-01	-1.251
SRSF5	serine/arginine-rich splicing factor 5	1.02E-02	1.674	2.60E-02	1.289	5.90E-01	-1.144	7.75E-01	1.067
TPP2	tripeptidyl peptidase II	2.42E-01	-2.510	4.73E-02	-1.323	1.41E-01	-1.243	8.26E-01	1.019
TXNDC5	thioredoxin domain containing 5 (endoplasmic reticulum)	5.44E-02	-1.447	5.42E-03	-1.371	6.94E-01	-1.045	9.38E-01	-1.008

	LMP1-with Inhibitors: Proliferation of Cells (P = 2.05 x 10^{-10})	LMP1 no Inhs		LMP2 no Inhs		LMP1 with Inhs		LMP2 with Inhs	
**Symbol**	**Entrez Gene Name**	**p-value**	**Fold Change**	**p-value**	**Fold Change**	**p-value**	**Fold Change**	**p-value**	**Fold Change**
AARS	alanyl-tRNA synthetase	1.92E-02	-1.264	8.69E-01	-1.099	8.53E-03	-1.189	2.05E-01	1.093
AATF	apoptosis antagonizing transcription factor	3.12E-01	-1.370	4.76E-01	1.207	3.10E-02	-1.210	4.32E-01	-1.074
ACLY	ATP citrate lyase	3.01E-01	-1.500	9.17E-01	1.032	3.15E-02	1.159	3.79E-02	1.167
ACP1	acid phosphatase 1, soluble	3.10E-01	-1.434	3.69E-01	-1.690	6.27E-03	-1.337	8.63E-01	1.012
ACTG1	actin, gamma 1	4.38E-01	2.212	9.31E-01	1.090	6.29E-03	1.182	1.00E00	-1.548
ACTN1	actinin, alpha 1	2.46E-01	1.725	7.05E-01	-1.250	1.22E-03	1.884	9.70E-01	1.079
ACTN4	actinin, alpha 4	3.46E-01	1.264	9.40E-01	1.023	3.67E-04	2.025	5.14E-01	1.040
ADA	adenosine deaminase	1.00E00	1.395	1.00E00	1.473	4.25E-02	2.018	2.17E-01	-1.495
ADK	adenosine kinase	5.55E-01	1.209	1.50E-01	1.657	1.68E-02	-1.327	7.69E-02	-1.153
AGO2	argonaute RISC catalytic component 2	1.00E00	1.578	1.00E00	1.280	1.64E-02	-1.539	1.37E-01	-1.335
AMD1	adenosylmethionine decarboxylase 1	1.00E00	0.000	1.00E00	0.000	1.12E-02	-1.933	1.00E00	
ANXA1	annexin A1	1.02E-01	1.681	1.66E-01	1.297	1.25E-02	1.272	7.77E-01	-1.021
ANXA2	annexin A2	6.69E-01	1.181	1.65E-01	1.204	1.96E-02	1.394	2.54E-01	1.175
ANXA11	annexin A11	9.61E-01	-1.021	8.77E-01	1.084	7.01E-03	1.739	1.27E-01	1.222
ARHGEF11	Rho guanine nucleotide exchange factor (GEF) 11	1.00E00	0.000	1.00E00	0.000	1.83E-02	1.612	6.07E-01	-1.132

ATP6V0D1	ATPase, H+ transporting, lysosomal 38kDa, V0 subunit d1	1.00E00	0.000	1.00E00	0.000	3.25E-02	1.934	4.54E-02	1.720
BAG1	BCL2-associated athanogene	1.00E00	0.000	1.00E00	0.000	2.96E-03	2.613	9.49E-01	1.028
BCLAF1	BCL2-associated transcription factor 1	2.45E-01	1.432	8.27E-01	-1.070	1.38E-02	-1.654	8.10E-01	1.037
BIN3	bridging integrator 3	1.00E00	0.000	1.00E00	0.000	1.01E-02	2.156	9.13E-01	1.033
BIRC3	baculoviral IAP repeat containing 3	1.00E00	0.000	1.00E00	0.000	1.58E-02	-1.760	6.50E-01	-1.027
C9orf78	chromosome 9 open reading frame 78	1.00E00	0.000	1.00E00	0.000	4.73E-02	-1.681	1.18E-02	-2.795
CALD1	caldesmon 1	4.76E-03	2.401	5.94E-01	1.512	2.10E-03	3.792	8.07E-01	-1.175
CASP1	caspase 1, apoptosis-related cysteine peptidase	1.00E00	1.079	1.57E-01	-1.776	3.91E-02	1.376	4.70E-01	-1.299
CAST	calpastatin	5.69E-01	1.357	1.59E-01	2.535	6.79E-03	1.556	2.02E-01	1.279
CBX1	chromobox homolog 1	1.00E00	-2.276	1.00E00	1.612	2.66E-02	-1.502	2.12E-01	-1.267
CCDC88A	coiled-coil domain containing 88A	1.00E00	0.000	1.00E00	0.000	9.06E-03	-1.440	9.99E-01	1.000
CD44	CD44 molecule (Indian blood group)	4.40E-01	-2.277	1.34E-01	-3.127	5.16E-03	-1.316	4.93E-01	-1.089
CD99	CD99 molecule	1.00E00	0.000	1.00E00	0.000	1.94E-02	2.356	2.42E-01	1.329
CDK5	cyclin-dependent kinase 5	1.00E00	0.000	1.00E00	0.000	1.64E-02	-1.426	2.70E-01	1.029
CDK2AP1	cyclin-dependent kinase 2 associated protein 1	1.00E00	0.000	1.00E00	0.000	4.59E-03	-2.260	7.60E-01	-1.022
CFDP1	craniofacial development protein 1	1.00E00	0.000	1.00E00	0.000	4.08E-02	2.017	4.03E-01	1.514
CGREF1	cell growth regulator with EF-hand domain 1					2.30E-02	-1.165	3.49E-01	-1.542
CIB1	calcium and integrin binding 1 (calmyrin)					1.50E-02	3.427	1.00E00	1.468
CLIP1	CAP-GLY domain containing linker protein 1	7.27E-01	1.138	8.90E-02	-2.449	4.59E-03	1.901	5.89E-01	1.072
CNN2	calponin 2	3.14E-02	1.550	3.23E-01	1.388	8.13E-04	1.753	6.26E-02	1.246
CNPY2	canopy FGF signaling regulator 2	3.30E-01	-1.142	1.91E-01	-1.227	4.87E-02	1.708	8.21E-02	1.679
COPS6	COP9 signalosome subunit 6	1.00E00	0.000	1.00E00	0.000	5.68E-03	-1.935	3.01E-05	-2.304
CRABP2	cellular retinoic acid binding protein 2	1.00E00	0.000	1.00E00	0.000	2.01E-02	10.196	5.80E-01	1.753
CRKL	v-crk avian sarcoma virus CT10 oncogene homolog-like	7.18E-01	1.127	9.97E-01	1.001	3.25E-02	2.215	7.70E-01	1.132
CSK	c-src tyrosine kinase	5.95E-03	-1.902	3.88E-01	-1.572	1.36E-02	1.380	1.62E-01	-1.099
CSNK1D	casein kinase 1, delta	1.00E00	0.000	1.00E00	0.000	2.53E-02	-1.754	4.94E-02	-1.485
CSTF2	cleavage stimulation factor, 3' pre-RNA, subunit 2, 64kDa	7.34E-01	-1.161	7.83E-01	-1.222	1.17E-02	-1.791	2.56E-01	-1.242
CTBP1	C-terminal binding protein 1	5.49E-01	-1.175	5.46E-01	1.181	3.58E-02	-1.525	2.73E-01	-1.224
CTSB	cathepsin B	9.68E-02	1.528	9.13E-01	1.055	1.55E-02	1.920	1.58E-01	1.368
CUL1	cullin 1	1.00E00	-3.027	1.00E00	2.030	3.57E-03	-1.414	9.59E-01	-1.002
DCTN2	dynactin 2 (p50)	3.07E-01	1.260	6.56E-01	1.180	7.02E-07	1.417	7.76E-02	-1.068
DDX58	DEAD (Asp-Glu-Ala-Asp) box polypeptide 58					2.50E-02	4.906	9.74E-01	-1.016
DPYSL2	dihydropyrimidinase-like 2	3.59E-01	-1.275	3.09E-01	-1.991	2.49E-03	1.351	1.60E-01	1.083
ECSIT	ECSIT signalling integrator	1.00E00	0.000	1.00E00	0.000	1.41E-02	-2.365	1.77E-02	-2.634
EIF3B	eukaryotic translation initiation factor 3, subunit B	3.42E-01	-1.386	8.48E-01	1.074	1.02E-02	-1.676	2.12E-03	-1.435
EIF3C	eukaryotic translation initiation factor 3, subunit C	5.73E-01	-1.271	7.02E-01	-1.177	9.18E-03	-1.354	1.15E-01	-1.170
EIF3E	eukaryotic translation initiation factor 3, subunit E	2.79E-01	1.270	5.54E-01	-1.191	4.88E-02	-1.981	2.96E-02	-1.483
EIF4A1	eukaryotic translation initiation factor 4A1	2.22E-02	-1.494	6.00E-01	-1.108	5.36E-03	-1.474	2.21E-03	-1.518
EIF4E3	eukaryotic translation initiation factor 4E family member 3					4.31E-02	1.453	1.11E-01	-1.372
ELMO2	engulfment and cell motility 2	1.00E00	0.000	1.00E00	0.000	3.89E-02	1.267	5.69E-01	-1.392
ENO1	enolase 1, (alpha)	3.39E-01	1.109	1.62E-01	1.484	6.46E-03	-2.532	8.54E-03	-2.414
EP400	E1A binding protein p400	1.00E00	0.000	1.00E00	0.000	5.27E-03	1.258	6.43E-01	-1.248
ERBB2IP	erbb2 interacting protein	1.00E00	0.000	1.00E00	1.288	4.08E-02	3.980	1.52E-01	2.284
EZR	ezrin	4.70E-01	1.533	6.72E-01	1.354	3.55E-02	2.326	9.76E-01	-1.012
F3	coagulation factor III (thromboplastin, tissue factor)	1.00E00	2.169	1.00E00	0.000	4.20E-03	4.412	3.68E-01	1.350
FANCD2	Fanconi anemia, complementation group D2	1.00E00	0.000	1.00E00	0.000	2.87E-03	2.852	1.05E-01	2.113
FAP	fibroblast activation protein, alpha					2.21E-02	13.090	1.00E00	
FHL1	four and a half LIM domains 1					2.10E-02	-2.880	3.44E-02	-1.783
FKBP5	FK506 binding protein 5	5.84E-01	-1.202	5.13E-01	1.276	3.74E-03	-1.378	2.37E-01	1.083

FPGS	folylpolyglutamate synthase	1.00E00	0.000	1.00E00	0.000	6.45E-03	4.414	7.01E-01	-1.229
FSCN1	fascin actin-bundling protein 1	7.47E-01	1.046	3.12E-01	-1.807	2.35E-02	-1.344	1.81E-02	-1.349
FXN	frataxin	1.00E00	0.000	1.00E00	0.000	3.71E-04	-1.877	1.48E-01	-1.835
G3BP1	GTPase activating protein (SH3 domain) binding protein 1	7.62E-01	1.162	9.59E-01	1.024	2.86E-02	-1.388	2.53E-02	-1.356
G6PD	glucose-6-phosphate dehydrogenase	4.85E-01	-1.477	8.33E-01	-1.124	4.02E-02	-1.406	8.21E-01	-1.038
GBP2	guanylate binding protein 2, interferon-inducible	1.00E00	0.000	1.00E00	0.000	1.36E-02	2.228	1.29E-01	-1.931
GLDC	glycine dehydrogenase (decarboxylating)	1.00E00	0.000	1.00E00	0.000	1.68E-02	-2.631	5.56E-02	1.997
GLRX	glutaredoxin (thioltransferase)	1.00E00	1.035	1.00E00	-1.986	3.26E-02	2.976	2.46E-01	-1.715
GPX1	glutathione peroxidase 1	5.95E-01	1.428	6.56E-01	1.449	1.46E-02	1.273	1.51E-01	1.193
GRB7	growth factor receptor-bound protein 7	1.00E00	0.000	1.00E00	0.000	3.20E-02	1.867	1.00E00	1.058
GRN	granulin					7.46E-04	1.284	2.37E-01	1.124
GSN	gelsolin	4.22E-01	1.945	1.00E00	-1.706	2.18E-03	1.703	1.23E-01	-1.197
GTF2I	general transcription factor IIi	2.48E-01	-1.295	5.24E-01	-1.528	5.99E-03	-1.282	1.35E-02	-1.186
HDAC2	histone deacetylase 2	1.00E00	-3.841	1.00E00	-1.241	4.63E-02	-1.220	4.45E-01	-1.123
HDGFRP3	hepatoma-derived growth factor, related protein 3	1.00E00	0.000	1.00E00	0.000	4.47E-03	-2.817	4.36E-02	-2.259
HNRNPK	heterogeneous nuclear ribonucleoprotein K	6.23E-01	1.079	6.77E-01	1.057	1.97E-02	-1.205	3.08E-01	-2.032
HNRNPR	heterogeneous nuclear ribonucleoprotein R	9.68E-01	1.014	6.88E-01	1.149	2.24E-03	-1.249	3.83E-01	-1.107
HSP90AA1	heat shock protein 90kDa alpha (cytosolic), class A member 1	6.54E-01	-1.196	9.28E-01	-1.024	3.43E-02	-1.335	1.36E-01	-1.143
HSPA8	heat shock 70kDa protein 8	7.91E-01	1.055	3.99E-01	1.176	4.62E-02	-2.934	5.98E-01	2.678
HSPA1A/HSP/	heat shock 70kDa protein 1A	1.43E-01	1.284	3.50E-01	1.333	2.34E-03	-1.242	1.93E-01	-1.064
ICAM1	intercellular adhesion molecule 1	1.00E00	1.102	1.00E00	0.000	1.20E-02	4.093	4.97E-01	-1.175
IFI16	interferon, gamma-inducible protein 16	9.19E-01	-1.030	3.01E-01	-1.441	4.97E-02	1.281	9.11E-01	1.012
IKBKG	inhibitor of kappa light polypeptide gene enhancer in B-cells, kinase gamma	1.00E00	1.673	1.00E00	1.769	5.03E-03	1.742	2.19E-01	1.327
IL18	interleukin 18	3.74E-01	-1.508	6.32E-01	-1.222	5.63E-03	1.981	1.43E-03	1.372
IL1RN	interleukin 1 receptor antagonist	1.00E00	0.000	1.00E00	0.000	4.59E-02	5.796	2.60E-01	3.522
ILKAP	integrin-linked kinase-associated serine/threonine phosphatase	1.00E00	-1.000	1.00E00	-1.490	4.27E-02	-1.589	2.27E-01	-1.253
IMPDH1	IMP (inosine 5'-monophosphate) dehydrogenase 1	4.14E-01	-1.638	3.19E-01	1.312	3.17E-02	-1.864	9.72E-01	1.006
IMPDH2	IMP (inosine 5'-monophosphate) dehydrogenase 2	4.36E-01	-1.179	1.30E-01	1.633	3.88E-02	-1.335	5.64E-01	1.060
ITGA5	integrin, alpha 5 (fibronectin receptor, alpha polypeptide)	3.64E-01	-2.172	1.00E00	-3.726	4.33E-02	-2.098	2.27E-02	-2.119
ITGB4	integrin, beta 4	5.06E-01	-1.180	7.55E-01	-1.092	3.98E-02	-1.164	1.00E00	-1.292
KEAP1	kelch-like ECH-associated protein 1	1.00E-01	1.388	1.00E00	0.000	4.76E-02	1.310	1.49E-01	-1.211
KLF13	Kruppel-like factor 13					2.56E-02	-2.008	1.00E00	-2.097
LAMA5	laminin, alpha 5	2.69E-01	-2.706	1.00E00	-3.277	1.45E-02	-1.401	4.71E-02	-2.294
LAMB1	laminin, beta 1	2.92E-02	-2.479	1.00E00	1.431	1.80E-02	-1.475	2.43E-01	-1.522
LARP1	La ribonucleoprotein domain family, member 1	9.25E-01	-1.031	7.45E-01	1.121	4.05E-02	-1.338	2.63E-01	-1.148
LASP1	LIM and SH3 protein 1	8.38E-01	1.067	2.15E-01	-1.671	2.73E-02	1.248	8.94E-01	-1.013
LGALS3	lectin, galactoside-binding, soluble, 3	2.19E-01	1.602	5.08E-01	-1.695	1.83E-02	2.188	6.11E-01	1.139
MAFF	v-maf avian musculoaponeurotic fibrosarcoma oncogene homolog F	1.00E00	0.000	1.00E00	0.000	6.53E-04	2.808	2.29E-01	1.384
MAP2	microtubule-associated protein 2	1.00E00	0.000	1.00E00	0.000	1.21E-03	14.588	1.00E00	5.876
MAP2K2	mitogen-activated protein kinase kinase 2	1.00E00	-1.030	1.00E00	1.079	2.57E-02	-1.397	2.02E-02	-1.306
MCM2	minichromosome maintenance complex component 2	2.17E-01	-1.655	4.85E-01	-1.317	2.09E-03	-1.322	1.63E-01	-1.166
MCM3	minichromosome maintenance complex component 3	8.46E-02	-1.798	4.92E-01	-1.091	4.82E-02	-1.403	1.22E-01	-1.283
MCM5	minichromosome maintenance complex component 5	1.04E-01	-2.672	2.31E-01	-2.414	4.21E-02	-1.204	9.31E-01	1.006
MET	MET proto-oncogene, receptor tyrosine kinase	1.00E00	0.000	1.00E00	0.000	4.99E-02	-1.277	6.91E-01	1.046
MTHFD1	methylenetetrahydrofolate dehydrogenase (NADP+ dependent) 1, methenyltetrahy	8.02E-03	-1.311	2.64E-01	-1.172	1.29E-03	-1.305	3.85E-01	-1.071
MVP	major vault protein	1.50E-02	1.448	5.34E-01	-1.110	3.14E-04	1.512	1.29E-01	-1.259
MYBBP1A	MYB binding protein (P160) 1a	1.16E-02	-1.524	5.32E-01	-1.070	3.38E-02	-1.329	5.27E-02	-1.161
MYL9	myosin, light chain 9, regulatory	1.00E00	0.000	1.00E00	0.000	4.54E-02	3.181	1.00E00	-1.094
NASP	nuclear autoantigenic sperm protein (histone-binding)	1.51E-01	-2.073	2.57E-01	-1.420	1.37E-02	-1.205	7.64E-01	1.015

NCK1	NCK adaptor protein 1	1.00E00	1.371	1.00E00	1.688	3.97E-03	1.850	3.49E-01	1.681
NDE1	nudE neurodevelopment protein 1	4.11E-01	1.270	1.00E00	0.000	2.32E-03	2.586	9.40E-01	1.014
NOLC1	nucleolar and coiled-body phosphoprotein 1	5.80E-01	1.785	8.25E-01	1.283	4.09E-06	-1.731	3.65E-02	-8.090
NT5C3A	5'-nucleotidase, cytosolic IIIA	1.00E00	-1.229			2.99E-02	1.600	1.99E-01	1.855
ORC5	origin recognition complex, subunit 5					2.19E-02	-1.486	7.71E-02	-1.196
P4HA2	prolyl 4-hydroxylase, alpha polypeptide II	1.22E-01	1.756	5.53E-01	1.271	3.82E-02	-1.767	2.44E-02	-1.820
PACSIN2	protein kinase C and casein kinase substrate in neurons 2	4.88E-01	-1.095	8.09E-01	-1.092	3.43E-02	-1.191	3.82E-02	-1.199
PAK1	p21 protein (Cdc42/Rac)-activated kinase 1					3.34E-02	-1.594	1.00E00	
PARP1	poly (ADP-ribose) polymerase 1	2.31E-01	-1.364	4.50E-01	-1.262	2.46E-03	-1.234	3.58E-01	1.134
PAWR	PRKC, apoptosis, WT1, regulator	1.00E00	1.098	1.00E00	0.000	7.64E-04	2.685	1.95E-01	1.444
PDE4D	phosphodiesterase 4D, cAMP-specific	1.00E00	0.000	1.00E00	0.000	7.98E-03	-3.140	1.15E-01	-1.516
PDK1	pyruvate dehydrogenase kinase, isozyme 1					6.12E-03	-1.794	6.34E-02	-1.437
PDXK	pyridoxal (pyridoxine, vitamin B6) kinase	2.98E-01	-1.704	8.37E-01	-1.117	1.01E-02	1.757	2.94E-01	1.203
PGRMC1	progesterone receptor membrane component 1	8.46E-01	1.168	3.56E-01	-2.648	1.94E-02	-1.380	4.82E-02	-1.261
PHC3	polyhomeotic homolog 3 (Drosophila)					1.16E-02	-1.614	1.00E00	-1.017
PHF6	PHD finger protein 6	1.00E00	0.000	1.00E00	0.000	2.84E-02	-1.496	4.81E-01	-1.107
PKD1	polycystic kidney disease 1 (autosomal dominant)					3.21E-02	1.598	1.83E-02	1.797
PKHD1	polycystic kidney and hepatic disease 1 (autosomal recessive)					2.93E-02	2.475	6.49E-02	2.228
PKM	pyruvate kinase, muscle	5.43E-01	-1.856	1.00E00	-1.791	4.20E-02	-1.686	2.55E-01	-1.288
PLA2G4A	phospholipase A2, group IVA (cytosolic, calcium-dependent)	1.00E00	0.000	1.00E00	0.000	4.44E-02	-1.379	3.22E-01	1.211
POLR2H	polymerase (RNA) II (DNA directed) polypeptide H	1.00E00	-1.289	1.00E00	-1.050	2.42E-02	-1.731	3.23E-01	-1.323
PPAT	phosphoribosyl pyrophosphate amidotransferase	5.21E-01	-1.316	4.20E-01	-1.097	4.83E-02	1.675	2.37E-01	1.608
PPM1A	protein phosphatase, Mg2+/Mn2+ dependent, 1A	1.00E00	3.041	1.00E00	0.000	1.54E-02	-1.391	3.32E-01	-1.153
PPP1CC	protein phosphatase 1, catalytic subunit, gamma isozyme	1.00E00	0.000	1.00E00	-3.795	3.37E-02	1.428	2.43E-03	1.450
PPP1R10	protein phosphatase 1, regulatory subunit 10	1.00E00	-1.016	1.00E00	1.139	2.36E-02	-1.314	6.81E-02	-1.247
PRDX4	peroxiredoxin 4	2.51E-01	-1.744	1.00E00	-1.241	2.45E-02	-1.501	3.44E-01	-1.218
PSMB10	proteasome (prosome, macropain) subunit, beta type, 10	1.00E00	0.000	1.00E00	0.000	1.03E-02	2.319	1.00E00	-1.566
PSMC2	proteasome (prosome, macropain) 26S subunit, ATPase, 2	9.94E-01	-1.001	3.26E-01	1.152	1.89E-02	1.127	2.62E-03	1.287
PTPN3	protein tyrosine phosphatase, non-receptor type 3					2.54E-02	1.387	1.00E00	
RAC2	ras-related C3 botulinum toxin substrate 2 (rho family, small GTP binding protein R	1.00E00	0.000	1.00E00	0.000	1.04E-03	1.399	7.67E-02	-1.210
RAPH1	Ras association (RalGDS/AF-6) and pleckstrin homology domains 1	1.00E00	-2.120	1.00E00	0.000	8.84E-03	2.205	1.00E00	-1.481
RB1CC1	RB1-inducible coiled-coil 1	1.00E00	0.000	1.00E00	0.000	3.58E-02	1.808	5.38E-02	1.256
RBBP7	retinoblastoma binding protein 7	9.33E-01	1.031	7.44E-01	1.077	1.38E-02	-3.281	6.28E-03	-6.779
RBX1	ring-box 1, E3 ubiquitin protein ligase	1.00E00	0.000	1.00E00	0.000	2.30E-02	-1.494	7.36E-01	-1.081
RGL2	ral guanine nucleotide dissociation stimulator-like 2					3.57E-02	1.957	1.00E00	
RPS6KA4	ribosomal protein S6 kinase, 90kDa, polypeptide 4	1.00E00	-1.026	1.00E00	-1.418	2.13E-02	-1.624	1.13E-01	-1.257
S100A4	S100 calcium binding protein A4	9.45E-01	1.096	3.90E-03	7.360	3.00E-02	-1.797	1.74E-01	1.771
S100A7	S100 calcium binding protein A7	1.00E00	0.000	1.00E00	0.000	1.46E-03	4.970	1.18E-01	-2.276
S100A11	S100 calcium binding protein A11	7.07E-01	-1.259	7.38E-01	-1.312	1.84E-03	1.639	6.48E-02	1.255
S100P	S100 calcium binding protein P	1.00E00	0.000	1.00E00	0.000	1.45E-02	61.983	5.04E-01	2.709
SAA1	serum amyloid A1	1.00E00	-1.394	1.00E00	10.687	2.30E-02	4.835	7.62E-02	3.014
SCD5	stearoyl-CoA desaturase 5	1.00E00	0.000	1.00E00	0.000	1.86E-03	4.292	8.06E-01	-1.127
SERPINB2	serpin peptidase inhibitor, clade B (ovalbumin), member 2	1.00E00	0.000	1.00E00	0.000	4.57E-02	8.354	3.64E-01	1.973
SF1	splicing factor 1	7.79E-01	-1.178	3.83E-01	-1.619	4.35E-02	-2.053	2.36E-01	-1.544
SF3B2	splicing factor 3b, subunit 2, 145kDa	5.57E-01	1.231	4.54E-01	1.157	4.07E-02	-1.251	1.39E-01	-1.242
SLC1A3	solute carrier family 1 (glial high affinity glutamate transporter), member 3	1.00E00	0.000	1.00E00	0.000	1.05E-02	1.773	3.50E-01	1.439
SLC35F6	solute carrier family 35, member F6	1.00E00	0.000	1.00E00	0.000	2.17E-02	2.283	7.38E-01	-1.205
SLPI	secretory leukocyte peptidase inhibitor					1.03E-02	9.998	3.86E-01	1.397
SMARCB1	SWI/SNF related, matrix associated, actin dependent regulator of chromatin, subfa	1.00E00	0.000	1.00E00	0.000	1.38E-02	-1.689	1.69E-01	-1.220

SMYD3	SET and MYND domain containing 3	1.00E00	0.000	1.00E00	0.000	1.27E-02	-1.560	4.29E-01	1.101
SND1	staphylococcal nuclease and tudor domain containing 1	4.51E-01	-1.173	7.43E-01	1.075	2.77E-02	-1.319	3.66E-01	-1.094
SNX3	sorting nexin 3	5.64E-01	-1.325	9.25E-01	1.030	1.45E-02	-1.306	4.88E-02	-1.335
SOD1	superoxide dismutase 1, soluble	4.63E-01	2.670	7.65E-01	1.356	1.68E-02	2.011	6.23E-01	-1.434
SPTAN1	spectrin, alpha, non-erythrocytic 1	5.03E-01	1.440	7.50E-01	1.140	8.59E-03	-2.105	5.65E-01	1.132
SPTBN1	spectrin, beta, non-erythrocytic 1	2.24E-01	-1.138	2.01E-02	-1.613	7.98E-03	1.407	7.00E-01	-1.251
SRGAP2	SLIT-ROBO Rho GTPase activating protein 2	1.00E00	-1.391	1.00E00	0.000	4.23E-02	1.481	5.28E-01	1.125
SRRT	serrate, RNA effector molecule	7.55E-01	-1.258	2.95E-01	-2.175	4.81E-02	-1.271	5.74E-01	1.054
STARD13	StAR-related lipid transfer (START) domain containing 13	1.00E00	0.000	1.00E00	0.000	2.96E-02	1.725	5.80E-02	-1.598
STEAP3	STEAP family member 3, metalloreductase	1.00E00	0.000	1.00E00	0.000	3.72E-02	-4.257	1.00E00	-3.302
STK38L	serine/threonine kinase 38 like					5.28E-03	1.529	1.95E-01	-2.129
STRAP	serine/threonine kinase receptor associated protein	4.66E-01	-1.445	7.06E-01	-1.186	2.65E-02	-1.333	6.87E-03	-1.382
SUPV3L1	suppressor of var1, 3-like 1 (S. cerevisiae)	8.95E-01	1.038	5.81E-01	1.242	2.67E-02	-1.578	2.31E-01	1.220
SURF1	surfeit 1	1.00E00	0.000	1.00E00	0.000	1.08E-02	2.147	5.18E-01	-1.324
TAB3	TGF-beta activated kinase 1/MAP3K7 binding protein 3	1.00E00	0.000	1.00E00	0.000	4.07E-02	-2.665	8.68E-01	-1.077
TAGLN	transgelin	2.67E-01	3.005	5.64E-01	1.918	3.32E-02	3.084	6.83E-01	-1.179
TBC1D8	TBC1 domain family, member 8 (with GRAM domain)	1.00E00	0.000	1.00E00	0.000	4.02E-02	7.103	1.89E-01	6.284
TBK1	TANK-binding kinase 1	1.00E00	1.058	1.00E00	0.000	4.25E-02	-1.305	3.19E-01	-1.093
TCEB3	transcription elongation factor B (SIII), polypeptide 3 (110kDa, elongin A)	8.17E-01	1.238	1.00E00	1.033	1.71E-02	-2.843	5.92E-01	-1.370
TCF12	transcription factor 12					1.43E-02	-1.871	1.00E00	-2.456
TCP1	t-complex 1	2.91E-01	-1.347	8.89E-01	1.042	2.03E-02	-1.191	5.97E-01	1.028
TFG	TRK-fused gene	3.41E-01	-1.314	1.00E00	-1.723	5.74E-03	2.478	1.40E-01	1.537
THEM4	thioesterase superfamily member 4	1.00E00	-1.251	1.00E00	1.066	5.45E-03	-1.605	6.83E-01	-1.141
TIMP1	TIMP metallopeptidase inhibitor 1	8.12E-01	1.079	1.00E00	-1.174	1.98E-02	1.741	9.21E-01	1.008
TIMP3	TIMP metallopeptidase inhibitor 3	1.00E00	-2.697	1.00E00	0.000	4.13E-03	-1.852	4.38E-01	-1.628
TJP1	tight junction protein 1	2.00E-01	1.561	7.48E-01	-1.153	2.54E-02	1.457	6.26E-01	-1.098
TJP2	tight junction protein 2	9.86E-01	-1.004	3.42E-01	-1.502	1.24E-02	1.377	1.70E-01	1.164
TJP3	tight junction protein 3					4.53E-02	2.144	5.41E-01	1.335
TLR3	toll-like receptor 3					4.57E-02	-1.300	4.35E-02	-2.468
TMSB10/TMSE	thymosin beta 10	2.63E-01	3.370	2.81E-01	1.836	3.77E-02	5.288	7.55E-01	-1.269
TNS3	tensin 3	1.00E00	-1.941	1.00E00	0.000	2.92E-02	-1.453	1.28E-01	-1.159
TOM1L1	target of myb1 (chicken)-like 1	1.00E00	2.504	1.00E00	0.000	2.89E-02	-1.651	7.87E-02	-1.487
TOP1MT	topoisomerase (DNA) I, mitochondrial					3.86E-02	1.338	7.10E-02	2.347
TPD52	tumor protein D52	3.64E-01	-1.420	1.00E00	0.000	4.08E-02	2.962	3.36E-01	1.648
TPD52L1	tumor protein D52-like 1	4.74E-02	1.692	3.73E-01	1.739	3.92E-02	2.140	8.26E-01	1.079
TPM3	tropomyosin 3	2.84E-01	1.673	7.94E-01	1.192	4.80E-02	1.372	1.14E-01	-2.110
TRIM27	tripartite motif containing 27	1.00E00	0.000	1.00E00	0.000	2.37E-02	1.677	3.69E-01	1.230
TXNRD1	thioredoxin reductase 1	5.04E-01	1.560	9.93E-01	1.012	2.08E-02	-2.032	9.20E-02	-1.882
UBE2I	ubiquitin-conjugating enzyme E2I	1.38E-01	-1.801	8.79E-02	-1.654	1.19E-02	-1.485	6.95E-02	-1.283
UBE2L3	ubiquitin-conjugating enzyme E2L 3	9.58E-01	-1.053	9.55E-01	-1.079	1.42E-02	4.031	2.62E-01	1.810
UNC93B1	unc-93 homolog B1 (C. elegans)	1.00E00	0.000	1.00E00	0.000	3.29E-02	-2.407	1.00E00	-1.960
VIM	vimentin	9.23E-01	-1.058	7.32E-01	-1.226	9.94E-04	-1.740	2.98E-02	-1.433
WDR6	WD repeat domain 6	1.00E00	-1.574	1.00E00	0.000	2.82E-02	-1.777	3.01E-02	-2.105
YBX3	Y box binding protein 3	6.81E-01	1.320	4.39E-01	-1.621	3.77E-02	-1.300	1.55E-02	-1.410
ZMYM2	zinc finger, MYM-type 2					2.60E-03	-3.282	3.67E-03	-4.371

	LMP2-with Inhs Proliferation of Cells (P = 3.35 x 10^{-6})	LMP1 no Inhs		LMP2 no Inhs		LMP1 with Inhs		LMP2 with Inhs	
**Symbol**	**Entrez Gene Name**	**p-value**	**Fold Change**	**p-value**	**Fold Change**	**p-value**	**Fold Change**	**p-value**	**Fold Change**
ACLY	ATP citrate lyase	3.01E-01	-1.500	9.17E-01	1.032	3.15E-02	1.159	3.79E-02	1.167

AGTRAP	angiotensin II receptor-associated protein	1.00E00	0.000	1.00E00	0.000	2.73E-01	-1.247	1.72E-02	-1.592
AKR1C1/AKR1	aldo-keto reductase family 1, member C2	1.00E00	2.836	1.00E00	-1.145	2.56E-01	-1.795	1.29E-02	2.105
ALOX15B	arachidonate 15-lipoxygenase, type B	1.00E00	2.993	1.00E00	0.000	5.00E-02	4.297	3.11E-02	1.402
ANXA6	annexin A6	1.00E00	0.000	1.00E00	0.000	9.17E-01	1.062	3.45E-02	-1.977
API5	apoptosis inhibitor 5	6.53E-01	1.114	7.95E-01	1.234	8.46E-02	-1.360	1.87E-02	-1.382
ATAT1	alpha tubulin acetyltransferase 1	1.00E00	0.000	1.00E00	0.000	1.00E00	-1.707	3.77E-02	1.776
ATF1	activating transcription factor 1					5.54E-01	1.108	3.67E-02	1.330
ATP5B	ATP synthase, H+ transporting, mitochondrial F1 complex, beta polypeptide	3.44E-01	-1.221	8.35E-01	1.103	1.00E00	-1.570	1.52E-02	-2.313
ATP6V0D1	ATPase, H+ transporting, lysosomal 38kDa, V0 subunit d1	1.00E00	0.000	1.00E00	0.000	3.25E-02	1.934	4.54E-02	1.720
ATPIF1	ATPase inhibitory factor 1	1.00E00	3.577	1.00E00	6.682	1.86E-01	-1.186	2.81E-03	1.617
B4GALT1	UDP-Gal:betaGlcNAc beta 1,4- galactosyltransferase, polypeptide 1	1.00E00	0.000	1.00E00	0.000	9.51E-02	1.507	1.64E-02	-1.201
BCL2L11	BCL2-like 11 (apoptosis facilitator)					2.64E-01	-2.048	3.15E-02	-1.593
BPNT1	3'(2'), 5'-bisphosphate nucleotidase 1	8.47E-01	1.040	5.96E-01	1.109	7.85E-02	1.155	4.49E-02	1.199
BRK1	BRICK1, SCAR/WAVE actin-nucleating complex subunit	1.00E00	1.672	1.00E00	0.000	4.45E-01	1.045	2.15E-02	-1.382
C9orf78	chromosome 9 open reading frame 78	1.00E00	0.000	1.00E00	0.000	4.73E-02	-1.681	1.18E-02	-2.795
CA12	carbonic anhydrase XII	1.00E00	0.000	1.00E00	0.000	5.84E-01	-1.034	8.98E-03	2.884
CBR1	carbonyl reductase 1	9.75E-01	-1.004	1.50E-01	1.242	5.81E-01	1.061	1.39E-02	1.397
CBX4	chromobox homolog 4	1.00E00	0.000	1.00E00	0.000	5.50E-01	-1.056	4.31E-02	1.425
CCHCR1	coiled-coil alpha-helical rod protein 1					3.59E-01	1.339	3.34E-02	1.914
CD2AP	CD2-associated protein	6.28E-01	1.300	1.59E-01	2.012	8.76E-01	1.007	1.93E-02	-1.208
CDC27	cell division cycle 27	1.00E00	0.000	1.00E00	0.000	2.98E-01	-1.452	1.10E-02	-2.389
CDKN1C	cyclin-dependent kinase inhibitor 1C (p57, Kip2)	1.00E00	0.000	1.00E00	0.000	2.48E-01	1.536	4.13E-03	-1.767
CEBPD	CCAAT/enhancer binding protein (C/EBP), delta	9.02E-02	-1.547	2.94E-01	-1.458	6.40E-01	1.213	3.42E-02	1.828
CENPB	centromere protein B, 80kDa	1.00E00	0.000	1.00E00	0.000	7.29E-01	1.061	8.70E-03	-1.487
CHD4	chromodomain helicase DNA binding protein 4	7.52E-01	-1.213	7.10E-01	1.110	9.81E-02	-1.276	4.86E-02	-1.420
CKS2	CDC28 protein kinase regulatory subunit 2					9.88E-02	1.622	2.27E-02	1.783
CLCA2	chloride channel accessory 2	3.87E-01	-1.357	1.00E00	-2.046	7.82E-01	-1.084	1.99E-02	2.104
COPS6	COP9 signalosome subunit 6	1.00E00	0.000	1.00E00	0.000	5.68E-03	-1.935	3.01E-05	-2.304
CSNK1D	casein kinase 1, delta	1.00E00	0.000	1.00E00	0.000	2.53E-02	-1.754	4.94E-02	-1.485
CYR61	cysteine-rich, angiogenic inducer, 61	1.00E00	0.000	1.00E00	0.000	4.67E-01	-1.418	9.40E-03	-2.249
DAB2IP	DAB2 interacting protein	1.00E00	0.000	1.00E00	0.000	1.99E-01	1.401	3.60E-02	-1.565
DHCR24	24-dehydrocholesterol reductase	1.00E00	0.000	1.00E00	0.000	2.07E-01	1.410	7.50E-03	-1.884
DNAJA1	DnaJ (Hsp40) homolog, subfamily A, member 1	2.81E-01	1.344	9.41E-01	-1.020	1.21E-01	-1.493	5.00E-02	-1.763
DYRK1A	dual-specificity tyrosine-(Y)-phosphorylation regulated kinase 1A					7.80E-02	-1.271	3.54E-02	-1.395
ECSIT	ECSIT signalling integrator	1.00E00	0.000	1.00E00	0.000	1.41E-02	-2.365	1.77E-02	-2.634
EEF1D	eukaryotic translation elongation factor 1 delta (guanine nucleotide exchange prote	6.47E-01	1.485	5.90E-01	1.601	1.17E-01	-1.375	8.67E-03	3.326
EI24	etoposide induced 2.4	1.00E00	-3.013	1.00E00	0.000	9.20E-01	1.041	4.92E-02	-2.038
EIF5	eukaryotic translation initiation factor 5	3.46E-01	1.602	6.89E-01	1.275	1.22E-01	-1.603	4.54E-02	-1.458
EIF3B	eukaryotic translation initiation factor 3, subunit B	3.42E-01	-1.386	8.48E-01	1.074	1.02E-02	-1.676	2.12E-03	-1.435
EIF3E	eukaryotic translation initiation factor 3, subunit E	2.79E-01	1.270	5.54E-01	-1.191	4.88E-02	-1.981	2.96E-02	-1.483
EIF4A1	eukaryotic translation initiation factor 4A1	2.22E-02	-1.494	6.00E-01	-1.108	5.36E-03	-1.474	2.21E-03	-1.518
ENO1	enolase 1, (alpha)	3.39E-01	1.109	1.62E-01	1.484	6.46E-03	-2.532	8.54E-03	-2.414
ERCC2	excision repair cross-complementation group 2	1.00E00	0.000	1.00E00	0.000	9.09E-01	1.060	4.97E-02	1.761
EXOC5	exocyst complex component 5	1.00E00	0.000	1.00E00	0.000	1.52E-01	-1.403	1.07E-02	-2.131
FAS	Fas cell surface death receptor	1.00E00	-1.102	1.00E00	0.000	1.24E-01	-1.230	3.08E-03	-1.720
FHL1	four and a half LIM domains 1					2.10E-02	-2.880	3.44E-02	-1.783
FMR1	fragile X mental retardation 1	7.18E-02	-2.219	3.05E-01	-1.654	3.63E-01	-1.454	3.98E-02	-1.650
FSCN1	fascin actin-bundling protein 1	7.47E-01	1.046	3.12E-01	-1.807	2.35E-02	-1.344	1.81E-02	-1.349
G3BP1	GTPase activating protein (SH3 domain) binding protein 1	7.62E-01	1.162	9.59E-01	1.024	2.86E-02	-1.388	2.53E-02	-1.356

GALNS	galactosamine (N-acetyl)-6-sulfatase	1.00E00	0.000	1.00E00	0.000	6.33E-01	-1.127	1.59E-02	-2.250
GALNT2	polypeptide N-acetylgalactosaminyltransferase 2	5.04E-01	-1.913	1.00E00	-3.062	1.15E-01	-1.612	1.00E-02	-3.197
GPHN	gephyrin	3.38E-01	1.207	1.00E00	1.016	3.95E-01	1.581	3.82E-02	3.456
GTF2I	general transcription factor IIi	2.48E-01	-1.295	5.24E-01	-1.528	5.99E-03	-1.282	1.35E-02	-1.186
H2AFX	H2A histone family, member X	8.16E-01	-1.078	9.95E-01	1.009	8.35E-01	1.046	4.72E-02	1.489
HDGFRP3	hepatoma-derived growth factor, related protein 3	1.00E00	0.000	1.00E00	0.000	4.47E-03	-2.817	4.36E-02	-2.259
HMMR	hyaluronan-mediated motility receptor (RHAMM)	1.00E00	0.000	1.00E00	0.000	1.00E00	-3.729	2.60E-02	-3.481
HNRNPA2B1	heterogeneous nuclear ribonucleoprotein A2/B1	3.72E-01	-1.186	9.16E-01	1.021	7.09E-02	-1.330	2.14E-03	2.533
IGBP1	immunoglobulin (CD79A) binding protein 1	8.70E-01	-1.056	1.00E00	-1.076	2.00E-01	-1.267	7.97E-03	-1.947
IGF2BP3	insulin-like growth factor 2 mRNA binding protein 3	7.30E-01	1.216	3.56E-03	-1.963	5.05E-01	-1.102	1.29E-02	-2.325
IL18	interleukin 18	3.74E-01	-1.508	6.32E-01	-1.222	5.63E-03	1.981	1.43E-03	1.372
INPP5K	inositol polyphosphate-5-phosphatase K					3.88E-01	1.251	3.46E-02	-1.270
ITGA5	integrin, alpha 5 (fibronectin receptor, alpha polypeptide)	3.64E-01	-2.172	1.00E00	-3.726	4.33E-02	-2.098	2.27E-02	-2.119
KANK2	KN motif and ankyrin repeat domains 2	1.00E00	0.000	1.00E00	0.000	8.80E-02	-1.928	6.18E-03	-2.708
KIF15	kinesin family member 15	1.00E00	0.000	1.00E00	0.000	6.96E-01	1.255	3.44E-02	1.374
LAMA5	laminin, alpha 5	2.69E-01	-2.706	1.00E00	-3.277	1.45E-02	-1.401	4.71E-02	-2.294
LAMB2	laminin, beta 2 (laminin S)	1.00E00	0.000	1.00E00	0.000	9.33E-02	1.599	3.25E-02	1.709
LIMA1	LIM domain and actin binding 1	1.68E-01	1.738	6.78E-01	1.140	1.00E00	1.439	1.59E-03	1.192
MAGED2	melanoma antigen family D, 2	1.00E00	0.000	1.00E00	0.000	2.86E-01	-1.460	4.14E-02	-2.129
MAP2K2	mitogen-activated protein kinase kinase 2	1.00E00	-1.030	1.00E00	1.079	2.57E-02	-1.397	2.02E-02	-1.306
MBD1	methyl-CpG binding domain protein 1	1.00E00	0.000	1.00E00	0.000	3.34E-01	-1.117	2.46E-03	-1.734
MCAM	melanoma cell adhesion molecule					5.91E-01	1.219	3.11E-02	1.409
MCFD2	multiple coagulation factor deficiency 2	1.00E00	0.000	1.00E00	0.000	5.04E-02	2.420	3.80E-02	2.469
MCM7	minichromosome maintenance complex component 7	2.36E-01	-1.658	1.82E-01	-1.311	1.73E-01	-1.253	4.74E-02	-1.355
MORC3	MORC family CW-type zinc finger 3	1.00E00	0.000	1.00E00	0.000	4.95E-01	-1.263	1.13E-02	-2.036
MSI2	musashi RNA-binding protein 2	1.00E00	1.317	1.00E00	1.552	1.00E00	-1.322	1.95E-02	-1.841
MYCBP	MYC binding protein	1.00E00	-1.266	1.00E00	-1.433	1.96E-01	1.464	4.50E-02	-1.363
NFKB1	nuclear factor of kappa light polypeptide gene enhancer in B-cells 1	7.54E-01	-1.280	8.35E-01	1.177	9.42E-02	1.205	1.63E-02	1.226
NOLC1	nucleolar and coiled-body phosphoprotein 1	5.80E-01	1.785	8.25E-01	1.283	4.09E-06	-1.731	3.65E-02	-8.090
NPM1	nucleophosmin (nucleolar phosphoprotein B23, numatrin)	3.04E-01	-1.476	1.10E-01	-1.239	2.68E-01	-1.670	1.58E-02	1.215
OGT	O-linked N-acetylglucosamine (GlcNAc) transferase	4.88E-01	1.135	9.90E-01	1.007	9.40E-01	1.015	2.70E-02	-1.424
P4HA2	prolyl 4-hydroxylase, alpha polypeptide II	1.22E-01	1.756	5.53E-01	1.271	3.82E-02	-1.767	2.44E-02	-1.820
PACSIN2	protein kinase C and casein kinase substrate in neurons 2	4.88E-01	-1.095	8.09E-01	-1.092	3.43E-02	-1.191	3.82E-02	-1.199
PCYT1A	phosphate cytidylyltransferase 1, choline, alpha	1.00E00	-1.189	1.00E00	1.122	9.14E-02	-1.214	2.16E-03	-1.463
PELO	pelota homolog (Drosophila)	1.00E00	0.000	1.00E00	0.000	7.19E-01	-1.076	4.25E-02	-1.640
PFKP	phosphofructokinase, platelet	2.67E-01	-1.420	1.07E-01	-1.426	2.00E-01	-1.247	4.26E-02	-1.148
PGRMC1	progesterone receptor membrane component 1	8.46E-01	1.168	3.56E-01	-2.648	1.94E-02	-1.380	4.82E-02	-1.261
PKD1	polycystic kidney disease 1 (autosomal dominant)					3.21E-02	1.598	1.83E-02	1.797
PLEC	plectin					1.00E00	-3.095	3.74E-03	3.622
PLXNA4	plexin A4					3.17E-01	-1.142	3.87E-03	-3.151
PML	promyelocytic leukemia	4.01E-01	-1.174	2.33E-02	-2.367	7.11E-01	1.114	4.66E-02	-1.449
PNP	purine nucleoside phosphorylase	1.61E-01	1.495	9.39E-01	-1.053	8.46E-01	-1.029	4.98E-02	-1.793
POLB	polymerase (DNA directed), beta	1.00E00	1.219	1.00E00	0.000	2.14E-01	-1.677	4.88E-02	-1.640
PPP1CC	protein phosphatase 1, catalytic subunit, gamma isozyme	1.00E00	0.000	1.00E00	-3.795	3.37E-02	1.428	2.43E-03	1.450
PPP1R13L	protein phosphatase 1, regulatory subunit 13 like	4.18E-01	1.766	3.18E-01	-1.383	3.89E-01	1.170	4.07E-02	-1.352
PPP3R1	protein phosphatase 3, regulatory subunit B, alpha	1.00E00	0.000	1.00E00	0.000	1.33E-01	-1.373	1.02E-02	-1.585
PSMC2	proteasome (prosome, macropain) 26S subunit, ATPase, 2	9.94E-01	-1.001	3.26E-01	1.152	1.89E-02	1.127	2.62E-03	1.287
PSMD2	proteasome (prosome, macropain) 26S subunit, non-ATPase, 2	5.38E-03	1.171	1.21E-01	1.472	2.87E-01	-1.067	2.70E-02	-1.227
PTPN12	protein tyrosine phosphatase, non-receptor type 12	9.20E-02	2.137	1.80E-01	1.541	3.56E-01	-1.087	2.47E-02	-1.305

PXN	paxillin	2.26E-01	1.287	1.27E-01	-1.543	3.24E-01	1.546	1.68E-02	-1.271
RAD9A	RAD9 homolog A (S. pombe)	1.00E00	0.000	1.00E00	0.000	3.19E-01	-1.244	3.39E-02	-1.702
RBBP7	retinoblastoma binding protein 7	9.33E-01	1.031	7.44E-01	1.077	1.38E-02	-3.281	6.28E-03	-6.779
RBBP9	retinoblastoma binding protein 9	1.00E00	0.000	1.00E00	0.000	1.12E-01	-1.520	9.16E-03	-3.018
RHEB	Ras homolog enriched in brain	1.00E00	0.000	1.00E00	0.000	4.60E-01	-1.640	3.30E-02	-1.975
RIPK1	receptor (TNFRSF)-interacting serine-threonine kinase 1	1.00E00	0.000	1.00E00	0.000	3.61E-01	1.444	3.39E-03	-1.610
RNASEH2B	ribonuclease H2, subunit B	1.00E00	0.000	1.00E00	0.000	1.59E-01	1.209	1.02E-02	1.960
RPL26	ribosomal protein L26	6.22E-01	-1.402	8.23E-01	1.176	5.30E-01	1.102	1.21E-02	1.300
RPS15A	ribosomal protein S15a	6.98E-02	-1.702	3.15E-01	-1.182	9.19E-01	-1.093	2.40E-03	1.238
RPS6KB1	ribosomal protein S6 kinase, 70kDa, polypeptide 1	1.00E00	1.861	1.00E00	1.983	1.04E-01	-1.675	3.62E-02	-1.878
RSL1D1	ribosomal L1 domain containing 1	3.79E-01	-1.525	6.53E-01	1.254	9.58E-01	-1.005	4.30E-02	1.264
RTN4	reticulon 4	7.07E-01	-1.774	1.00E00	-1.053	6.29E-02	1.171	8.41E-03	-1.397
SCAF11	SR-related CTD-associated factor 11	1.00E00	0.000	1.00E00	0.000	7.35E-02	-1.303	2.09E-02	-1.508
SCAMP2	secretory carrier membrane protein 2	1.00E00	-1.884	1.00E00	0.000	7.57E-01	-1.075	8.07E-03	-2.146
SDCBP	syndecan binding protein (syntenin)	2.20E-01	-1.488	1.69E-01	-2.287	2.44E-01	-1.103	2.26E-03	-1.420
SLC3A2	solute carrier family 3 (amino acid transporter heavy chain), member 2	1.88E-01	-1.396	9.50E-01	-1.051	3.39E-01	-1.430	3.13E-02	1.445
SMAD2	SMAD family member 2	9.07E-01	-1.022	1.00E00	-1.036	3.94E-01	1.118	3.74E-02	-1.304
SNX3	sorting nexin 3	5.64E-01	-1.325	9.25E-01	1.030	1.45E-02	-1.306	4.88E-02	-1.335
STAU1	staufen double-stranded RNA binding protein 1	2.56E-01	1.342	4.38E-01	1.344	4.04E-01	1.111	3.33E-02	-1.379
STIM1	stromal interaction molecule 1	1.00E00	0.000	1.00E00	0.000	4.59E-01	1.167	3.48E-02	-1.984
STRAP	serine/threonine kinase receptor associated protein	4.66E-01	-1.445	7.06E-01	-1.186	2.65E-02	-1.333	6.87E-03	-1.382
SYNM	synemin, intermediate filament protein					5.28E-01	-1.141	1.52E-03	-2.020
TAF4	TAF4 RNA polymerase II, TATA box binding protein (TBP)-associated factor, 135k	1.00E00	0.000	1.00E00	0.000	8.06E-01	-1.053	4.00E-02	-1.449
TAF9	TAF9 RNA polymerase II, TATA box binding protein (TBP)-associated factor, 32kD			1.00E00	1.159	6.24E-01	-1.180	4.79E-03	-1.507
TGM2	transglutaminase 2	1.00E00	1.933	1.00E00	0.000	5.35E-01	1.128	1.79E-03	-3.500
TLN1	talin 1	6.90E-01	1.081	1.64E-01	-1.316	7.06E-02	1.063	2.74E-02	-1.154
TLR3	toll-like receptor 3					4.57E-02	-1.300	4.35E-02	-2.468
TOE1	target of EGR1, member 1 (nuclear)	1.00E00	0.000	1.00E00	0.000	5.20E-02	-1.797	4.99E-02	-2.612
TPX2	TPX2, microtubule-associated					7.32E-02	-7.719	3.53E-02	-19.498
TRAF6	TNF receptor-associated factor 6, E3 ubiquitin protein ligase	1.00E00	0.000	1.00E00	0.000	1.29E-01	-1.539	5.71E-03	-1.943
TRAP1	TNF receptor-associated protein 1	3.42E-01	-1.458	8.55E-01	-1.022	2.57E-01	-1.143	2.79E-02	1.389
TSC22D1	TSC22 domain family, member 1	1.00E00	0.000	1.00E00	0.000	1.00E00	-3.302	2.90E-02	-3.960
UBA1	ubiquitin-like modifier activating enzyme 1	3.03E-01	-1.249	6.97E-01	-1.154	6.85E-01	-1.019	2.85E-03	-1.117
UBE2C	ubiquitin-conjugating enzyme E2C	1.00E00	0.000	1.00E00	0.000	1.91E-01	-1.937	1.21E-02	-5.242
UBE2L6	ubiquitin-conjugating enzyme E2L 6	1.00E00	0.000	1.00E00	0.000	8.02E-01	1.172	2.87E-02	-2.430
VIM	vimentin	9.23E-01	-1.058	7.32E-01	-1.226	9.94E-04	-1.740	2.98E-02	-1.433
WDR6	WD repeat domain 6	1.00E00	-1.574	1.00E00	0.000	2.82E-02	-1.777	3.01E-02	-2.105
YBX3	Y box binding protein 3	6.81E-01	1.320	4.39E-01	-1.621	3.77E-02	-1.300	1.55E-02	-1.410
YME1L1	YME1-like 1 ATPase	4.45E-01	-1.282	1.00E00	-1.083	1.73E-01	-1.302	3.74E-03	-2.148
YTHDF2	YTH domain family, member 2	1.00E00	1.084	1.00E00	0.000	5.38E-02	-1.811	3.83E-02	-1.567
ZMYM2	zinc finger, MYM-type 2					2.60E-03	-3.282	3.67E-03	-4.371

LMP1-no Inhibitors: Cell death of Tumor Cell lines (P = 0.00018)

		LMP1 no Inhs		LMP2 no Inhs		LMP1 with Inhs		LMP2 with Inhs	
**Symbol**	**Entrez Gene Name**	**p-value**	**Fold Change**	**p-value**	**Fold Change**	**p-value**	**Fold Change**	**p-value**	**Fold Change**
ACACA	acetyl-CoA carboxylase alpha	1.81E-02	-2.870	2.91E-01	-1.911	8.33E-01	1.051	9.42E-01	-1.014
CAPN1	calpain 1, (mu/l) large subunit	4.38E-02	-1.239	9.13E-01	1.017	1.09E-01	1.277	8.11E-01	1.037
CAPNS1	calpain, small subunit 1	1.07E-02	1.386	1.35E-01	1.316	2.97E-01	1.078	1.28E-01	-1.125
CHTOP	chromatin target of PRMT1	1.98E-02	-1.944	1.10E-02	-1.675	1.13E-01	1.392	4.44E-02	1.757
CNN2	calponin 2	3.14E-02	1.550	3.23E-01	1.388	8.13E-04	1.753	6.26E-02	1.246
EIF2AK2	eukaryotic translation initiation factor 2-alpha kinase 2	3.04E-02	-1.756	5.90E-01	1.081	5.77E-01	-1.088	3.84E-01	-1.125
EIF2B4	eukaryotic translation initiation factor 2B, subunit 4 delta, 67kDa	1.95E-02	1.233	1.00E00	1.446	3.35E-01	-1.208	5.09E-01	-1.133
ETHE1	ethylmalonic encephalopathy 1	3.43E-02	2.076	6.88E-01	1.082	2.01E-02	1.558	6.43E-01	-1.084
HK1	hexokinase 1	3.25E-03	-1.363	2.59E-01	-1.360	5.00E-02	-1.264	8.03E-01	1.025
HSPB1	heat shock 27kDa protein 1	1.20E-02	3.427	2.30E-01	1.936	2.29E-01	1.449	9.29E-01	-1.043
ILF2	interleukin enhancer binding factor 2	4.35E-02	-1.524	7.87E-01	1.098	5.75E-01	-1.092	8.51E-01	-1.031
KRT18	keratin 18	1.12E-02	-1.391	3.43E-01	-1.225	2.47E-01	1.202	3.85E-01	1.150
LIG3	ligase III, DNA, ATP-dependent	1.98E-02	-2.267	1.00E00	-1.304	2.61E-01	1.431	2.63E-01	1.433
MVP	major vault protein	1.50E-02	1.448	5.34E-01	-1.110	3.14E-04	1.512	1.29E-01	-1.259
NDRG1	N-myc downstream regulated 1	1.89E-04	2.066	3.24E-02	2.279	2.95E-01	-1.325	1.22E-01	1.474
PCNA	proliferating cell nuclear antigen	1.39E-02	-1.567	8.60E-01	-1.042	1.07E-01	-1.523	8.93E-02	-1.434
PHB	prohibitin	2.29E-02	1.435	2.84E-01	1.437	7.44E-01	1.094	4.09E-01	1.192
PRKCDBP	protein kinase C, delta binding protein	2.12E-02	2.481	3.25E-01	2.001	3.10E-01	2.533	6.45E-01	1.506
PSMD2	proteasome (prosome, macropain) 26S subunit, non-ATPase, 2	5.38E-03	1.171	1.21E-01	1.472	2.87E-01	-1.067	2.70E-02	-1.227
SERPINB5	serpin peptidase inhibitor, clade B (ovalbumin), member 5	1.15E-02	1.984	9.97E-02	1.719	5.35E-01	1.261	5.81E-01	1.299
SFN	stratifin	3.53E-02	2.029	2.01E-01	1.754	2.44E-01	-1.200	6.46E-01	-1.123
THOC1	THO complex 1	7.01E-03	-2.126	1.00E00	-2.396	3.57E-01	-1.181	8.65E-01	1.021
TOP1	topoisomerase (DNA) I	4.71E-02	-1.434	6.68E-01	-1.064	2.27E-01	-1.276	9.74E-01	1.005
TXN	thioredoxin	1.16E-02	1.420	2.42E-01	1.211	9.26E-02	1.313	5.66E-01	1.169
UBE2V1	ubiquitin-conjugating enzyme E2 variant 1	1.10E-03	1.739	9.67E-01	-1.021	5.64E-01	2.638	8.11E-01	1.516
XRCC1	X-ray repair complementing defective repair in Chinese hamster cells 1	1.26E-02	-5.060	1.00E00	0.000	8.02E-01	1.055	8.55E-01	1.061

LMP2-no Inhibitors: Cell death of Tumor Cell lines (P = 0.0433)

		LMP1 no Inhs		LMP2 no Inhs		LMP1 with Inhs		LMP2 with Inhs	
**Symbol**	**Entrez Gene Name**	**p-value**	**Fold Change**	**p-value**	**Fold Change**	**p-value**	**Fold Change**	**p-value**	**Fold Change**
CCAR2	cell cycle and apoptosis regulator 2	6.19E-01	-1.054	3.15E-02	-1.378	7.81E-01	1.024	8.25E-02	1.190
CHTOP	chromatin target of PRMT1	1.98E-02	-1.944	1.10E-02	-1.675	1.13E-01	1.392	4.44E-02	1.757
DST	dystonin	8.11E-02	5.021	3.65E-02	4.176	9.43E-01	1.041	1.43E-01	2.468
HNRNPA1	heterogeneous nuclear ribonucleoprotein A1	1.89E-01	-1.175	4.40E-02	1.292	1.79E-01	-1.311	4.20E-01	-1.220
NDRG1	N-myc downstream regulated 1	1.89E-04	2.066	3.24E-02	2.279	2.95E-01	-1.325	1.22E-01	1.474
PML	promyelocytic leukemia	4.01E-01	-1.174	2.33E-02	-2.367	7.11E-01	1.114	4.66E-02	-1.449
PRDX2	peroxiredoxin 2	1.52E-01	1.178	3.39E-02	1.213	6.38E-01	1.268	6.44E-01	1.294
S100A4	S100 calcium binding protein A4	9.45E-01	1.096	3.90E-03	7.360	3.00E-02	-1.797	1.74E-01	1.771
TPP2	tripeptidyl peptidase II	2.42E-01	-2.510	4.73E-02	-1.323	1.41E-01	-1.243	8.26E-01	1.019

LMP1-with Inhibitors: Cell death of Tumor Cell lines (P = 2.78 x 10^{-6})

		LMP1 no Inhs		LMP2 no Inhs		LMP1 with Inhs		LMP2 with Inhs	
**Symbol**	**Entrez Gene Name**	**p-value**	**Fold Change**	**p-value**	**Fold Change**	**p-value**	**Fold Change**	**p-value**	**Fold Change**
AATF	apoptosis antagonizing transcription factor	3.12E-01	-1.370	4.76E-01	1.207	3.10E-02	-1.210	4.32E-01	-1.074
ANXA2	annexin A2	6.69E-01	1.181	1.65E-01	1.204	1.96E-02	1.394	2.54E-01	1.175
APMAP	adipocyte plasma membrane associated protein	8.45E-01	1.068	7.91E-01	1.145	4.52E-02	-1.669	9.95E-01	1.001
ATP1A1	ATPase, Na+/K+ transporting, alpha 1 polypeptide	9.89E-02	-1.790	2.75E-01	-1.747	1.62E-02	-1.453	5.24E-01	-1.082
BAG1	BCL2-associated athanogene	1.00E00	0.000	1.00E00	0.000	2.96E-03	2.613	9.49E-01	1.028
BAG3	BCL2-associated athanogene 3	3.91E-01	1.292	1.46E-01	1.807	1.74E-03	1.712	2.66E-01	-1.176
BCLAF1	BCL2-associated transcription factor 1	2.45E-01	1.432	8.27E-01	-1.070	1.38E-02	-1.654	8.10E-01	1.037
BIRC3	baculoviral IAP repeat containing 3	1.00E00	0.000	1.00E00	0.000	1.58E-02	-1.760	6.50E-01	-1.027

CAMK2G	calcium/calmodulin-dependent protein kinase II gamma	1.00E00	-1.864	1.00E00	1.149	1.04E-02	2.462	8.26E-01	1.060
CASP1	caspase 1, apoptosis-related cysteine peptidase	1.00E00	1.079	1.57E-01	-1.776	3.91E-02	1.376	4.70E-01	-1.299
CAST	calpastatin	5.69E-01	1.357	1.59E-01	2.535	6.79E-03	1.556	2.02E-01	1.279
CCT8	chaperonin containing TCP1, subunit 8 (theta)	4.11E-01	-1.253	9.92E-01	1.002	9.35E-03	-1.291	4.24E-01	1.079
CD44	CD44 molecule (Indian blood group)	4.40E-01	-2.277	1.34E-01	-3.127	5.16E-03	-1.316	4.93E-01	-1.089
CD99	CD99 molecule	1.00E00	0.000	1.00E00	0.000	1.94E-02	2.356	2.42E-01	1.329
CDK5	cyclin-dependent kinase 5	1.00E00	0.000	1.00E00	0.000	1.64E-02	-1.426	2.70E-01	1.029
CIB1	calcium and integrin binding 1 (calmyrin)					1.50E-02	3.427	1.00E00	1.468
CNN2	calponin 2	3.14E-02	1.550	3.23E-01	1.388	8.13E-04	1.753	6.26E-02	1.246
CNPY2	canopy FGF signaling regulator 2	3.30E-01	-1.142	1.91E-01	-1.227	4.87E-02	1.708	8.21E-02	1.679
CRABP2	cellular retinoic acid binding protein 2	1.00E00	0.000	1.00E00	0.000	2.01E-02	10.196	5.80E-01	1.753
CTBP1	C-terminal binding protein 1	5.49E-01	-1.175	5.46E-01	1.181	3.58E-02	-1.525	2.73E-01	-1.224
CTSB	cathepsin B	9.68E-02	1.528	9.13E-01	1.055	1.55E-02	1.920	1.58E-01	1.368
CUL1	cullin 1	1.00E00	-3.027	1.00E00	2.030	3.57E-03	-1.414	9.59E-01	-1.002
CYFIP2	cytoplasmic FMR1 interacting protein 2					4.71E-02	-1.275	1.42E-01	-3.469
DDX58	DEAD (Asp-Glu-Ala-Asp) box polypeptide 58					2.50E-02	4.906	9.74E-01	-1.016
EIF2A	eukaryotic translation initiation factor 2A, 65kDa	9.33E-01	-1.021	3.74E-01	1.287	2.67E-02	-1.316	5.59E-02	-1.223
EIF2S1	eukaryotic translation initiation factor 2, subunit 1 alpha, 35kDa	1.87E-01	1.365	7.04E-01	1.067	2.93E-03	-1.354	7.24E-01	-1.028
ENO1	enolase 1, (alpha)	3.39E-01	1.109	1.62E-01	1.484	6.46E-03	-2.532	8.54E-03	-2.414
ETHE1	ethylmalonic encephalopathy 1	3.43E-02	2.076	6.88E-01	1.082	2.01E-02	1.558	6.43E-01	-1.084
EZR	ezrin	4.70E-01	1.533	6.72E-01	1.354	3.55E-02	2.326	9.76E-01	-1.012
FAP	fibroblast activation protein, alpha					2.21E-02	13.090	1.00E00	
FKBP5	FK506 binding protein 5	5.84E-01	-1.202	5.13E-01	1.276	3.74E-03	-1.378	2.37E-01	1.083
FLNB	filamin B, beta	1.24E-01	1.607	6.49E-01	1.104	3.33E-02	1.281	1.84E-01	-3.752
FXN	frataxin	1.00E00	0.000	1.00E00	0.000	3.71E-04	-1.877	1.48E-01	-1.835
G6PD	glucose-6-phosphate dehydrogenase	4.85E-01	-1.477	8.33E-01	-1.124	4.02E-02	-1.406	8.21E-01	-1.038
GBE1	glucan (1,4-alpha-), branching enzyme 1	9.21E-01	1.035	2.90E-01	1.178	9.30E-03	-1.472	1.42E-02	-1.254
GLRX	glutaredoxin (thioltransferase)	1.00E00	1.035	1.00E00	-1.986	3.26E-02	2.976	2.46E-01	-1.715
GPX1	glutathione peroxidase 1	5.95E-01	1.428	6.56E-01	1.449	1.46E-02	1.273	1.51E-01	1.193
GRN	granulin					7.46E-04	1.284	2.37E-01	1.124
GSN	gelsolin	4.22E-01	1.945	1.00E00	-1.706	2.18E-03	1.703	1.23E-01	-1.197
HDAC2	histone deacetylase 2	1.00E00	-3.841	1.00E00	-1.241	4.63E-02	-1.220	4.45E-01	-1.123
HNRNPK	heterogeneous nuclear ribonucleoprotein K	6.23E-01	1.079	6.77E-01	1.057	1.97E-02	-1.205	3.08E-01	-2.032
HSPA8	heat shock 70kDa protein 8	7.91E-01	1.055	3.99E-01	1.176	4.62E-02	-2.934	5.98E-01	2.678
HSPA1A/HS	heat shock 70kDa protein 1A	1.43E-01	1.284	3.50E-01	1.333	2.34E-03	-1.242	1.93E-01	-1.064
IFI16	interferon, gamma-inducible protein 16	9.19E-01	-1.030	3.01E-01	-1.441	4.97E-02	1.281	9.11E-01	1.012
IKBKG	inhibitor of kappa light polypeptide gene enhancer in B-cells, kinase gamma	1.00E00	1.673	1.00E00	1.769	5.03E-03	1.742	2.19E-01	1.327
IL18	interleukin 18	3.74E-01	-1.508	6.32E-01	-1.222	5.63E-03	1.981	1.43E-03	1.372
IL1RN	interleukin 1 receptor antagonist	1.00E00	0.000	1.00E00	0.000	4.59E-02	5.796	2.60E-01	3.522
ILKAP	integrin-linked kinase-associated serine/threonine phosphatase	1.00E00	-1.000	1.00E00	-1.490	4.27E-02	-1.589	2.27E-01	-1.253
ITGA5	integrin, alpha 5 (fibronectin receptor, alpha polypeptide)	3.64E-01	-2.172	1.00E00	-3.726	4.33E-02	-2.098	2.27E-02	-2.119
ITGB4	integrin, beta 4	5.06E-01	-1.180	7.55E-01	-1.092	3.98E-02	-1.164	1.00E00	-1.292
KEAP1	kelch-like ECH-associated protein 1	1.00E-01	1.388	1.00E00	0.000	4.76E-02	1.310	1.49E-01	-1.211
KIF1C	kinesin family member 1C	1.00E00	1.102	1.00E00	0.000	1.49E-02	1.998	5.97E-01	1.141
LAMA5	laminin, alpha 5	2.69E-01	-2.706	1.00E00	-3.277	1.45E-02	-1.401	4.71E-02	-2.294
LETM1	leucine zipper-EF-hand containing transmembrane protein 1	4.86E-01	1.262	8.10E-01	1.134	3.56E-02	1.305	3.12E-01	1.164
LGALS3	lectin, galactoside-binding, soluble, 3	2.19E-01	1.602	5.08E-01	-1.695	1.83E-02	2.188	6.11E-01	1.139
MED21	mediator complex subunit 21	1.00E00	0.000	1.00E00	0.000	1.37E-02	1.604	3.51E-01	1.245
MET	MET proto-oncogene, receptor tyrosine kinase	1.00E00	0.000	1.00E00	0.000	4.99E-02	-1.277	6.91E-01	1.046
MVP	major vault protein	1.50E-02	1.448	5.34E-01	-1.110	3.14E-04	1.512	1.29E-01	-1.259
MYO6	myosin VI	7.07E-01	1.104	2.96E-01	-1.384	2.53E-02	1.468	7.71E-02	-1.689
PACS2	phosphofurin acidic cluster sorting protein 2	1.00E00	0.000	1.00E00	0.000	4.29E-02	1.448	1.00E00	

PAK1	p21 protein (Cdc42/Rac)-activated kinase 1					3.34E-02	-1.594	1.00E00	
PARP1	poly (ADP-ribose) polymerase 1	2.31E-01	-1.364	4.50E-01	-1.262	2.46E-03	-1.234	3.58E-01	1.134
PAWR	PRKC, apoptosis, WT1, regulator	1.00E00	1.098	1.00E00	0.000	7.64E-04	2.685	1.95E-01	1.444
PCBP2	poly(rC) binding protein 2	8.84E-01	1.065	5.91E-01	-1.373	3.77E-02	1.196	1.54E-01	-2.347
PDE4D	phosphodiesterase 4D, cAMP-specific	1.00E00	0.000	1.00E00	0.000	7.98E-03	-3.140	1.15E-01	-1.516
PGRMC1	progesterone receptor membrane component 1	8.46E-01	1.168	3.56E-01	-2.648	1.94E-02	-1.380	4.82E-02	-1.261
PKM	pyruvate kinase, muscle	5.43E-01	-1.856	1.00E00	-1.791	4.20E-02	-1.686	2.55E-01	-1.288
PLA2G4A	phospholipase A2, group IVA (cytosolic, calcium-dependent)	1.00E00	0.000	1.00E00	0.000	4.44E-02	-1.379	3.22E-01	1.211
PPAT	phosphoribosyl pyrophosphate amidotransferase	5.21E-01	-1.316	4.20E-01	-1.097	4.83E-02	1.675	2.37E-01	1.608
PPID	peptidylprolyl isomerase D	1.43E-01	-1.434	1.18E-01	-1.530	3.17E-02	-1.441	6.87E-01	-1.041
PPP1R10	protein phosphatase 1, regulatory subunit 10	1.00E00	-1.016	1.00E00	1.139	2.36E-02	-1.314	6.81E-02	-1.247
PSMD14	proteasome (prosome, macropain) 26S subunit, non-ATPase, 14	8.70E-01	-1.028	9.65E-01	1.043	4.95E-02	-1.390	4.85E-01	-1.234
RAP1GDS1	RAP1, GTP-GDP dissociation stimulator 1	7.18E-01	-1.186	8.78E-01	-1.036	1.16E-03	-2.307	2.87E-02	-2.073
RBX1	ring-box 1, E3 ubiquitin protein ligase	1.00E00	0.000	1.00E00	0.000	2.30E-02	-1.494	7.36E-01	-1.081
S100A4	S100 calcium binding protein A4	9.45E-01	1.096	3.90E-03	7.360	3.00E-02	-1.797	1.74E-01	1.771
S100A11	S100 calcium binding protein A11	7.07E-01	-1.259	7.38E-01	-1.312	1.84E-03	1.639	6.48E-02	1.255
SLPI	secretory leukocyte peptidase inhibitor					1.03E-02	9.998	3.86E-01	1.397
SND1	staphylococcal nuclease and tudor domain containing 1	4.51E-01	-1.173	7.43E-01	1.075	2.77E-02	-1.319	3.66E-01	-1.094
SOD1	superoxide dismutase 1, soluble	4.63E-01	2.670	7.65E-01	1.356	1.68E-02	2.011	6.23E-01	-1.434
SVIL	supervillin	9.52E-01	1.056	1.00E00	-1.227	2.40E-04	2.199	3.06E-01	1.326
SYNGR2	synaptogyrin 2					2.59E-03	1.885	4.98E-01	1.113
TBK1	TANK-binding kinase 1	1.00E00	1.058	1.00E00	0.000	4.25E-02	-1.305	3.19E-01	-1.093
TCEB1	transcription elongation factor B (SIII), polypeptide 1 (15kDa, elongin C)	6.52E-01	1.202	7.71E-01	1.235	4.49E-02	-1.414	8.04E-01	-1.028
TCP1	t-complex 1	2.91E-01	-1.347	8.89E-01	1.042	2.03E-02	-1.191	5.97E-01	1.028
TIMP1	TIMP metallopeptidase inhibitor 1	8.12E-01	1.079	1.00E00	-1.174	1.98E-02	1.741	9.21E-01	1.008
TIMP3	TIMP metallopeptidase inhibitor 3	1.00E00	-2.697	1.00E00	0.000	4.13E-03	-1.852	4.38E-01	-1.628
TLR3	toll-like receptor 3					4.57E-02	-1.300	4.35E-02	-2.468
TMSB10/TM	thymosin beta 10	2.63E-01	3.370	2.81E-01	1.836	3.77E-02	5.288	7.55E-01	-1.269
TPD52	tumor protein D52	3.64E-01	-1.420	1.00E00	0.000	4.08E-02	2.962	3.36E-01	1.648
TXNRD1	thioredoxin reductase 1	5.04E-01	1.560	9.93E-01	1.012	2.08E-02	-2.032	9.20E-02	-1.882
UBE2K	ubiquitin-conjugating enzyme E2K	5.17E-01	-1.320	6.95E-01	1.205	1.86E-03	-1.490	4.19E-01	-1.069
UBE2L3	ubiquitin-conjugating enzyme E2L 3	9.58E-01	-1.053	9.55E-01	-1.079	1.42E-02	4.031	2.62E-01	1.810
XAB2	XPA binding protein 2	1.00E00	1.029	1.00E00	1.851	2.07E-02	-1.513	2.66E-01	-1.533

	LMP2-with Inhibitors: Cell Death of Tumor Cell lines (P = 0.0151)	LMP1 no Inhs		LMP2 no Inhs		LMP1 with Inhs		LMP2 with Inhs	
**Symbol**	**Entrez Gene Name**	**p-value**	**Fold Change**	**p-value**	**Fold Change**	**p-value**	**Fold Change**	**p-value**	**Fold Change**
API5	apoptosis inhibitor 5	6.53E-01	1.114	7.95E-01	1.234	8.46E-02	-1.360	1.87E-02	-1.382
ATF1	activating transcription factor 1					5.54E-01	1.108	3.67E-02	1.330
BCL2L11	BCL2-like 11 (apoptosis facilitator)					2.64E-01	-2.048	3.15E-02	-1.593
CBR1	carbonyl reductase 1	9.75E-01	-1.004	1.50E-01	1.242	5.81E-01	1.061	1.39E-02	1.397
CDC34	cell division cycle 34					8.61E-01	-1.070	3.99E-02	-2.274
CDKN1C	cyclin-dependent kinase inhibitor 1C (p57, Kip2)	1.00E00	0.000	1.00E00	0.000	2.48E-01	1.536	4.13E-03	-1.767
CEBPD	CCAAT/enhancer binding protein (C/EBP), delta	9.02E-02	-1.547	2.94E-01	-1.458	6.40E-01	1.213	3.42E-02	1.828
CENPB	centromere protein B, 80kDa	1.00E00	0.000	1.00E00	0.000	7.29E-01	1.061	8.70E-03	-1.487
CHTOP	chromatin target of PRMT1	1.98E-02	-1.944	1.10E-02	-1.675	1.13E-01	1.392	4.44E-02	1.757
CLCA2	chloride channel accessory 2	3.87E-01	-1.357	1.00E00	-2.046	7.82E-01	-1.084	1.99E-02	2.104
CYR61	cysteine-rich, angiogenic inducer, 61	1.00E00	0.000	1.00E00	0.000	4.67E-01	-1.418	9.40E-03	-2.249
DAPK3	death-associated protein kinase 3	1.00E00	0.000	1.00E00	0.000	2.18E-01	1.285	4.16E-02	-1.518
DHCR24	24-dehydrocholesterol reductase	1.00E00	0.000	1.00E00	0.000	2.07E-01	1.410	7.50E-03	-1.884
DUT	deoxyuridine triphosphatase	2.13E-01	-1.391	5.59E-01	-1.146	8.93E-02	-1.673	3.18E-03	-1.737
EI24	etoposide induced 2.4	1.00E00	-3.013	1.00E00	0.000	9.20E-01	1.041	4.92E-02	-2.038
ENO1	enolase 1, (alpha)	3.39E-01	1.109	1.62E-01	1.484	6.46E-03	-2.532	8.54E-03	-2.414

FAS	Fas cell surface death receptor	1.00E00	-1.102	1.00E00	0.000	1.24E-01	-1.230	3.08E-03	-1.720
GBE1	glucan (1,4-alpha-), branching enzyme 1	9.21E-01	1.035	2.90E-01	1.178	9.30E-03	-1.472	1.42E-02	-1.254
H2AFX	H2A histone family, member X	8.16E-01	-1.078	9.95E-01	1.009	8.35E-01	1.046	4.72E-02	1.489
HMMR	hyaluronan-mediated motility receptor (RHAMM)	1.00E00	0.000	1.00E00	0.000	1.00E00	-3.729	2.60E-02	-3.481
IL18	interleukin 18	3.74E-01	-1.508	6.32E-01	-1.222	5.63E-03	1.981	1.43E-03	1.372
ITGA5	integrin, alpha 5 (fibronectin receptor, alpha polypeptide)	3.64E-01	-2.172	1.00E00	-3.726	4.33E-02	-2.098	2.27E-02	-2.119
KANK2	KN motif and ankyrin repeat domains 2	1.00E00	0.000	1.00E00	0.000	8.80E-02	-1.928	6.18E-03	-2.708
LAMA5	laminin, alpha 5	2.69E-01	-2.706	1.00E00	-3.277	1.45E-02	-1.401	4.71E-02	-2.294
MAN2C1	mannosidase, alpha, class 2C, member 1	1.00E00	0.000	1.00E00	0.000	2.67E-01	-1.161	3.69E-02	-2.514
MBD1	methyl-CpG binding domain protein 1	1.00E00	0.000	1.00E00	0.000	3.34E-01	-1.117	2.46E-03	-1.734
MCM7	minichromosome maintenance complex component 7	2.36E-01	-1.658	1.82E-01	-1.311	1.73E-01	-1.253	4.74E-02	-1.355
MSI2	musashi RNA-binding protein 2	1.00E00	1.317	1.00E00	1.552	1.00E00	-1.322	1.95E-02	-1.841
MT1X	metallothionein 1X					6.06E-01	1.160	9.25E-03	2.307
NFKB1	nuclear factor of kappa light polypeptide gene enhancer in B-cells 1	7.54E-01	-1.280	8.35E-01	1.177	9.42E-02	1.205	1.63E-02	1.226
NPM1	nucleophosmin (nucleolar phosphoprotein B23, numatrin)	3.04E-01	-1.476	1.10E-01	-1.239	2.68E-01	-1.670	1.58E-02	1.215
OGT	O-linked N-acetylglucosamine (GlcNAc) transferase	4.88E-01	1.135	9.90E-01	1.007	9.40E-01	1.015	2.70E-02	-1.424
PDCD6IP	programmed cell death 6 interacting protein	1.16E-01	1.678	5.08E-01	1.196	1.39E-01	-1.067	2.27E-02	-1.149
PDLIM7	PDZ and LIM domain 7 (enigma)	2.42E-01	1.589	4.20E-01	-1.346	7.52E-01	-1.132	5.62E-03	-1.875
PGRMC1	progesterone receptor membrane component 1	8.46E-01	1.168	3.56E-01	-2.648	1.94E-02	-1.380	4.82E-02	-1.261
PIAS3	protein inhibitor of activated STAT, 3					9.02E-02	4.761	1.35E-02	6.194
PML	promyelocytic leukemia	4.01E-01	-1.174	2.33E-02	-2.367	7.11E-01	1.114	4.66E-02	-1.449
PPP1R13L	protein phosphatase 1, regulatory subunit 13 like	4.18E-01	1.766	3.18E-01	-1.383	3.89E-01	1.170	4.07E-02	-1.352
PPP2R2A	protein phosphatase 2, regulatory subunit B, alpha	4.81E-01	1.459	8.73E-01	1.147	2.85E-01	-1.232	7.48E-03	-1.358
PSIP1	PC4 and SFRS1 interacting protein 1	4.98E-01	-1.186	1.00E00	1.241	1.30E-01	-1.136	4.78E-03	-1.159
PSMD2	proteasome (prosome, macropain) 26S subunit, non-ATPase, 2	5.38E-03	1.171	1.21E-01	1.472	2.87E-01	-1.067	2.70E-02	-1.227
RAD9A	RAD9 homolog A (S. pombe)	1.00E00	0.000	1.00E00	0.000	3.19E-01	-1.244	3.39E-02	-1.702
RAP1GDS1	RAP1, GTP-GDP dissociation stimulator 1	7.18E-01	-1.186	8.78E-01	-1.036	1.16E-03	-2.307	2.87E-02	-2.073
RIPK1	receptor (TNFRSF)-interacting serine-threonine kinase 1	1.00E00	0.000	1.00E00	0.000	3.61E-01	1.444	3.39E-03	-1.610
RPS6KB1	ribosomal protein S6 kinase, 70kDa, polypeptide 1	1.00E00	1.861	1.00E00	1.983	1.04E-01	-1.675	3.62E-02	-1.878
RTN4	reticulon 4	7.07E-01	-1.774	1.00E00	-1.053	6.29E-02	1.171	8.41E-03	-1.397
SLC3A2	solute carrier family 3 (amino acid transporter heavy chain), member 2	1.88E-01	-1.396	9.50E-01	-1.051	3.39E-01	-1.430	3.13E-02	1.445
SMAD2	SMAD family member 2	9.07E-01	-1.022	1.00E00	-1.036	3.94E-01	1.118	3.74E-02	-1.304
STIM1	stromal interaction molecule 1	1.00E00	0.000	1.00E00	0.000	4.59E-01	1.167	3.48E-02	-1.984
TGM2	transglutaminase 2	1.00E00	1.933	1.00E00	0.000	5.35E-01	1.128	1.79E-03	-3.500
TLR3	toll-like receptor 3					4.57E-02	-1.300	4.35E-02	-2.468
TPX2	TPX2, microtubule-associated					7.32E-02	-7.719	3.53E-02	-19.498
TRAF6	TNF receptor-associated factor 6, E3 ubiquitin protein ligase	1.00E00	0.000	1.00E00	0.000	1.29E-01	-1.539	5.71E-03	-1.943
TRAP1	TNF receptor-associated protein 1	3.42E-01	-1.458	8.55E-01	-1.022	2.57E-01	-1.143	2.79E-02	1.389
UBE2C	ubiquitin-conjugating enzyme E2C	1.00E00	0.000	1.00E00	0.000	1.91E-01	-1.937	1.21E-02	-5.242

LMP1-no Inhibitors: mTOR signaling (P = 0.307)

Symbol	Entrez Gene Name	LMP1 no Inhs p-value	LMP1 no Inhs Fold Change	LMP2 no Inhs p-value	LMP2 no Inhs Fold Change	LMP1 with Inhs p-value	LMP1 with Inhs Fold Change	LMP2 with Inhs p-value	LMP2 with Inhs Fold Change
EIF3L	eukaryotic translation initiation factor 3, subunit L	7.50E-04	-1.578	3.03E-01	-1.421	1.52E-01	-1.223	1.01E-01	-1.191
EIF4A1	eukaryotic translation initiation factor 4A1	2.22E-02	-1.494	6.00E-01	-1.108	5.36E-03	-1.474	2.21E-03	-1.518

LMP2-no Inhibitors: mTOR signaling (P = 0.351)

Symbol	Entrez Gene Name	LMP1 no Inhs p-value	LMP1 no Inhs Fold Change	LMP2 no Inhs p-value	LMP2 no Inhs Fold Change	LMP1 with Inhs p-value	LMP1 with Inhs Fold Change	LMP2 with Inhs p-value	LMP2 with Inhs Fold Change
RPS25	ribosomal protein S25	1.52E-01	1.14	3.99E-02	1.423	8.54E-01	-1.043	4.58E-01	-1.207

LMP1-with Inhibitors: mTOR signaling (P = 0.271)

Symbol	Entrez Gene Name	LMP1 no Inhs p-value	LMP1 no Inhs Fold Change	LMP2 no Inhs p-value	LMP2 no Inhs Fold Change	LMP1 with Inhs p-value	LMP1 with Inhs Fold Change	LMP2 with Inhs p-value	LMP2 with Inhs Fold Change
EIF3B	eukaryotic translation initiation factor 3, subunit B	3.42E-01	-1.386	8.48E-01	1.074	1.02E-02	-1.676	2.12E-03	-1.435
EIF3C	eukaryotic translation initiation factor 3, subunit C	5.73E-01	-1.271	7.02E-01	-1.177	9.18E-03	-1.354	1.15E-01	-1.17
EIF3E	eukaryotic translation initiation factor 3, subunit E	2.79E-01	1.27	5.54E-01	-1.191	4.88E-02	-1.981	2.96E-02	-1.483
EIF4A1	eukaryotic translation initiation factor 4A1	2.22E-02	-1.494	6.00E-01	-1.108	5.36E-03	-1.474	2.21E-03	-1.518
EIF4G3	eukaryotic translation initiation factor 4 gamma, 3	1.00E+00	-1.319	1.00E+00	1.355	2.49E-02	1.703	1.09E-01	-1.517
RHOT1	ras homolog family member T1	1.00E+00	1.452	1.00E+00	-1.341	4.97E-02	2.05	6.21E-01	1.195
RPS6KA4	ribosomal protein S6 kinase, 90kDa, polypeptide 4	1.00E+00	-1.026	1.00E+00	-1.418	2.13E-02	-1.624	1.13E-01	-1.257

LMP2-with Inhibitors: mTOR signaling (P = 0.0013)

Symbol	Entrez Gene Name	LMP1 no Inhs p-value	LMP1 no Inhs Fold Change	LMP2 no Inhs p-value	LMP2 no Inhs Fold Change	LMP1 with Inhs p-value	LMP1 with Inhs Fold Change	LMP2 with Inhs p-value	LMP2 with Inhs Fold Change
EIF3B	eukaryotic translation initiation factor 3, subunit B	3.42E-01	-1.386	8.48E-01	1.074	1.02E-02	-1.676	2.12E-03	-1.435
EIF3E	eukaryotic translation initiation factor 3, subunit E	2.79E-01	1.27	5.54E-01	-1.191	4.88E-02	-1.981	2.96E-02	-1.483
EIF3K	eukaryotic translation initiation factor 3, subunit K	4.74E-01	-1.416	6.92E-01	-1.129	3.06E-01	-1.21	4.08E-02	-1.481
EIF3M	eukaryotic translation initiation factor 3, subunit M	8.12E-01	-1.073	1.13E-01	-1.587	2.70E-01	-1.202	4.54E-02	-1.495
EIF4A1	eukaryotic translation initiation factor 4A1	2.22E-02	-1.494	6.00E-01	-1.108	5.36E-03	-1.474	2.21E-03	-1.518
EIF4A2	eukaryotic translation initiation factor 4A2	1.00E+00	0	1.00E+00	0	5.80E-01	1.224	4.97E-02	-2.874
PPP2R2A	protein phosphatase 2, regulatory subunit B, alpha	4.81E-01	1.459	8.73E-01	1.147	2.85E-01	-1.232	7.48E-03	-1.358
RHEB	Ras homolog enriched in brain	1.00E+00	0	1.00E+00	0	4.60E-01	-1.64	3.30E-02	-1.975
RPS27	ribosomal protein S27	8.41E-01	1.058	2.67E-01	-2.025	4.95E-01	1.154	3.28E-02	1.64
RPS15A	ribosomal protein S15a	6.98E-02	-1.702	3.15E-01	-1.182	9.19E-01	-1.093	2.40E-03	1.238
RPS6KB1	ribosomal protein S6 kinase, 70kDa, polypeptide 1	1.00E+00	1.861	1.00E+00	1.983	1.04E-01	-1.675	3.62E-02	-1.878

Symbol	LMP1-no Inhibitors: PI3K/AKT signaling (P = 0.523)	LMP1 no Inhs		LMP2 no Inhs		LMP1 with Inhs		LMP2 with Inhs	
Symbol	Entrez Gene Name	p-value	Fold Change	p-value	Fold Change	p-value	Fold Change	p-value	Fold Change
SFN	stratifin	3.53E-02	2.029	2.01E-01	1.754	2.44E-01	-1.2	6.46E-01	-1.123

	LMP2-no Inhibitors: PI3K/AKT signaling (P = 1)	LMP1 no Inhs	LMP2 no Inhs	LMP1 with Inhs	LMP2 with Inhs
none					

	LMP1 with Inhibitors: PI3K/AKT signaling (P = 0.264)	LMP1 no Inhs		LMP2 no Inhs		LMP1 with Inhs		LMP2 with Inhs	
Symbol	Entrez Gene Name	p-value	Fold Change	p-value	Fold Change	p-value	Fold Change	p-value	Fold Change
HSP90AA1	heat shock protein 90kDa alpha (cytosolic), class A member 1	6.54E-01	-1.196	9.28E-01	-1.024	3.43E-02	-1.335	1.36E-01	-1.143
IKBKG	inhibitor of kappa light polypeptide gene enhancer in B-cells, kinase gamma	1.00E+00	1.673	1.00E+00	1.769	5.03E-03	1.742	2.19E-01	1.327
ITGA5	integrin, alpha 5 (fibronectin receptor, alpha polypeptide)	3.64E-01	-2.172	1.00E+00	-3.726	4.33E-02	-2.098	2.27E-02	-2.119
MAP2K2	mitogen-activated protein kinase kinase 2	1.00E+00	-1.03	1.00E+00	1.079	2.57E-02	-1.397	2.02E-02	-1.306
THEM4	thioesterase superfamily member 4	1.00E+00	-1.251	1.00E+00	1.066	5.45E-03	-1.605	6.83E-01	-1.141

	LMP2 with Inhibitors: PI3K/AKT signaling (P = 0.003)	LMP1 no Inhs		LMP2 no Inhs		LMP1 with Inhs		LMP2 with Inhs	
Symbol	Entrez Gene Name	p-value	Fold Change	p-value	Fold Change	p-value	Fold Change	p-value	Fold Change
INPP5K	inositol polyphosphate-5-phosphatase K					3.88E-01	1.251	3.46E-02	-1.27
ITGA5	integrin, alpha 5 (fibronectin receptor, alpha polypeptide)	3.64E-01	-2.172	1.00E+00	-3.726	4.33E-02	-2.098	2.27E-02	-2.119
MAP2K2	mitogen-activated protein kinase kinase 2	1.00E+00	-1.03	1.00E+00	1.079	2.57E-02	-1.397	2.02E-02	-1.306
NFKB1	nuclear factor of kappa light polypeptide gene enhancer in B-cells 1	7.54E-01	-1.28	8.35E-01	1.177	9.42E-02	1.205	1.63E-02	1.226
PPP2R2A	protein phosphatase 2, regulatory subunit B, alpha	4.81E-01	1.459	8.73E-01	1.147	2.85E-01	-1.232	7.48E-03	-1.358
RHEB	Ras homolog enriched in brain	1.00E+00	0	1.00E+00	0	4.60E-01	-1.64	3.30E-02	-1.975
RPS6KB1	ribosomal protein S6 kinase, 70kDa, polypeptide 1	1.00E+00	1.861	1.00E+00	1.983	1.04E-01	-1.675	3.62E-02	-1.878
SYNJ2	synaptojanin 2	1.00E+00	0	1.00E+00	0	2.15E-01	1.608	4.39E-02	1.937

LMP1-no Inhibitors: PTEN signaling (P = 1)

		LMP1 no Inhs		LMP2 no Inhs		LMP1 with Inhs		LMP2 with Inhs	

none

LMP2-no Inhibitors: PTEN signaling (P = 1)

		LMP1 no Inhs		LMP2 no Inhs		LMP1 with Inhs		LMP2 with Inhs	

none

LMP1-with Inhibitors: PTEN signaling (P = 0.407)

		LMP1 no Inhs		LMP2 no Inhs		LMP1 with Inhs		LMP2 with Inhs	
**Symbol**	**Entrez Gene Name**	**p-value**	**Fold Change**	**p-value**	**Fold Change**	**p-value**	**Fold Change**	**p-value**	**Fold Change**
IKBKG	inhibitor of kappa light polypeptide gene enhancer in B-cells, kinase gamma	1.00E+00	1.673	1.00E+00	1.769	5.03E-03	1.742	2.19E-01	1.327
ITGA5	integrin, alpha 5 (fibronectin receptor, alpha polypeptide)	3.64E-01	-2.172	1.00E+00	-3.726	4.33E-02	-2.098	2.27E-02	-2.119
MAP2K2	mitogen-activated protein kinase kinase 2	1.00E+00	-1.03	1.00E+00	1.079	2.57E-02	-1.397	2.02E-02	-1.306
RAC2	ras-related C3 botulinum toxin substrate 2 (rho family, small GTP binding protein Rac2)	1.00E+00	0	1.00E+00	0	1.04E-03	1.399	7.67E-02	-1.21

LMP2-with Inhibitors: PTEN signaling (P = 0.008)

		LMP1 no Inhs		LMP2 no Inhs		LMP1 with Inhs		LMP2 with Inhs	
**Symbol**	**Entrez Gene Name**	**p-value**	**Fold Change**	**p-value**	**Fold Change**	**p-value**	**Fold Change**	**p-value**	**Fold Change**
BCL2L11	BCL2-like 11 (apoptosis facilitator)					2.64E-01	-2.048	3.15E-02	-1.593
INPP5K	inositol polyphosphate-5-phosphatase K					3.88E-01	1.251	3.46E-02	-1.27
ITGA5	integrin, alpha 5 (fibronectin receptor, alpha polypeptide)	3.64E-01	-2.172	1.00E+00	-3.726	4.33E-02	-2.098	2.27E-02	-2.119
MAP2K2	mitogen-activated protein kinase kinase 2	1.00E+00	-1.03	1.00E+00	1.079	2.57E-02	-1.397	2.02E-02	-1.306
NFKB1	nuclear factor of kappa light polypeptide gene enhancer in B-cells 1	7.54E-01	-1.28	8.35E-01	1.177	9.42E-02	1.205	1.63E-02	1.226
RPS6KB1	ribosomal protein S6 kinase, 70kDa, polypeptide 1	1.00E+00	1.861	1.00E+00	1.983	1.04E-01	-1.675	3.62E-02	-1.878
SYNJ2	synaptojanin 2	1.00E+00	0	1.00E+00	0	2.15E-01	1.608	4.39E-02	1.937

LMP1-no Inhibitors: NFkB signaling (P = 1)

		LMP1 no Inhs		LMP2 no Inhs		LMP1 with Inhs		LMP2 with Inhs	
**Symbol**	**Entrez Gene Name**	**p-value**	**Fold Change**	**p-value**	**Fold Change**	**p-value**	**Fold Change**	**p-value**	**Fold Change**
EIF2AK2	eukaryotic translation initiation factor 2-alpha kinase 2	3.04E-02	-1.756						
UBE2V1	ubiquitin-conjugating enzyme E2 variant 1	1.10E-03	1.739						

LMP2-no Inhibitors: NFkB signaling (P = 1)

		LMP1 no Inhs	LMP2 no Inhs	LMP1 with Inhs	LMP2 with Inhs
none					

LMP1-with Inhibitors: NFkB signaling (P = 4.27×10^{-4})

		LMP1 no Inhs		LMP2 no Inhs		LMP1 with Inhs		LMP2 with Inhs	
**Symbol**	**Entrez Gene Name**	**p-value**	**Fold Change**	**p-value**	**Fold Change**	**p-value**	**Fold Change**	**p-value**	**Fold Change**
HDAC2	histone deacetylase 2					4.63E-02	-1.22		
IKBKG	inhibitor of kappa light polypeptide gene enhancer in B-cells, kinase gamma					5.03E-03	1.742		
IL18	interleukin 18					5.63E-03	1.981	1.43E-03	1.372
TAB3	TGF-beta activated kinase 1/MAP3K7 binding protein 3					4.07E-02	-2.665		
TBK1	TANK-binding kinase 1					4.25E-02	-1.305		
TLR3	toll-like receptor 3					4.57E-02	-1.3	4.35E-02	-2.468

LMP2-with Inhibitors: NFkB signaling (P = 1.48×10^{-4})

		LMP1 no Inhs		LMP2 no Inhs		LMP1 with Inhs		LMP2 with Inhs	
**Symbol**	**Entrez Gene Name**	**p-value**	**Fold Change**	**p-value**	**Fold Change**	**p-value**	**Fold Change**	**p-value**	**Fold Change**
ITGA5	integrin, alpha 5 (fibronectin receptor, alpha polypeptide)					4.33E-02	-2.098	2.27E-02	-2.119
NFKB1	nuclear factor of kappa light polypeptide gene enhancer in B-cells 1							1.63E-02	1.226
RIPK1	receptor (TNFRSF)-interacting serine-threonine kinase 1							3.39E-03	-1.61

LMP2-with Inhibitors: NFkB signaling (Manual curation)

		LMP1 no Inhs		LMP2 no Inhs		LMP1 with Inhs		LMP2 with Inhs	
**Symbol**	**Entrez Gene Name**	**p-value**	**Fold Change**	**p-value**	**Fold Change**	**p-value**	**Fold Change**	**p-value**	**Fold Change**
NKAP	NFkB activating protein							3.79E-02	1.52

LMP1-no Inhibitors: NFkB Activation by Viruses (P = 1)

		LMP1 no Inhs		LMP2 no Inhs		LMP1 with Inhs		LMP2 with Inhs	
**Symbol**	**Entrez Gene Name**	**p-value**	**Fold Change**	**p-value**	**Fold Change**	**p-value**	**Fold Change**	**p-value**	**Fold Change**
EIF2AK2	eukaryotic translation initiation factor 2-alpha kinase 2	3.04E-02	-1.756						

LMP2-no Inhibitors: NFkB Activation by Viruses (P = 1)

		LMP1 no Inhs		LMP2 no Inhs		LMP1 with Inhs		LMP2 with Inhs	
none									

LMP1-with Inhibitors: NFkB Activation by Viruses (P = 4.95 x 10^{-8})

		LMP1 no Inhs		LMP2 no Inhs		LMP1 with Inhs		LMP2 with Inhs	
**Symbol**	**Entrez Gene Name**	**p-value**	**Fold Change**	**p-value**	**Fold Change**	**p-value**	**Fold Change**	**p-value**	**Fold Change**
IKBKG	inhibitor of kappa light polypeptide gene enhancer in B-cells, kinase gamma					5.03E-03	1.742		
ITGA5	integrin, alpha 5 (fibronectin receptor, alpha polypeptide)					4.33E-02	-2.098	2.27E-02	-2.119

LMP2-with Inhibitors: NFkB Activation by Viruses (P = 1.69 x 10^{-8})

		LMP1 no Inhs		LMP2 no Inhs		LMP1 with Inhs		LMP2 with Inhs	
**Symbol**	**Entrez Gene Name**	**p-value**	**Fold Change**	**p-value**	**Fold Change**	**p-value**	**Fold Change**	**p-value**	**Fold Change**
ITGA5	integrin, alpha 5 (fibronectin receptor, alpha polypeptide)					4.33E-02	-2.098	2.27E-02	-2.119
NFKB1	nuclear factor of kappa light polypeptide gene enhancer in B-cells 1							1.63E-02	1.226
RIPK1	receptor (TNFRSF)-interacting serine-threonine kinase 1							3.39E-03	-1.61

LMP1-no Inhibitors: p53 signaling (P = 0.0184)

		LMP1 no Inhs		LMP2 no Inhs		LMP1 with Inhs		LMP2 with Inhs	
**Symbol**	**Entrez Gene Name**	**p-value**	**Fold Change**	**p-value**	**Fold Change**	**p-value**	**Fold Change**	**p-value**	**Fold Change**
PCNA	proliferating cell nuclear antigen	1.39E-02	-1.567	8.60E-01	-1.042	1.07E-01	-1.523	8.93E-02	-1.434
SERPINB5	serpin peptidase inhibitor, clade B (ovalbumin), member 5	1.15E-02	1.984	9.97E-02	1.719	5.35E-01	1.261	5.81E-01	1.299
SFN	stratifin	3.53E-02	2.029	2.01E-01	1.754	2.44E-01	-1.2	6.46E-01	-1.123

LMP2-no Inhibitors: p53 signaling (P = 0.1956)

		LMP1 no Inhs		LMP2 no Inhs		LMP1 with Inhs		LMP2 with Inhs	
**Symbol**	**Entrez Gene Name**	**p-value**	**Fold Change**	**p-value**	**Fold Change**	**p-value**	**Fold Change**	**p-value**	**Fold Change**
PML	promyelocytic leukemia	4.01E-01	-1.174	2.33E-02	-2.367	7.11E-01	1.114	4.66E-02	-1.449

LMP1-with Inhibitors: p53 signaling (P = 0.4955)

		LMP1 no Inhs		LMP2 no Inhs		LMP1 with Inhs		LMP2 with Inhs	
**Symbol**	**Entrez Gene Name**	**p-value**	**Fold Change**	**p-value**	**Fold Change**	**p-value**	**Fold Change**	**p-value**	**Fold Change**
CSNK1D	casein kinase 1, delta	1.00E+00	0	1.00E+00	0	2.53E-02	-1.754	4.94E-02	-1.485
PERP	PERP, TP53 apoptosis effector					4.94E-02	-7.403	1.00E+00	
TP53I3	tumor protein p53 inducible protein 3	2.06E-01	1.219	1.08E-01	-1.341	9.11E-04	3.243	9.31E-01	-1.022

LMP2-with Inhibitors: p53 signaling (P = 0.2889)

		LMP1 no Inhs		LMP2 no Inhs		LMP1 with Inhs		LMP2 with Inhs	
**Symbol**	**Entrez Gene Name**	**p-value**	**Fold Change**	**p-value**	**Fold Change**	**p-value**	**Fold Change**	**p-value**	**Fold Change**
CSNK1D	casein kinase 1, delta	1.00E+00	0	1.00E+00	0	2.53E-02	-1.754	4.94E-02	-1.485
FAS	Fas cell surface death receptor	1.00E+00	-1.102	1.00E+00	0	1.24E-01	-1.23	3.08E-03	-1.72
PML	promyelocytic leukemia	4.01E-01	-1.174	2.33E-02	-2.367	7.11E-01	1.114	4.66E-02	-1.449

LMP1-noInhs_CDC42 signaling (P = 0.6239)

		LMP1 no Inhs		LMP2 no Inhs		LMP1 with Inhs		LMP2 with Inhs	
**Symbol**	**Entrez Gene Name**	**p-value**	**Fold Change**	**p-value**	**Fold Change**	**p-value**	**Fold Change**	**p-value**	**Fold Change**
ARPC4	actin related protein 2/3 complex, subunit 4, 20kDa	1.11E-02	-1.511	9.32E-01	-1.01	5.30E-01	-1.042	2.83E-01	1.177

LMP2-noInhs_CDC42 signaling (P = 0.3135)

		LMP1 no Inhs		LMP2 no Inhs		LMP1 with Inhs		LMP2 with Inhs	
**Symbol**	**Entrez Gene Name**	**p-value**	**Fold Change**	**p-value**	**Fold Change**	**p-value**	**Fold Change**	**p-value**	**Fold Change**
BAIAP2	BAI1-associated protein 2	9.89E-01	-1.006	3.81E-02	-1.488	1.71E-01	1.399	7.03E-01	-1.09

LMP1 with Inhs_CDC42 signaling (P = 0.0167)

		LMP1 no Inhs		LMP2 no Inhs		LMP1 with Inhs		LMP2 with Inhs	
**Symbol**	**Entrez Gene Name**	**p-value**	**Fold Change**	**p-value**	**Fold Change**	**p-value**	**Fold Change**	**p-value**	**Fold Change**
ARPC1A	actin related protein 2/3 complex, subunit 1A, 41kDa	3.33E-01	1.922	7.89E-01	1.212	3.81E-02	1.382	2.37E-01	-1.175
CLIP1	CAP-GLY domain containing linker protein 1	7.27E-01	1.138	8.90E-02	-2.449	4.59E-03	1.901	5.89E-01	1.072
EXOC3	exocyst complex component 3	1.00E+00	0	1.00E+00	0	1.28E-02	1.561	3.09E-02	-1.456
FGD1	FYVE, RhoGEF and PH domain containing 1	1.00E+00	0	1.00E+00	0	1.73E-02	1.362	1.00E+00	-1.038
HLA-B	major histocompatibility complex, class I, B	1.00E+00	-2.445	1.00E+00	-1.504	2.06E-02	4.993	1.00E+00	1.329
IQGAP3	IQ motif containing GTPase activating protein 3	1.00E+00	0	1.00E+00	0	8.66E-03	2.33	3.17E-01	-1.12
ITGA5	integrin, alpha 5 (fibronectin receptor, alpha polypeptide)	3.64E-01	-2.172	1.00E+00	-3.726	4.33E-02	-2.098	2.27E-02	-2.119
MYL6	myosin, light chain 6, alkali, smooth muscle and non-muscle	5.11E-02	1.436	4.10E-01	1.254	1.40E-02	1.973	9.73E-03	1.755
MYL9	myosin, light chain 9, regulatory	1.00E+00	0	1.00E+00	0	4.54E-02	3.181	1.00E+00	-1.094
PAK1	p21 protein (Cdc42/Rac)-activated kinase 1					3.34E-02	-1.594	1.00E+00	

LMP2 with Inhs_CDC42 signaling (P = 0.2233)

		LMP1 no Inhs		LMP2 no Inhs		LMP1 with Inhs		LMP2 with Inhs	
**Symbol**	**Entrez Gene Name**	**p-value**	**Fold Change**	**p-value**	**Fold Change**	**p-value**	**Fold Change**	**p-value**	**Fold Change**
ACTR2	ARP2 actin-related protein 2 homolog (yeast)	3.55E-01	1.594	9.05E-01	-1.028	8.73E-01	-1.01	4.13E-02	-1.124
EXOC3	exocyst complex component 3	1.00E+00	0	1.00E+00	0	1.28E-02	1.561	3.09E-02	-1.456
EXOC5	exocyst complex component 5	1.00E+00	0	1.00E+00	0	1.52E-01	-1.403	1.07E-02	-2.131
ITGA5	integrin, alpha 5 (fibronectin receptor, alpha polypeptide)	3.64E-01	-2.172	1.00E+00	-3.726	4.33E-02	-2.098	2.27E-02	-2.119
MYL6	myosin, light chain 6, alkali, smooth muscle and non-muscle	5.11E-02	1.436	4.10E-01	1.254	1.40E-02	1.973	9.73E-03	1.755
